# Machine‐Learning‐Assisted Discovery of Mechanosynthesized Lead‐Free Metal Halide Perovskites for the Oxidative Photocatalytic Cleavage of Alkenes

**DOI:** 10.1002/advs.202309714

**Published:** 2024-05-28

**Authors:** Yonghao Xiao, Khokan Choudhuri, Adisak Thanetchaiyakup, Wei Xin Chan, Xinwen Hu, Mansour Sadek, Ying Hern Tam, Ryan Guanying Loh, Sharifah Nadhirah Binte Shaik Mohammed, Kendric Jian Ying Lim, Ju Zheng Ten, Felipe Garcia, Vijila Chellappan, Tej S. Choksi, Yee‐Fun Lim, Han Sen Soo

**Affiliations:** ^1^ School of Chemistry Chemical Engineering and Biotechnology Nanyang Technological University 21 Nanyang Link Singapore 637371 Singapore; ^2^ Departamento de Química Orgánica e Inorgánica Facultad de Química Universidad de Oviedo Julián Claveria 8 Oviedo Asturias 33006 Spain; ^3^ School of Chemistry Monash University Clayton Victoria 3800 Australia; ^4^ Institute of Materials Research and Engineering (IMRE) Agency for Science Technology and Research (A*STAR) Fusionopolis Way, Innovis #08‐03 Singapore 138634 Singapore; ^5^ Institute for Functional Intelligent Materials National University of Singapore 4 Science Drive 2 Singapore 117544 Singapore; ^6^ School of Chemistry Chemical Engineering and Biotechnology Nanyang Technological University 62 Nanyang Drive Singapore 637459 Singapore; ^7^ Cambridge Centre for Advanced Research and Education in Singapore CREATE Tower 1 Create Way Singapore 138602 Singapore; ^8^ Institute of Sustainability for Chemicals Energy and Environment (ISCE2) Agency of Science Technology and Research (A*STAR) 1 Pesek Road Singapore 627833 Singapore

**Keywords:** alkene oxidation, lead‐free perovskites, machine learning, mechanosynthesis, photocatalysis

## Abstract

Lead‐free metal halide perovskites can potentially be air‐ and water‐stable photocatalysts for organic synthesis, but there are limited studies on them for this application. Separately, machine learning (ML), a critical subfield of artificial intelligence, has played a pivotal role in identifying correlations and formulating predictions based on extensive datasets. Herein, an iterative workflow by incorporating high‐throughput experimental data with ML to discover new lead‐free metal halide perovskite photocatalysts for the aerobic oxidation of styrene is described. Through six rounds of ML optimization guided by SHapley Additive exPlanations (SHAP) analysis, BA_2_CsAg_0.95_Na_0.05_BiBr_7_ as a photocatalyst that afforded an 80% yield of benzoic acid under the standard conditions is identified, which is a 13‐fold improvement compared to the 6% with when using Cs_2_AgBiBr_6_ as the initial photocatalyst benchmark that is started. BA_2_CsAg_0.95_Na_0.05_BiBr_7_ can tolerate various functional groups with 22 styrene derivatives, highlighting the generality of the photocatalytic properties demonstrated. Radical scavenging studies and density functional theory calculations revealed that the formation of the reactive oxygen species superoxide and singlet oxygen in the presence of BA_2_CsAg_0.95_Na_0.05_BiBr_7_ are critical for photocatalysis.

## Graphical abstract

Lead‐free metal halide perovskite photocatalysts are discovered via a combination of mechanosyntheses, high‐throughput characterization, and machine learning. After 6 rounds of iterations between experiments and machine learning, a champion perovskite photocatalyst for styrene oxidation to benzoic acid in 80% yield is achieved, representing a 13‐fold improvement from the Cs_2_AgBiBr_6_ benchmark initially started with.

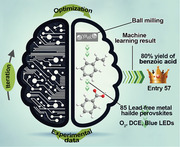

## 1 Introduction

Metal halide perovskites have dominated the attention of the energy and optoelectronics community, especially materials scientists and applied physicists, since 2009 because of their exceptional versatility and performance in many optoelectronic applications such as photovoltaics (PVs),^[^ ^1^ ^]^ light‐emitting diodes (LEDs),^[^ ^2^ ^]^ non‐linear optics,^[^ ^3^ ^]^ photodetectors,^[^ ^4^ ^]^ and lasers.^[^ ^5^ ^]^ Since Miyasaka and co‐workers first described the seminal use of perovskites methylammonium lead iodide (MAPbI_3_) as light harvesting materials for PVs,^[^ ^6^ ^]^ their power conversion efficiencies (PCEs) have reached almost 26% for single‐junction PVs in 2021,^[^ ^7^ ^]^ with even more outstanding performances for tandem cells previously achieved by more expensive III/V semiconductor materials.^[^ ^1^, ^8^ ^]^ The remarkable performances of perovskite PVs indicate that the perovskites are effective at light harvesting and charge separation, which in turn suggest that they should also be suitable as photocatalysts. Accordingly, there have been sporadic reports of metal halide perovskites being employed as photocatalysts for hydrogen evolution,^[^ ^9^ ^]^ pollutant degradation,^[^ ^10^ ^]^ and synthetic organic applications.^[^ ^11^ ^]^ Nonetheless, in stark contrast to their proliferating reports in optoelectronic applications, perovskites have remained relatively under‐explored in photocatalysis, likely because of the notorious instability of the lead and tin halide perovskites in the presence of solvents.^[^ ^12^ ^]^

Currently, the most popular and best‐performing perovskite light harvesters have been the 3D lead iodide variants containing small cations such as methylammonium (MA), formadinium (FA), cesium (Cs), and their ion mixtures.^[^ ^13^ ^]^ However, the perovskites containing these hydrophilic cations are susceptible to thermal^[^ ^14^ ^]^ and moisture^[^ ^15^ ^]^ degradation. In addition, the lead (Pb) in the commonly encountered perovskites is toxic and will add costs to end‐of‐use recycling, whereas the tin (Sn) and germanium Group 14 congeners are oxidatively unstable under aerobic conditions.^[^ ^8a^ ^]^ Moreover, the majority of the perovskite synthetic methods involve organic solvents like *N*,*N*‐dimethylformamide (DMF), which is included in the European Union's (EU) substance of very high concern (SVHC) list.^[^ ^16^ ^]^ A vexing development is that the European Commission announced regulations to restrict DMF to concentrations below 0.3% in the EU market from the end of 2023,^[^ ^16^ ^]^ which will adversely impact most of the solution‐processed perovskite synthetic procedures. The crystallinity, phase purity, and final elemental compositions are also highly sensitive to the solvent combinations used, while their complex crystallization dynamics and polymorphic transformation are poorly understood, which makes these solution‐based processes too fastidious to scale up reproducibly.^[^ ^17^ ^]^ Furthermore, the solvents contribute to the waste and greenhouse gas emissions during manufacturing.^[^ ^17^ ^]^

Lately, mechanosynthesis has re‐emerged as a greener approach to reliably and effectively access materials and molecular compounds under solvent‐free conditions at room temperature, even for metastable products that would otherwise have required harsher reaction conditions by solution‐based chemistry.^[^ ^4^, ^18^ ^]^ Mechanochemical reactions are now typically conducted with automated shaker mills or large‐scale planetary mills that consist of hardened stainless steel jars and multiple ball bearings, which are oscillated or spun at adjustable frequencies. These mechanosynthetic solid‐state milling methods circumvent the tedious and arbitrary process of discovering optimal solvents and have been successfully applied for preparing pharmaceutical cocrystals,^[^ ^18^, ^19^ ^]^ inorganic alloys and oxides,^[^ ^18a^ ^]^ metal‐organic frameworks (MOFs),^[^ ^20^ ^]^ and also lead halide perovskites.^[^ ^21^ ^]^

To overcome the above‐mentioned challenges in the use and synthesis of metal halides perovskite photocatalysts, such as their instability in solvents or aerobic environments, the toxicity of specific components or solvents, and the uncertainties in crystallization, we sought to expand the palette of materials by mechanosynthesizing a wider range of candidates including lower dimensional perovskites and double perovskites to discover more metal halide perovskites as heterogeneous photocatalysts for organic syntheses.^[^ ^22^ ^]^ Heterogeneous photocatalysts can be more readily separated and recycled, which will facilitate their adoption by the chemical industry. Lower dimensional perovskites, such as 2D and 1D variants can contain larger and more hydrophobic organic ammonium cations that are stable even in the presence of polar, protic solvents.^[^ ^11a^ ^]^ Double perovskites can be practically insoluble in organic solvents and composed of earth‐abundant and less toxic elements so that they can be deployed as reusable heterogeneous photocatalysts.^[^ ^23^ ^]^ Furthermore, we can use the tricationic bismuth(III) (Bi^III^), antimony(III) (Sb^III^), and indium(III) (In^III^) or Sn^IV^, which form stronger metal‐halide bonds than Pb^II^ and Sn^II^ because of their stronger electrostatic interactions, to prepare more thermodynamically stable perovskites.^[^ ^23^ ^]^

However, although the opportunity to synthesize an infinite number of permutations can be valuable for eventually identifying an optimal photocatalyst, it would pose synthetic and characterization challenges, which is where machine learning (ML) and high‐throughput characterization tools can be game‐changing.^[^ ^24^ ^]^ ML is a branch of artificial intelligence that thrives on using algorithms and statistical models to discover patterns and make predictions from large data sets and has been deployed for accelerated materials discovery by others^[^ ^24^, ^25^ ^]^ and our team^[^ ^26^ ^]^ recently, but remains in the embryonic stages for application‐oriented explorations.^[^ ^10^, ^24^, ^27^ ^]^ In a typical ML‐assisted experimentation workflow, a dataset is first generated that is then used to train the ML algorithm, which suggests new experimental conditions to try. However, the ML workflow we implemented here has certain different and unique elements that are worth mentioning. In our workflow, we carefully curated a set of descriptors that are based on the electronegativities of the elements at sites A, B, and X, and also the crystal space groups. Subsequently, at each iteration, we trained our ML algorithm based on these descriptors, from which we extracted the SHapley Additive exPlanations (SHAP) feature importances. These feature importance rankings, together with our chemical domain knowledge, then guide the next set of experiments.

**Figure 1:**
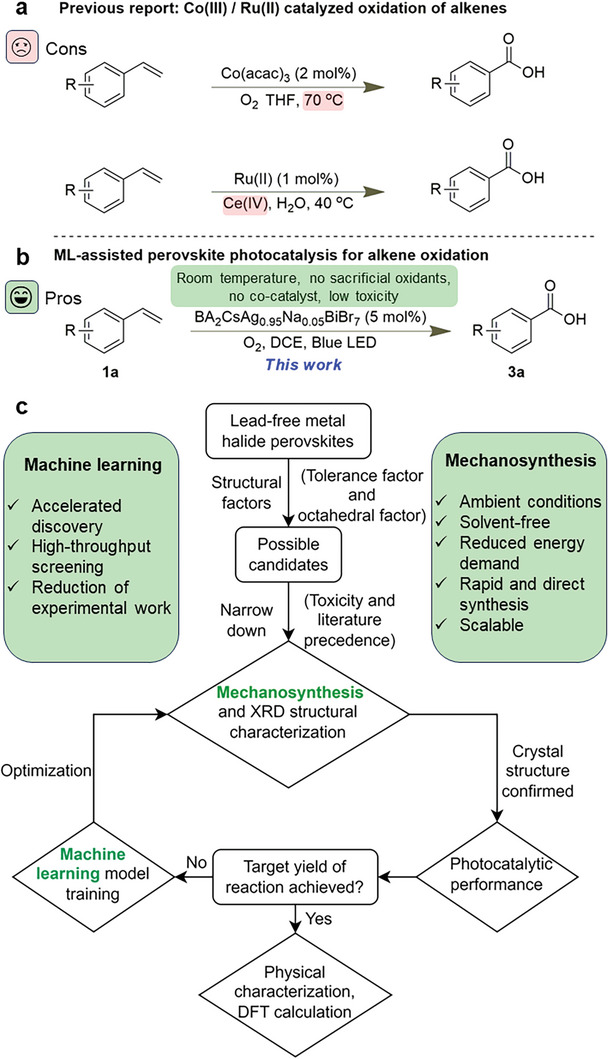
a) Previous reports on Co(III) and Ru(II) catalyzed thermal oxidation of alkenes. b) ML‐assisted perovskite photocatalysis for alkene oxidation. c) Workflow of the ML‐assisted iterative discovery of optimal metal halide perovskite photocatalysts.

Here, we describe a combined experimental and computational study to discover lead‐free metal halide perovskites that are effective, robust photocatalysts for the transformation of alkenes under oxidative conditions as a representative organic synthetic reaction. Traditionally, alkene oxidation reactions required elevated temperatures and/or harsh sacrificial oxidants such as cerium(IV) (**Figure** **1**a).^[^ ^28^ ^]^ In this work, mechanosynthesis was effectively deployed to synthesize perovskites with diverse compositions and dimensionalities without the use of solvents at room temperature. The synergistic use of ML, mechanosynthesis, and high‐throughput characterization enabled us to discover a wide range of new perovskites as photocatalysts for alkene oxidation, synthesize them reliably under solvent‐free conditions, and rapidly characterize their structural and optical properties. We integrated experimental results into our ML model to identify features of perovskites that impact the photocatalytic performance, despite the sheer number of possible permutations of perovskite compositions. Through an iterative experimental and ML workflow, we progressively identified high‐performing photocatalyst candidates that could catalyze the oxidation of styrene to benzoic acid with high selectivity and conversions under blue LEDs after 72 h. The highest‐performing perovskite photocatalyst predicted using our ML model afforded an 80% yield of benzoic acid experimentally, a 13‐fold improvement from the initial Cs_2_AgBiBr_6_ benchmark (Figure 1b), thus demonstrating the exceptional capabilities of ML for materials discovery.

## 2 Results and Discussion

### 2.1 Iterative Experimental and Machine Learning Workflow for Screening Perovskite Candidates

In our endeavor to discover optimal metal halide perovskite photocatalysts, we implemented a systematic and iterative workflow, underpinned by both experiments and computational studies. This approach ensures a comprehensive assessment of the potential candidates while optimizing their performance metrics, as shown in Figure 1c.

Preliminary screening is first conducted based on the structural parameters for stable metal halide perovskites. Specifically, the tolerance factor (TF) and octahedral factor (*µ*) are used to provide insights and predictions about the stability and potential feasibility of accessing the perovskites under consideration (Equations S2 and S3 in Supporting information). After this structural evaluation, a subset of promising candidates emerges (Table S1, Supporting Information). This subset is further scrutinized by considering existing literature precedents and the toxicity of the elements so that the chosen materials will be more practical with fewer hazards. We then prepare the selected candidates by ball‐milling mechanosynthesis, a process that is usually conducted solvent‐free and can produce the perovskites in the desired phase homogeneity and purity. Subsequently, X‐ray diffraction (XRD) analysis is used to validate the phase composition and crystalline nature of the synthesized perovskites Then, a critical phase of our workflow is the assessment of the photocatalytic performance of the candidates to ensure that the material(s) can not only harness and transform photon energy efficiently but is also functional.

With the accumulated experimental data in hand, we create and train our machine learning models (vide infra). These models facilitate the predictive analytics for further optimizations. The workflow is iterative in nature and hinges on the achievement of predefined reaction yield targets. If we meet or exceed the yield target, the workflow will progress to an advanced stage that encompasses detailed physical characterization of the materials. This step offers atomic‐level insights into the material's electronic structure and potential performance enhancers. However, in scenarios where the target yield remains elusive, the workflow returns to an earlier phase using the newly synthesized and evaluated perovskites as part of the training set to facilitate re‐evaluation and optimization. This workflow offers an integrative and feedback‐driven approach to the discovery and optimization of functional metal halide perovskite photocatalysts. Through a blend of experimental rigor and machine learning, it can lead to the identification of next‐generation materials with superior photocatalytic efficiencies. Using the optimal metal halide perovskite, we will then generalize the substrate scope and conduct more detailed mechanistic studies in line with traditional catalyst optimization approaches.

### 2.2 Synthesis and High‐Throughput Characterization of Metal Halide Perovskites

As mentioned above, after the preliminary screening, we identified 18 metal halide perovskites to be synthesized. In a typical synthesis, the precursor chemicals were weighed in a glovebox filled with inert N_2_ at room temperature since many of the metal halides are hygroscopic or oxidatively unstable even if the perovskite products are air‐stable. Then, for each perovskite, the reagents in the precise stoichiometric ratios were placed in a 10 mL zirconia milling jar. This assembly was hermetically sealed and then taken out from the glovebox, installed in a mixer mill, and ground at a frequency of 30 Hz for various time durations as detailed in the Supporting Information. After the initial group of perovskites were screened for their photocatalytic performances, new perovskites were proposed based on the insights from the machine learning model. All the lead‐free perovskites that we explored with the assistance of ML and successfully synthesized by ball milling have been categorized and are shown in **Table** **1**. The perovskites marked in black, red, blue, green, pink, and orange correspond to those added to the ML dataset in the first to the sixth rounds, respectively. Starting from the second round, the syntheses of each round of perovskites were guided by the ML results from all samples in the preceding rounds. After obtaining the experimental yields from the photocatalytic reactions using the synthesized perovskites, the ML database was expanded to generate predictions for the next round, thus continuing the cycle. The complete set of XRD, UV‐vis absorption, and photoluminescence (PL) characterization data can be found in the Supporting Information as detailed below.

**Table 1:** Metal halide perovskites synthesized and characterized in this study.

3D perovskites				2D double perovskites [layer number, *n*]					
No.	Single	No.	Double	No.	*n* = 1	No.	*n* = 2	No.	*n* = 3
1.	Cs_3_BiCl_6_	20.	K_2_CsBiCl_6_	47.	PA_4_AgBiBr_8_	50‐52.	EA_2_CsAg_n_Na_1‐n_BiBr_7_ ^d^ ^)^	79‐85.	Cs_4_Cu_t_Mn_1‐t_Sb_2_Cl_12_ ^j^ ^)^
2.	Cs_3_Bi_2_Cl_9_	21.	Rb_2_AgBiCl_6_	48.	BA_4_AgInCl_8_	53‐55.	PA_2_CsAg_o_Na_1‐o_BiBr_7_ ^e^ ^)^		
3.	Cs_3_Bi_2_Br_9_	22‐32.	Cs_2_Ag_k_Na_1‐k_BiCl_6_ ^a^ ^)^	49.	BA_4_AgBiBr_8_	56‐60.	BA_2_CsAg_p_Na_1‐p_BiBr_7_ ^f^ ^)^		
4.	Cs_3_Bi_2_I_9_	33‐38.	Cs_2_AgBiBr_l_Cl_6‐l_ ^b^ ^)^			61–67.	BA_2_CsAgBiBr_q_I_7‐q_ ^g^ ^)^		
5.	Rb_3_Bi_2_Br_9_	39‐44.	Cs_2_Ag_m_Na_1‐m_InCl_6_ ^c^ ^)^			68.	BA_2_CsAg_0_._95_Na_0_._05_BiBr_6_I		
6.	MA_3_Bi_2_Cl_9_	45.	Cs_2_KInCl_6_			69.	BA_2_CsAg_0_._95_Na_0_._05_BiCl_6_Br		
7.	MA_3_Bi_2_I_9_	46.	Cs_2_AgSbCl_6_			70.	BA_2_CsAg_0_._95_K_0_._05_BiBr_7_		
8.	FA_3_Bi_2_Cl_9_					71.	BA_2_CsAg_0_._95_Cu(I)_0_._05_BiBr_7_		
9.	FA_3_Bi_2_Br_9_					72.	BA_2_CsAg_0_._95_Li_0_._05_BiBr_7_		
10.	FA_3_Bi_2_I_9_					73.	BA_1_._5_Cs_1_._5_Ag_0_._95_Na_0_._05_BiBr_7_		
11.	Cs_3_Sb_2_Br_9_					74–75.	PEA_2_CsAg_r_Na_1‐r_BiBr_7_ ^h^ ^)^		
12.	Cs_3_Sb_2_I_9_					76–78.	BA_2_CsAg_s_Na_1‐s_SbBr_7_ ^i^ ^)^		
13.	Rb_3_Sb_2_Br_9_								
14.	Rb_3_Sb_2_I_9_								
15.	MA_3_Sb_2_Br_9_								
16.	MASnCl_3_								
17.	FASnCl_3_								
18.	FASnBr_3_								
19.	FASnI_3_								

^a)^ k = 0, 0.05, 0.1, 0.2,0.4, 0.5, 0.6, 0.8, 0.9, 0.95, 1;

^b)^ l = 1, 2, 3, 4, 5, 6;

^c)^ m = 0, 0.2, 0.4, 0.6, 0.8, 1;

^d)^ n = 1, 0.95, 0.9;

^e)^ o = 1, 0.95, 0.9;

^f)^ p = 1, 0.95, 0.9, 0.75, 0.25;

^g)^ q = 0, 1, 2, 3, 4, 5, 6;

^h)^ r = 1, 0.95;

^i)^ s = 1, 0.95, 0.9;

^j)^ t = 0, 0.2, 0.4, 0.5, 0.6, 0.8, 1;

where PA is *n*‐propylammonium, BA is *n*‐butylammonium, EA is ethylammonium, and PEA is phenethylammonium. The perovskites marked in black, red, blue, green, pink, and orange correspond to those added to the ML library from the first to the sixth rounds, respectively.

XRD was extensively used to verify the crystal structure and phase purity of the lead‐free perovskites. An auto‐sampler was used to accelerate the process of structurally characterizing the perovskite samples. The diffractometer has a batch sample automatic loader, which allows us to automatically examine up to 12 samples at one time. In the preliminary round of synthesizing the lead‐free double perovskites, we not only successfully synthesized six double perovskites (No. 1, 2, 10, 19, 31, 61 in Table S1, Supporting Information), but also prepared 11 single perovskites by ball‐milling mechanosynthesis (No. 3, 4, 5, 20, 21, 32, 33, 34, 50, 51, 52 in Table S1, Supporting Information). This formed our initial library of perovskite candidates. To further expand the library, we extended to mixed‐ion double perovskites during the photocatalysis and ML iterative cycles.

**Figure 2:**
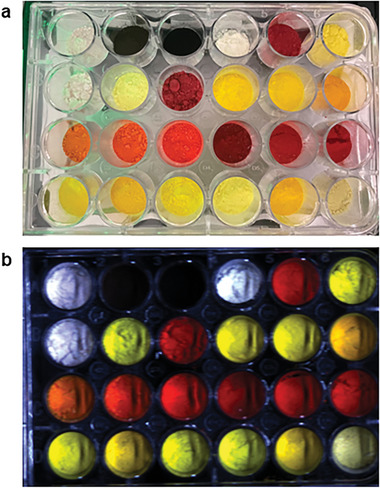
a) Photograph and b) the corresponding raw hyperspectral image from the Spectronon Pro software of a typical 24‐well plate containing the perovskite samples used for the high‐throughput spectroscopic characterization.

Likewise, to cope with the large number of samples, we used a Resonon Pika L hyperspectral imaging camera and an automated Tecan Infinite 200 Pro spectrometer together with the samples in 24‐well plates (**Figure** **2**a) to obtain high‐throughput PL and UV–Vis absorption spectral data of all the perovskites. In a typical experiment, ≈500 mg of each perovskite was placed in one of the wells until all 24 wells were filled. After some basic calibration and signal optimization, the reflectance of the samples was collected by the hyperspectral imaging camera within 5 s. The reflectance data was first preliminarily processed by the Spectronon Pro software (Figure 2b) and then transformed and normalized to absorption data using a Kubelka‐Munk function. The band gap of each sample was obtained by taking the tangents in the Tauc plots. In total, nine plates of data were collected, of which some were later removed from further studies since the XRD measurements showed that the samples were impure or not of the expected phase. The UV‐vis absorption data of all 85 samples can be found in Figures S1–S47 in the Supporting Information. Subsequently, we were able to perform high‐throughput PL measurements on the perovskite samples in the same 24‐well plates with a Tecan Infinite 200 Pro spectrometer integrated into a Zinsser Lissy automated synthetic platform. Using 370 nm excitation, the PL data for the 85 samples were collected in four batches of 24‐well plates with the data collected in the 400–800 nm range. An acquisition time of 20 µs was standardized so that the samples most strongly emissive would not saturate the detector and could still be collected during the same measurement. The data collection for each 24‐well plate could be completed within 50 min. Among the 85 samples that we examined in total, 58% of them were not emissive or poorly emissive, 16% were moderately emissive, and the remaining 26% appeared to be highly emissive. The PL data for the synthesized perovskites are displayed in Figures S1–S47 in the Supporting Information. This workflow enabled us to rapidly synthesize and obtain functional information about the perovskite samples, which facilitated our subsequent photocatalysis and ML studies.

### 2.3 Perovskite‐Photocatalyzed Oxidation of Alkenes

The oxidative cleavage of alkenes is widely employed in organic synthesis for converting economical alkene feedstocks into valuable compounds by creating oxygen‐containing groups such as ketones, aldehydes, and carboxylic acids. Although there are established traditional methods for the oxidative cleavage of alkenes like ozonolysis, it is hindered by the need for an O_3_ generator and the health and environmental hazards associated with O_3_. Hence, there has been a revival in interest for O_3_‐free ozonolysis‐like reactions as illustrated by the seminal work from Leonori^[^ ^29^ ^]^ and Parasram,^[^ ^30^ ^]^ but stoichiometric reagents or the use of UV radiation are often necessary. Recently, transition

Metal catalysis has emerged as an alternative to traditional ozonolysis for alkene oxidative cleavage, eliminating the need for O_3_.^[^ ^31^ ^]^ Nevertheless, drawbacks include the potential for partial oxidation or the necessity of harsh conditions with hazardous oxidants. In this context, we chose to apply the metal halide perovskites derived from our ML studies for the challenging oxidative cleavage of alkenes to showcase how we can discover air‐stable, photocatalytic, and lead‐free perovskites using our integrated experimental‐ML workflow.

We started optimizing the alkene oxidation reactions using styrene (1a, 0.522 mmol) as the representative substrate (**Table** **2**) and Cs_2_AgBiBr_6_ as a known, lead‐free perovskite photocatalyst. Initially, only 20% of benzoic acid (3a) was observed in the presence of 5 mol% Cs_2_AgBiBr_6_ as the photocatalyst in 1,2‐dichloroethane (DCE) under 50 W white LED irradiation and an O_2_ atmosphere after 72 h (Table 2, entry 1). When we screened other solvents such as acetonitrile and dichloromethane, the product yields did not improve substantially (Table 2, entries 2 and 3).

**Table 2:** Optimization of the conditions for the photocatalytic oxidation of styrene to benzaldehyde and benzoic acid with Cs_2_AgBiBr_6_ as a representative photocatalyst.

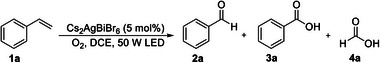					
Entry	Catalyst [5 mol %]	Solvent	50 W LED	2a [%]	3a [%]
1.	Cs_2_AgBiBr_6_	DCE	White LED	4	20
2.	Cs_2_AgBiBr_6_	CH_2_Cl_2_	White LED	10	0
3.	Cs_2_AgBiBr_6_	CH_3_CN	White LED	4	0
4.	Cs_2_AgBiBr_6_	Neat	White LED	14	18
5.	Cs_2_AgBiBr_6_	Neat	Blue LED	28	45
6.	Cs_2_AgBiBr_6_	Heptane	Blue LED	6	8
7.	Cs_2_AgBiBr_6_	EtOAc	Blue LED	12	0
**8**.	**Cs_2_AgBiBr_6_**	**DCE**	**Blue LED**	**20**	**4**
9.	Cs_2_AgBiBr_6_ ^a^ ^)^	DCE	Blue LED	14	2
10.	none	DCE	Blue LED	0	0
11.	Cs_2_AgBiBr_6_	DCE	Dark	0	0
12^c^.	Cs_2_AgBiBr_6_ ^b^ ^)^	DCE	Blue LED	0	0

Reaction conditions: Styrene (60 µL, 0.522 mmol), Cs_2_AgBiBr_6_ (27.5 mg, 0.026 mmol), solvent (3 mL), O_2_ (1 atm), room temperature, 50 W LED; Yield was determined by ^1^H NMR spectroscopy using 1,1,2,2‐tetrachloroethane as an internal standard;

^a)^ 10 mol% of Cs_2_AgBiBr_6_ was used;

^b)^ Ar atmosphere; EtOAc: ethyl acetate; DCE: 1,2‐dichloroethane.

Subsequently, we attempted the photocatalytic reactions under neat conditions and found that exposure to white light resulted in the formation of 14% benzaldehyde (**2a**) and 18% benzoic acid (**3a**) (Table 2, entry 4), while the use of a blue LED resulted in 28% **2a** and 45% **3a** (Table 2, entry 5). In both reactions, we observed that most of the remaining 1a underwent polymerization to form polystyrene. Thus, although the yields of 2a and 3a were high, we decided that solvent‐free conditions were unsuitable. Interestingly, when the photocatalysis was performed in DCE under O_2_ with a 50 W blue LED and 5 mol% Cs_2_AgBiBr_6_ as the photocatalyst (Table 2, entry 8), although only 20% 2a and 4% 3a were observed, the ^1^H NMR spectrum of the crude reaction mixture appeared to show no other side products, which was promising (Figure S55, Supporting Information). Hence, we opted to use blue LEDs for subsequent experiments since the use of white LEDs under identical reaction conditions led to more undesired side products, (Figure S49, Supporting Information). Moreover, the yield of 3a decreased when the Cs_2_AgBiBr_6_ loading was doubled to 10 mol% (Table 2, entry 9), possibly because of insufficient light penetration. In control experiments in the absence of Cs_2_AgBiBr_6_, light, or O_2_, no 2a or 3a was detected (Table 2, entries 10–12 respectively), indicating that each of these plays a vital role in the photocatalytic oxidative cleavage of **1a**. Thus, we chose the conditions from entry 8 as the starting point for further catalyst screening studies.

### 2.4 Application and Testing of Machine Learning Model

**Figure 3:**
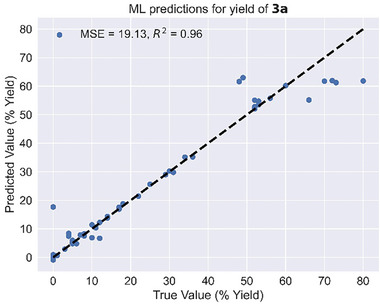
The predicted versus the true values of the benzoic acid yields obtained using our ML model based on the sixth‐round dataset. The mean squared error (MSE) and R^2^ are used as indicators of the ML performance.

An ML model was constructed to predict the desired output, the yield of **3a**, from the curated features. First, we constructed ML parameters using the basic ABX_3_ chemical formula unit of halide perovskites. We defined A_m_ (m = 1, 2), B_n_ (*n* = 1 to 4), and X_o_ (o = 1, 2) as the electronegativities of the elements at sites A, B, and X, respectively, with different subscripts representing the values based on the following formulae: (A_m_)_3_B_n_(X_o_)_6_, (A_m_)_3_(B_n_)_2_(X_o_)_9_, (A_m_)_2_(B_n_)_2_(X_o_)_6_, (A_m_)_3_(B_n_)_2_(X_o_)_7_, and (A_m_)_4_(B_n_)_3_(X_o_)_12_. In many cases, the A, B^I^, B^III^, and X sites were each evaluated with up to two species. Thus, A_10_, B_10_, and X_10_ were designated to represent the percentage of the minor constituents in A_m_, B_n_, and X_o_, respectively. Some examples of the electronegativity definitions for the ML model that we used are shown in Table S4, Supporting Information. Then, we introduced the crystal space group and the yield of 3a as additional features. Since the space group is non‐numerical, it was treated as categorical data and converted for input into the ML model. The ML model is a dense neural network, implemented using the Keras Python package. Hyperparameter tuning was performed via Bayesian Optimization using the Scikit‐Optimize package, following which each **3a** yield was predicted individually using leave‐one‐out‐cross‐validation (LOOCV). The hyperparameter tuning and LOOCV method are especially effective in overcoming the limitations of our relatively small dataset after we considered a comprehensive range of parameters, including regularizers and dropout. A few common ML algorithms were tested, including linear regression, gradient boosting, random forest, and neural networks. The first three are implemented using the Scikit‐Learn Python package, but we ultimately chose the neural network algorithm because of the highest accuracy of its LOOCV predictions (Figure S226, Supporting Information). The predicted versus actual experimental values from the final round are plotted as shown in **Figure** **3**. Notably, when the final round of data was processed using the four different ML algorithms

(Figure S227, Supporting Information), the results mirrored those from the first round, thus validating that the neural network algorithm is the most effective in this study. A high R^2^‐score of over 0.96 was achieved, indicating the accuracy of the ML predictions.

In order to extract interpretability from the ML predictions, we used the SHAP, which is a unified approach for extracting the importance of input features for model predictions.^[^ ^32^ ^]^ The SHAP approach is very general and independent of the choice of ML model. The features can be ranked by their SHAP values to indicate the most important features that contribute to the model predictions. Based on the SHAP feature importance, we used the insights to choose the next iteration of photocatalyst compositions.

**Figure 4:**
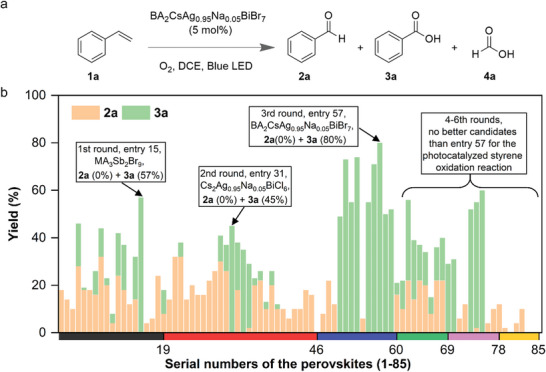
a) Photocatalyzed oxidation of **1a** to **2a**, **3a**, and **4a** using our optimal perovskite BA_2_CsAg_0.95_Na_0.05_BiBr_7_ (BA = *n*‐butylammonium). b) Yields of **2a** and **3a** from the photocatalyzed oxidation of **1a** by screening different perovskites through six iterative rounds of ML. The serial numbers of the perovskites match those shown in **Table** **1**. The label colors correspond to those in **Table** **1** and also to the perovskite candidates newly added to the ML dataset from the first to the sixth rounds.

When we began the initial rounds of machine learning, we examined a series of 1–19 perovskite catalysts in the first round of experiments, utilizing DCE as the solvent and visible light as the activation source (**Figure** **4**a). Several crucial observations emerged from these reactions. Notably, perovskites containing I (entries 4, 10, 12, 19) exhibited a tendency to decompose during the three‐day irradiation, although some product formation was observed. This suggested a limitation in the oxidative stability of the I‐containing perovskite photocatalysts under our applied conditions. Although the Cl‐containing perovskites (entries 1, 2, 6, 8, 16, 17) were stable, the product yields were generally lower compared to those with Br (entries 3, 5, 9, 11, 13, 15, 18). In the first round, FA_3_Bi_2_Cl_9_ led to the highest yield of **2a** (32%), whereas MA_3_Sb_2_Br_9_ delivered 57% of **3a**. After incorporating the yields of **3a** from the oxidation of styrene by these 19 perovskites into the ML dataset, the SHAP analysis suggested that medium values of X_1_ and X_2_ (electronegativities of the halides) showed better performances, meaning that the Br among the three halides was most suited in the photocatalyst to oxidize **1a** to **3a**. This is further corroborated by the observation that small X_10_ values (the % of the minor halide component) led to higher yields of **3a**, affirming that pure bromides are more effective than any mixed halide perovskites.

In the second round of our ML screening, we expanded to 3D double perovskites (entries 20–46). For the B^I^ site, we explored using two similarly sized mono‐cations B_1_ and B_2_. Combining the yield values of **3a** from the new 3D double perovskites into the original ML model, we found that a low B_2_ value led to higher

Yields based on the SHAP analysis. This guided us to select Na (electronegativity: 0.93) for B_2_ and Ag (1.93) for B_1_. An intriguing observation emerged when introducing a nominal amount (5%) of Na into the B^I^ site. This modification significantly enhanced the photocatalytic efficacy (entry 31), leading to an impressive 45% yield of **3a**. Additionally, a small B_10_ was found to enhance the **3a** yield, suggesting that the percentage of B_2_ should be kept at a low value, which led to our adoption of a 5% Na content. When we explored mixed halide perovskites (entries 33–37), the yields of **2a** or **3a** were modest, in line with our expectations from the first round of ML.

Subsequently, based on the results from the first two rounds of ML, we further introduced different A‐site cations to seek higher‐yielding lead‐free perovskite photocatalysts. When we examined the effects of two different A‐site mono‐cations on the yields of **3a** in a third round of ML, we found that higher A_1_ values (electronegativity of the A‐site cation) were beneficial, indicating that organic ammonium cations that have more electronegative elements than alkali metal cations are suitable for the A_1_ site. However, high values of A_2_ were detrimental, necessitating the introduction of alkali metal cations with low electronegativities as the minor components. Accordingly, we engineered a series of 2D double perovskites (entries 47–60) by varying the Na concentrations in the B_2_ sites.

We found that BA_2_CsAg_0.95_Na_0.05_BiBr_7_ with the more electronegative BA cation (relative to alkali metal cations) as the major A site component exhibited a remarkable 80% yield of **3a** under our standard conditions (Figure 4b). This represents an over 13 times improvement in the yield of **3a** from when we started with Cs_2_AgBiBr_6_ (6%). We attempted to achieve even higher yields through three additional rounds of ML. We sought to replace the B^I^ site Na with a less electronegative alkali metal (entries 70–72), vary the organic cations in the A sites, and substitute Sb for Bi in the B^III^ site, but none led to a higher yield of **3a**. Thus, it appeared that the yield improvements from the ML studies had plateaued without additional feature inputs.

**Figure 5:**
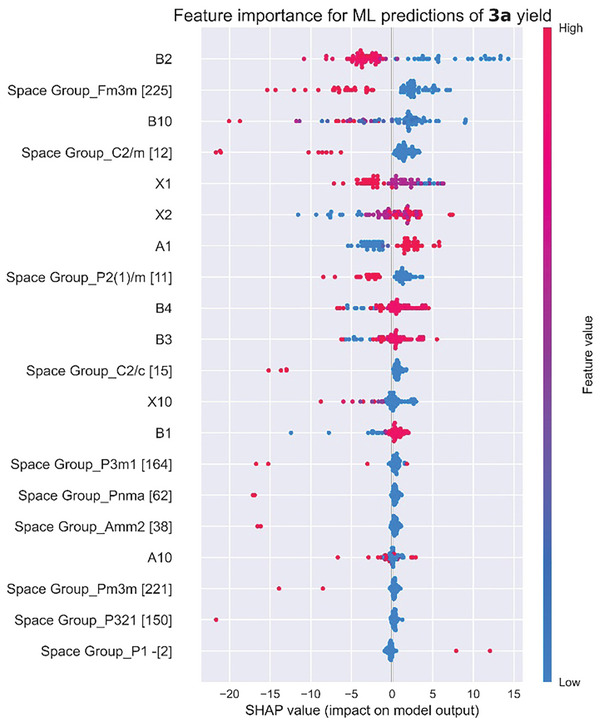
The SHAP derived from six rounds of ML on 85 perovskite samples, highlighting the most important features influencing the yields of **3a**.

After six rounds of ML on the perovskite candidates, **Figure** **5** shows the input features sorted by the final SHAP values, which indicated that the lead‐free metal halide perovskites suitable for the oxidative production of **3a** from **1a** must meet several criteria, starting with a low B_2_ and low B_10_, which means that the electronegativity of the element in B_2_ should be lower than that in B_1_ and the % of the B_2_ element should be kept at a low level. Second, a high A_1_ value, along with medium X_1_ and X_2_ values, are required. Guided by this SHAP analysis, we were able to discover the multiple new perovskite photocatalysts described above in an iterative process.

To investigate the stability of the perovskites, we examined three photocatalysts that showed respectable yields of **3a** (See Supporting Information, Table S3, Supporting Information), namely entry 31 Cs_2_Ag_0.95_Na_0.05_BiCl_6_ (45%), entry 57 BA_2_CsAg_0.95_Na_0.05_BiBr_7_ (80%), and entry 75 PEA_2_CsAg_0.95_Na_0.05_BiBr_7_ (60%). We used each of them for the photocatalytic oxidation of 1a and after 72 h, we collected the precipitates after centrifugation and performed XRD measurements on them. As shown in FigureS232, Supporting Information, the XRD patterns of Cs_2_Ag_0.95_Na_0.05_BiCl_6_ and PEA_2_CsAg_0.95_Na_0.05_BiBr_7_ appeared mostly similar to the samples before the photocatalytic reactions. However, the diffraction peaks of BA_2_CsAg_0.95_Na_0.05_BiBr_7_ had changed significantly (Figure S232c, Supporting Information) and appeared remarkably similar to the pattern for Cs_2_Ag_0.95_Na_0.05_BiCl_6_, suggesting that BA_2_CsAg_0.95_Na_0.05_BiBr_7_ may have lost the organic ammonium cations and converted into a 3D double perovskite with mixed halides (Figure S232d, Supporting Information). We selected both 2D perovskite samples BA_2_CsAg_0.95_Na_0.05_BiBr_7_ and PEA_2_CsAg_0.95_Na_0.05_BiBr_7_ that had been collected by centrifugation and subjected them to a second round of photocatalysis for another 72 h. Interestingly, although BA_2_CsAg_0.95_Na_0.05_BiBr_7_ had appeared to transform into a different perovskite phase, we observed a 40% yield of 3a (Figure S118, Supporting Information). On the other hand, for the second round of photocatalysis using PEA_2_CsAg_0.95_Na_0.05_BiBr_7_, we observed merely a 4% yield of 3a (Figure S136, Supporting Information), despite the fact that the XRD pattern suggested the perovskite had remained intact. These experiments reflect the limitations of ML in predicting performances beyond the boundaries of the experimental inputs.

### 2.5 Alkene Substrate Scope

**Figure 6:**
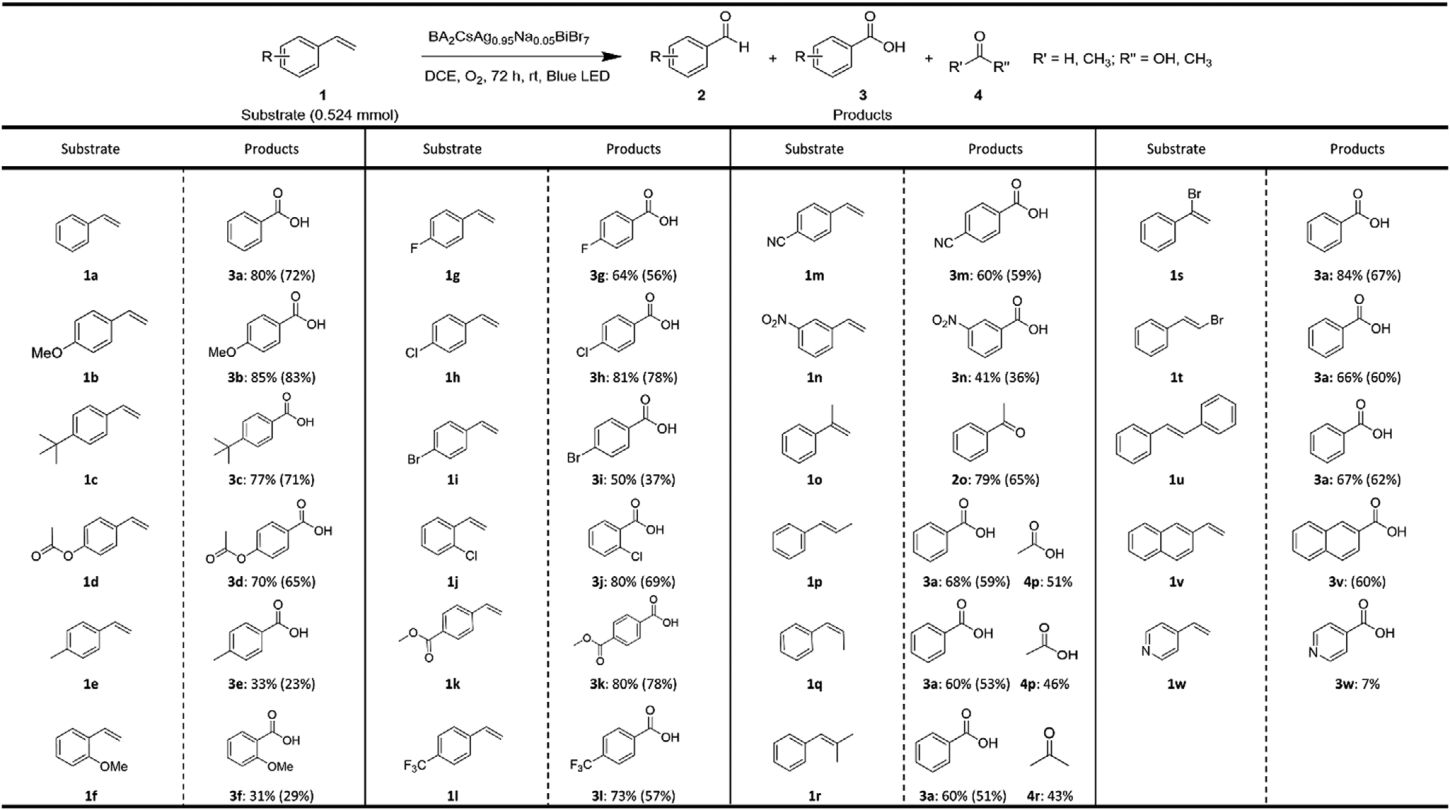
Substrate scope for the photocatalytic oxidation of alkenes under the optimized reaction conditions: BA_2_CsAg_0.95_ Na_0.05_BiBr_7_ (0.030 g, 0.026 mmol, 5 mol%), 3 mL DCE, one 50 W blue LED, O_2_ (1 atm), room temperature. The NMR yields are shown and the isolated yields are given in parentheses.

After we had screened through the 85 perovskite candidates for the photocatalyzed oxidation of the representative substrate **1a**, we expanded the substrate scope to 22 other related aryl alkenes; we applied the same optimized conditions using the highest yielding BA_2_CsAg_0.95_Na_0.05_BiBr_7_ to examine if the performance could be generalized (**Figure** **6**). In all the photocatalytic experiments for each substrate, the reactions were conducted at least twice and typically thrice.

To assess the functional group tolerance and the stability of the perovskite, we used derivatives of **1a** by introducing various substituents on both the aryl ring and vinyl group. Gratifyingly, most of the substrates underwent highly selective, quantitative conversions and yielded carboxylic acid or carbonyl derivatives in moderate to high yields in the presence of a broad range of both electron‐donating and electron‐withdrawing functional groups as shown in Figure 6. In addition, the results indicated that the steric and electronic effects of the substituents on both the aryl ring and the vinyl group of **1a** can impact the selectivities and yields of the products.

Substrates with electron‐donating groups in the *para* position such as methoxy, *tert*‐butyl, acetoxy, and methyl were well‐tolerated, achieving the highest yield of 85% for **3b**, 77% for **3c**, and 70% for **3d** (Figure 6). Benzoic acids bearing *para*‐methyl and *ortho*‐methoxy groups were produced in lower yields (**3e**: 33%; **3f**: 31%). Interestingly, substrates with electron‐deficient substituents such as halogens, ester, trifluoromethyl, cyano, and nitro groups were also tolerated to produce the corresponding carboxylic acids (**3g‐** **n**) in moderate to high yields (41‐81%). Notably, the substrates with the weakly electron‐deficient *para*‐chloro, *ortho*‐chloro, and *para*‐methyl ester substituents still gave fairly high yields (81% for **3** **h** and 80% for **3j** and **3k** respectively). However, the *meta*‐nitrostyrene gave a modest yield of only 41% for **3n**, likely owing to the stronger electron‐withdrawing effects.

In addition, substrates with methyl substituents on the vinyl group (**1o**‐**1r**), including the sterically demanding *cis*‐position, reacted readily under the standard conditions to provide moderate to high yields of the expected products, such as acetophenone (**2o**; 79%), **3a** (60%–68%), acetic acid (**4p**; 46%–51%), and acetone (**4r**; 43%). Products **4p** and **4r** were confirmed by ^1^H and ^13^C NMR spectroscopy, and the yields were calculated from the NMR spectra of the crude reaction mixture (see Supporting Information, Figures S163–S167, Supporting Information). The mechanisms of forming 2o, 4p, and 4r are consistent with the general mechanism obtained from our computational insights below and they arise because of the additional methyl substituents on the styrene. Likewise, substrates with electron‐deficient Br on the vinyl group, 1s, and 1t, achieved high and moderate yields of 3a (84% and 66% respectively). Stilbene was oxidized to 67% **3a**, taking into account that two equivalents would be produced for every equivalent of stilbene. In addition, 2‐vinylnaphthalene (**1v**) containing a fused ring likewise reached full conversion and moderate product yields (**3u**, 60% isolated yield). With 4‐vinyl pyridine (**1w**), a low 7% yield of isonicotinic acid (**3w**) was observed, possibly because the pyridine nitrogen may exchange with the cations on the surface of the perovskite. Overall, the reasonable substrate scope indicated that the new BA_2_CsAg_0.95_ Na_0.05_BiBr_7_ photocatalyst we discovered via ML for **1a** oxidation is not an anomaly but is indeed applicable for a diverse range of related aryl alkenes with electron‐rich, electron‐deficient, and even sterically demanding substituents.

### 2.6 Mechanistic Studies

**Figure 7:**
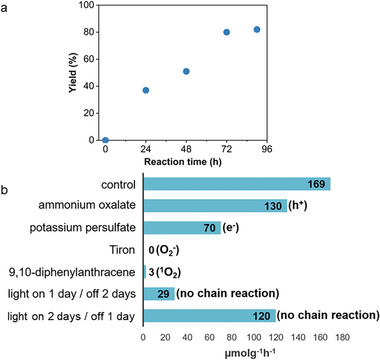
a) Yields of **3a** recorded over 90 h using blue LED irradiation. b) Mechanistic studies by radical scavenging and light on/off experiments to probe the role of different reactive oxygen species and light. The involved reactive oxygen species and the absence of a chain reaction are shown in parentheses.

To gain insights into the kinetics of the photocatalytic oxidation of **1a** into **3a**, the reaction progress under standard conditions was monitored over 90 h (**Figure** **7**a). The yield of **3a** showed a near‐linear increase with reaction time until it reached a maximum of 80% after 72 h. Prolonging the reaction time beyond 90 h did not result in a significant change to the yield of **3a**. We then performed a series of experiments under the optimized conditions in the presence of separately added radical scavengers. This included ammonium oxalate for hole scavenging, potassium persulfate for electron scavenging, Tiron for superoxide scavenging, and 9,10‐diphenylanthracene for singlet oxygen scavenging (Figure 7b). The stability of BA_2_CsAg_0.95_Na_0.05_BiBr_7_ after being suspended with each radical scavenger was first examined to ensure that the perovskite was stable in the presence of the scavengers and that the change in the photocatalytic reaction yields did not arise from decomposition BA_2_CsAg_0.95_Na_0.05_BiBr_7_ (Scheme S1, Supporting Information).

In the hole scavenging experiments with ammonium oxalate, the reaction rate decreased slightly compared to the optimized conditions in the control experiment (Figure 7b). Moreover, in the presence of potassium persulfate, the formation rate of **3a** was even slower. However, the use of Tiron completely inhibited the photocatalytic formation of **3a**. This result indicated that superoxide is indispensable in the photocatalytic oxidative cleavage reaction. Similarly, 9,10‐diphenylanthracene also strongly inhibited the reaction, likewise suggesting that singlet oxygen is also vital in this reaction. Light on/off experiments were conducted to evaluate if BA_2_CsAg_0.95_Na_0.05_BiBr_7_ was just a photoinitiator for a chain reaction in the dark. When the reaction was irradiated for one day and stirred in the dark for the next two days, the production of **3a** ceased after the light was turned off. Likewise, when the reaction was irradiated for two days, the yield increased above the one‐day irradiation experiment but was still lower than that of the control experiment, which confirmed that continuous irradiation is necessary for BA_2_CsAg_0.95_Na_0.05_BiBr_7_ to serve as a photocatalyst.

We also compared the ^1^O_2_ production rates of Cs_2_AgBiBr_6_ and BA_2_CsAg_0.95_Na_0.05_BiBr_7_ by using 9,10‐diphenylanthracene to trap ^1^O_2_ and generate 9,10‐diphenyl‐9,10‐epidioxyanthracene, which can be detected by ^1^H NMR spectroscopy (Figures S147 and S148, Supporting Information). After irradiating each perovskite and 9,10‐diphenylanthracene under O_2_ gas for two h, the spectra show that BA_2_CsAg_0.95_Na_0.05_BiBr_7_ can generate ^1^O_2_ from ^3^O_2_ faster than Cs_2_AgBiBr_6_ (67% vs 57%). Moreover, after six h of irradiation, the 9,10‐diphenyl‐9,10‐epidioxyanthracene from the BA_2_CsAg_0.95_Na_0.05_BiBr_7_ reaction was over oxidized, while the reaction with Cs_2_AgBiBr_6_ still showed some unreacted 9,10‐diphenylanthracene. These experiments allude to the faster‐photoexcited charge transfer and ^1^O_2_ generation abilities of BA_2_CsAg_0.95_Na_0.05_BiBr_7_ relative to Cs_2_AgBiBr_6_, which we confirmed using DFT calculations in the next section.

### 2.7 Computational Insights on the Photocatalysis

The results from the mechanistic studies suggest that both superoxide and singlet oxygen are indispensable for this photocatalytic reaction. Subsequently, we performed a density functional theory (DFT) study to determine the feasibility of forming singlet oxygen after a triplet oxygen molecule adsorbs on the surface of a model of BA_2_CsAg_0.95_Na_0.05_BiBr_7_. The spin polarization maps of the oxygen molecule before and after adsorption suggest that the triplet spin of the gas phase oxygen molecule has decreased upon adsorption (Figure S228, Supporting Information), consistent with the formation of singlet oxygen.

**Scheme 1:**
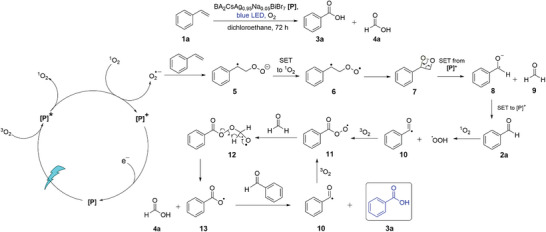
Proposed mechanism for the BA_2_CsAg_0.95_Na_0.05_BiBr_7_ photocatalyzed oxidative cleavage of **1a**.

Since the reaction involves multiple steps, there are several plausible pathways for the mechanism. Using our experimental results from the mechanistic studies and DFT calculations, which were performed at the B3LYP/6‐311++(d,p) level to obtain the Gibbs free energies of the possible reaction intermediates (Figure S229, Supporting Information), we propose one of the possible mechanisms as shown in **Scheme** **1**. We postulate that singlet oxygen is first generated by energy transfer from the photoexcited BA_2_CsAg_0.95_Na_0.05_BiBr_7_ and another photoexcited BA_2_CsAg_0.95_Na_0.05_BiBr_7_ will also separately transfer a conduction band electron to singlet or triplet oxygen to produce superoxide (Scheme 1). Superoxide may also be generated by electron transfer from reaction intermediates to singlet oxygen. These proposed multiple pathways for superoxide formation are more consistent with the electron scavenging experiments by potassium persulfate, since the photocatalytic reaction when electrons are scavenged is not fully inhibited; this means that the superoxide may have an alternative route for formation without direct electron transfer between BA_2_CsAg_0.95_Na_0.05_BiBr_7_ and oxygen gas. The superoxide can then react with **1a** to give **5** with a relatively high activation barrier of 27.1 kcal mol^−1^, suggesting that this is the rate‐determining step. The adiabatic ionization potential of **5** to generate the diradical **6** was calculated to be 3.81 eV. This value is too high to originate from holes in the photoexcited BA_2_CsAg_0.95_Na_0.05_BiBr_7_, but **5** can be oxidized by singlet oxygen, which has a DFT‐calculated adiabatic electron affinity of 5.06 eV. This is consistent with the results from the experiments using 9,10‐diphenylanthracene as a singlet oxygen scavenger, where the presence of 9,10‐diphenylanthracene drastically reduced the rate of **3a** formation. Intramolecular radical coupling of **6** to give the dioxetane **7** is expected to proceed quickly with an activation barrier of only 3.3 kcal mol^−1^.

The subsequent reduction of **7** by a single electron transfer from the photoexcited BA_2_CsAg_0.95_Na_0.05_BiBr_7_ is feasible based on the modest vertical electron affinity of **7** (−1.46 eV). The DFT calculations revealed that the reduction of **7** induces a barrierless ring‐opening reaction to give a benzaldehyde radical anion **8** and formaldehyde **9**. The energy barrier of the alternative, concerted retro‐cycloaddition step to give **2a** and **9** was calculated to be 30.8 kcal mol^−1^, which is likely too high for the photocatalytic reaction to operate at room temperature.

Given that the calculated adiabatic ionization potential of **8** to form **2a** is 2.47 eV, the valence band hole in BA_2_CsAg_0.95_Na_0.05_BiBr_7_ is likely sufficiently oxidizing for single electron transfer from **8** to proceed. A hydrogen atom abstraction from **2a** by singlet oxygen has an activation barrier of 19.4 kcal mol^−1^ and produces the benzoyl radical **10** and a hydroperoxyl radical. The benzoyl radical **10** is likely to combine with an oxygen molecule to afford a peroxybenzoyl radical **11** through a barrierless C‐O formation reaction, where the optimized transition state converged with a slightly negative activation energy and a geometry similar to intermediate **11**. Intermediate **11** can then add to **9** to form **12**, with a calculated C‐O bond formation barrier of 22.6 kcal mol^−1^. The rearrangement of **12** to yield formic acid

(which could be further oxidized to CO_2_) and a benzoate radical **13** is very facile and has a low barrier of only 1.6 kcal mol^−1^ (Scheme 1). Hydrogen atom abstraction from **2a** by **13** proceeds with an energy barrier of 10.4 kcal mol^−1^ to produce **3a** and **10** that can combine with oxygen gas to restart another cycle. Based on this proposed mechanism, the alkene in **1o** would transform into acetophenone instead of benzoic acid because of the additional α‐methyl substituent, whereas **1p** and **1q** would generate **4p** and **1r** would produce **4r** instead of formic acid because of the β‐methyl substituent(s) (Figure 6). These slightly adapted mechanisms can be found in Schemes S2–S4 in the Supporting Information.

The yield of **3a** when using BA_2_CsAg_0.95_Na_0.05_BiBr_7_ as the photocatalyst was 74% higher than that of the original Cs_2_AgBiBr_6_ photocatalyst under our reaction conditions. Additionally, the 5% substitution of Ag by Na at the B^I^ site led to a non‐negligible 9% improvement in the yield of **3a**. To understand the origin of these remarkable improvements in photocatalytic ability, we analyzed the differences in the electronic structures of Cs_2_AgBiBr_6_, BA_2_CsAgBiBr_7_, and BA_2_CsAg_p_Na_1‐p_BiBr_7_, where *p* = 0.875 to be computationally feasible but is not too far from the experimental value of 0.950. The projected densities of state imply that the valence band maxima and conduction band minima in both materials are mainly composed of orbital contributions by the [B^I^Br_6_]^5−^ (where B = Ag or Na) and [BiBr_6_]^3−^octahedra, with minor contributions from the A site cations (Figure S230, Supporting Information).

Since the results of both the mechanistic studies and the DFT‐verified reaction mechanism suggest the importance of superoxide in the oxidation of styrene and its derivatives, we focused our analyses particularly on the valence and conduction band edges of the perovskites since the formation of superoxide involves the transfer of photogenerated electrons from the excited photocatalyst to O_2_ The DFT‐calculated band structures of Cs_2_AgBiBr_6_ and BA_2_CsAgBiBr_7_ were found to contain indirect bandgaps, which is consistent with previously reported computational studies (Figure S231, Supporting Information).^[^ ^33^ ^]^ Remarkably, the Na‐substituted BA_2_CsAg_p_Na_1‐p_BiBr_7_ was found to have a direct band gap (Figure S231c, Supporting Information). The substitution of Ag by the small amount of Na reduces the symmetry of the double perovskite structure and introduces shallow defect states that appear at the conduction and valence band edges, which has been known to occur as a result of B‐site substitutions in the halide perovskites.^[^ ^34^ ^]^ Furthermore, the densities of electronic states within the conduction band were found to be highest in BA_2_CsAg_p_Na_1‐p_BiBr_7_ (Table S5, Supporting Information). Thus, the band structures and densities of state from our DFT calculations imply that BA_2_CsAg_0.95_Na_0.05_BiBr_7_ generates superoxide more readily because of the higher probability of photoexcited electron transfer to O_2_.

## 3 Conclusion

In summary, using an iterative workflow, we conducted experiments and built an ML model to discover correlations between the physical parameters and photocatalytic performances of lead‐free metal halide perovskites for the aerobic oxidation of styrene derivatives. With the assistance of six rounds of ML, we discovered a new BA_2_CsAg_0.95_Na_0.05_BiBr_7_ photocatalyst that was effective at catalyzing the aerobic oxidation of styrene. We demonstrated that the photocatalytic capabilities of BA_2_CsAg_0.95_Na_0.05_BiBr_7_ could be extended to a fairly general substrate scope. By combining radical scavenging experiments and DFT calculations, we proposed a mechanism involving superoxide and singlet oxygen generation on the perovskite surface. Our ML‐integrated iterative workflow with high‐throughput characterization can accelerate the discovery of photocatalyst candidates using information such as the electronegativities and space groups, coupled with a relatively small amount of reaction data, without requiring additional photophysical properties. Consequently, we propose that this iterative workflow can offer a model for a data‐driven approach to identify future catalysts from an even broader candidate space than metal halide perovskites.

## Conflict of Interest

The authors declare no conflict of interest.

## Supporting information

Supporting Information

Supporting Information

## Data Availability

The data that support the findings of this study are available from the corresponding author upon reasonable request.
