# Supplementary material for: Machine‐Learning‐Assisted Discovery of Mechanosynthesized Lead‐Free Metal Halide Perovskites for the Oxidative Photocatalytic Cleavage of Alkenes
Source: Adv Sci (Weinh). 2024 May 28;11(29):2309714. doi: 10.1002/advs.202309714 (PMC11304309; doi:10.1002/advs.202309714)
Supplement: Supplementary file 1 — Supporting Information [file ADVS-11-2309714-s002.pdf]

ADVANCED SCIENCE
Open Access

Supporting Information

for *Adv. Sci.*, DOI 10.1002/advs.202309714

Machine-Learning-Assisted Discovery of Mechanosynthesized Lead-Free Metal Halide Perovskites for the Oxidative Photocatalytic Cleavage of Alkenes

Yonghao Xiao, Khokan Choudhuri, Adisak Thanetchaiyakup, Wei Xin Chan, Xinwen Hu, Mansour Sadek, Ying Hern Tam, Ryan Guanying Loh, Sharifah Nadhirah Binte Shaik Mohammed, Kendric Jian Ying Lim, Ju Zheng Ten, Felipe Garcia, Vijila Chellappan, Tej S. Choksi, Yee-Fun Lim* and Han Sen Soo**

Supporting Information
©Wiley-VCH 2021
69451 Weinheim, Germany

Machine-Learning-Assisted Discovery of Mechanosynthesized Lead-Free Metal Halide Perovskites for the Oxidative Photocatalytic Cleavage of Alkenes

Yonghao Xiao,[a]+ Khokan Choudhuri,[a]+ Adisak Thanetchaiyakup,[a] Wei Xin Chan,[a] Xinwen Hu,[a] Mansour Sadek,[a] Ying Hern Tam,[a] Ryan Guanying Loh,[a] Sharifah Nadhirah Binte Shaik Mohammed,[a] Kendric Jian Ying Lim,[a] Ju Zheng Ten,[a] Felipe Garcia,[b,c] Vijila Chellappan,[d,e] Tej S. Choksi, [f,g],* Yee-Fun Lim,[d,h],* and Han Sen Soo[a],*

DOI: 10.1002/advs.2021XXXXX

Table of Contents

Experimental Procedures 2

Supporting Figures 5

Supporting Schemes 125

Supporting Tables 127

References 131

Author Contributions 131

Experimental Procedures

Reagents

All chemicals and solvents are of analytical grade and used without any further purification and stored in a glovebox to minimize exposure to oxygen gas. Cesium chloride (99.9%), cesium bromide (99.9%), cesium iodide (99.999%), methylammonium chloride (MACl, ⩾98%), antimony(III) chloride (⩾99%), antimony(III) iodide (98%), bismuth(III) bromide (⩾98%), bismuth(III) iodide (⩾ 99.998%), copper(II) chloride (97%), Indium(III) chloride (⩾99.998%), rubidium chloride (⩾99%), rubidium bromide (99.6%), rubidium iodide (99.9%), silver chloride (99%), silver bromide (99%), silver iodide (99%), sodium chloride (99%), potassium chloride (99%), potassium bromide (⩾99%), manganese (II) chloride (99%), hydrochloric acid (HCl, 37%), hydrobromic acid (HBr, 48%), ethyl acetate (EtOAc, ⩾99.5%), heptane (99%), toluene (99.9%), dichloromethane (DCM), and dichloroethane (DCE) were purchased from Sigma-Aldrich. Bismuth(III) chloride (＞97%), tin (II) chloride (98%), copper(I) bromide (＞98%), and hydriodic acid (HI, 57%) were purchased from Tokyo Chemical Industry. Antimony (III) bromide (99.9%) was purchased from Strem Chemicals.

General procedure for the synthesis of alkylammonium halides

Methylammonium bromide (MABr), methylammonium iodide (MAI), formamidine hydrochloride (FACl), formamidine bromide (FABr), formamidine iodide (FAI), ethylammonium bromide (EABr), propylammonium bromide (PABr), butylammonium chloride (BACl), butylammonium bromide (BABr), butylammonium iodide (BAI), and phenethylammonium bromide (PEABr): In a typical reaction, 28 mL (0.25 mol) of 48% HX (X = Cl, Br, I) solution was gradually dropped into 60 mL (0.17 mol) of methylamine, formamidine, ethylamine, propylamine, butylamine or phenylethylamine in an ice-water bath. The solution was stirred for 16 h, after which it was concentrated using a rotary evaporator at 65 ºC. Subsequently, the residue was washed at least three times with 100 mL diethyl ether. Finally, a colorless powder was collected after drying under a high vacuum for 24 h and the product was characterized by nuclear magnetic resonance (NMR) spectroscopy.

FAI: ^{1}H NMR (400 MHz, DMSO-d6) δ 1.00 (s, 2 H), 3.20 (s, 2 H) ppm. (40.78 g, 95%).

BACl: ^{1}H NMR (400 MHz, D_2O) δ 0.96 (t, *J* = 7.4 Hz, 1 H), 1.43 (dq, *J* = 14.7, 7.4 Hz, 1 H), 1.68 (p, *J* = 7.5 Hz, 2 H), 3.04 (t, *J* = 7.5 Hz, 2 H) ppm. (26.30 g, 96%).

BABr: ^{1}H NMR (400 MHz, D_2O) δ 0.97 (t, *J* = 7.3 Hz, 1 H), 1.43 (dq, *J* = 14.7, 7.4 Hz, 1 H), 1.68 (tt, *J* = 7.7, 6.5 Hz, 1 H), 3.04 (t, *J* = 7.5 Hz, 2 H) ppm. (36.58 g, 95%).

BAI: ^{1}H NMR (400 MHz, D_2O) δ 0.99 (t, *J* = 7.4 Hz, 2 H), 1.45 (dq, *J* = 14.7, 7.4 Hz, 2 H), 1.69 (q, *J* = 7.5 Hz, 2 H), 3.07 (t, *J* = 8.0 Hz, 2 H) ppm. (40.21 g, 80%).

PACl: ^{1}H NMR (400 MHz, D_2O) δ 1.01 (t, *J* = 7.5 Hz, 1 H), 1.71 (h, *J* = 7.5 Hz, 1 H), 3.00 (t, *J* = 7.5 Hz, 2 H) ppm. (22.69g. 95%).

PABr: ^{1}H NMR (400 MHz, D_2O) δ 1.03 (t, *J* = 7.5 Hz, 1 H), 1.74 (h, *J* = 7.5 Hz, 1 H), 3.03 (t, *J* = 7.4 Hz, 2 H) ppm. (33.25 g, 95%).

Machine learning workflow

The dataset for the perovskites was constructed using the curated electronegativity features A_1, A_2, B_1, etc, as described in the main text. Further to these features, the crystal space groups were added. In total, there are 23 features that make up the dataset input, indicated as follows:

['A1', 'A2', 'A10', 'B1', 'B2', 'B10', 'B3', 'B4', 'X1', 'X2', 'X10', 'Space Group_C2/c [15]', 'Space Group_C2/m', 'Space Group_Fm3m [225]', 'Space Group_P2(1)/m', 'Space Group_P21/c [14]', 'Space Group_P321 [150]', 'Space Group_P3m1 [164]', 'Space Group_P63/mmc [194]', 'Space Group_Pc [7]', 'Space Group_Pnma ', 'Space Group_Amm2 [38]', 'Space Group_Pm3m [221]'].

The dataset was used for prediction of the yield of **3a**. Before feeding into the machine learning (ML) model, a StandardScaler transformation was performed on the inputs to put them into the same scale for more consistent ML performance.

The ML algorithm was chosen to be a dense neural network (DNN), which was constructed using the Keras Python package. Owing to the small size of the dataset (85 data points), a leave-one-out cross validation method was used to evaluate the ML predictive performance. All the data points except one (i.e. 84 data points) were used for training, and the trained DNN was then used to predict

on the last datapoint (test set). This is iterated across the whole dataset, so each datapoint takes turn to become the test set. Hyperparameter tuning was performed to select the best hyperparameters for the DNN. These include the number of hidden layers, number of neurons, activation type, optimizer, learning rate, and dropout rate. Bayesian optimization (via the Scikit-Optimize Python package) was used for the hyperparameter tuning.

To extract information from the ML training, feature importances were extracted via the Python SHAP package. The SHAP KernelExplainer was used to extract the SHAP values, which were then plotted with the SHAP summary plot to illustrate the effects of each of the input features on the output (yield of **3a**).

Characterization

X-ray diffraction (XRD) was performed on a XRD Bruker D8 Discover diffractometer with a copper target X-ray tube set to 40 kV and 40 mA. The batch sample automatic loader could be set by the software and allowed us to continuously test 12 samples without the need for manual sample changing. The Bragg-Brentano geometry was used in the XRD investigations on the powder materials, which were conducted in the θ-2θ scan mode with a scan rate of 4° per min. An automated Tecan Infinite 200 Pro equipment with a ZINSSER-LISSY system was used to perform photoluminescence spectroscopy. The 24 powder samples are loaded into a 24-well polystyrene plate with a well diameter of 16 mm. During the photoluminescence experiments, an excitation wavelength of 370 nm, and a step size of 2 nm were used to measure the samples between 400 and 800 nm. The reflectance spectra of the samples were obtained by a Resonon Pika L hyperspectral imaging camera, examined through the Spectronon Pro software, and then transformed and normalized using the Kubelka-Munk **Equation S1** into absorption spectra.

$$F = \frac{(1-R)^2}{2R} \qquad (1)$$

where, F and R are the absorption and remission fraction of sample respectively.

Preparation of perovskites

Briefly, the precursor chemicals that match the stoichiometric ratio were weighed in a glovebox filled with N_2 at room temperature (RT). The substances were then loaded into a 10 mL ZrO_2 ball-milling jar (Ants Scientific) with one 10 mm 4.0 g ZrO_2 ball. It was sealed and taken out of the glovebox, installed into a Retsch MM400 shaker mill, and oscillated at a frequency of 30 Hz with various time durations. After unsealing the milling jar, the final products were collected in a fume hood and transferred back to the glovebox immediately.

General Procedure for the Synthesis of Perovskites

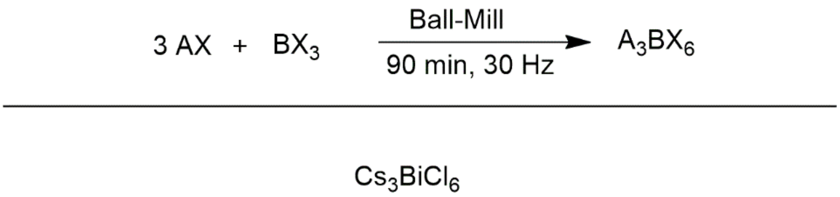

For a typical synthesis, AX (3 mmol), BX_3 (1 mmol), and one grinding ball (10 mm diameter, ZrO_2) were placed in an oven-dried 10 mL ZrO_2 ball-milling jar (Ants Scientific) in a glovebox. The milling was conducted at a frequency of 30 Hz for 90 min using a Retsch MM 400 mixer mill, after which the product was transferred and stored in a glovebox under a N_2 atmosphere.

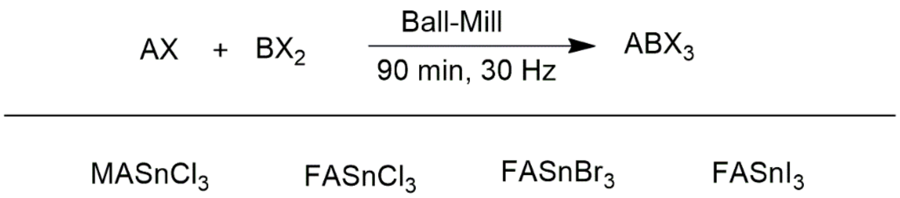

In a typical synthesis, AX (1 mmol), BX_3 (1 mmol), and one grinding ball were placed in a 10 mL ball-milling ZrO_2 jar. After continuous milling at a frequency of 30 Hz for 90 min, the product was transferred and stored in a glovebox under a N_2 atmosphere.

In a typical synthesis, AX (3 mmol), BX_3 (2 mmol), and one grinding ball were placed in a 10 mL ball-milling ZrO_2 jar. After continuous milling at a frequency of 30 Hz for 90 min, the product was transferred and stored in a glovebox under a N_2 atmosphere.

General Procedure for the Synthesis of Double Perovskites

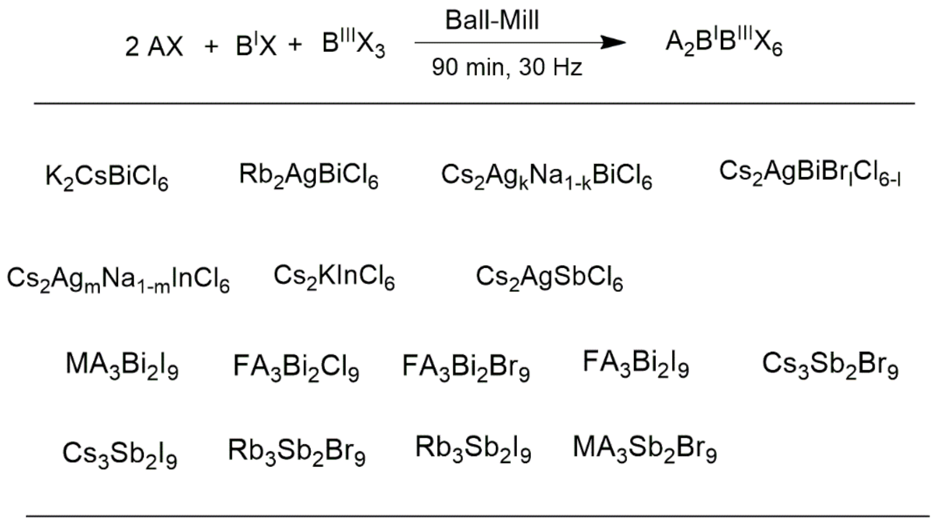

In a typical procedure, AX (2 mmol), BX (1 mmol), CX_3 (1 mmol), and one grinding ball (10 mm diameter, ZrO_2) were placed in a 10 mL ball-milling ZrO_2 jar. We performed the milling at a frequency of 30 Hz for 60 min. After that, the product was transferred and stored in a glove box under a N_2 atmosphere.

General Procedure for the Perovskite-Photocatalyzed Styrene Oxidation Reaction

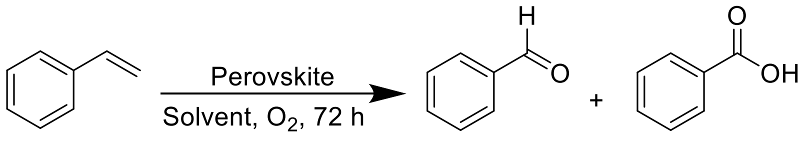

1a **2a** **3a**

In a Schlenk tube, styrene (**1a**, 60 µl, 0.523 mmol) was dissolved in DCE (3 mL) and a catalytic amount of the perovskite (5 mol%) was added. Then, the reaction mixture was stirred under an O_2 atmosphere (1 bar, O_2 balloon) at RT using blue light-emitting diode (LED) irradiation. After 72 h an internal standard (1,1,2,2-tetrachloroethane) was added (1 equiv. with respect to **1a**) to the reaction mixture. Subsequently, an aliquot of the sample was taken and analyzed by NMR spectroscopy to determine the percentage yield of the products in the reaction mixture. The product was purified through silica gel column chromatography using a mixture of EtOAc (80%) and hexane (20%) as the eluent.

The perovskites used for stability testing were recovered after a 72 h photocatalytic oxidation of styrene under the standard optimized conditions by centrifuging the crude reaction mixture and decanting the supernatant. After that, the precipitate was suspended in 30 mL of DCM and centrifuged again. Each centrifugation was conducted at 10,000 rpm for 10 min. After decanting the supernatant, the residues were dried under vacuum overnight and characterized by XRD measurements. $BA_2CsAg_{0.95}Na_{0.05}BiBr_7$ and $PEA_2CsAg_{0.95}Na_{0.05}BiBr_7$ were then used again for the photocatalytic oxidation of styrene in place of the fresh perovskite under the same optimized reaction conditions, and the products were likewise analyzed similarly by ^1H NMR spectroscopy.

For the perovskite structure, the most commonly used and most successful predictive geometric ratios are the Goldschmidt tolerance factor (TF, **Equation S2**) and octahedral factor (µ, **Equation S3**),

$$\mathrm{TF} = \frac{rA + rX}{\sqrt{2}\{[rM(\mathrm{I}) + rM(\mathrm{III})]/2 + rX\}} \quad (0.8\sim1.0) \qquad (2)$$

$$\mu = \frac{rM(\mathrm{III})}{rX} \quad (\geq 0.41) \qquad (3)$$

where, rA, rM(I), and rM(III) are the ionic radius of the A, M(I), and M(III) site cations respectively, and rX is the ionic radius of the anion.[1]

Computational Details

Density functional theory (DFT) was used for electronic structure analyses and for calculations involving the adsorption of O_2 on the perovskite surfaces and were performed using the QUANTUM ESPRESSO code.[2] The core-valence interaction was described by ultrasoft pseudopotentials[3] and the D3 correction method was used to account for the van der Waals interactions.[4] For geometry optimizations, a kinetic energy cutoff of 500 eV was used and the convergence criteria was chosen such that the maximum forces acting on each relaxed atom were less than 0.05 eV/Å and the energy change was less than 10^{-5} eV. The optimized bulk geometries of $Cs_2AgBiBr_6$ and $BA_2CsAgBiBr_7$ were calculated using Monkhorst-Pack *k*-point meshes of 4 × 4 × 4 and 6 × 6 × 2 respectively. The (002) facet of $BA_2CsAgBiBr_7$ was selected as the typical catalytic surface and was modelled using a slabs of approximately 20 Å thickness, while a 20 Å vacuum layer along the c axis was used to minimize the interactions between the layers.

For geometry optimization of the $BA_2Cs_2AgBiBr_7$ slab, a 6 × 6 × 1 *k*-point mesh was used and the uppermost BA chains and the inorganic perovskite layers were allowed to relax, while the rest of the atoms were constrained. To model the 5% Na doping in $BA_2CsAg_pNa_{1-p}Br_7$ within a reasonable time, a 2 × 2 slab supercell was created with one Ag atom replaced with Na, giving a Na concentration of 12.5%, and a 3 × 3 × 1 *k*-point mesh was used for self-consistent calculations. The non-self consistent calculations of the electronic structures were performed on the models of $Cs_2AgBiBr_6$, $BA_2CsAgBiBr_7$, and $BA_2CsAg_pNa_{1-p}Br_7$ using 12 × 12 × 12, 18 × 18 × 3, and 5 × 5 × 2 *k*-point meshes respectively. Band structure diagrams were calculated along high symmetry points in the Brillouin zone using the paths outlined by Tanaka and co-workers.[5]

For calculations involving the isolated gas phase O_2, a 1 x 1 x 1 Monkhorst-Pack *k*-point mesh was used and the O_2 molecules were separated by at least 40 Å of vacuum. Due to the heavy computational demands of modelling the 5% doping of Na, the undoped $BA_2CsAgBiBr_7$ was used for the O_2 adsorption studies. Spin-polarization was included for optimization involving the O_2 adsorption. After optimization, the spin polarization maps were visualized using the VESTA software.[6]

For DFT calculations modelling the oxidation of **1a**, the optimized equilibrium geometries and calculated vibrational frequencies were obtained with the Gaussian 16c program[7] at the B3LYP/6-311G++ (d,p) level,[8] with the inclusion of a conductor-like polarizable continuum model with the parameters for DCE as implemented in Gaussian 16c to mimic the interaction with the surrounding solvent. The calculated vibrational frequencies were used to ensure that the optimized structures were true minima on the potential energy surface. The transition states structures of the proposed mechanism for **1a** oxidation to **3a** were optimized at the same B3LYP/6-311G++(d,p) level, and frequency calculations were performed on the optimized structures to ensure that there is only one imaginary frequency.

Supporting Figures

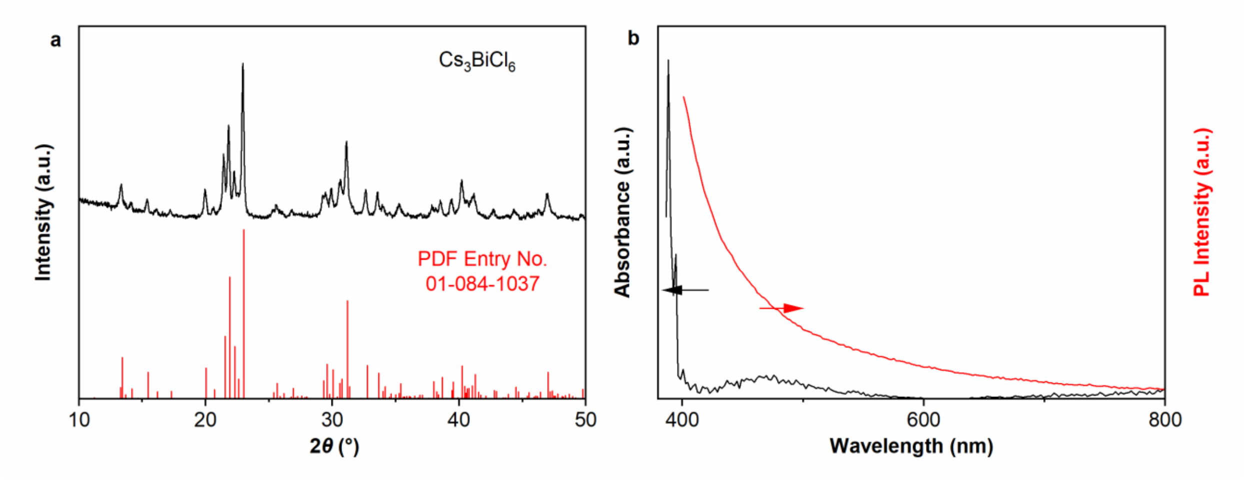

Figure S1. (a) XRD data (top) and reference pattern (bottom) of Cs_3BiCl_6. (b) Optical absorption (black) and PL (red) spectra of Cs_3BiCl_6.

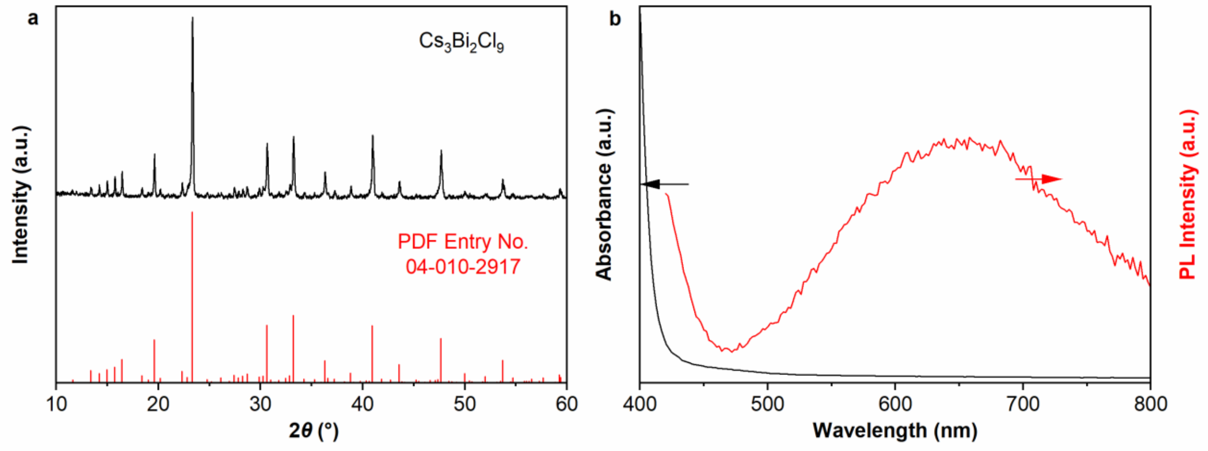

Figure S2. (a) XRD data (top) and reference pattern (bottom) of $Cs_3Bi_2Cl_9$. (b) Optical absorption (black) and PL (red) spectra of $Cs_3Bi_2Cl_9$.

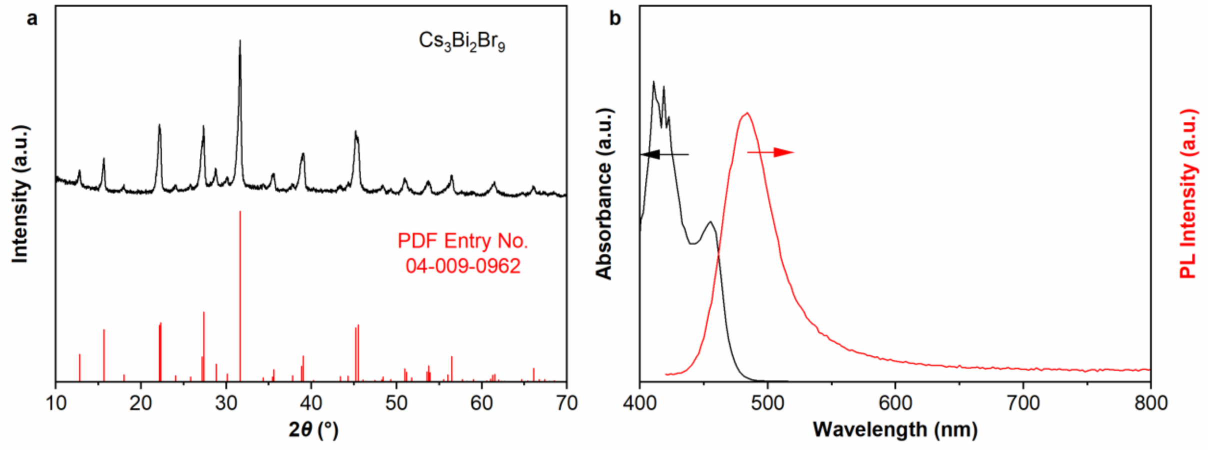

Figure S3. (a) XRD data (top) and reference pattern of $Cs_3Bi_2Br_9$. (b) Optical absorption (black) and PL (red) spectra of $Cs_3Bi_2Br_9$.

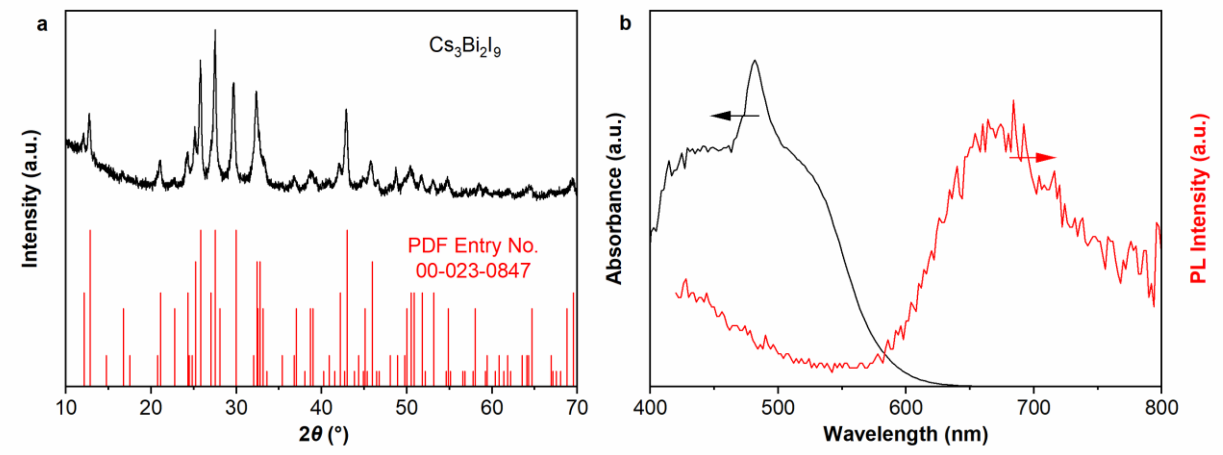

Figure S4. (a) XRD data (top) and reference pattern (bottom) of $Cs_3Bi_2I_9$. (b) Optical absorption (black) and PL (red) spectra of $Cs_3Bi_2I_9$.

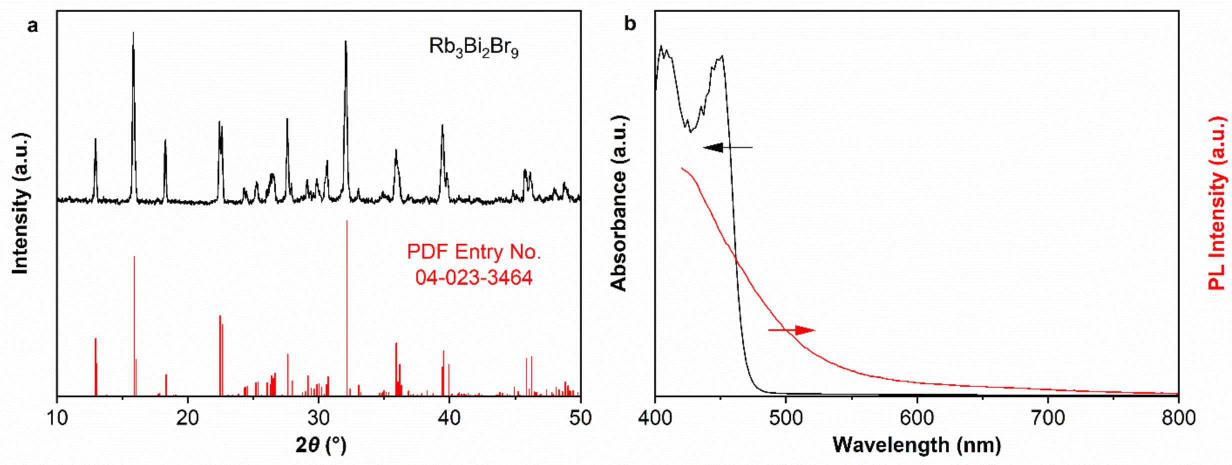

Figure S5. (a) XRD data (top) and reference pattern (bottom) of $Rb_3Bi_2Br_9$. (b) Optical absorption (black) and PL (red) spectra of $Rb_3Bi_2Br_9$.

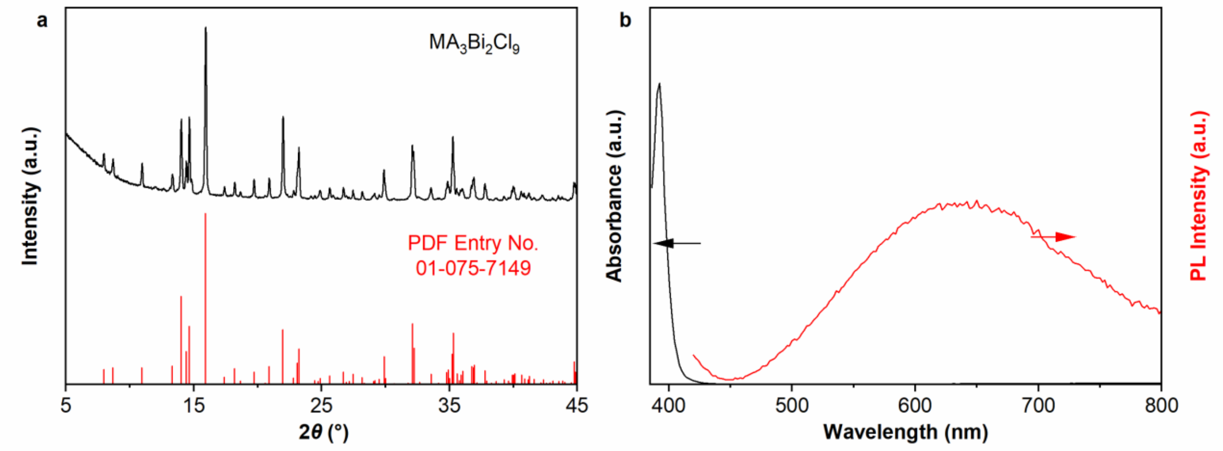

Figure S6. (a) XRD data (top) and reference pattern (bottom) of $MA_3Bi_2Cl_9$. (b) Optical absorption (black) and PL (red) spectra of $MA_3Bi_2Cl_9$.

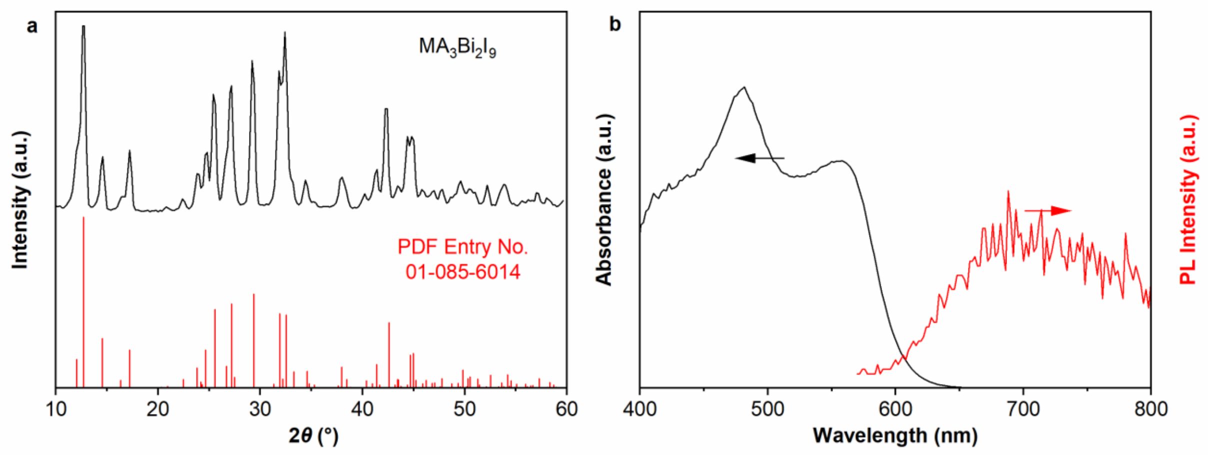

Figure S7. (a) XRD data (top) and reference pattern (bottom) of $MA_3Bi_2I_9$. (b) Optical absorption (black) and PL (red) spectra of $MA_3Bi_2I_9$.

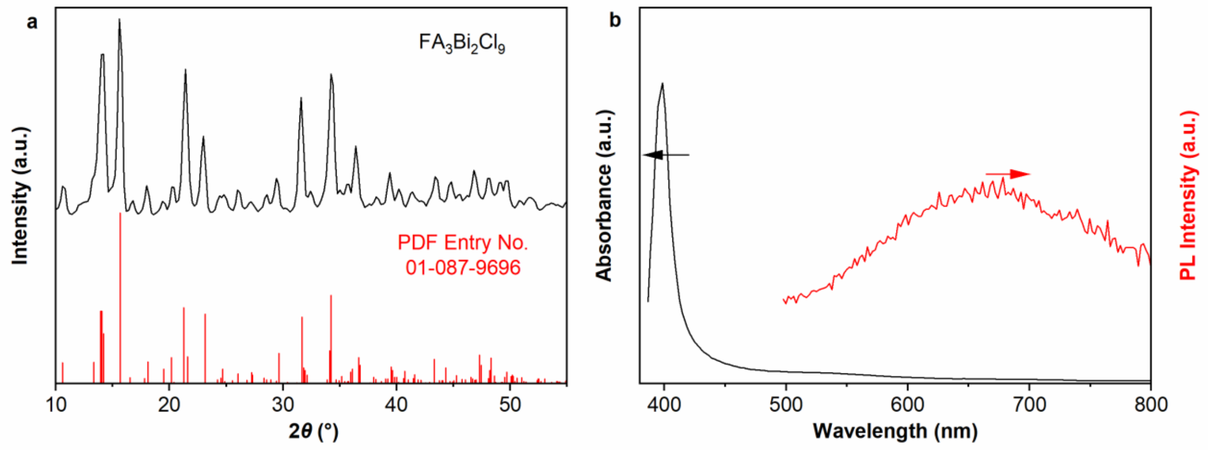

Figure S8. (a) XRD data (top) and reference pattern (bottom) of $FA_3Bi_2Cl_9$. (b) Optical absorption (black) and PL (red) spectra of $FA_3Bi_2Cl_9$.

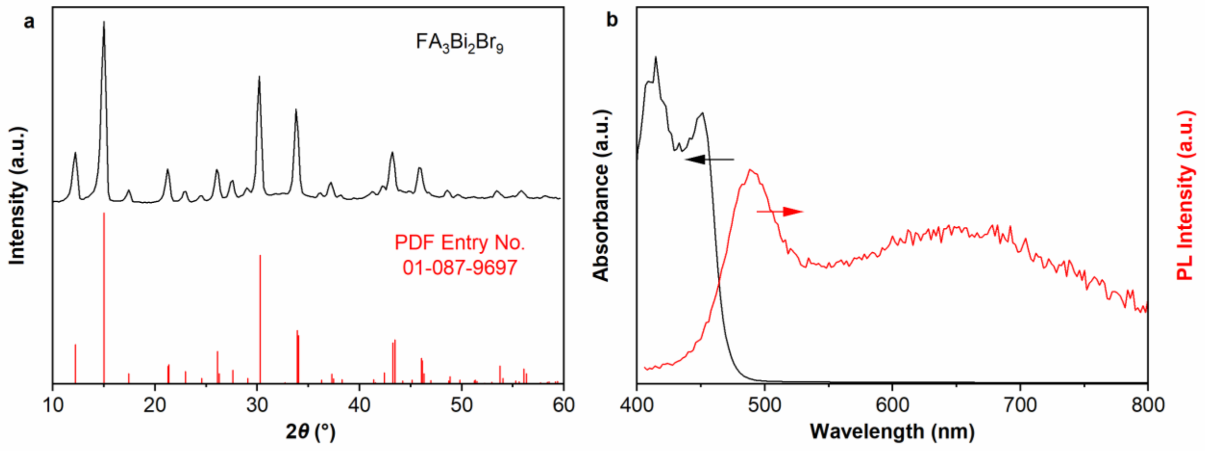

Figure S9. (a) XRD data (top) and reference pattern (bottom) of $FA_3Bi_2Br_9$. (b) Optical absorption (black) and PL (red) spectra of $FA_3Bi_2Br_9$.

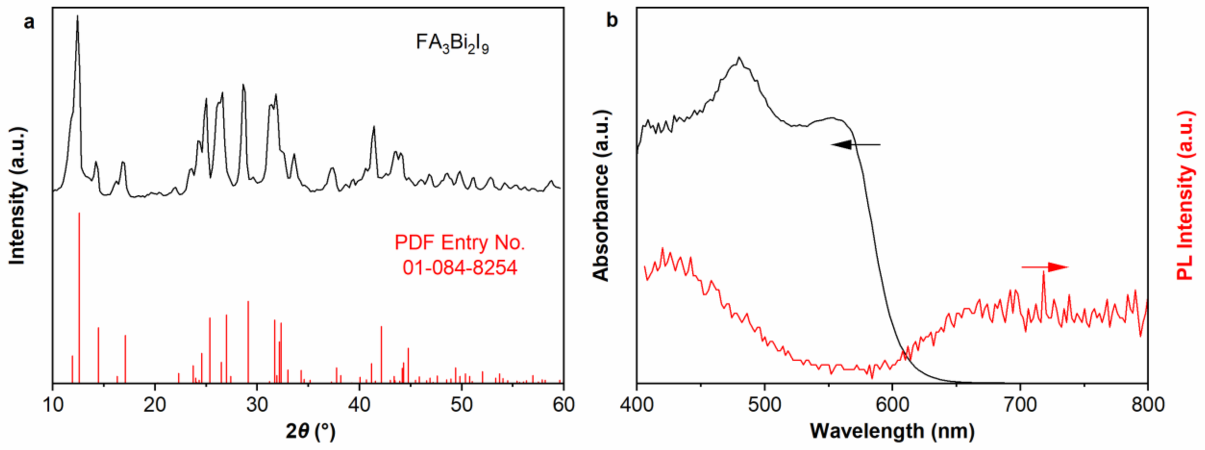

Figure S10. (a) XRD data (top) and reference pattern (bottom) of $FA_3Bi_2I_9$. (b) Optical absorption (black) and PL (red) spectra of $FA_3Bi_2I_9$.

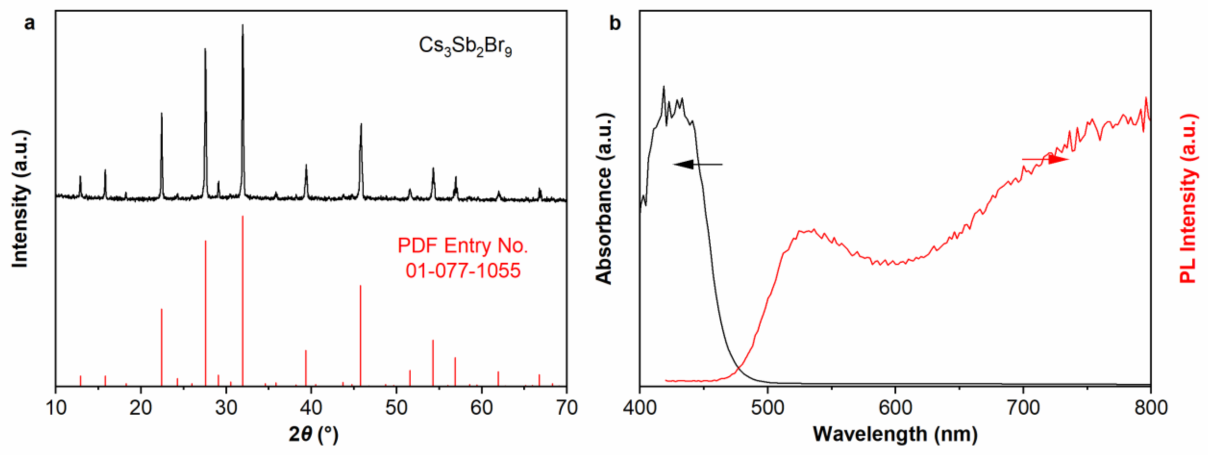

Figure S11. (a) XRD data (top) and reference pattern (bottom) of $Cs_3Sb_2Br_9$. (b) Optical absorption (black) and PL (red) spectra of $Cs_3Sb_2Br_9$.

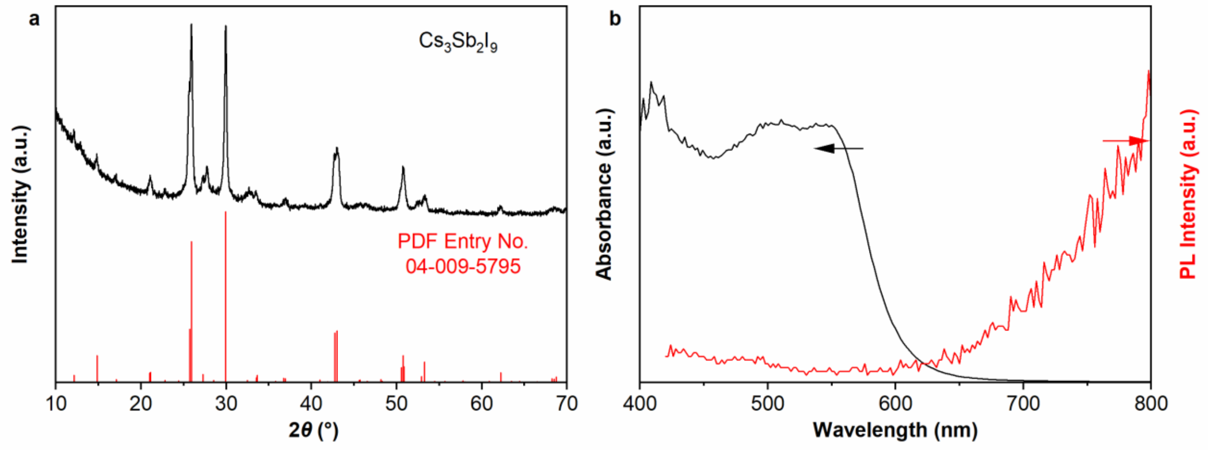

Figure S12. (a) XRD data (top) and reference pattern (bottom) of $Cs_3Sb_2I_9$. (b) Optical absorption (black) and PL (red) spectra of $Cs_3Sb_2I_9$.

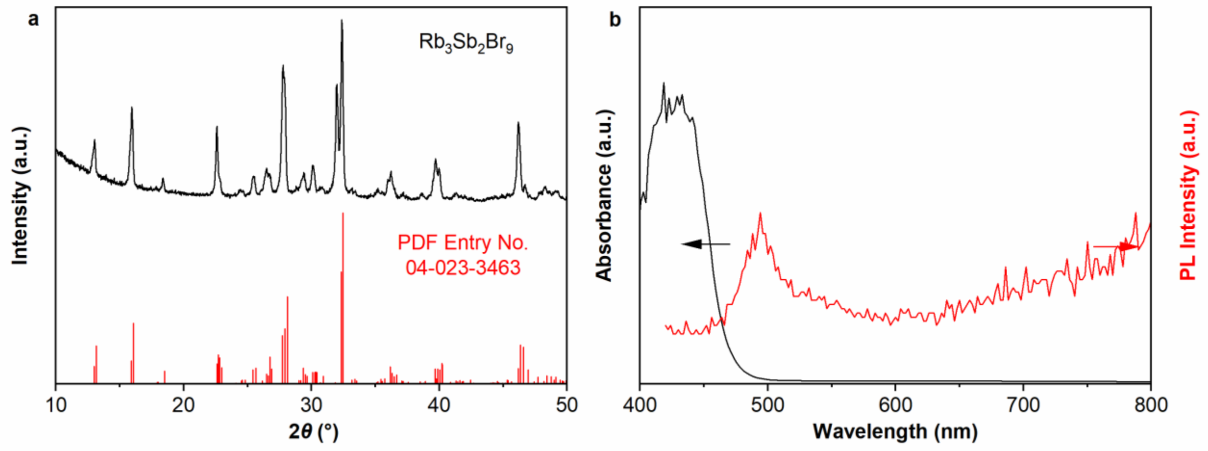

Figure S13. (a) XRD data (top) and reference pattern (bottom) of $Rb_3Sb_2Br_9$. (b) Optical absorption (black) and PL (red) spectra of $Rb_3Sb_2Br_9$.

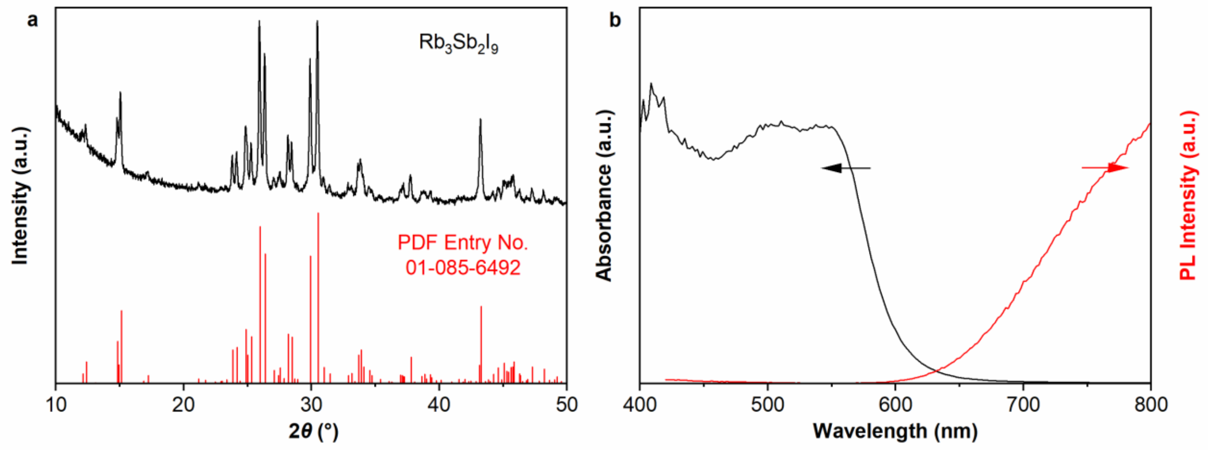

Figure S14. (a) XRD data (top) and reference pattern (bottom) of $Rb_3Sb_2I_9$. (b) Optical absorption (black) and PL (red) spectra of $Rb_3Sb_2I_9$.

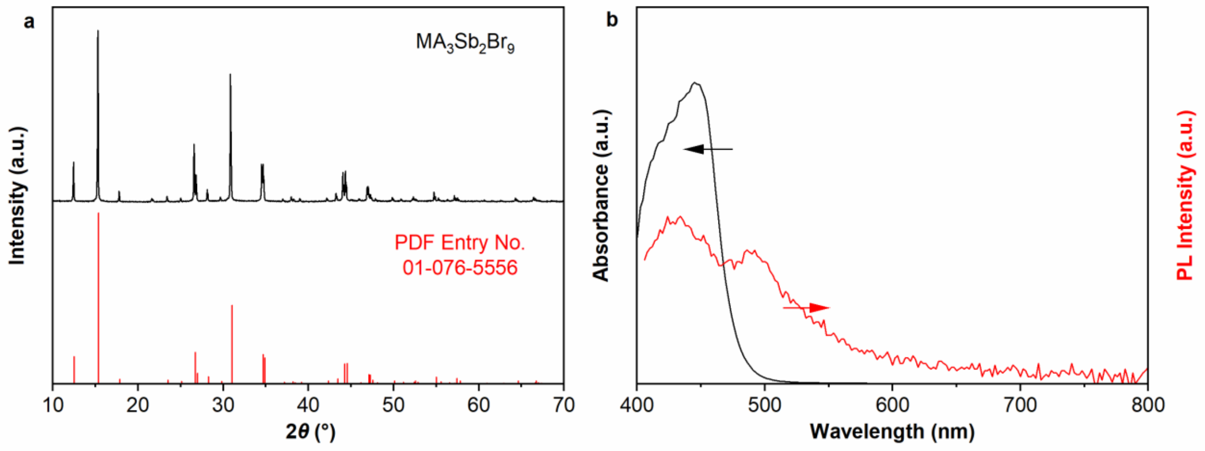

Figure S15. (a) XRD data (top) and reference pattern (bottom) of $MA_3Sb_2Br_9$. (b) Optical absorption (black) and PL (red) spectra of $MA_3Sb_2Br_9$.

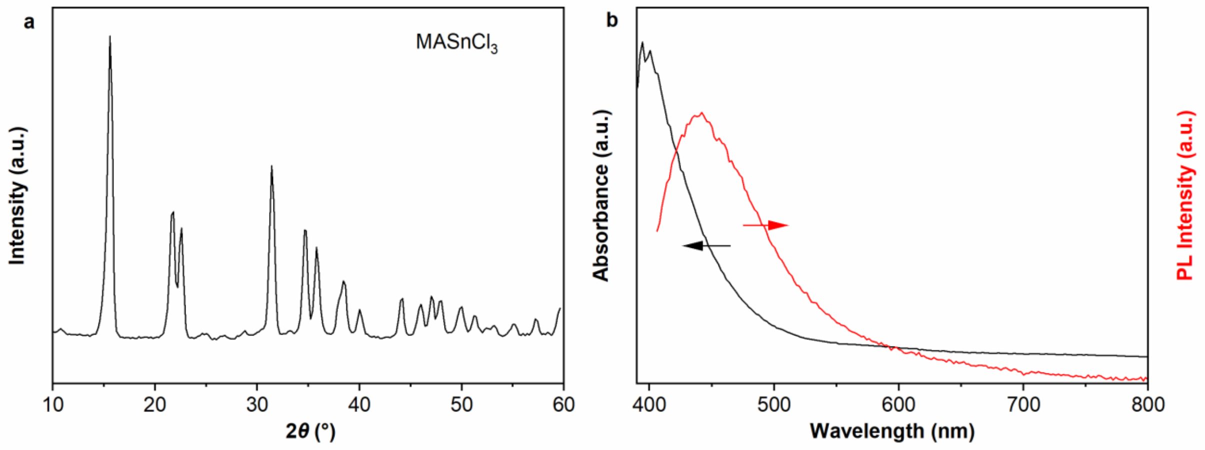

Figure S16. (a) XRD data of $MASnCl_3$. (b) Optical absorption (black) and PL (red) spectra of $MASnCl_3$.

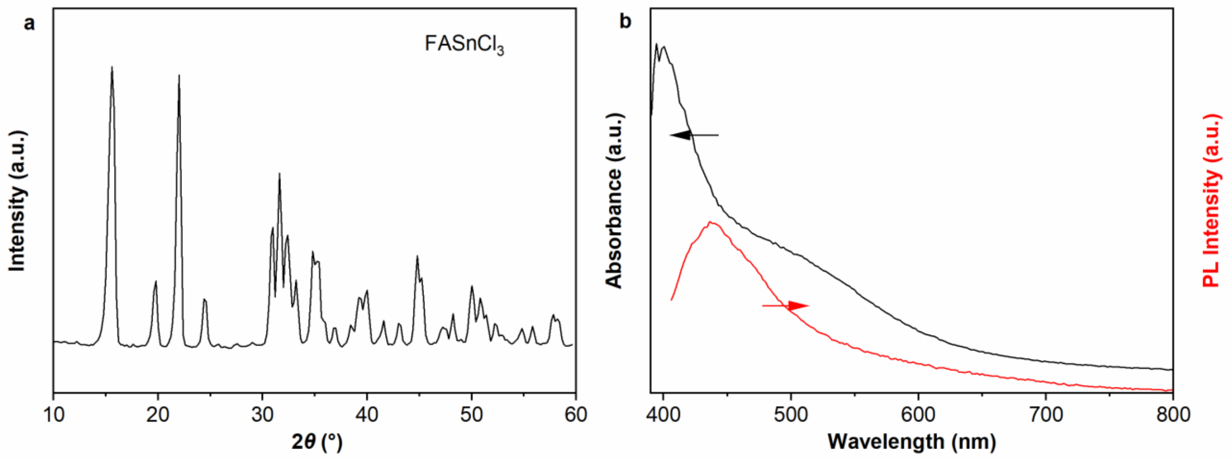

Figure S17. (a) XRD data of $FASnCl_3$. (b) Optical absorption (black) and PL (red) spectra of $FASnCl_3$.

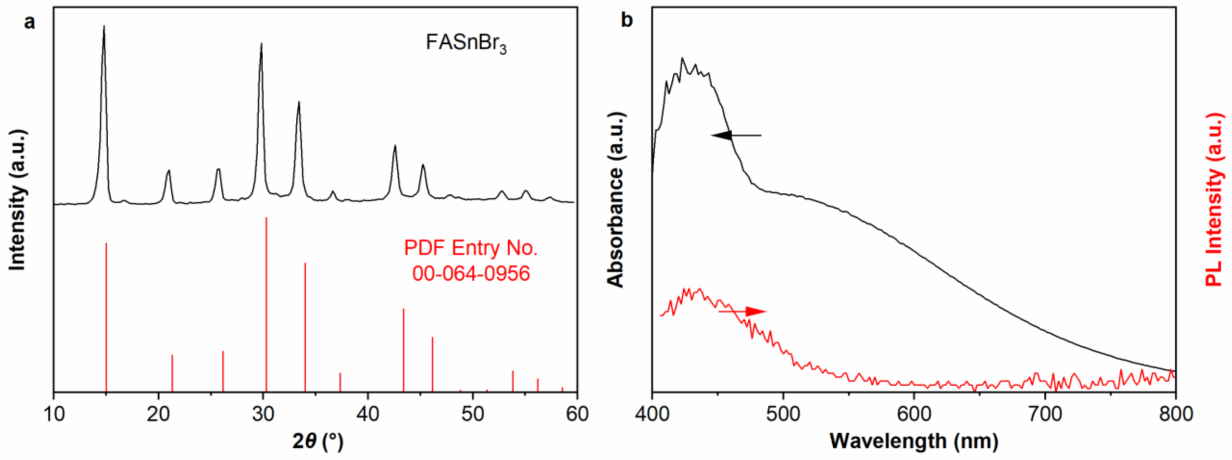

Figure S18. (a) XRD data (top) and reference pattern (bottom) of $FASnBr_3$. (b) Optical absorption (black) and PL (red) spectra of $FASnBr_3$.

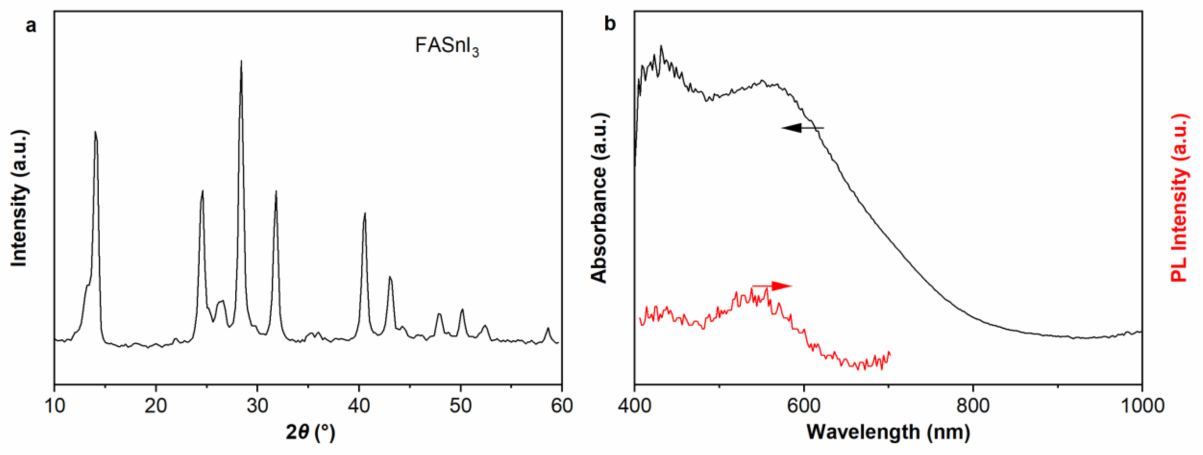

Figure S19. (a) XRD data of $FASnI_3$. (b) Optical absorption (black) and PL (red) spectra of $FASnI_3$.

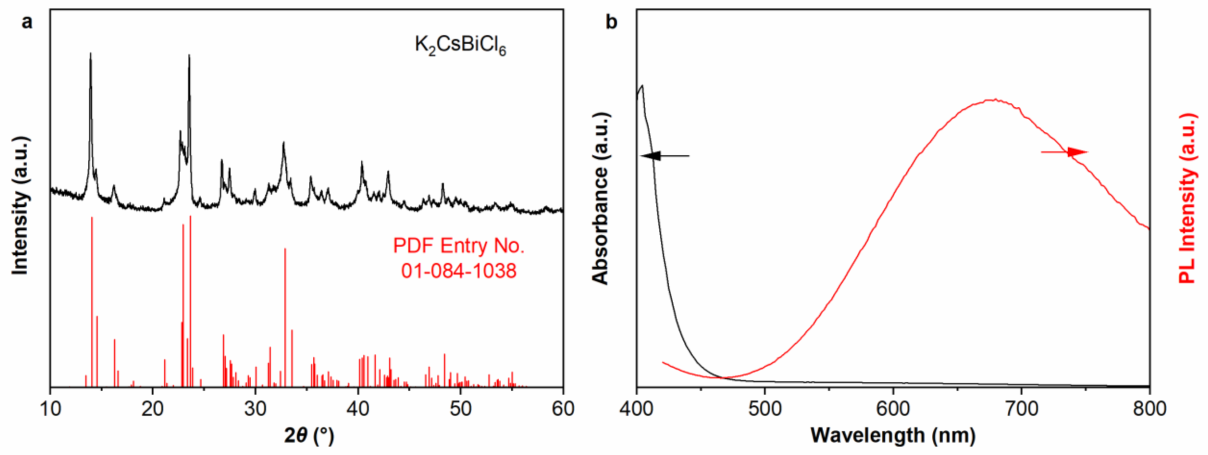

Figure S20. (a) XRD data (top) and reference pattern (bottom) of $K_2CsBiCl_6$. (b) Optical absorption (black) and PL (red) spectra of $K_2CsBiCl_6$.

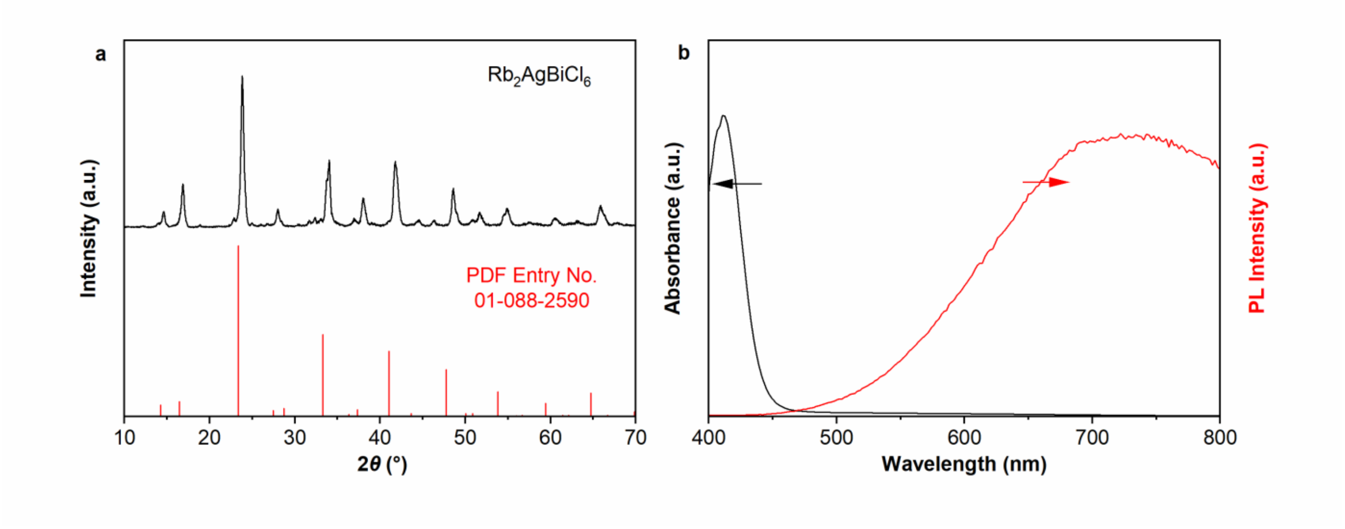

Figure S21. (a) XRD data (top) of $Rb_2AgBiCl_6$ and reference pattern of $Cs_2AgBiCl_6$. (b) Optical absorption (black) and PL (red) spectra of $Rb_2AgBiCl_6$.

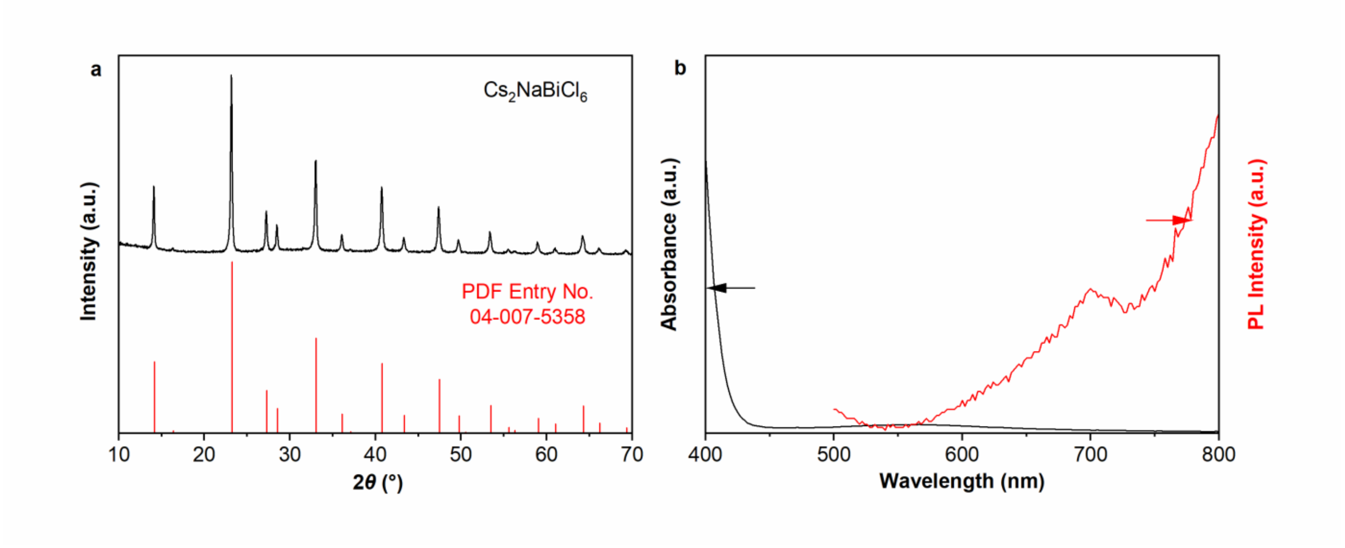

Figure S22. (a) XRD data (top) and reference pattern (bottom) of $Cs_2NaBiCl_6$. (b) Optical absorption (black) and PL (red) spectra of $Cs_2NaBiCl_6$.

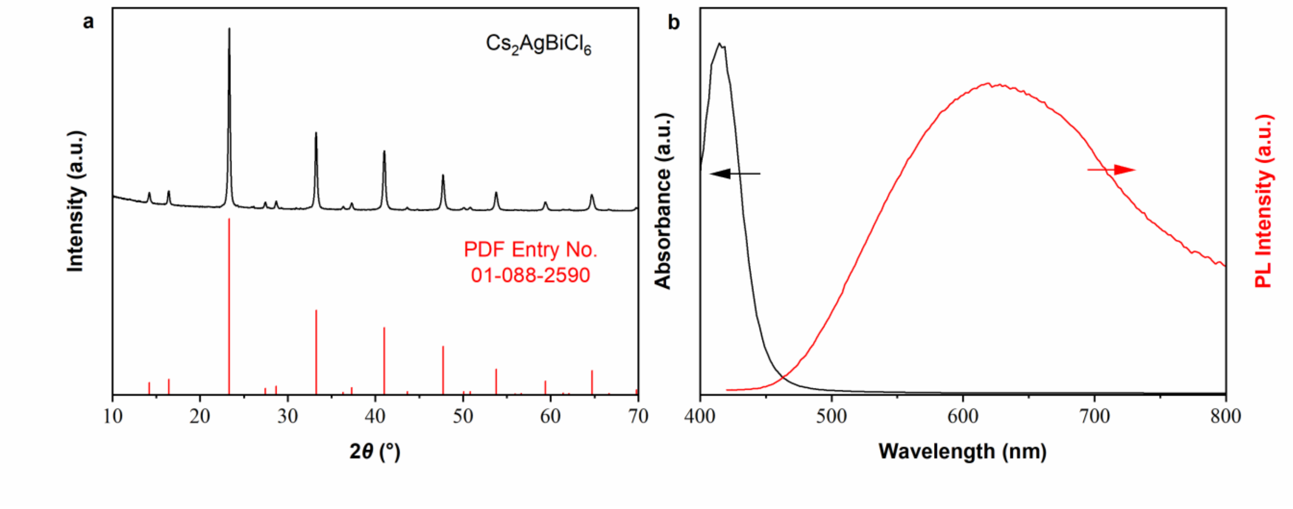

Figure S23. (a) XRD data (top) and reference pattern (bottom) of $Cs_2AgBiCl_6$. (b) Optical absorption (black) and PL (red) spectra of $Cs_2AgBiCl_6$.

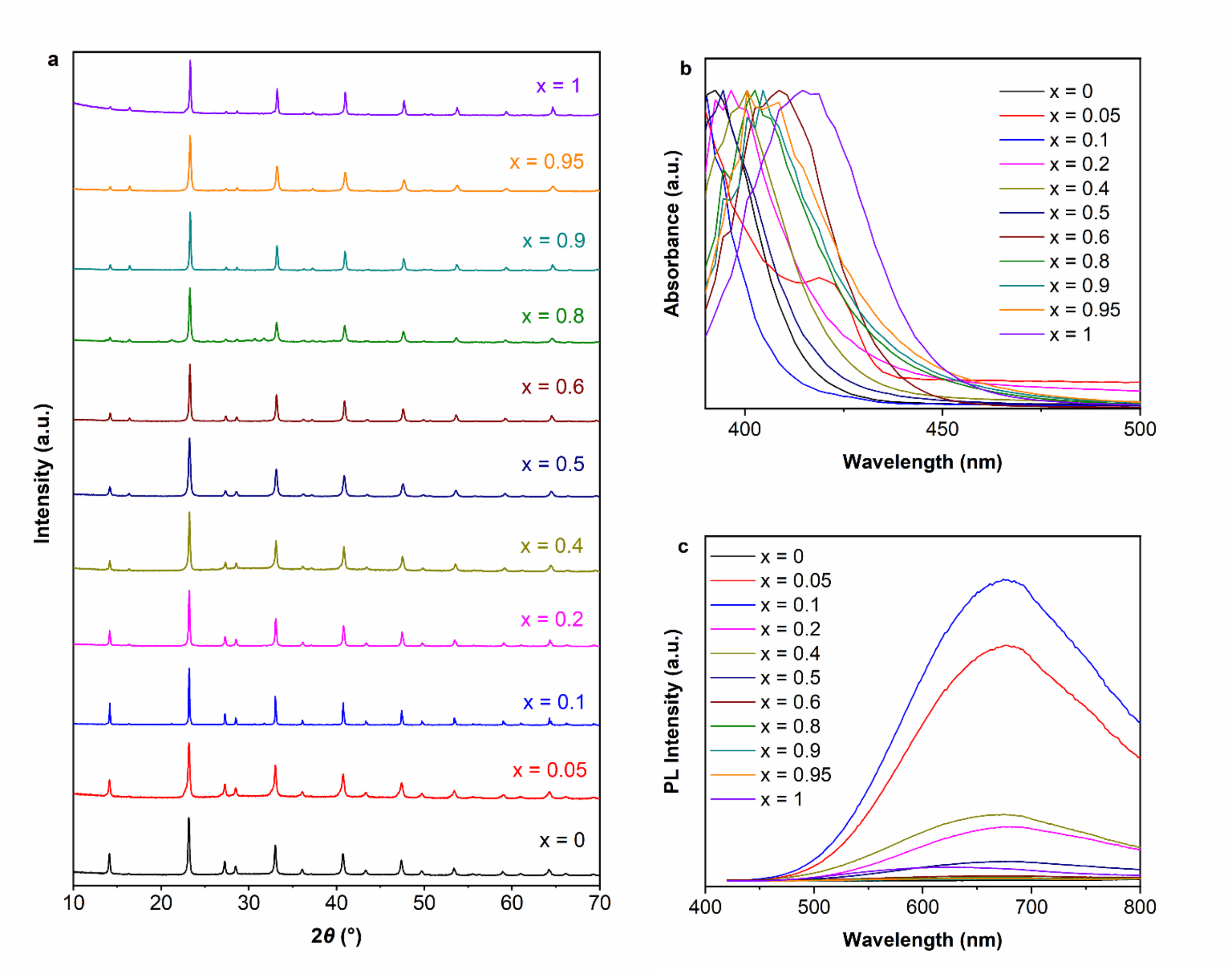

Figure S24. (a) XRD data, (b) optical absorption spectra, and (c) PL spectra of $Cs_2Ag_kNa_{1-k}BiCl_6$ (k = 0, 0.05, 0.1, 0.2,0.4, 0.5, 0.6, 0.8, 0.9, 0.95, 1.).

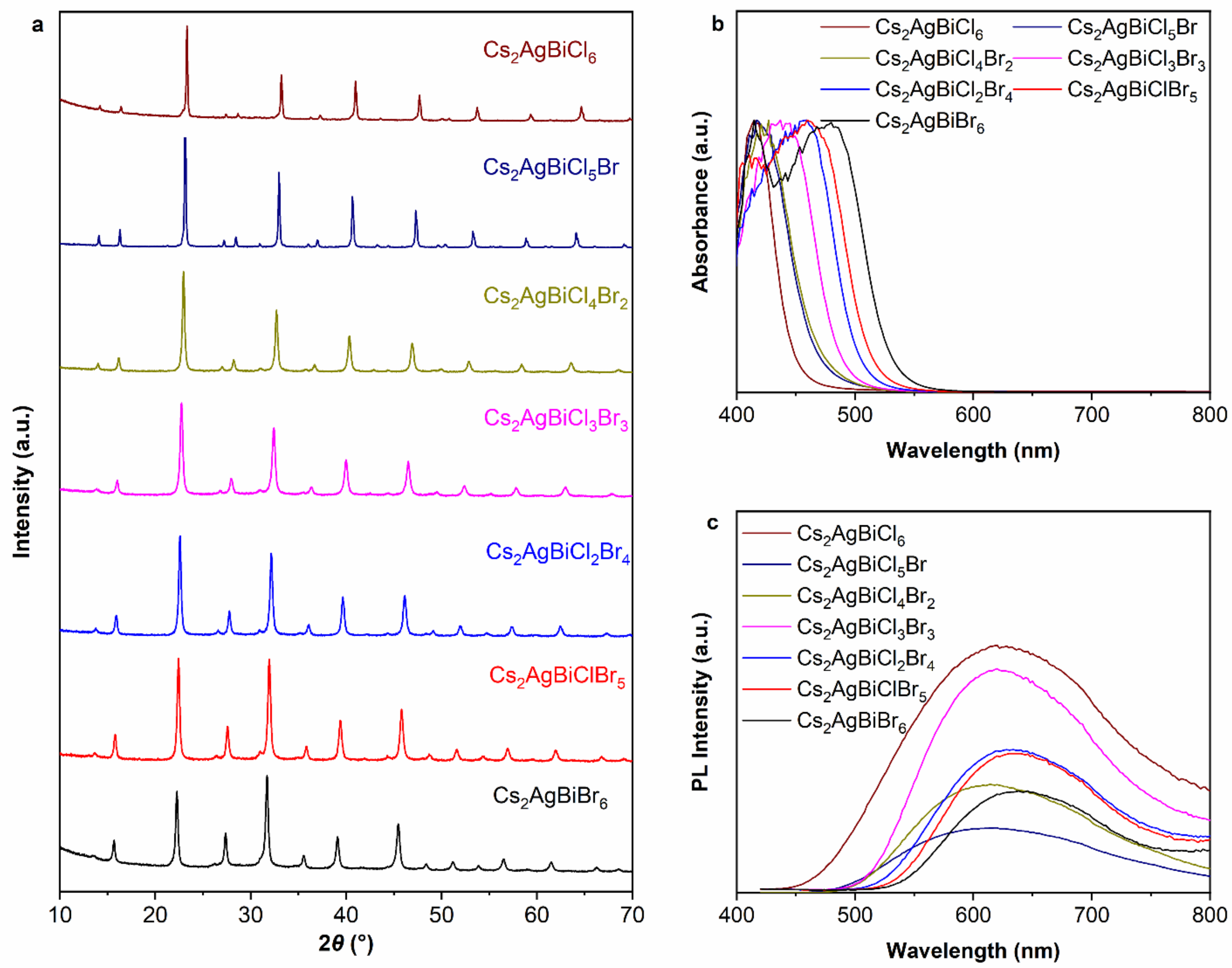

Figure S25. (a) XRD data, (b) optical absorption spectra, and (c) PL spectra of $Cs_2AgBiCl_iBr_{6-i}$ (i = 0.2, 0.4, 0.5, 0.6, 0.8, 1).

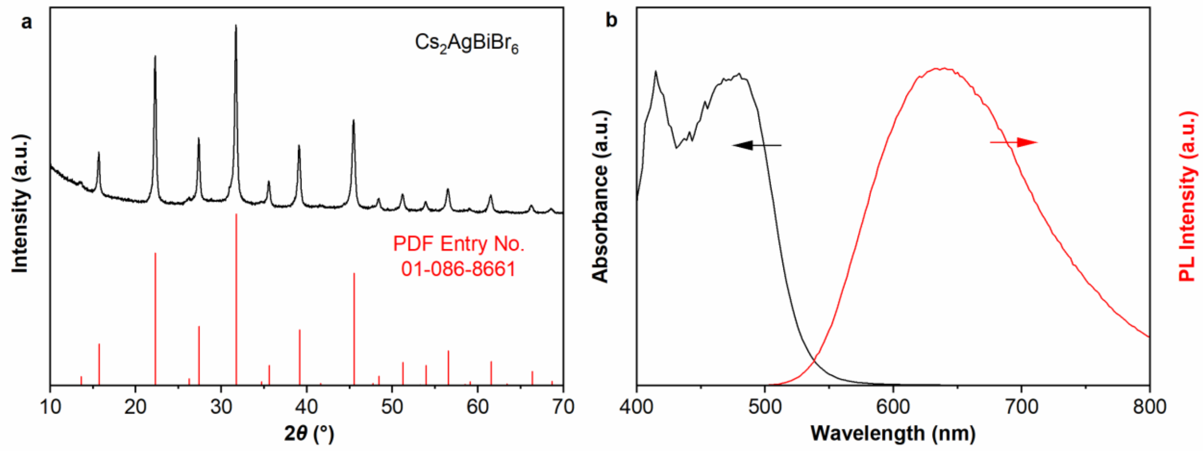

Figure S26. (a) XRD data (top) and reference pattern (bottom) of $Cs_2AgBiBr_6$. (b) Optical absorption (black) and PL (red) spectra of $Cs_2AgBiBr_6$.

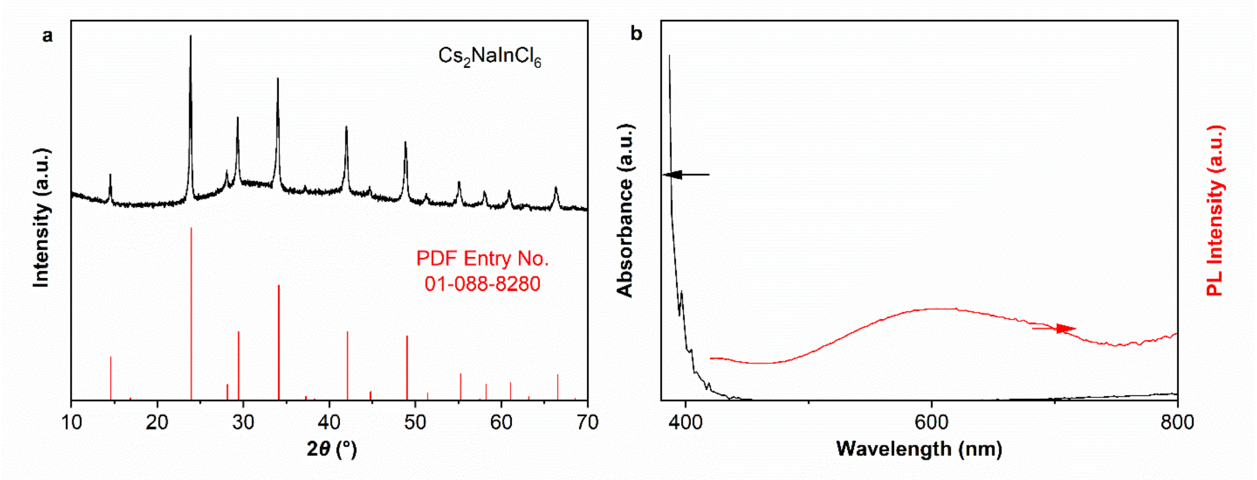

Figure S27. (a) XRD data (top) and reference pattern (bottom) of $Cs_2NaInCl_6$. (b) Optical absorption (black) and PL (red) spectra of $Cs_2NaInCl_6$.

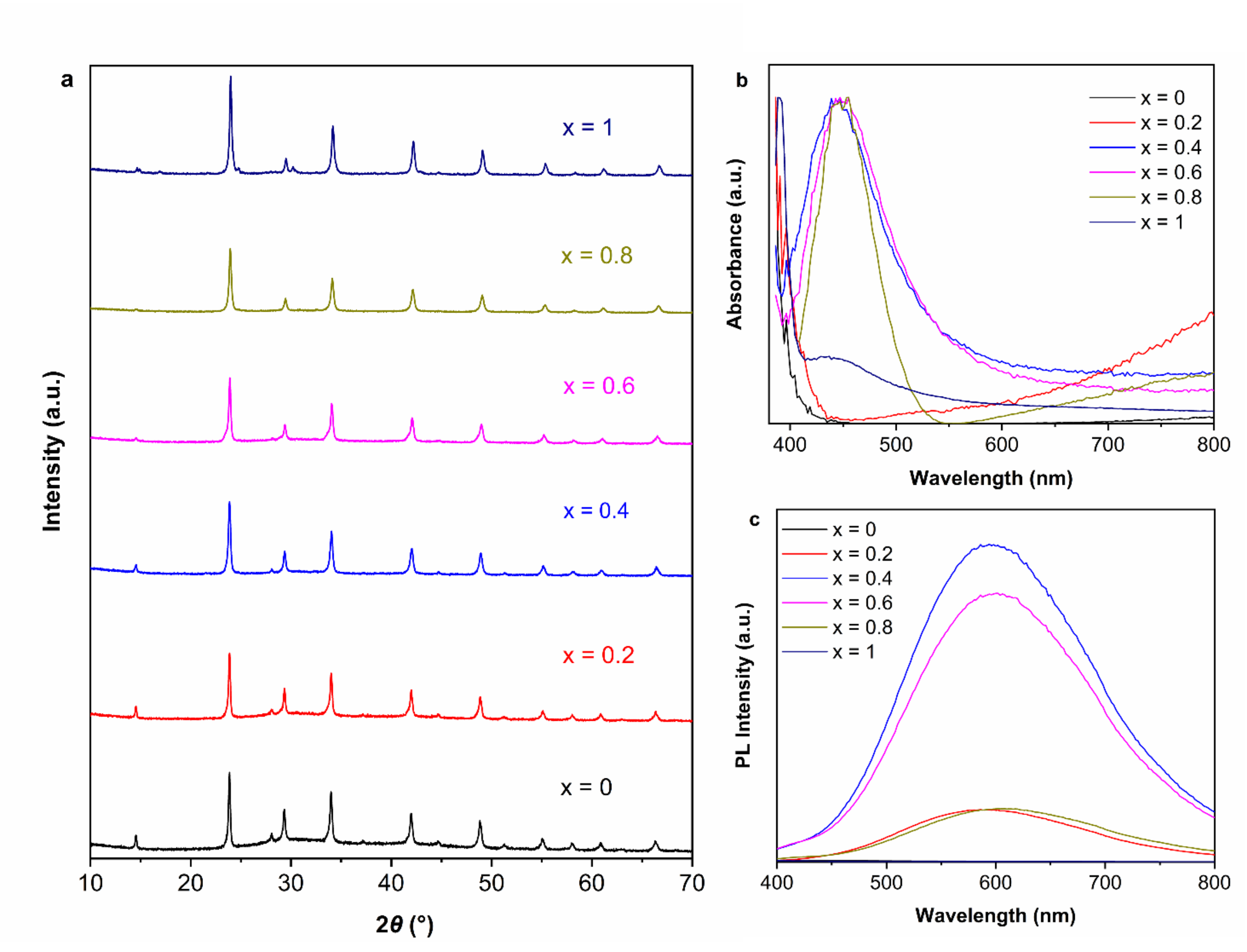

Figure S28. (a) XRD data, (b) optical absorption spectra, and (c) PL spectra of $Cs_2Ag_mNa_{1-m}InCl_6$ (m = 0, 0.2, 0.4, 0.6, 0.8, 1).

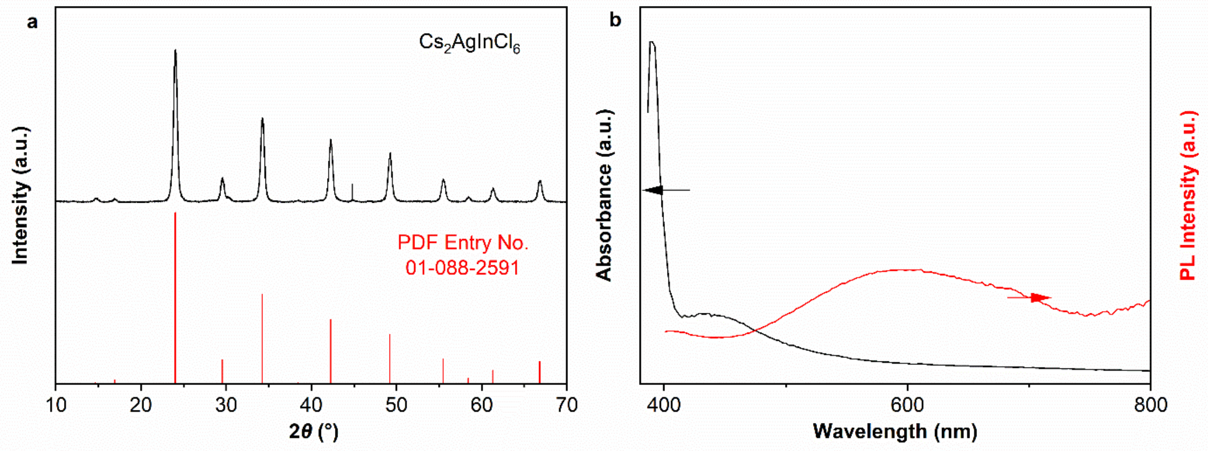

Figure S29. (a) XRD data (top) and reference pattern (bottom) of $Cs_2AgInCl_6$. (b) Optical absorption (black) and PL (red) spectra of $Cs_2AgInCl_6$.

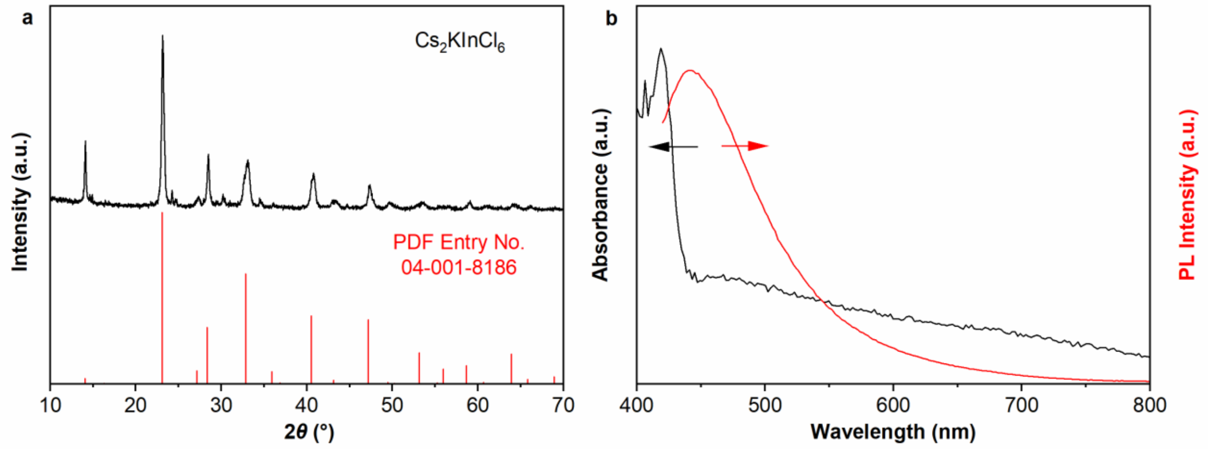

Figure S30. (a) XRD data (top) and reference pattern (bottom) of Cs_2KInCl_6. (b) Optical absorption (black) and PL (red) spectra of Cs_2KInCl_6.

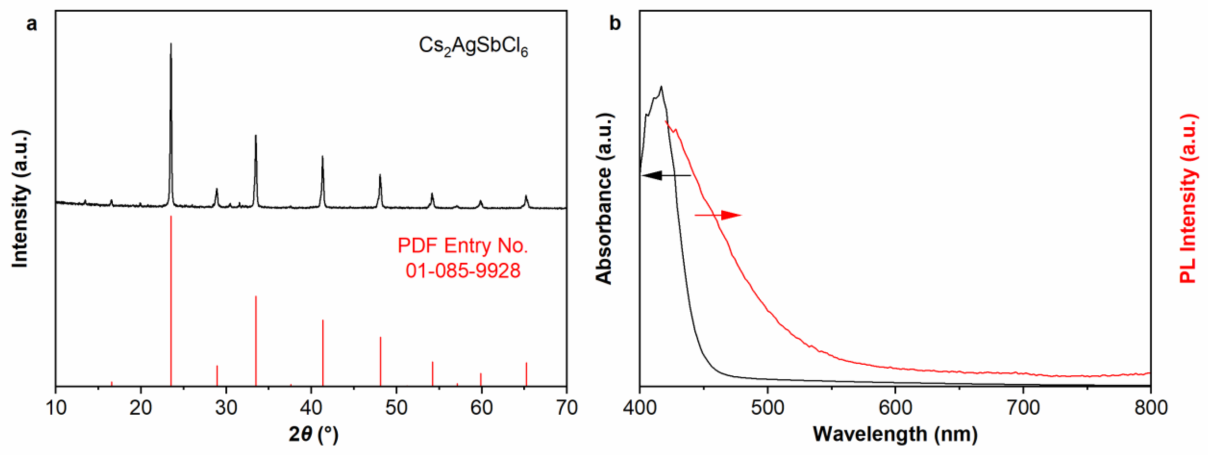

Figure S31. (a) XRD data (top) and reference pattern (bottom) of $Cs_2AgSbCl_6$. (b) Optical absorption (black) and PL (red) spectra of $Cs_2AgSbCl_6$.

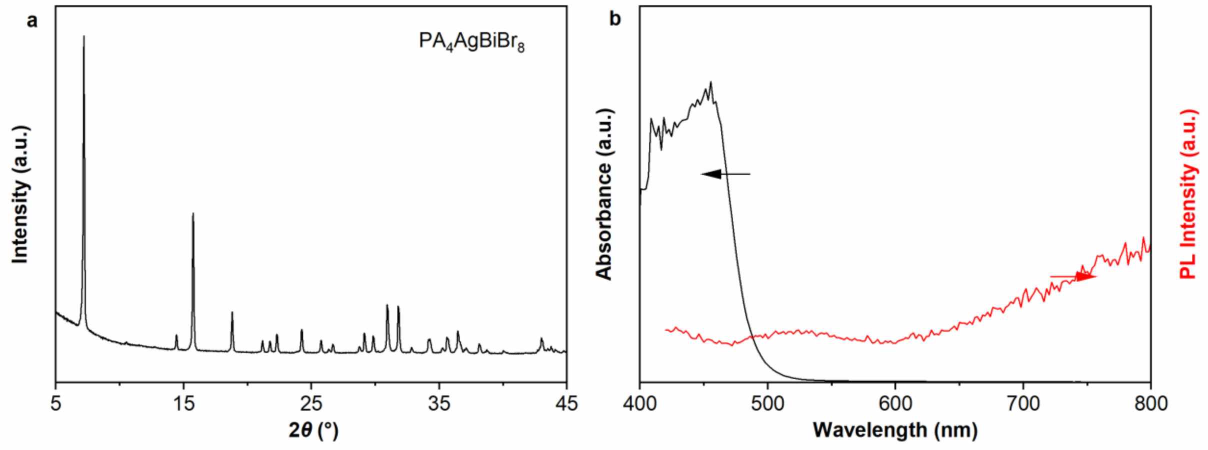

Figure S32. (a) XRD data of $PA_4AgBiBr_8$. (b) Optical absorption (black) and PL (red) spectra of $PA_4AgBiBr_8$.

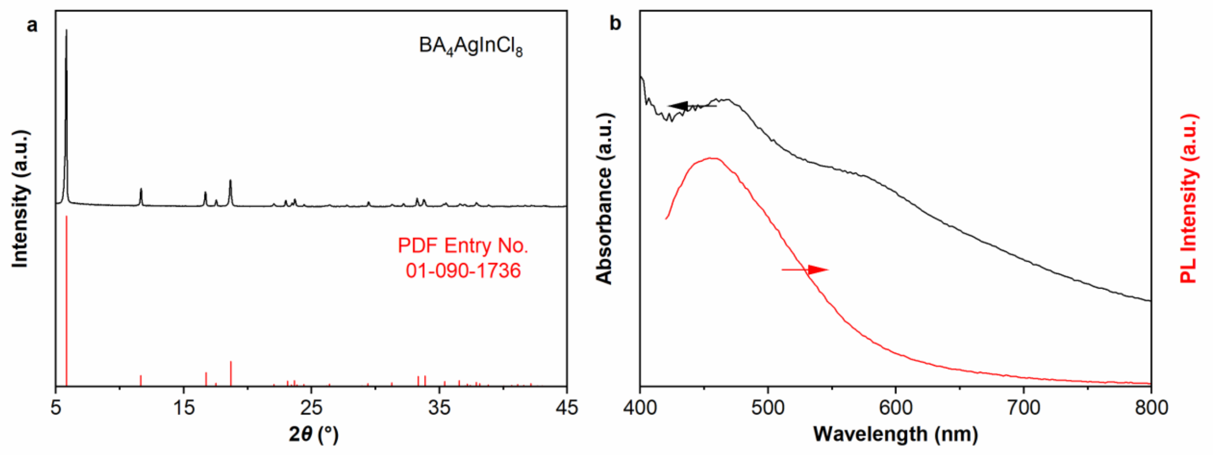

Figure S33. (a) XRD data (top) and reference pattern (bottom) of $BA_4AgInCl_8$. (b) Optical absorption (black) and PL (red) spectra of $BA_4AgInCl_8$.

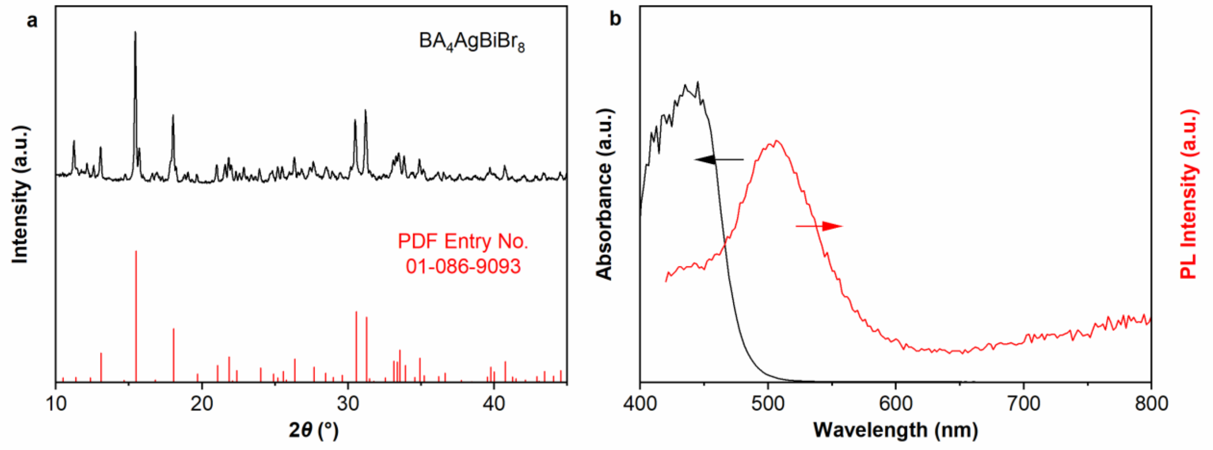

Figure S34. (a) XRD data (top) and reference pattern (bottom) of $BA_4AgBiBr_8$. (b) Optical absorption (black) and PL spectra of $BA_4AgBiBr_8$.

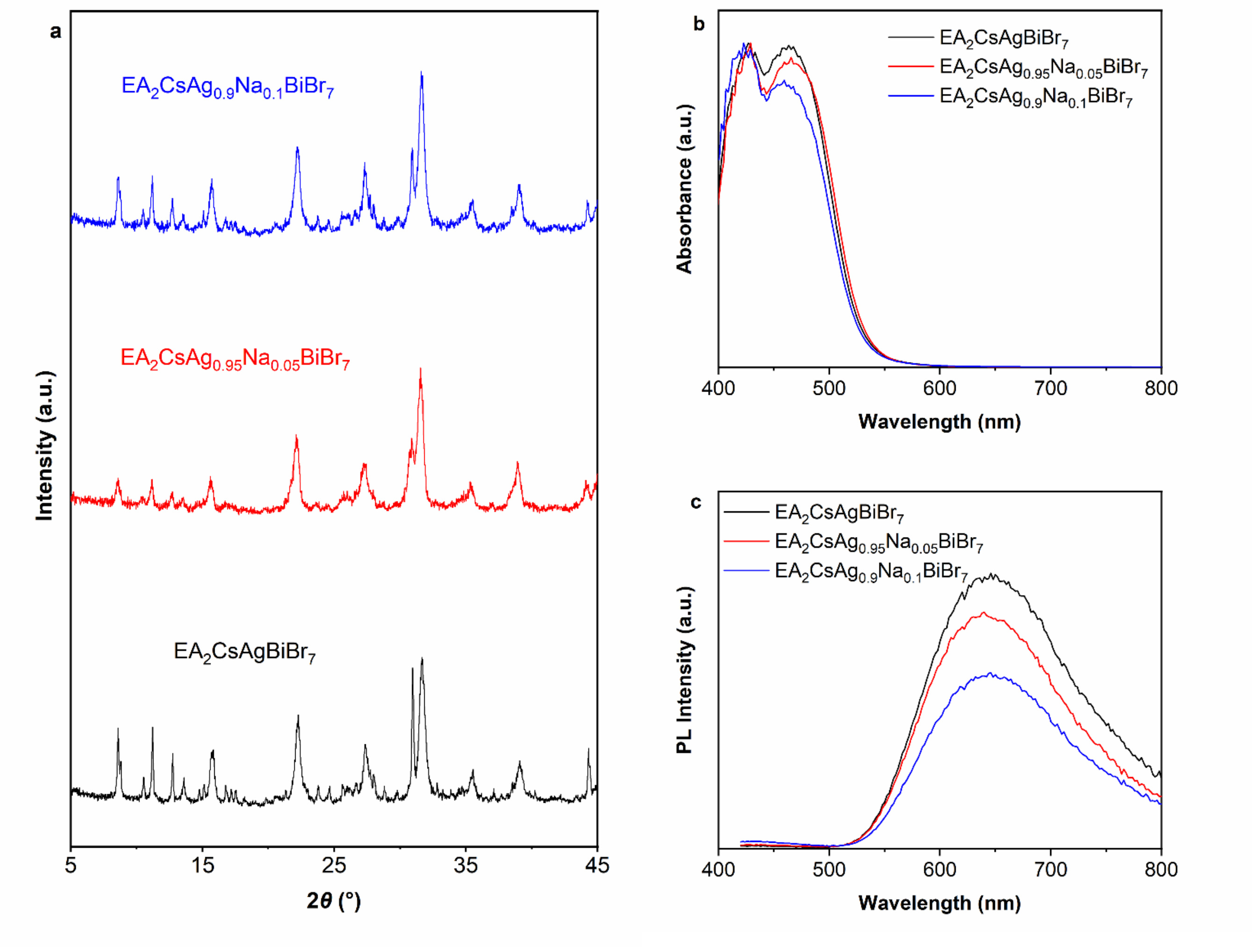

Figure S35. (a) XRD data, (b) optical absorption spectra, and (c) PL spectra of $EA_2CsAg_nNa_{1-n}BiBr_7$ (n = 1, 0.95, 0.9).

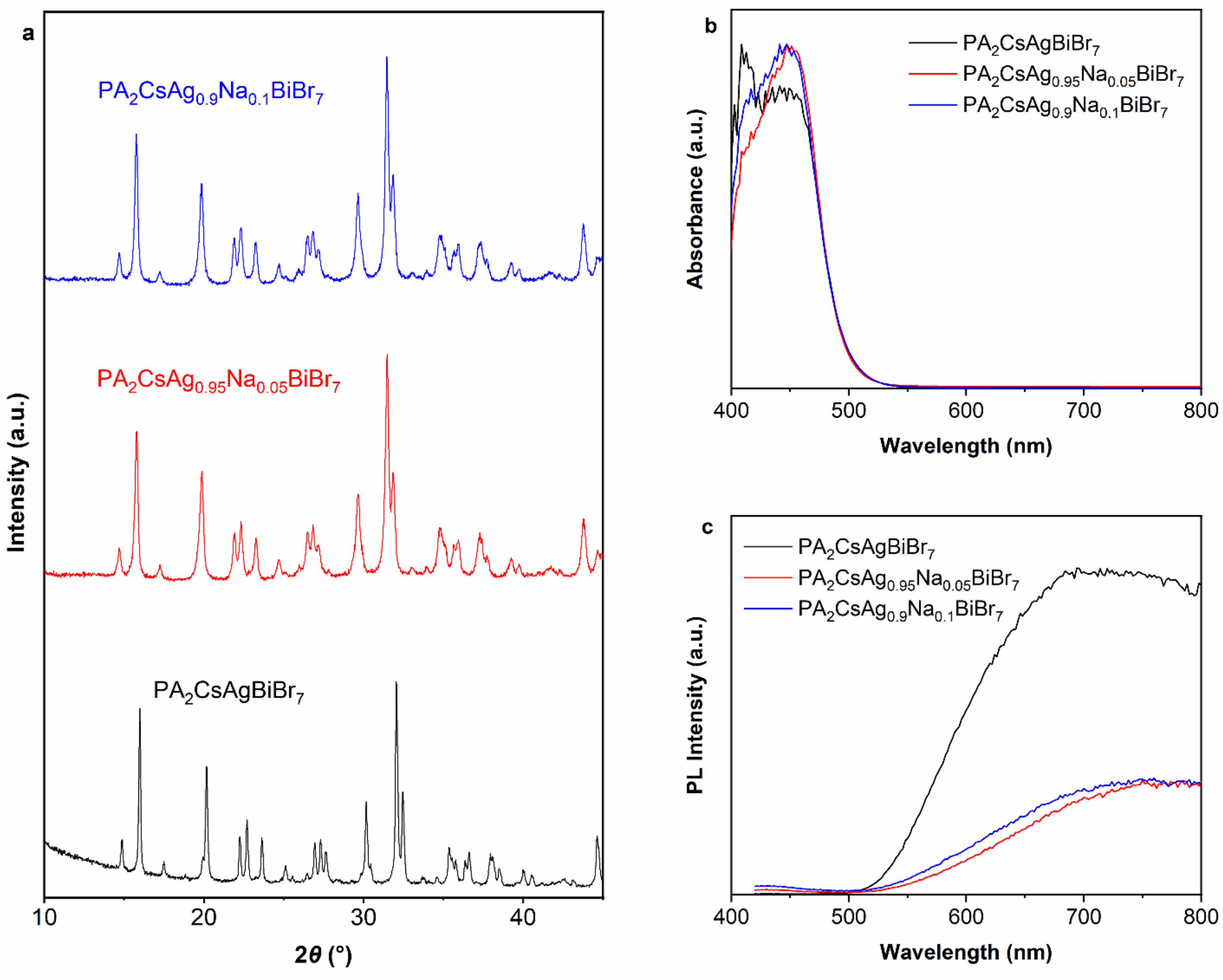

Figure S36. (a) XRD data, (b) optical absorption spectra, and (c) PL spectra of $PA_2CsAg_oNa_{1-o}BiBr_7$ (o = 1, 0.95, 0.9).

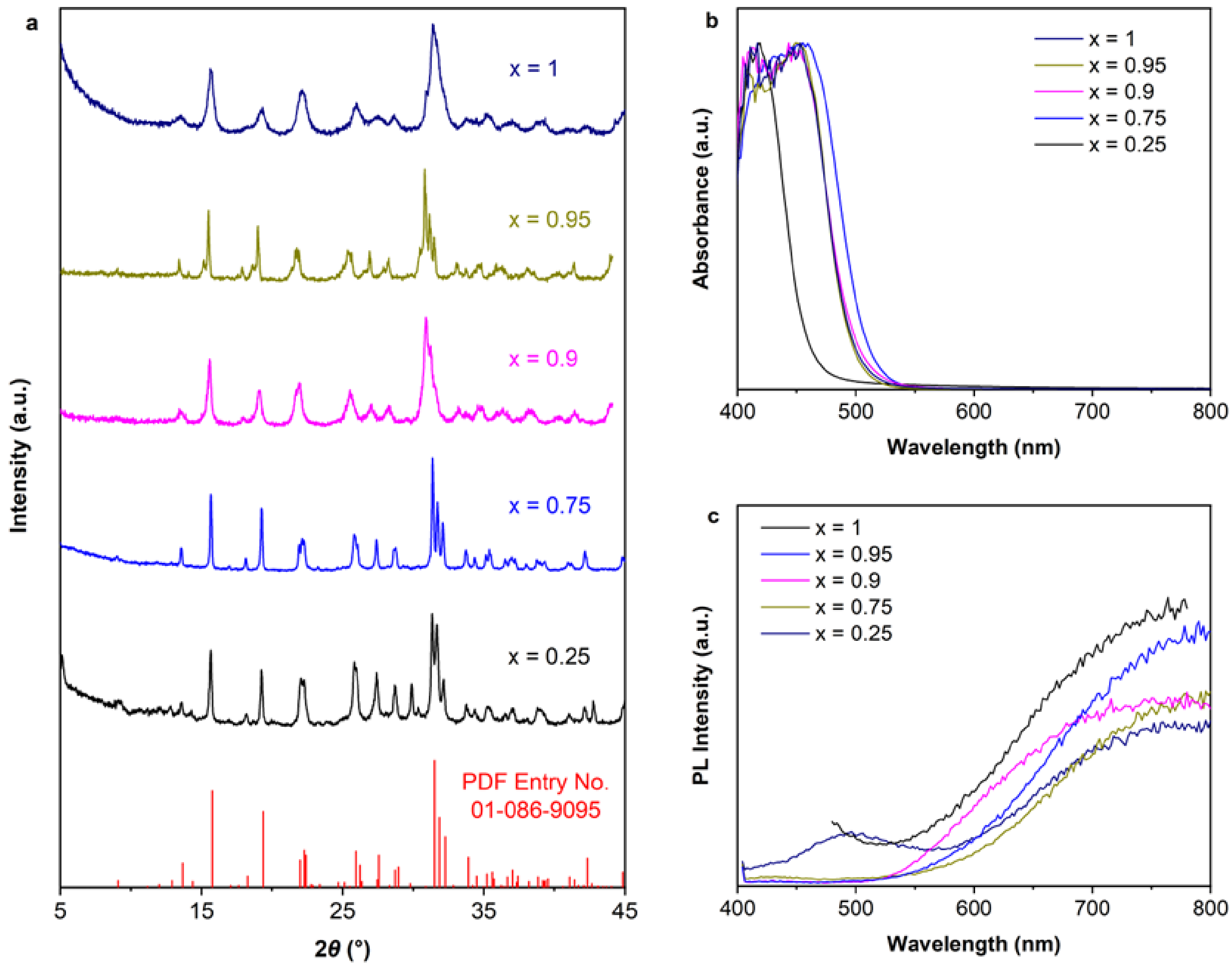

Figure S37. (a) XRD data, (b) optical absorption spectra, and (c) PL spectra of $BA_2CsAg_pNa_{1-p}BiBr_7$ (p = 1, 0.95, 0.9, 0.75, 0.25).

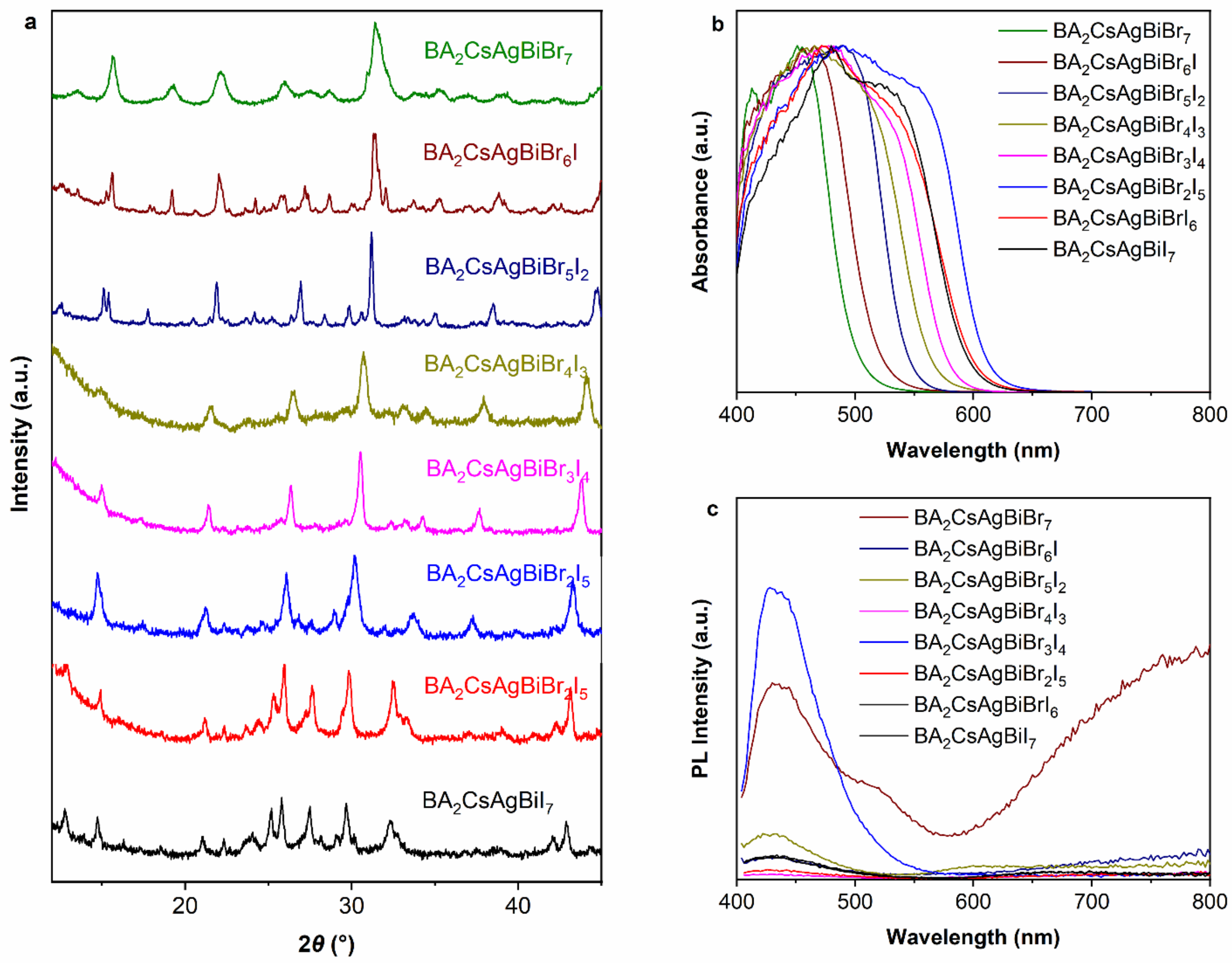

Figure S38. (a) XRD data, (b) optical absorption spectra, and (c) PL spectra of $BA_2CsAgBiBr_qI_{7-q}$ (q = 0, 1, 2, 3, 4, 5, 6).

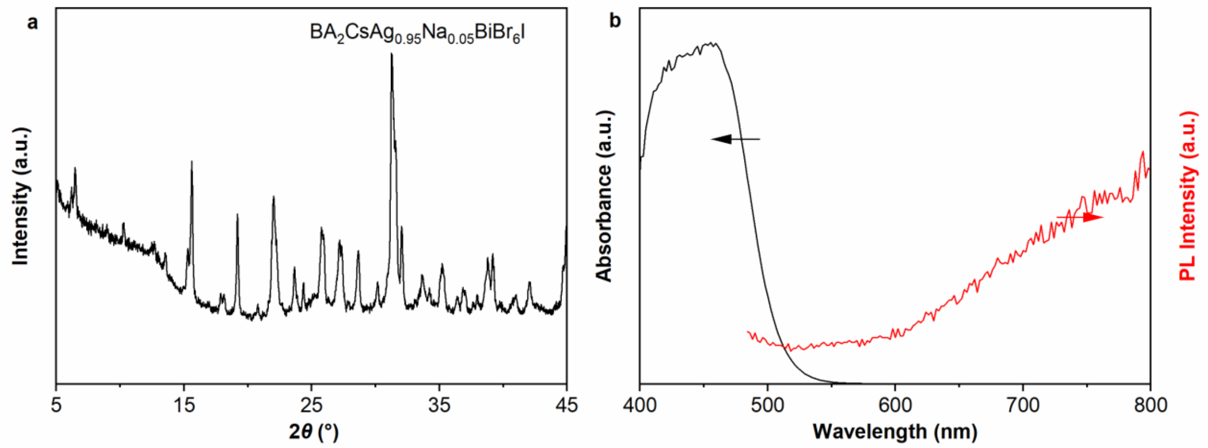

Figure S39. (a) XRD data, (b) optical absorption spectra (black), and PL(red) spectra of $BA_2CsAg_{0.95}Na_{0.05}BiBr_6I$.

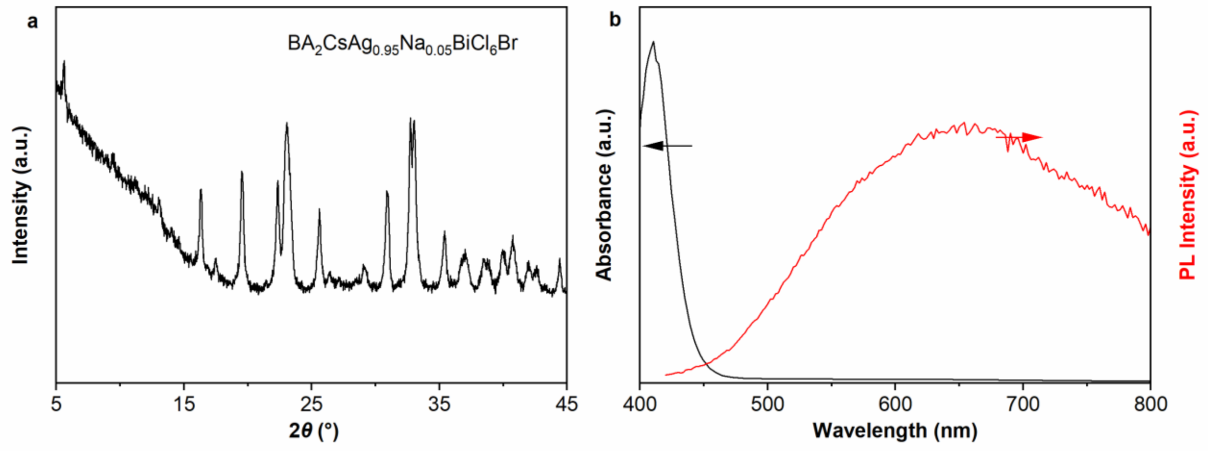

Figure S40. (a) XRD data, (b) optical absorption spectra (black), and PL (red) spectra of $BA_2CsAg_{0.95}Na_{0.05}BiCl_6Br$.

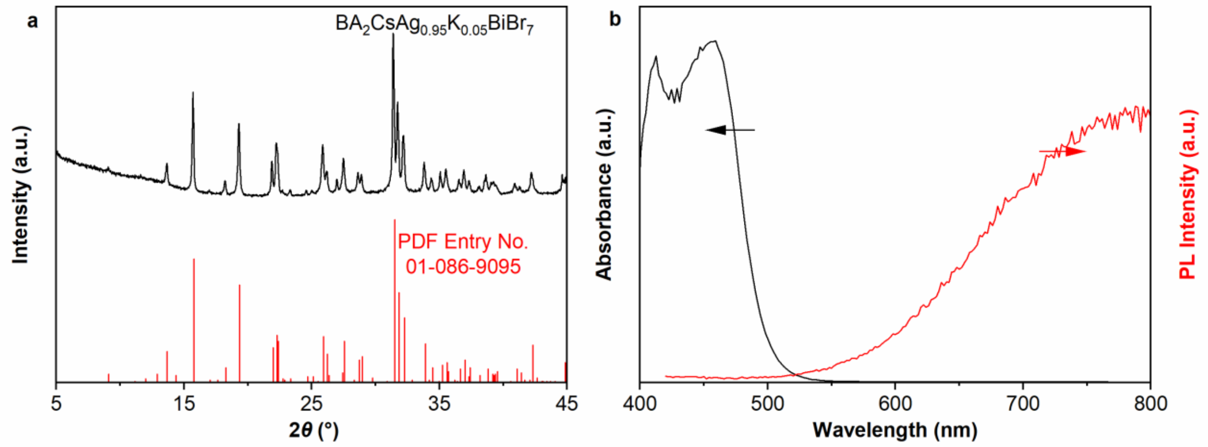

Figure S41. (a) XRD data (top) of $BA_2CsAg_{0.95}K_{0.05}BiBr_7$ and reference pattern (bottom) of $BA_2CsAgBiBr_7$. (b) Optical absorption (black) and PL (red) spectra of $BA_2CsAg_{0.95}K_{0.05}BiBr_7$.

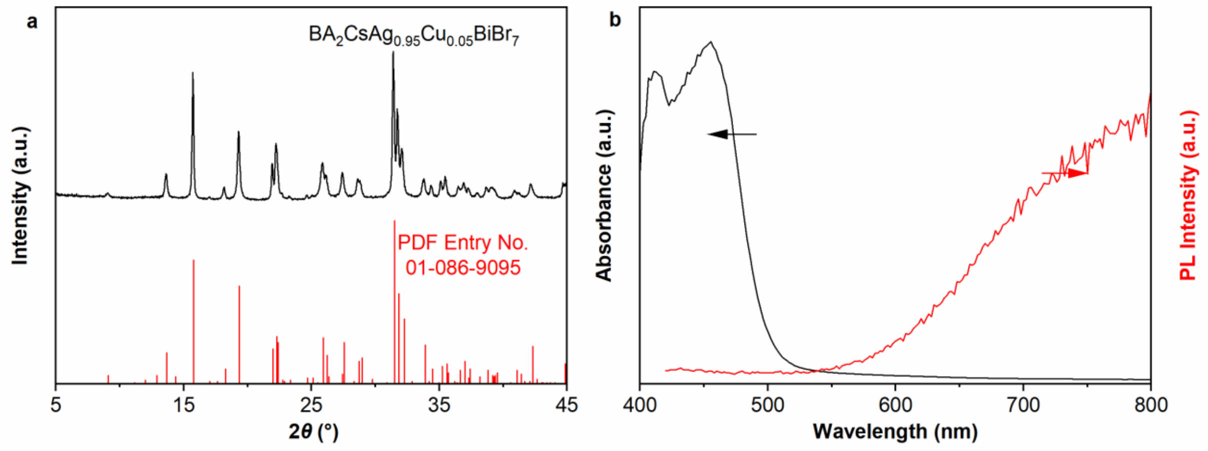

Figure S42. (a) XRD data (top) $BA_2CsAg_{0.95}Cu(I)_{0.05}BiBr_7$ and reference pattern (bottom) of $BA_2CsAgBiBr_7$. (b) Optical absorption (black) and PL (red) spectra of $BA_2CsAg_{0.95}Cu(I)_{0.05}BiBr_7$.

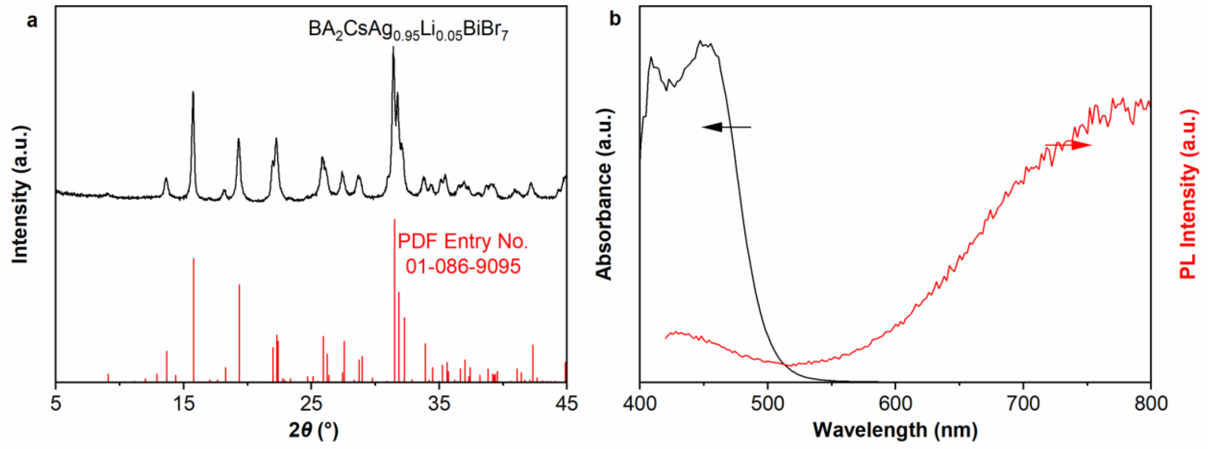

Figure S43. (a) XRD data (top) $BA_2CsAg_{0.95}Li_{0.05}BiBr_7$ and reference pattern (bottom) of $BA_2CsAgBiBr_7$. (b) Optical absorption (black) and PL (red) spectra of $BA_2CsAg_{0.95}Li_{0.05}BiBr_7$.

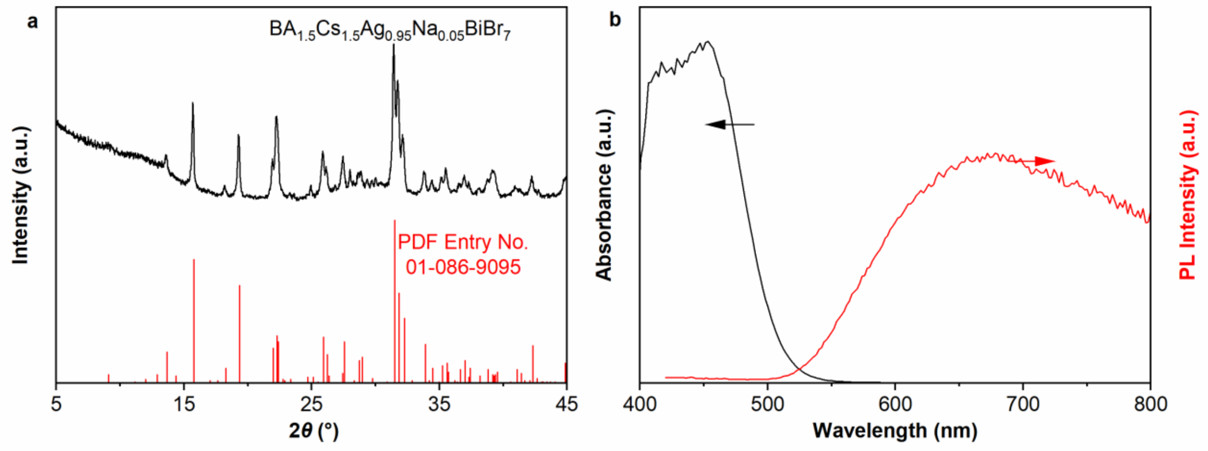

Figure S44. (a) XRD data (top) of $BA_{1.5}Cs_{1.5}Ag_{0.95}Na_{0.05}BiBr_7$ and reference pattern (bottom) of $BA_2CsAgBiBr_7$. (b) Optical absorption (black) and PL (red) spectra of $BA_{1.5}Cs_{1.5}Ag_{0.95}Na_{0.05}BiBr_7$.

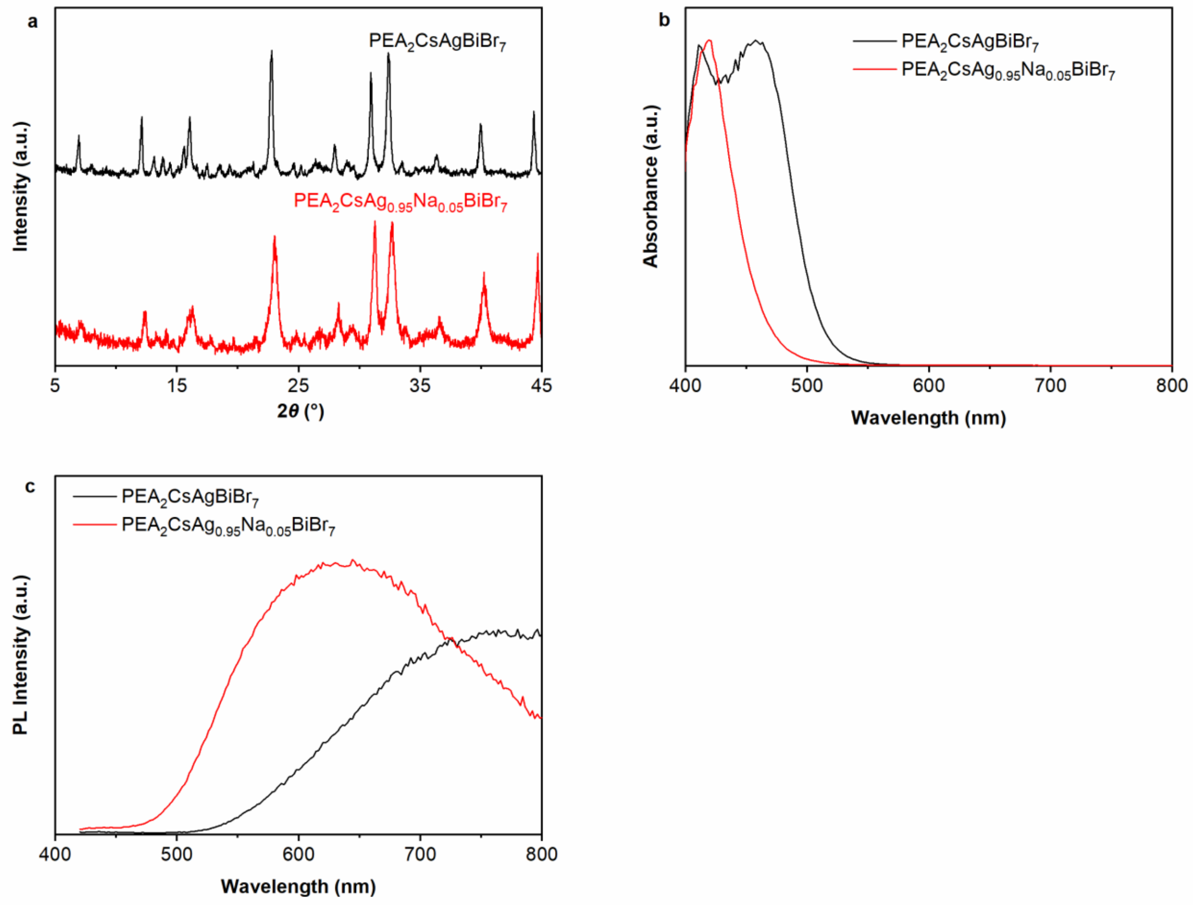

Figure S45. (a) XRD data, (b) optical absorption spectra, and (c) PL spectra of $PEA_2CsAg_rNa_{1-r}BiBr_7$ (r = 1, 0.95).

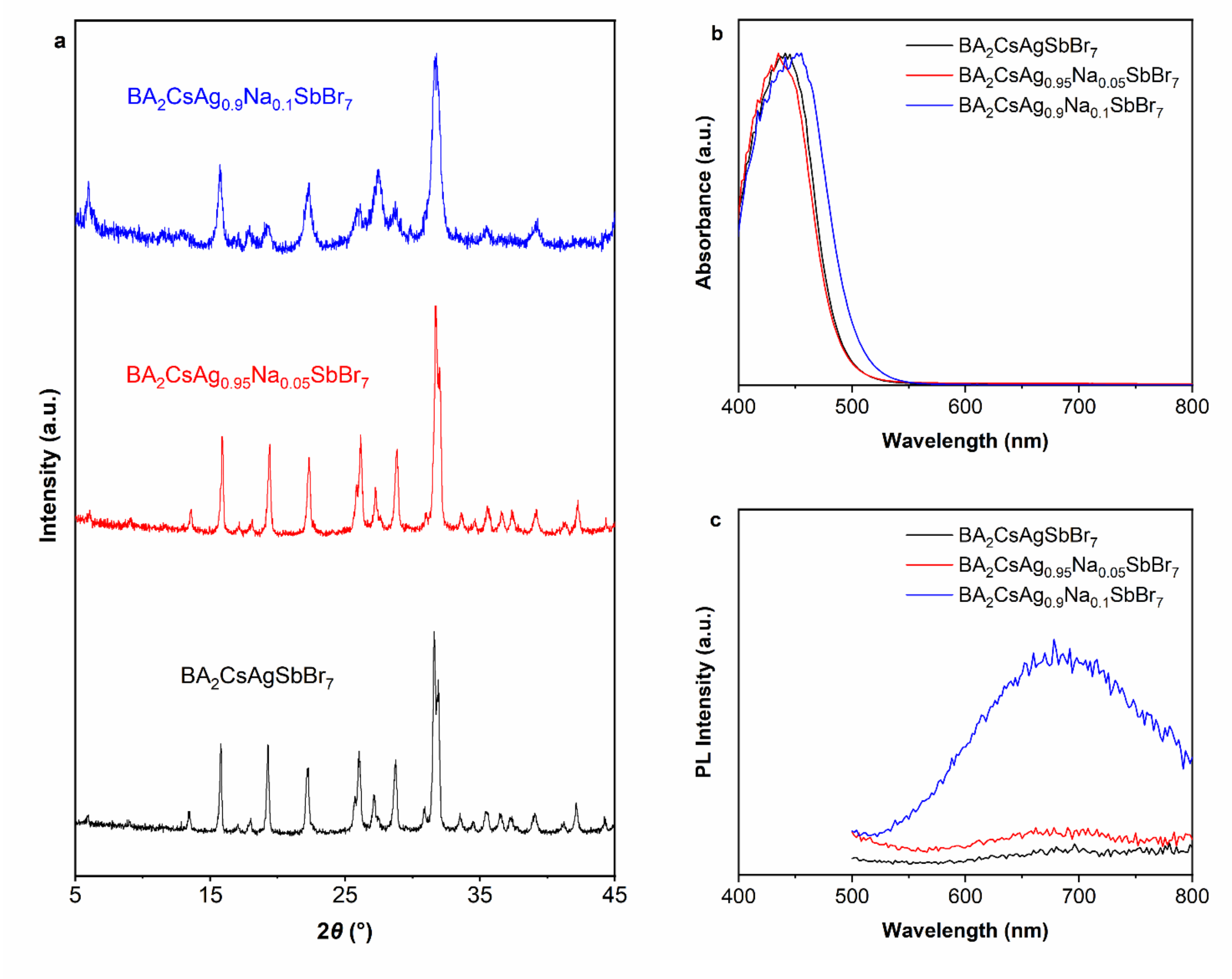

Figure S46. (a) XRD data, (b) optical absorption spectra, and (c) PL spectra of $BA_2CsAg_sNa_{1-s}SbBr_7$ (s = 1, 0.95, 0.9).

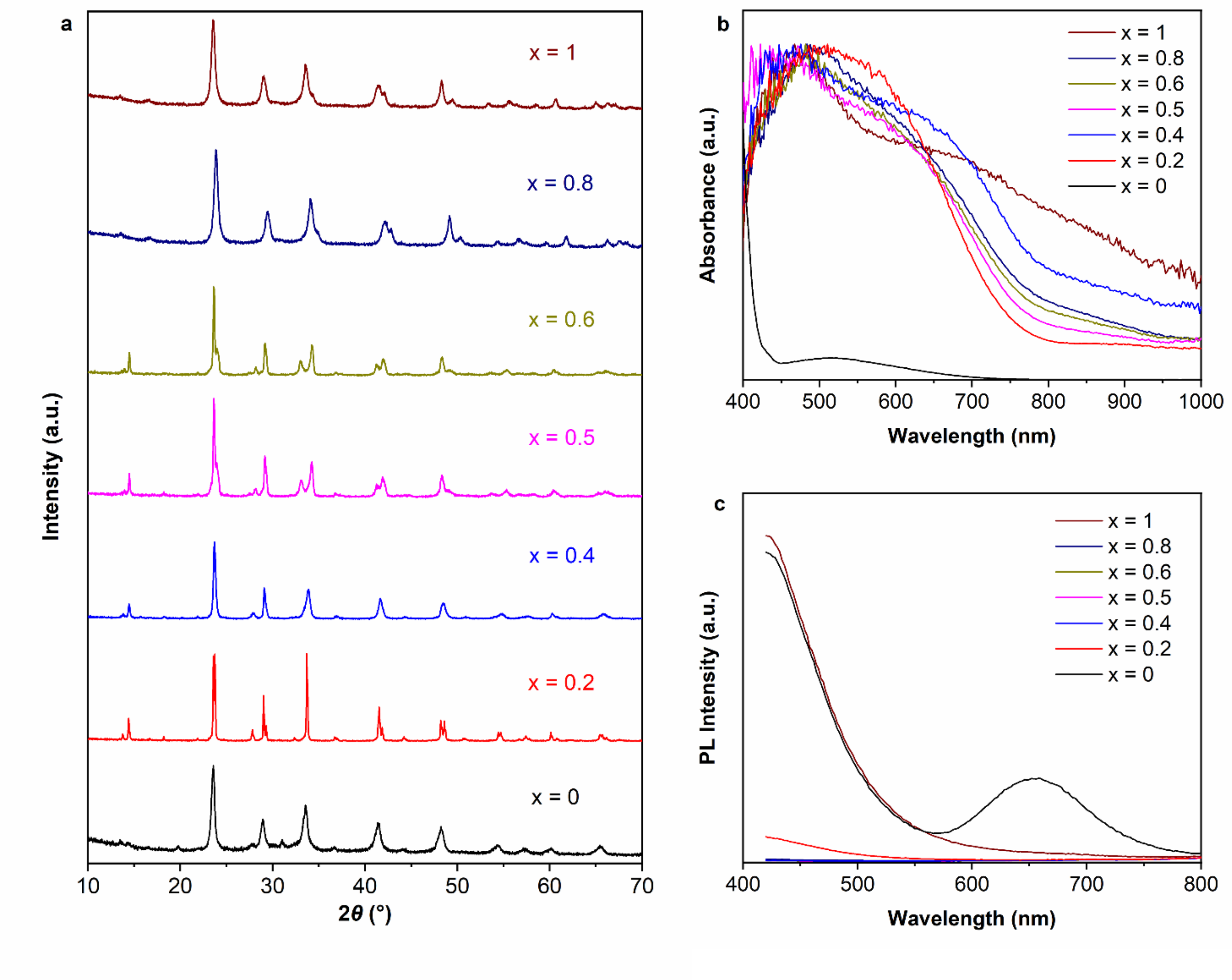

Figure S47. (a) XRD data, (b) optical absorption spectra, and (c) PL spectra of $Cs_4Cu_tMn_{1-t}Sb_2Cl_{12}$ (t = 0, 0.2, 0.4, 0.5, 0.6, 0.8, 1).

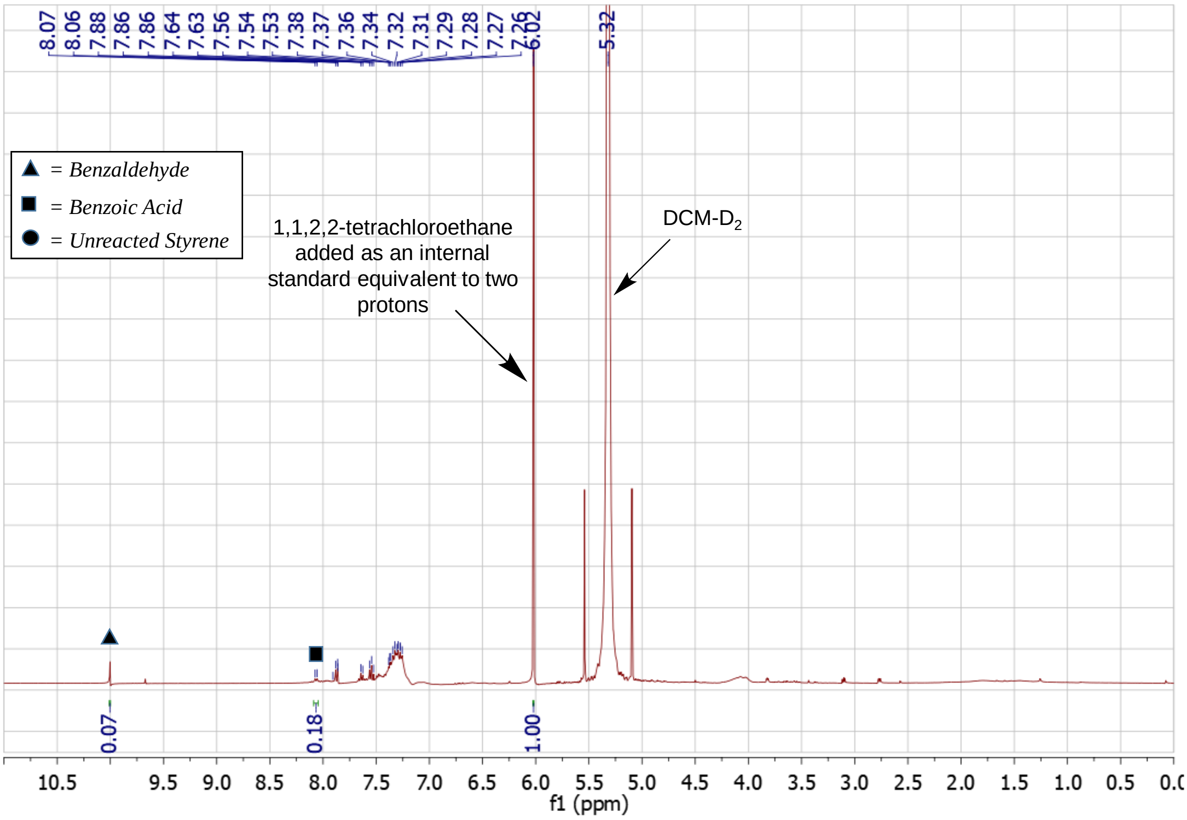

Figure S48: ^{1}H NMR spectrum of the styrene oxidation reaction in CD_2Cl_2 using $Cs_2AgBiBr_6$ as a photocatalyst under neat conditions with a 50 W white LED as the light source.

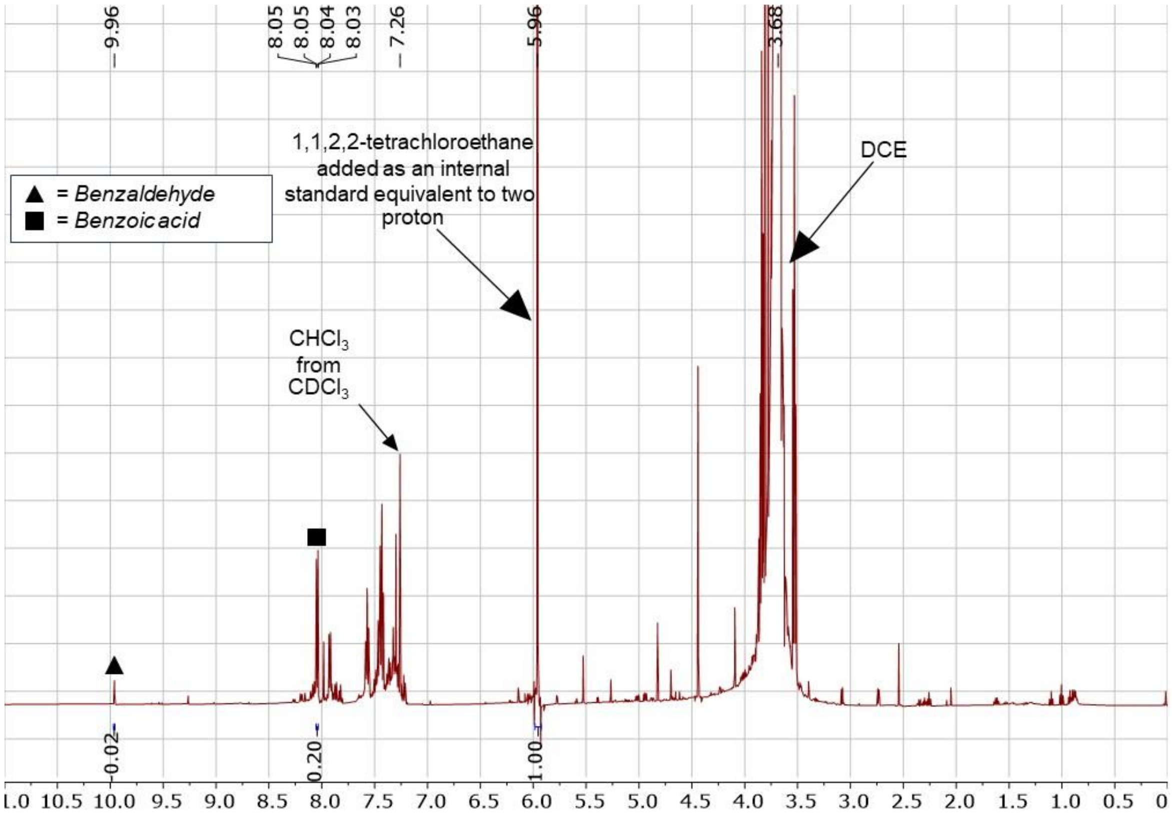

Figure S49: ^{1}H NMR spectrum of the styrene oxidation reaction in $CDCl_3$ using $Cs_2AgBiBr_6$ as a photocatalyst in DCE as a solvent with a 50 W white LED as the light source.

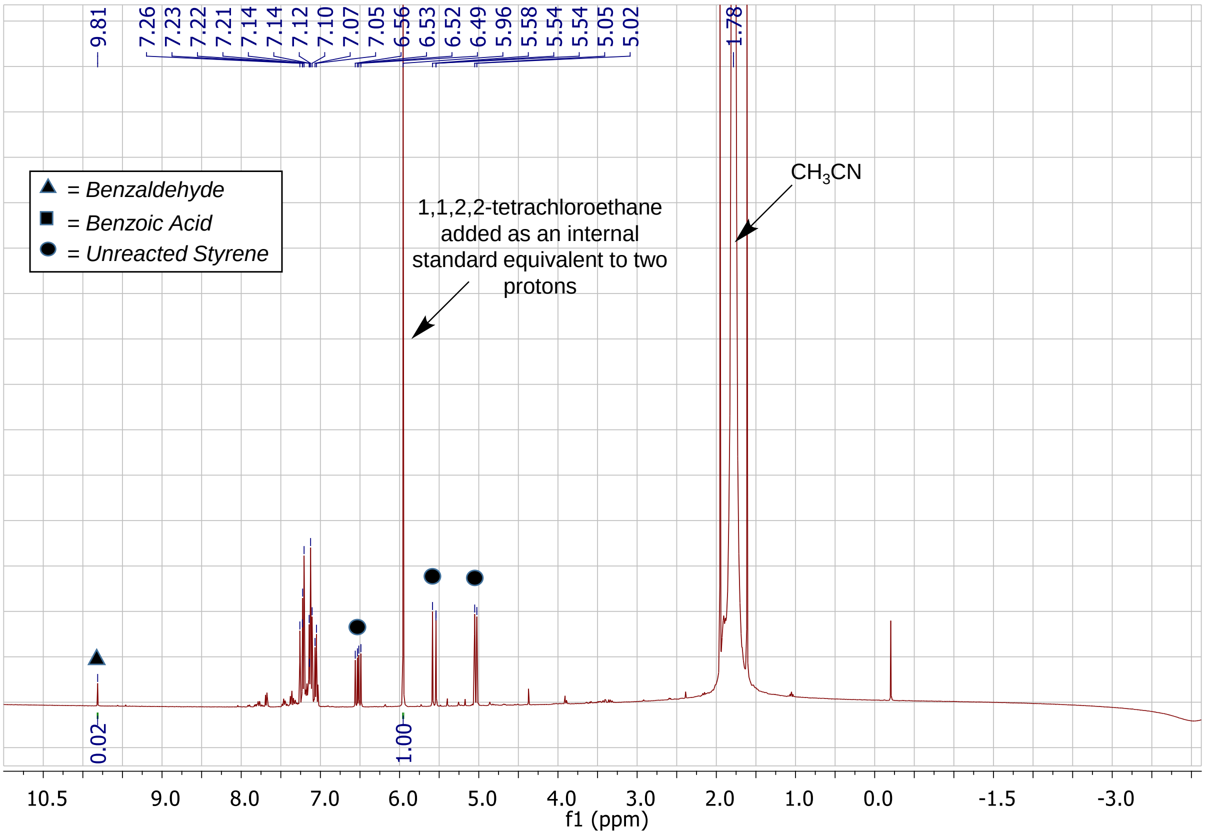

Figure S50: ^{1}H NMR spectrum of the styrene oxidation reaction in CD_3CN using $Cs_2AgBiBr_6$ as a photocatalyst in CH_3CN as a solvent with a 50 W white LED as the light source.

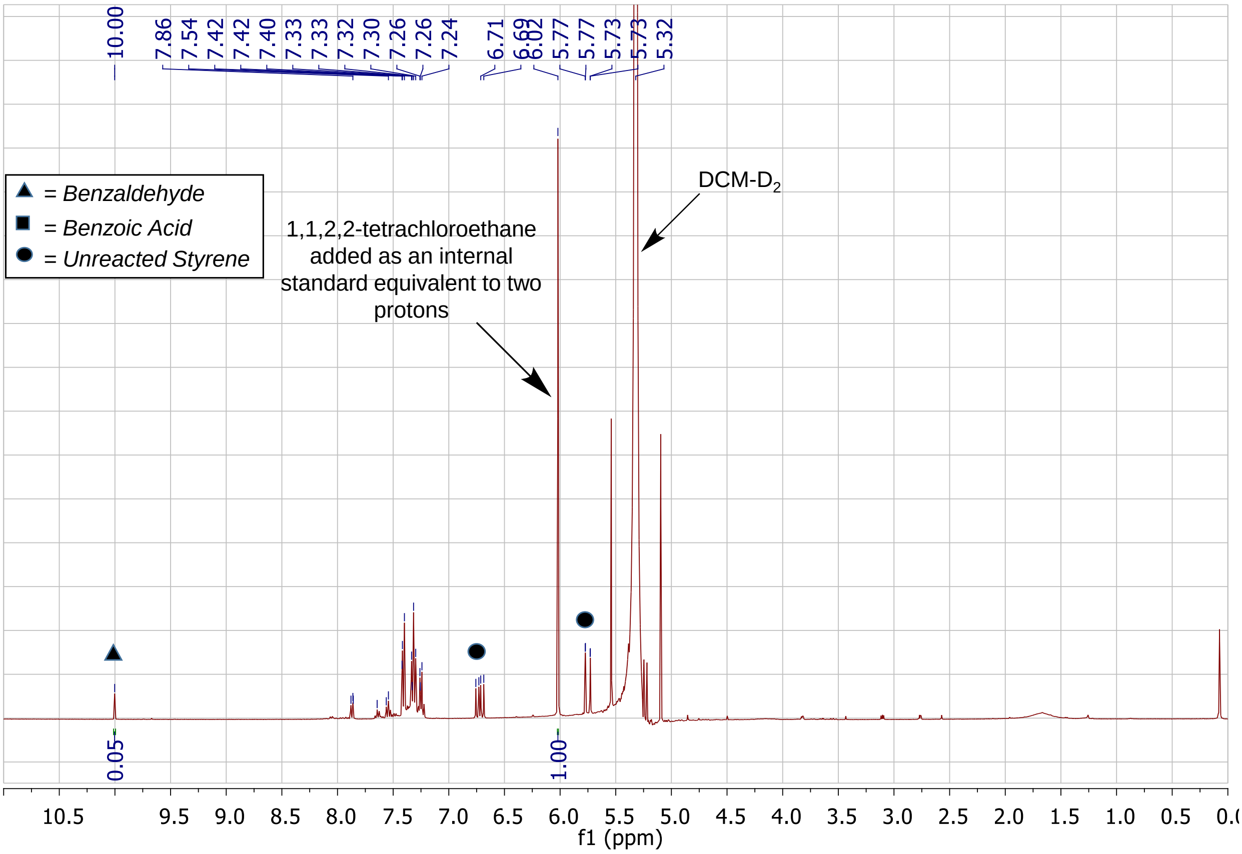

Figure S51: ^{1}H NMR spectrum of the styrene oxidation reaction in CD_2Cl_2 using $Cs_2AgBiBr_6$ as a photocatalyst in DCM as a solvent with a 50 W white LED as the light source.

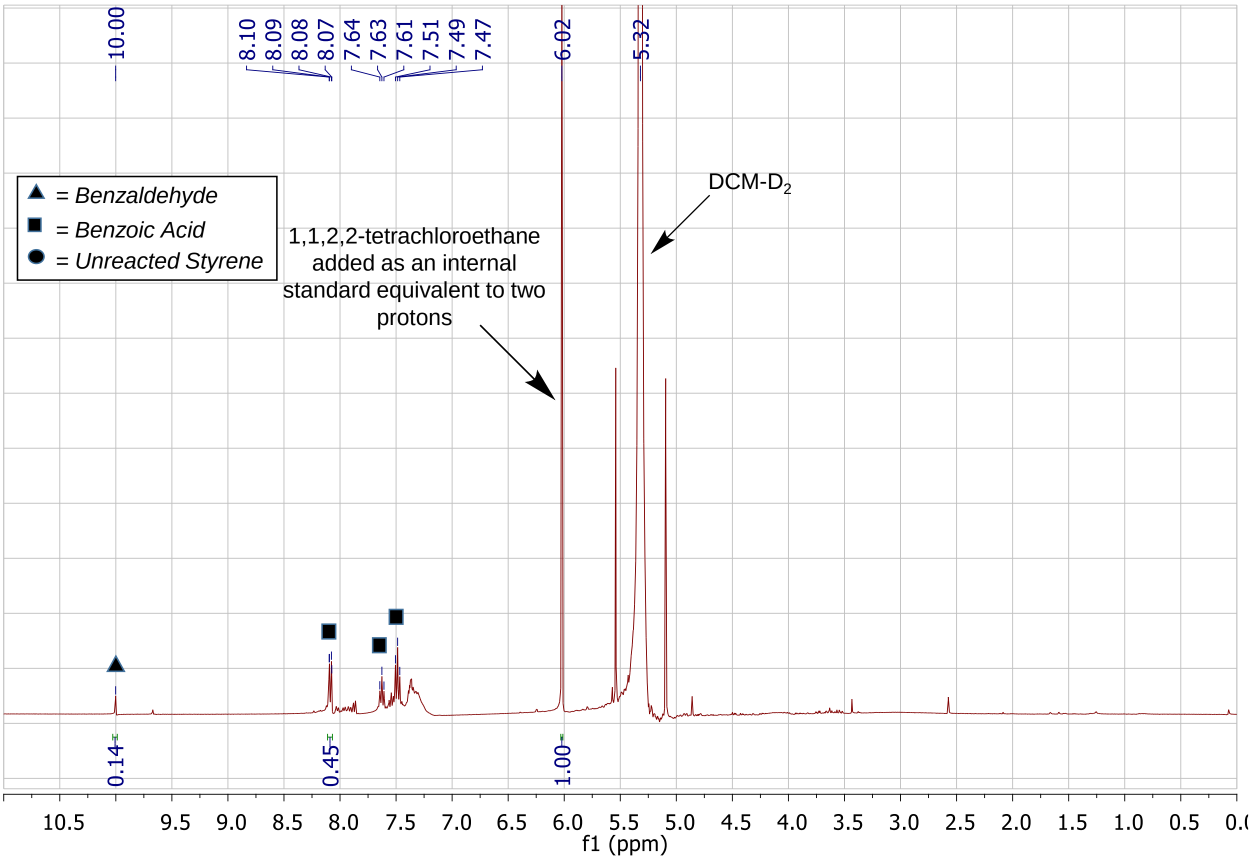

Figure S52: ^{1}H NMR spectrum of the styrene oxidation reaction in CD_2Cl_2 using $Cs_2AgBiBr_6$ as a photocatalyst under neat conditions with a 50 W blue LED as the light source.

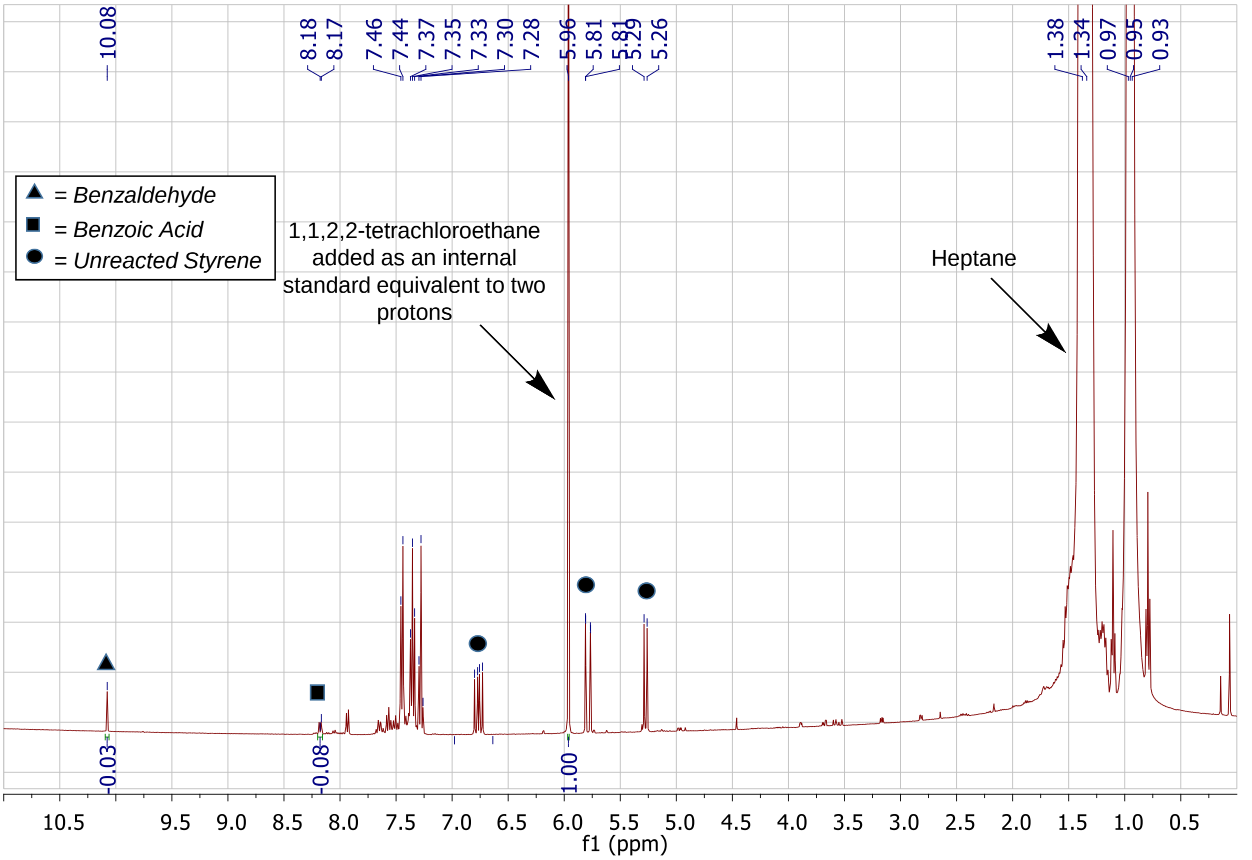

Figure S53: ^{1}H NMR spectrum of the styrene oxidation reaction in $CDCl_3$ using $Cs_2AgBiBr_6$ as a photocatalyst in heptane as a solvent with a 50 W blue LED as the light source.

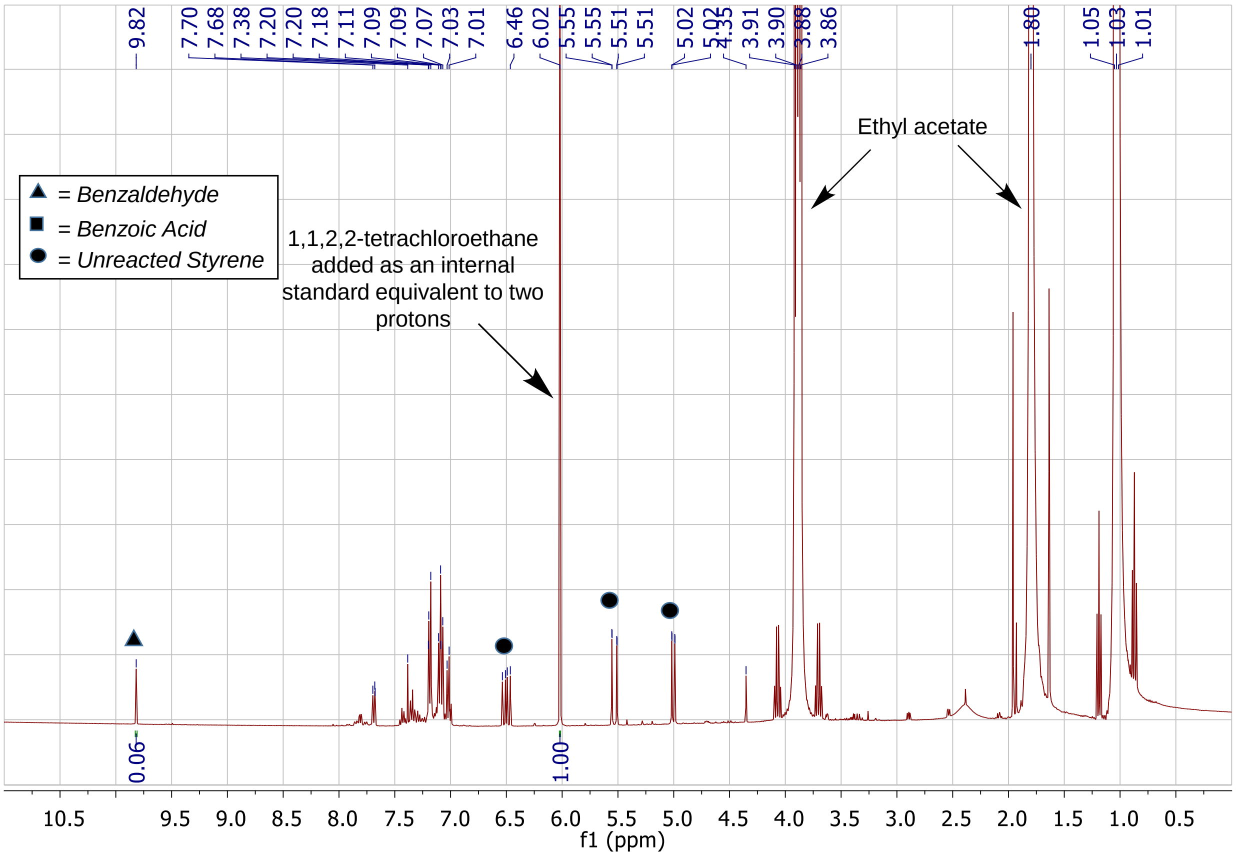

Figure S54: ^{1}H NMR spectrum of the styrene oxidation reaction in $CDCl_3$ using $Cs_2AgBiBr_6$ as a photocatalyst in ethyl acetate as a solvent with a 50 W blue LED as the light source.

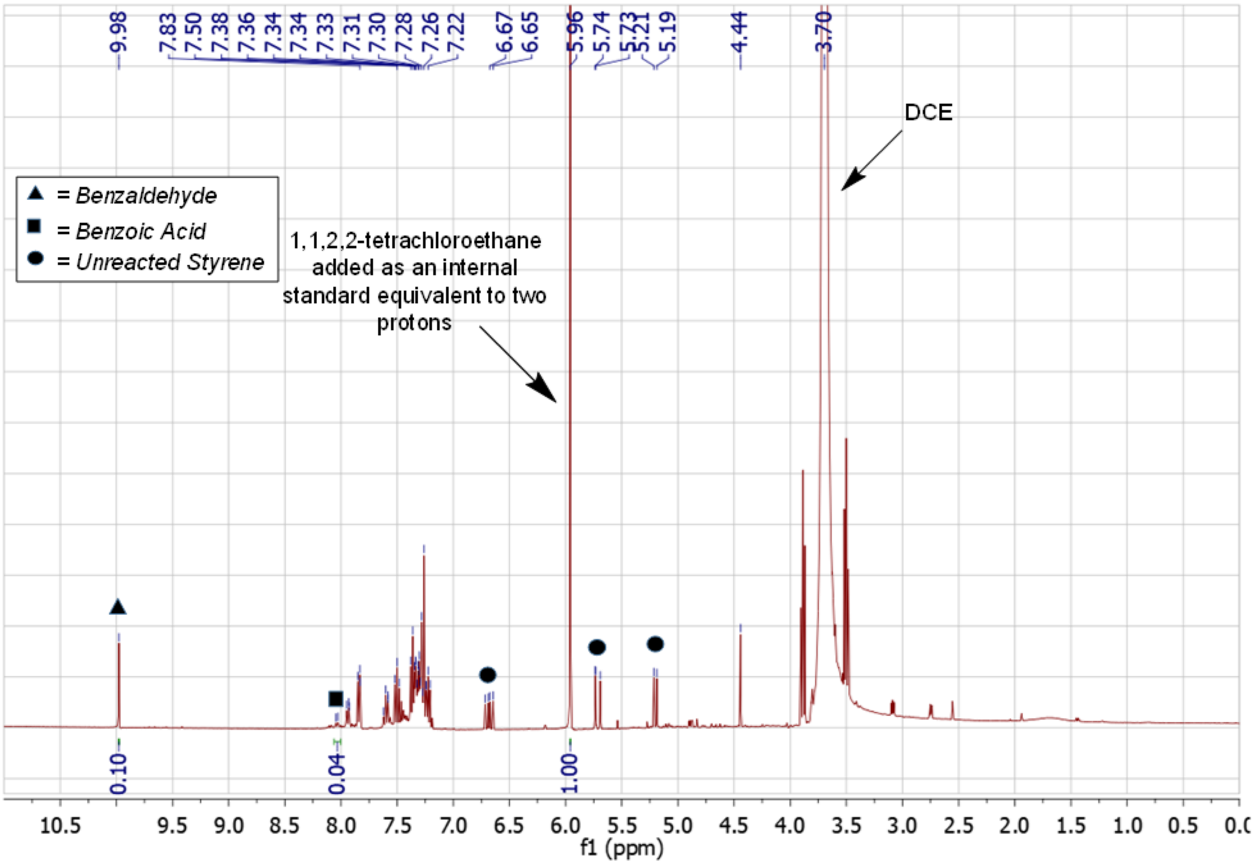

Figure S55: ^{1}H NMR spectrum of the styrene oxidation reaction in $CDCl_3$ using $Cs_2AgBiBr_6$ as a photocatalyst in DCE as a solvent with a 50 W blue LED as the light source.

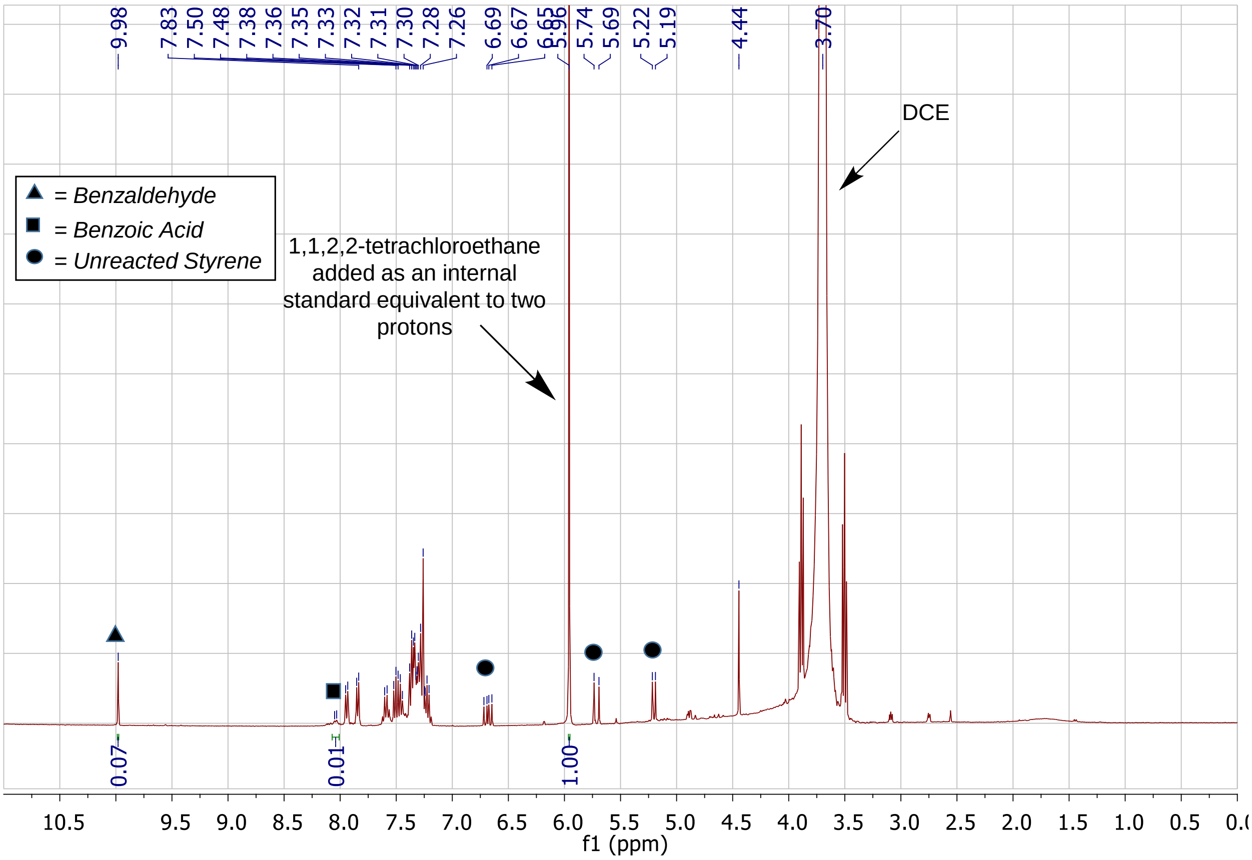

Figure S56: ^{1}H NMR spectrum of the styrene oxidation reaction in $CDCl_3$ using 10 mol% $Cs_2AgBiBr_6$ as a photocatalyst in DCE as a solvent with a 50 W blue LED as the light source.

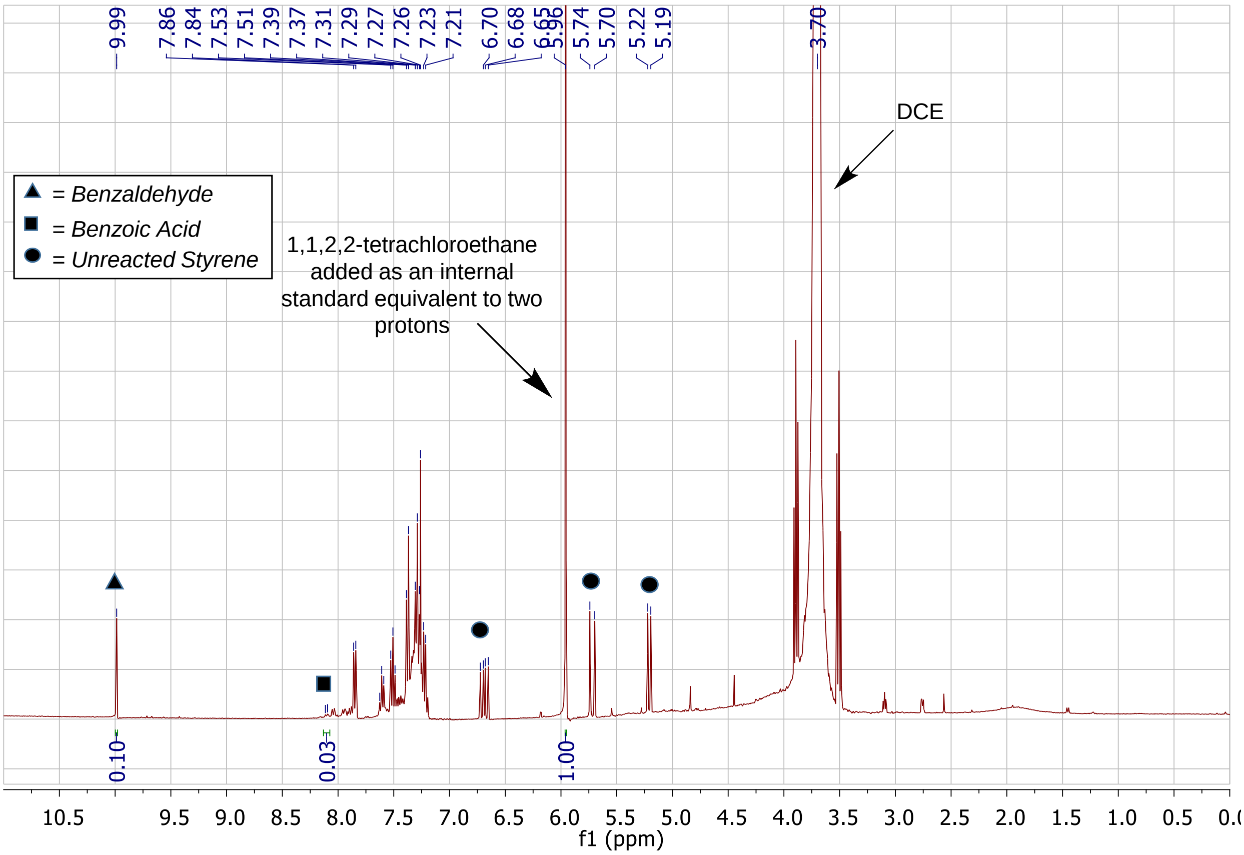

Figure S57: ^{1}H NMR spectrum of the styrene oxidation reaction in $CDCl_3$ using $Cs_2AgBiBr_3Cl_3$ as a photocatalyst in DCE as a solvent with a 50 W blue LED as the light source.

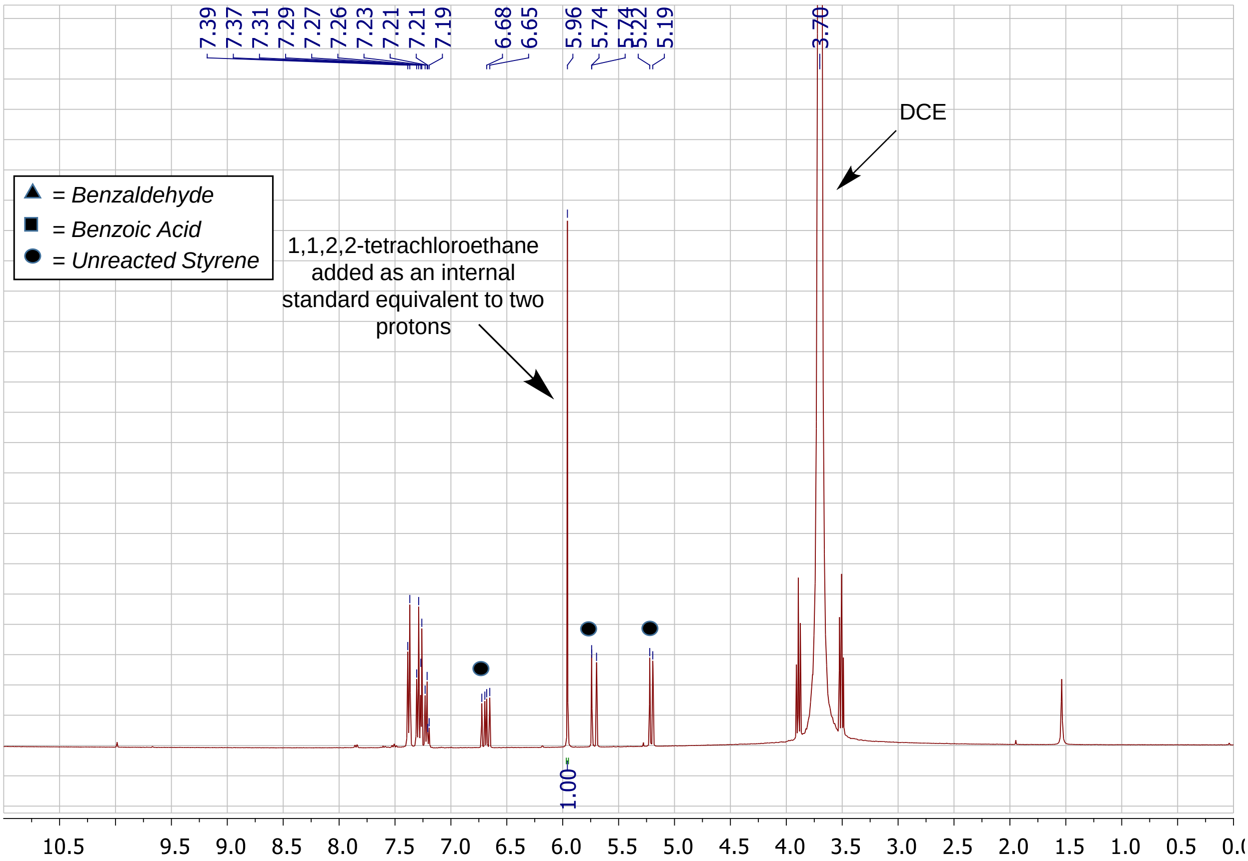

Figure S58: ^{1}H NMR spectrum of the styrene oxidation reaction in $CDCl_3$ using $Cs_2AgSbCl_6$ as a photocatalyst in DCE as a solvent with a 50 W blue LED as the light source.

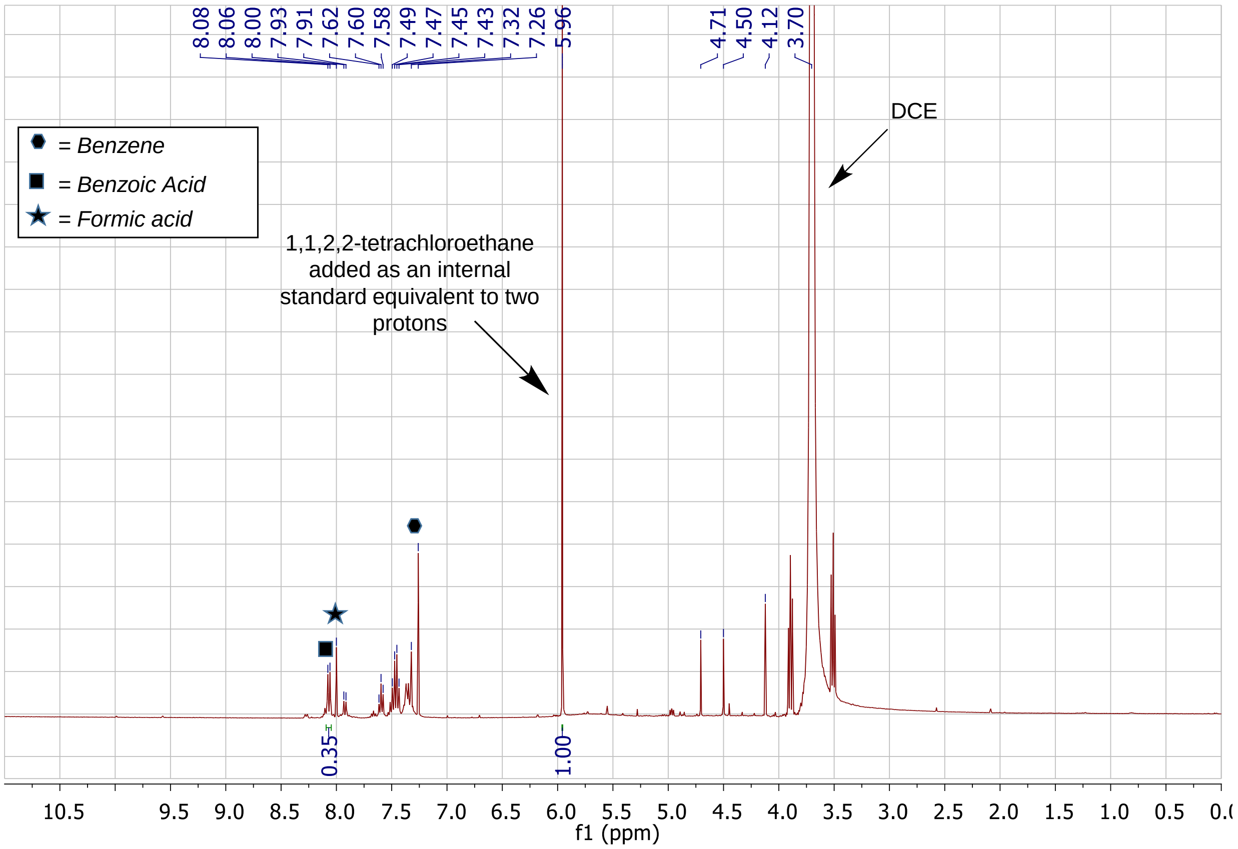

Figure S59: ^{1}H NMR spectrum of the styrene oxidation reaction in $CDCl_3$ using $Cs_2AgBiCl_5Br$ as a photocatalyst in DCE as a solvent with a 50 W blue LED as the light source.

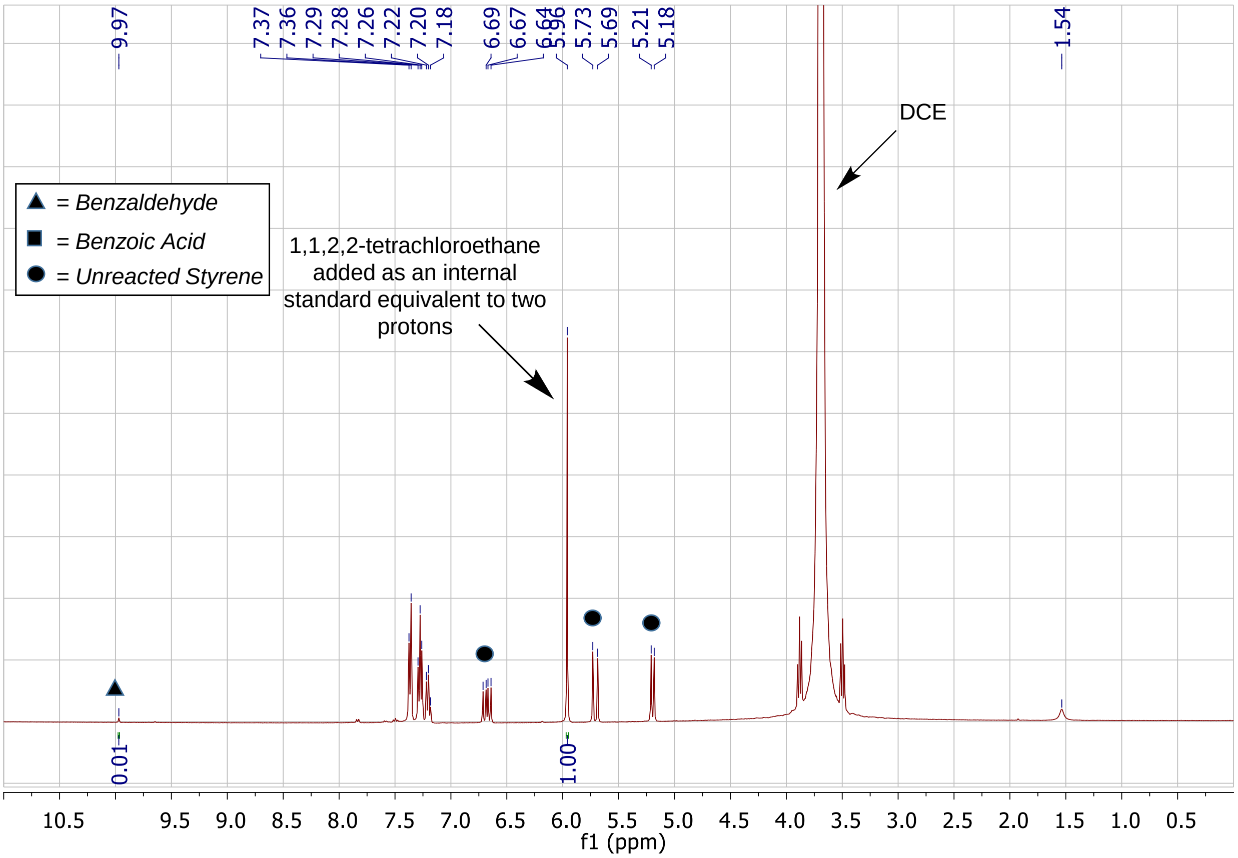

Figure S60: ^{1}H NMR spectrum of the styrene oxidation reaction in $CDCl_3$ using $Cs_4MnSb_2Cl_{12}$ as a photocatalyst in DCE as a solvent with a 50 W blue LED as the light source.

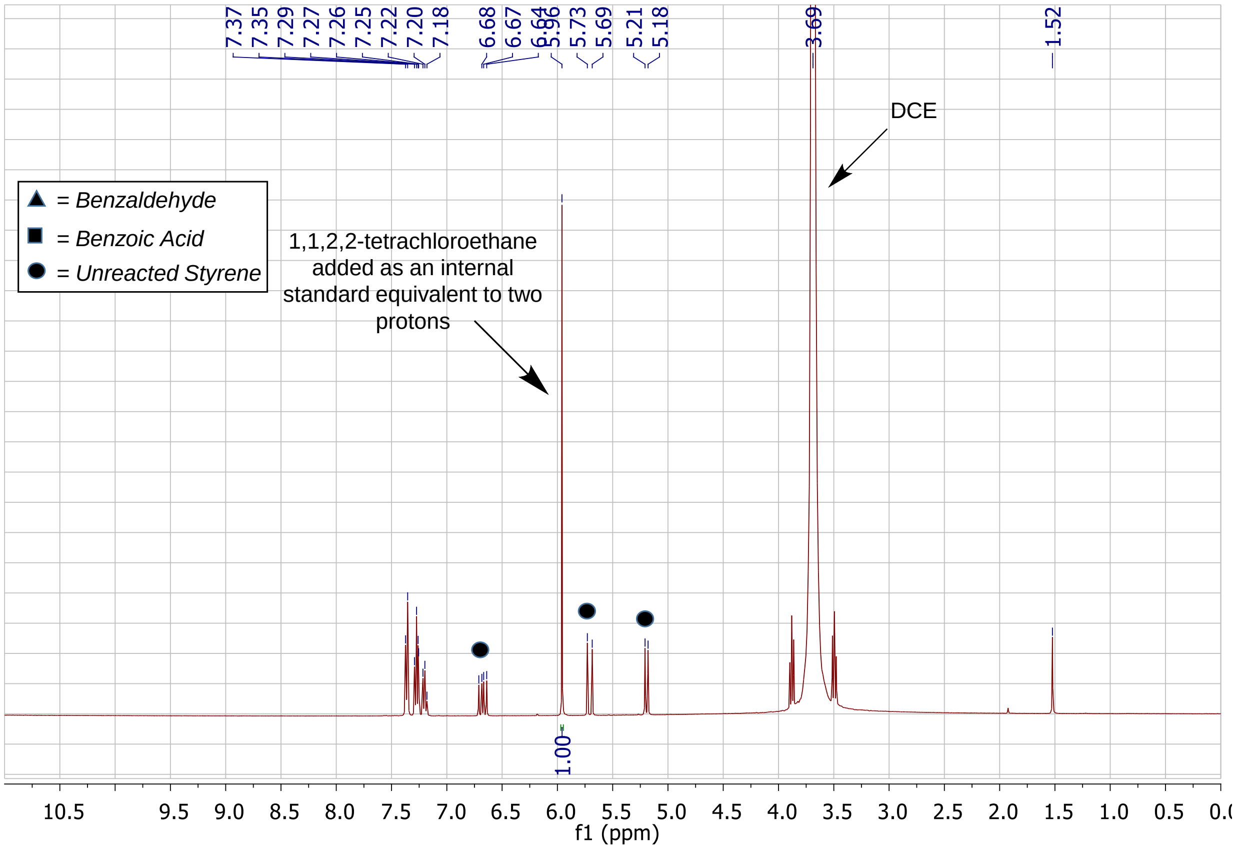

Figure S61: ^{1}H NMR spectrum of the styrene oxidation reaction in $CDCl_3$ using $Cs_4CuSb_2Cl_{12}$ as a photocatalyst in DCE as a solvent with a 50 W blue LED as the light source.

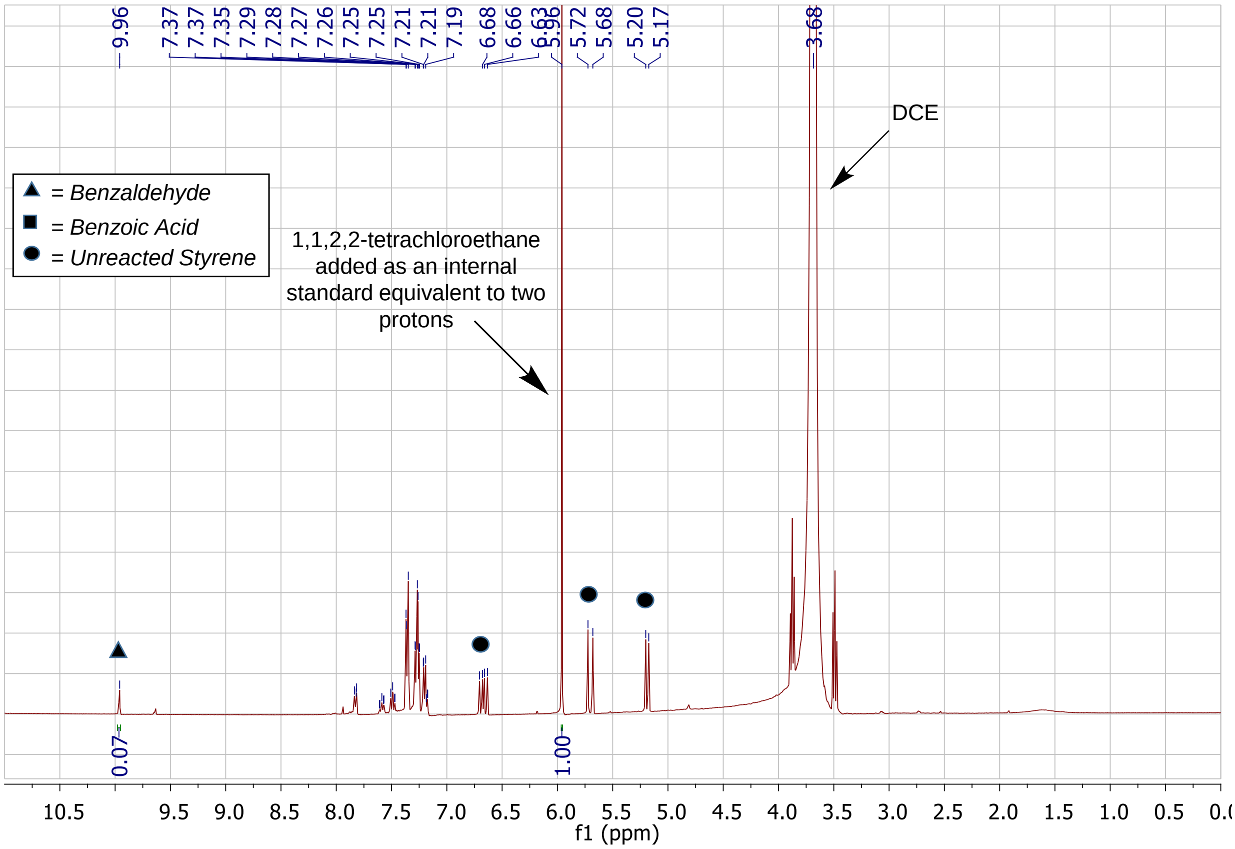

Figure S62: ^{1}H NMR spectrum of the styrene oxidation reaction in $CDCl_3$ using $K_2CsBiCl_6$ as a photocatalyst in DCE as a solvent with a 50 W blue LED as the light source.

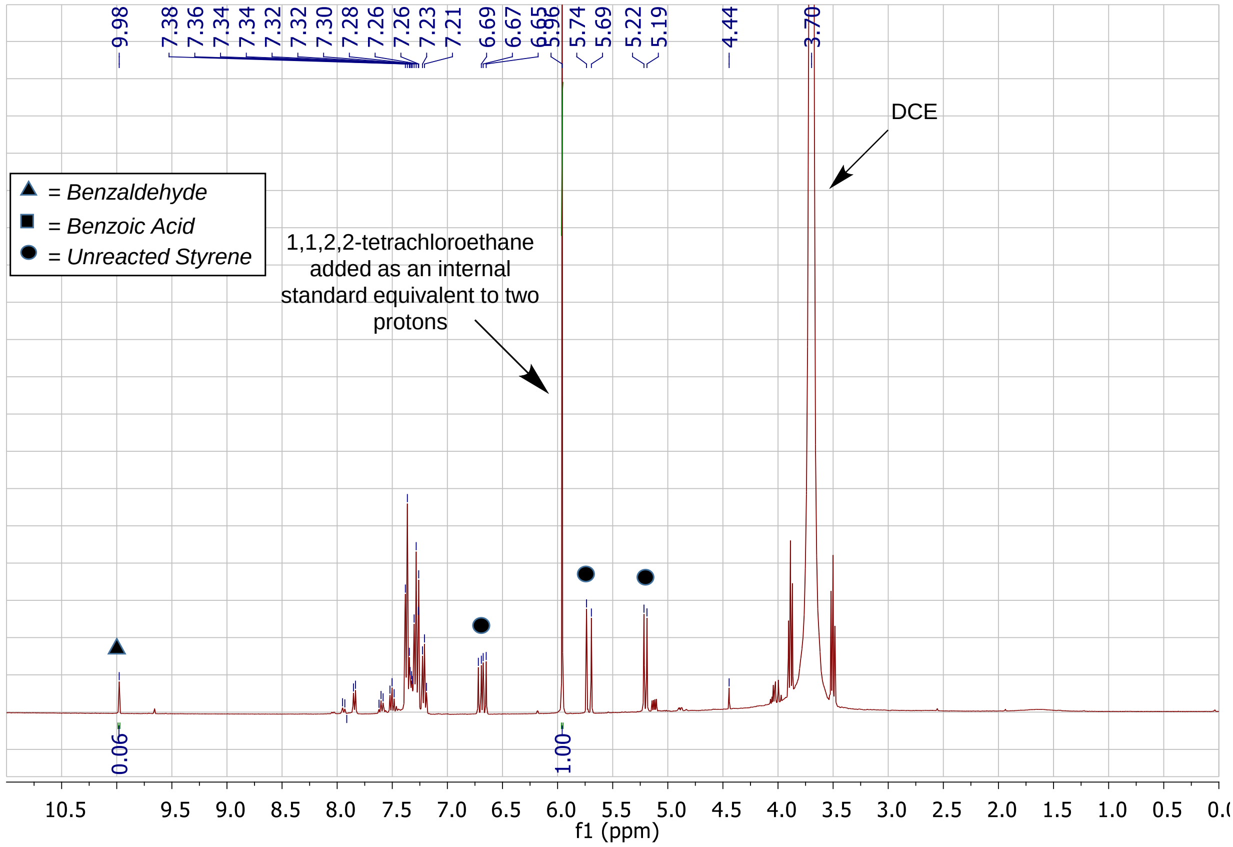

Figure S63: ^{1}H NMR spectrum of the styrene oxidation reaction in $CDCl_3$ using $Rb_3Sb_2Br_9$ as a photocatalyst in DCE as a solvent with a 50 W blue LED as the light source.

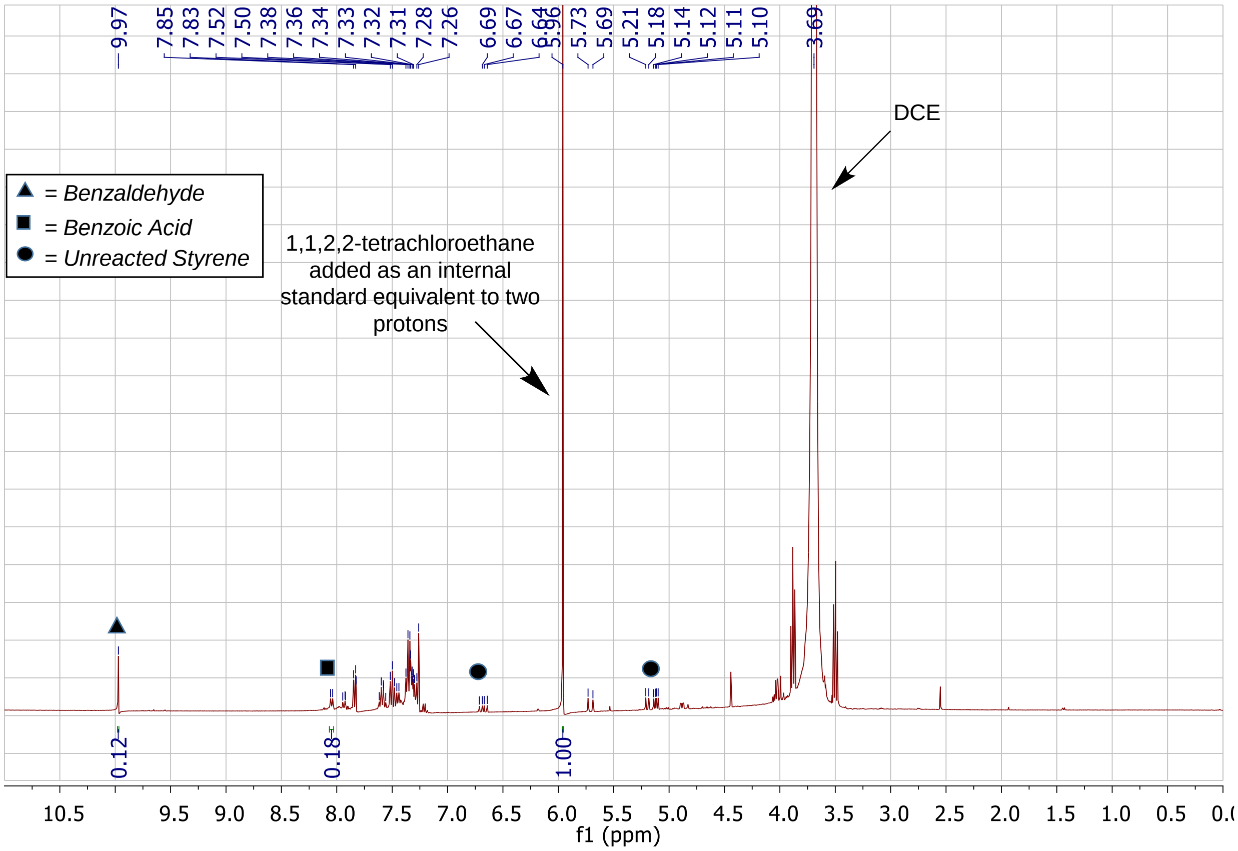

Figure S64: ^{1}H NMR spectrum of the styrene oxidation reaction in $CDCl_3$ using $Cs_3Sb_2Br_9$ as a photocatalyst in DCE as a solvent with a 50 W blue LED as the light source.

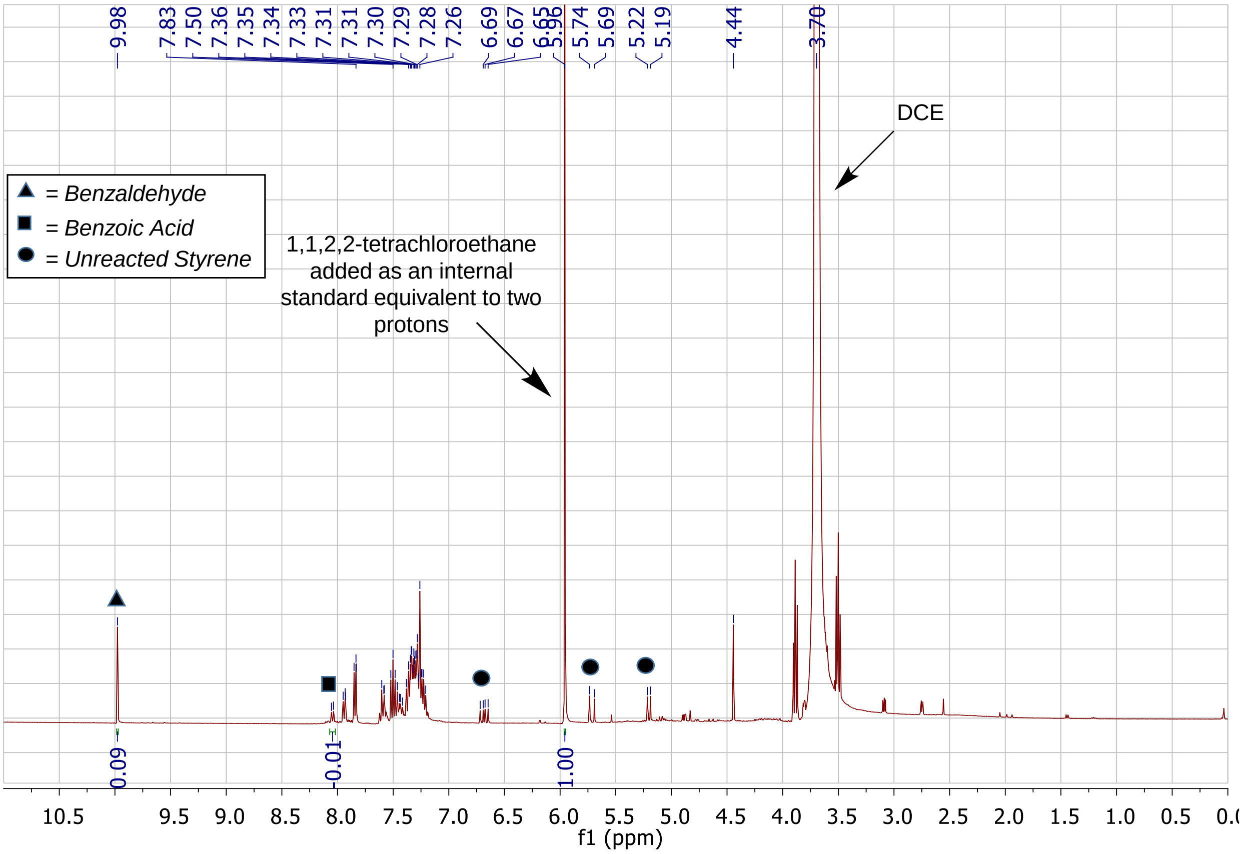

Figure S65: ^{1}H NMR spectrum of the styrene oxidation reaction in $CDCl_3$ using $Rb_3Bi_2Br_9$ as a photocatalyst in DCE as a solvent with a 50 W blue LED as the light source.

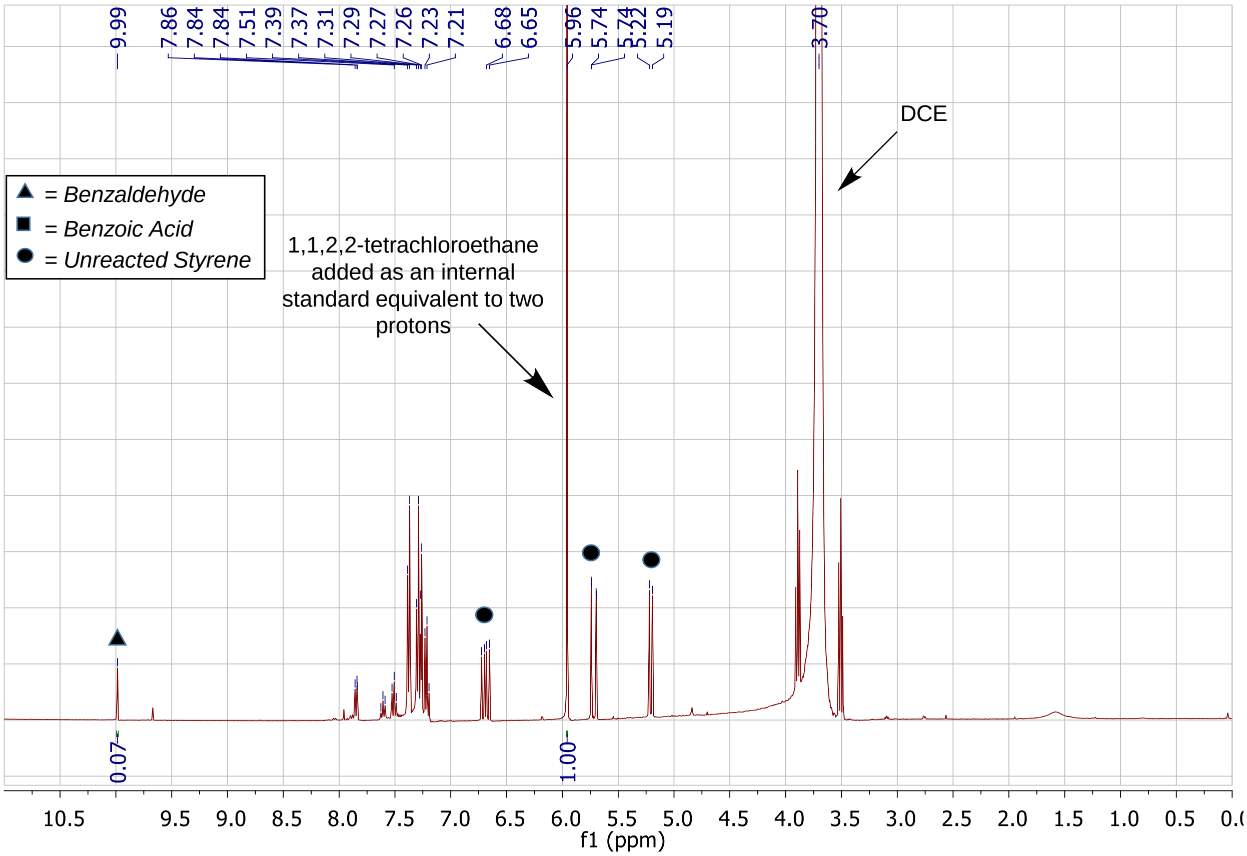

Figure S66: ^{1}H NMR spectrum of the styrene oxidation reaction in $CDCl_3$ using $Cs_3Bi_2Cl_9$ as a photocatalyst in DCE as a solvent with a 50 W blue LED as the light source.

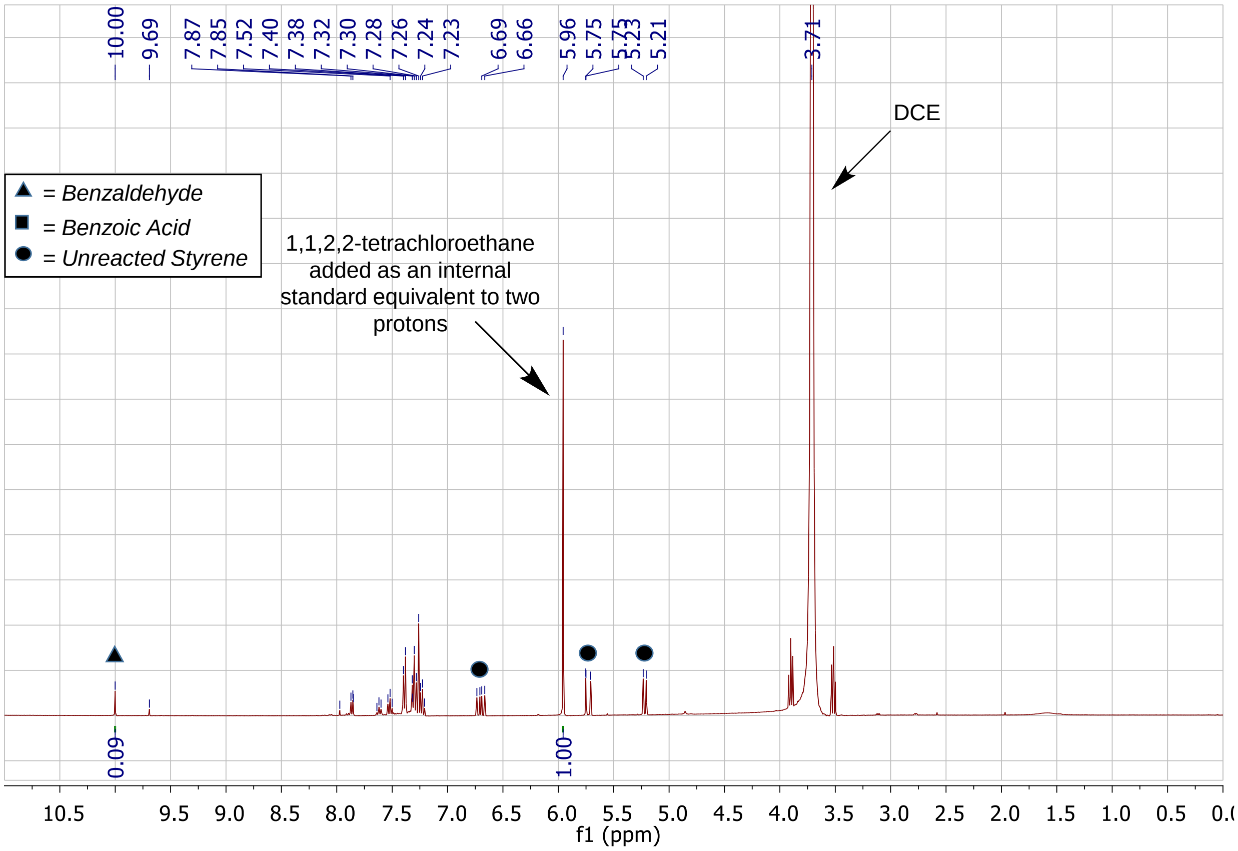

Figure S67: ^{1}H NMR spectrum of the styrene oxidation reaction in $CDCl_3$ using Cs_3BiCl_6 as a photocatalyst in DCE as a solvent with a 50 W blue LED as the light source.

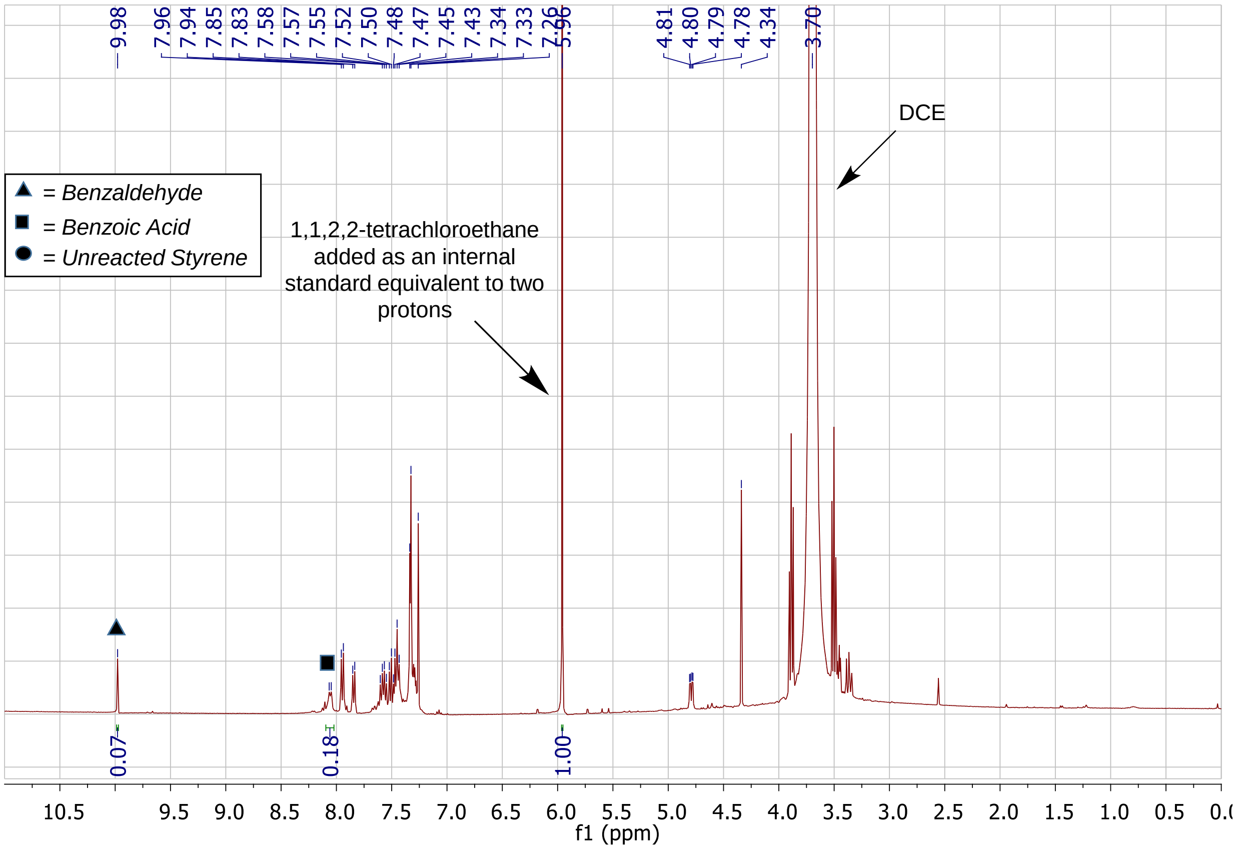

Figure S68: ^{1}H NMR spectrum of the styrene oxidation reaction in $CDCl_3$ using $Rb_3Sb_2I_9$ as a photocatalyst in DCE as a solvent with a 50 W blue LED as the light source.

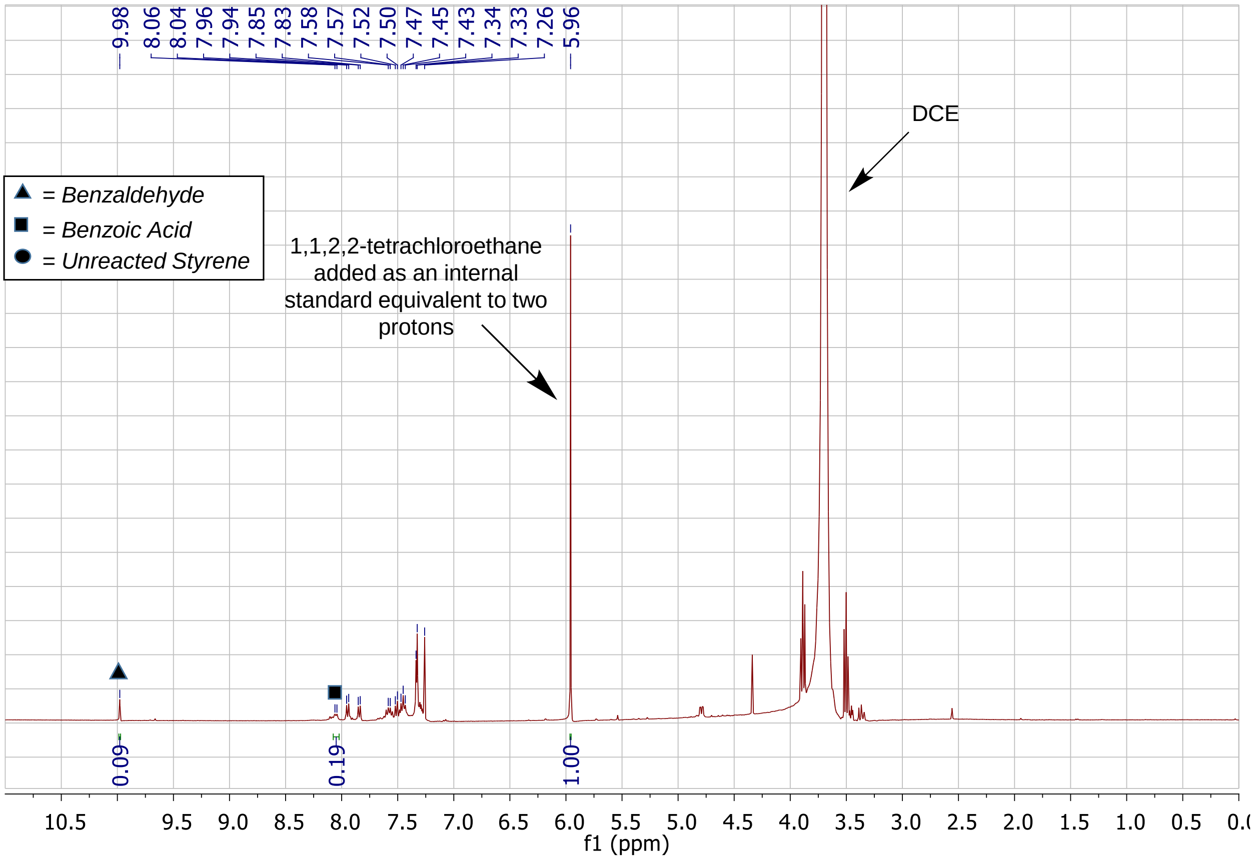

Figure S69: ^{1}H NMR spectrum of the styrene oxidation reaction in $CDCl_3$ using $Cs_3Sb_2I_9$ as a photocatalyst in DCE as a solvent with a 50 W blue LED as the light source.

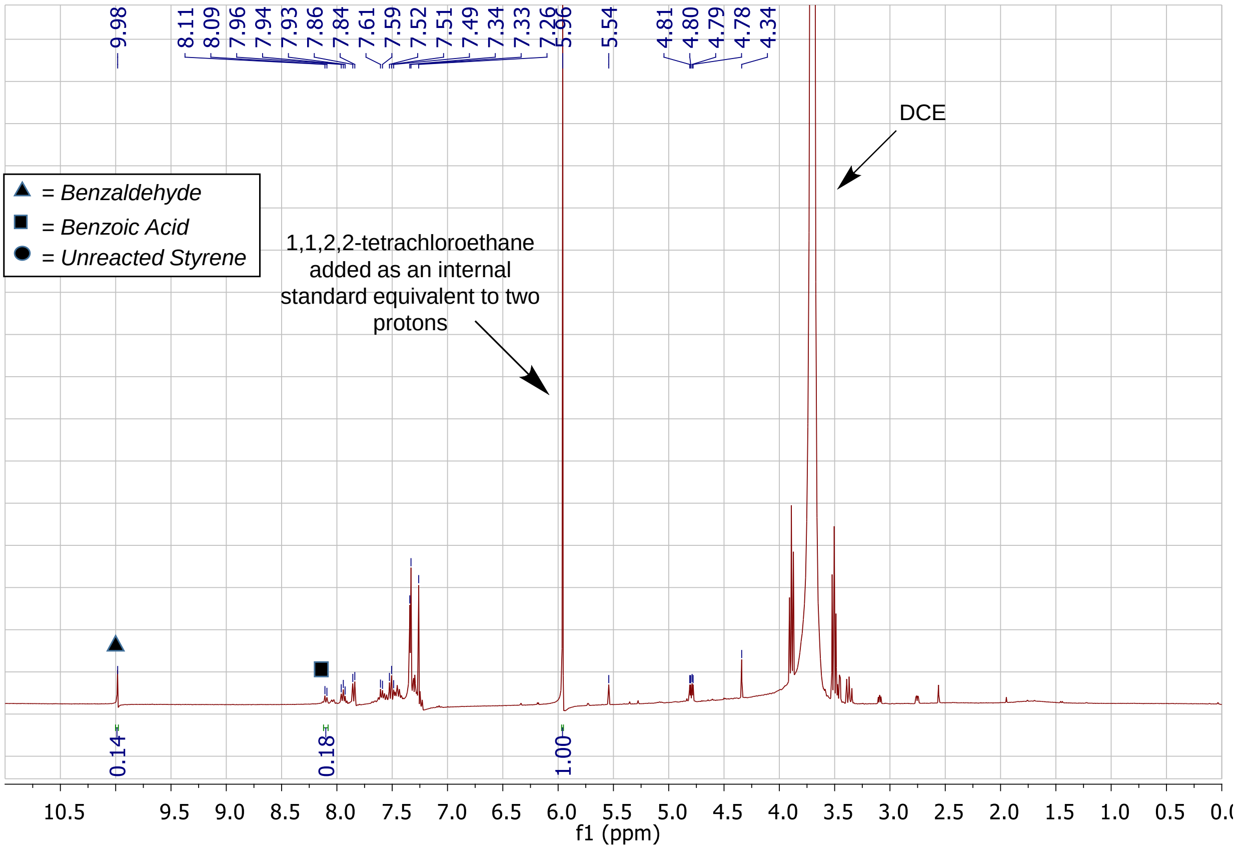

Figure S70: ^{1}H NMR spectrum of the styrene oxidation reaction in $CDCl_3$ using $Cs_3Bi_2I_9$ as a photocatalyst in DCE as a solvent with a 50 W blue LED as the light source.

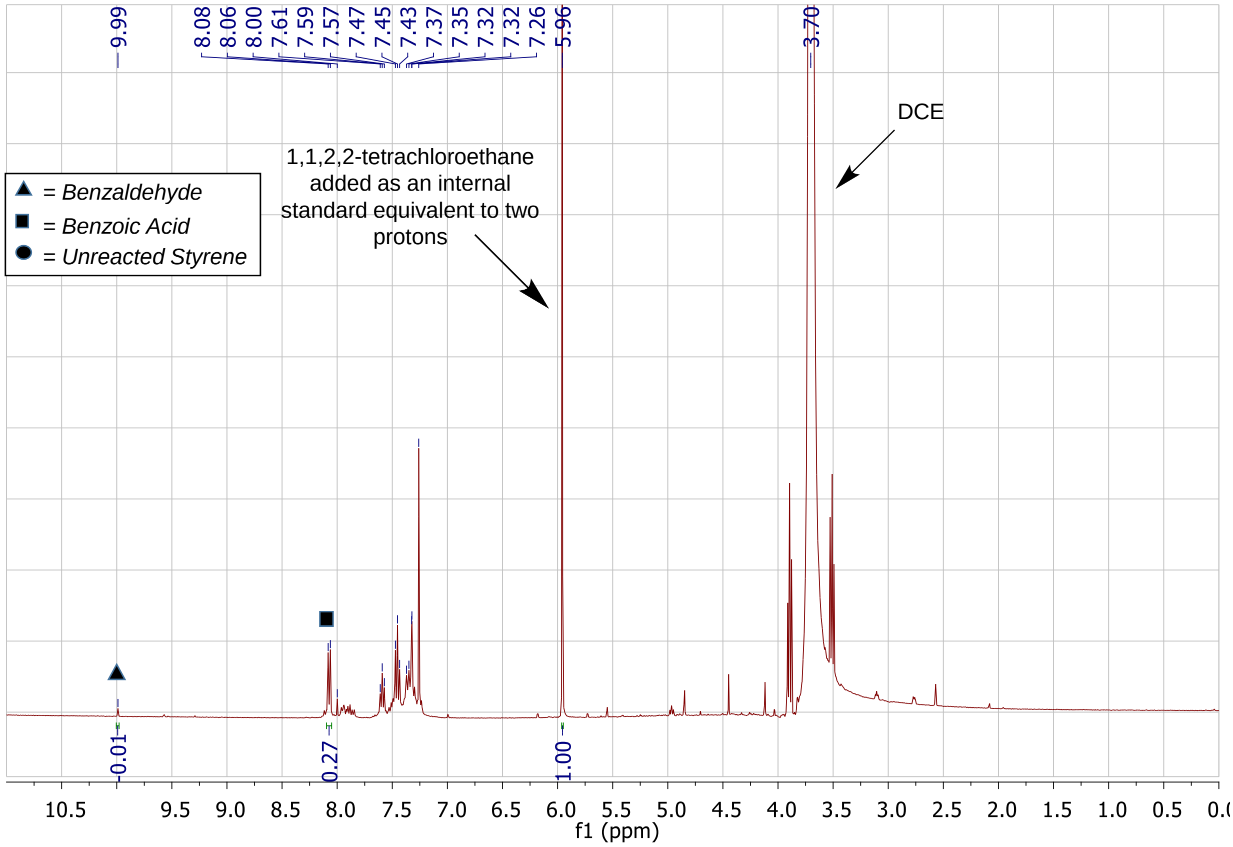

Figure S71: ^{1}H NMR spectrum of the styrene oxidation reaction in $CDCl_3$ using $Cs_2AgBiCl_4Br_2$ as a photocatalyst in DCE as a solvent with a 50 W blue LED as the light source after 72 h.

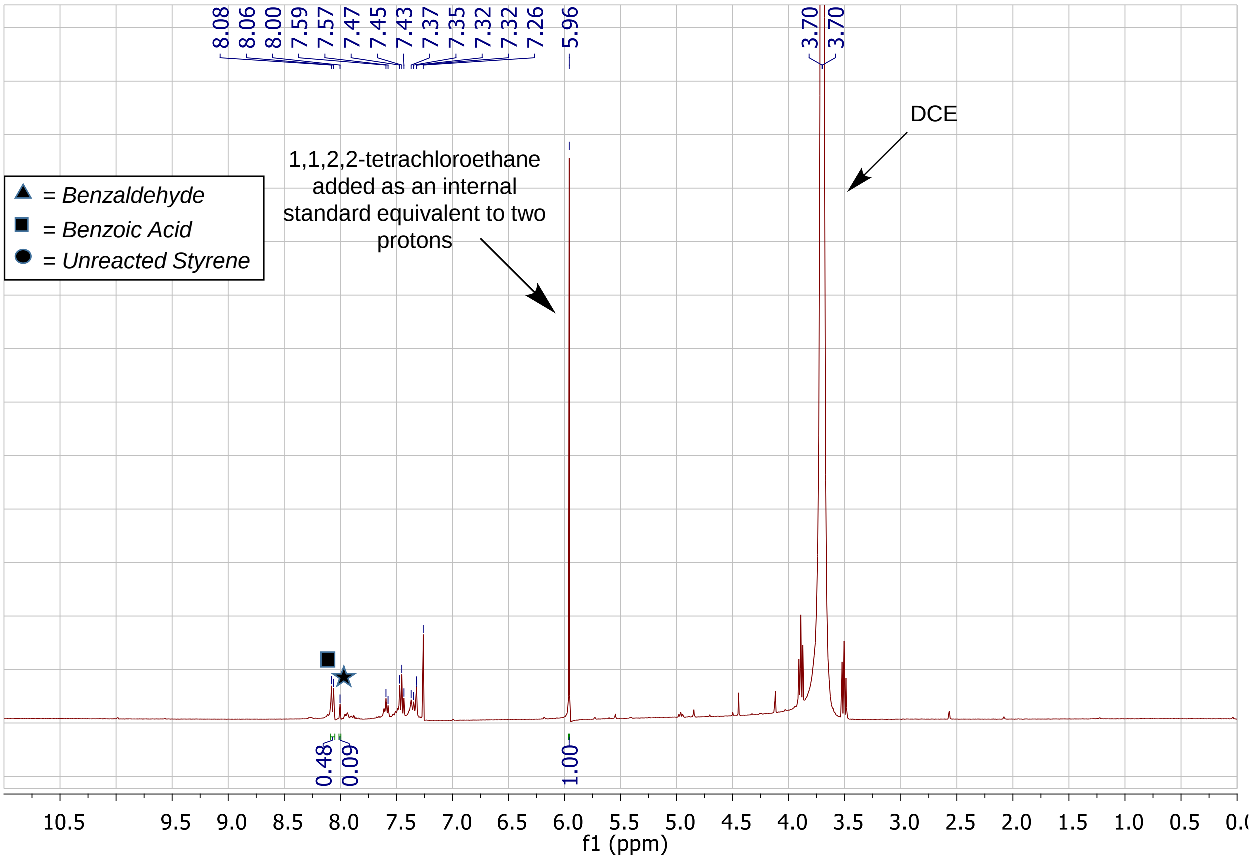

Figure S72: ^{1}H NMR spectrum of the styrene oxidation reaction in $CDCl_3$ using $Cs_2AgBiCl_4Br_2$ as a photocatalyst in DCE as a solvent with a 50 W blue LED as the light source after 96 h.

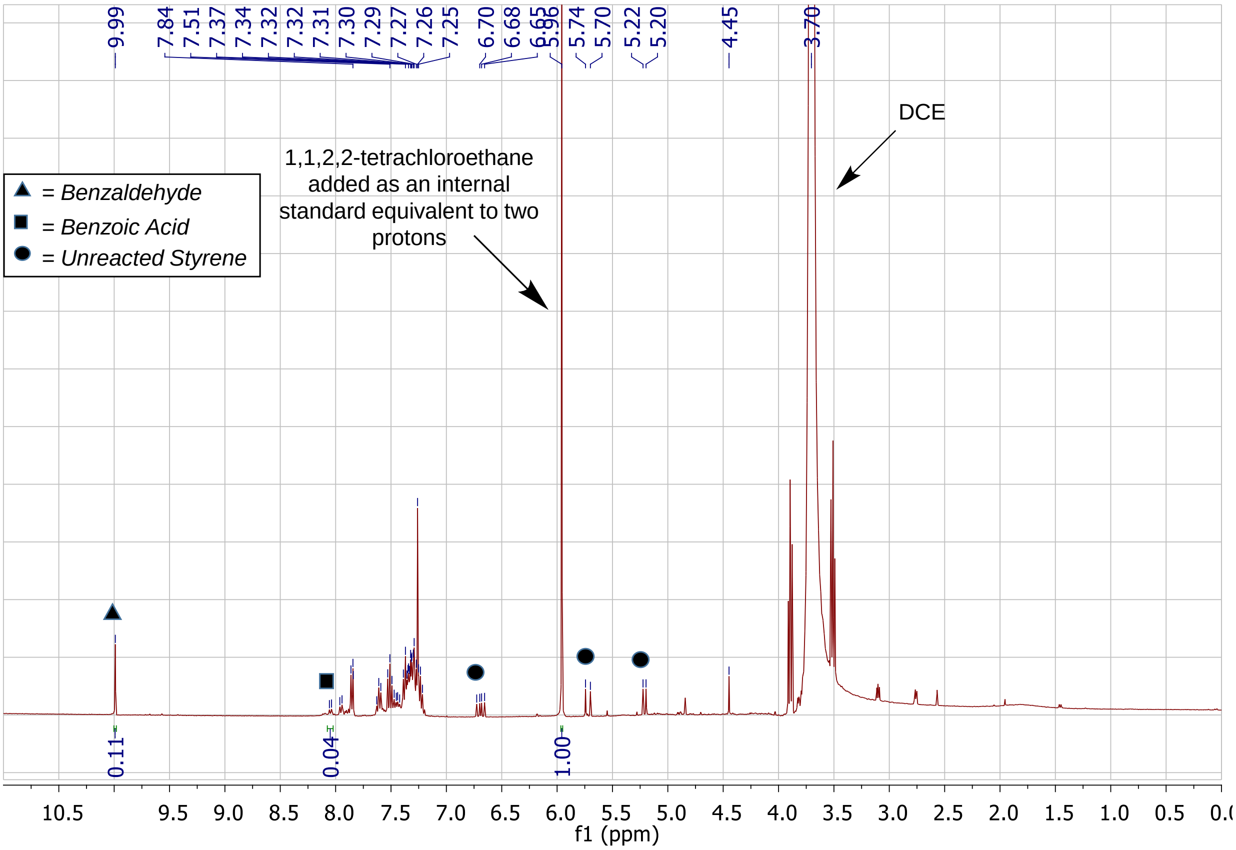

Figure S73: ^{1}H NMR spectrum of the styrene oxidation reaction in $CDCl_3$ using $Cs_2AgBiCl_2Br_4$ as a photocatalyst in DCE as a solvent with a 50 W blue LED as the light source.

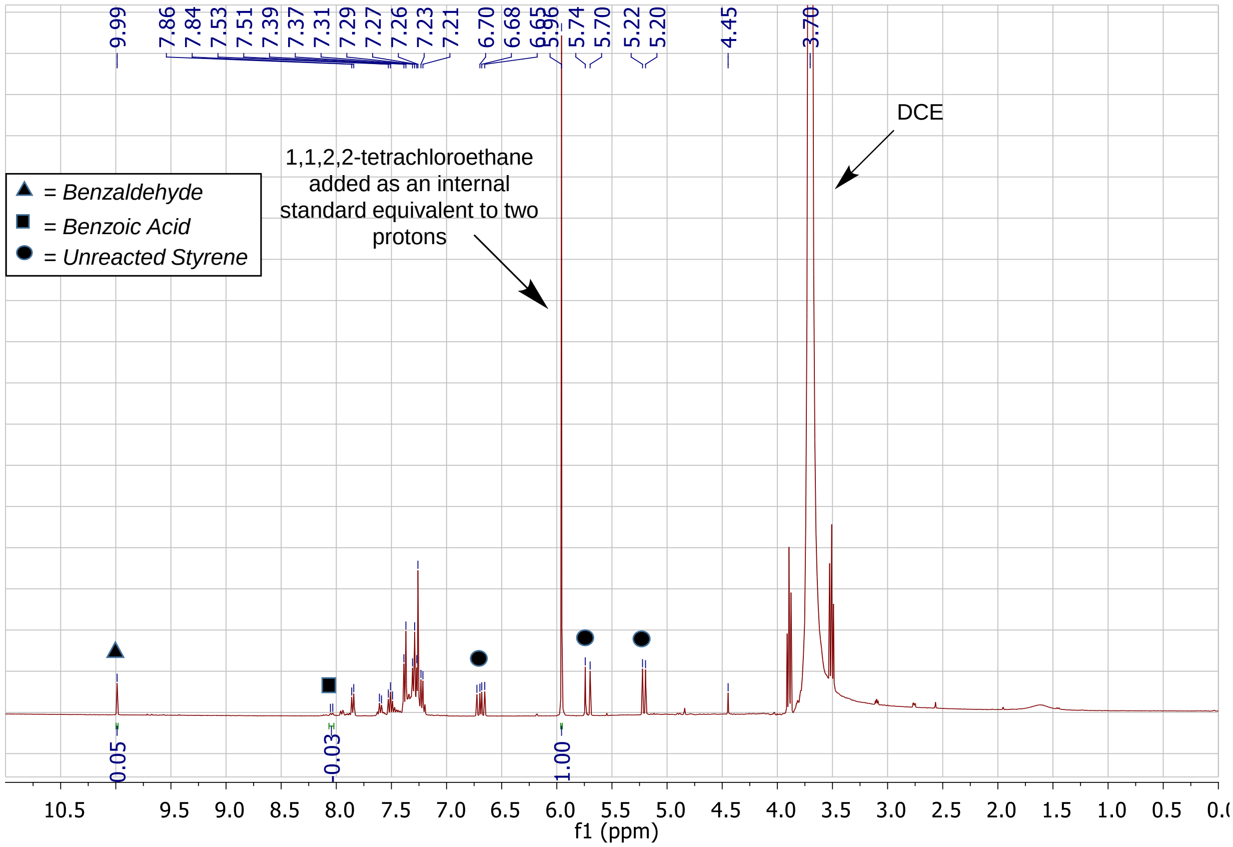

Figure S74: ^{1}H NMR spectrum of the styrene oxidation reaction in $CDCl_3$ using $Cs_2AgBiCl_5Br$ as a photocatalyst in DCE as a solvent with a 50 W blue LED as the light source.

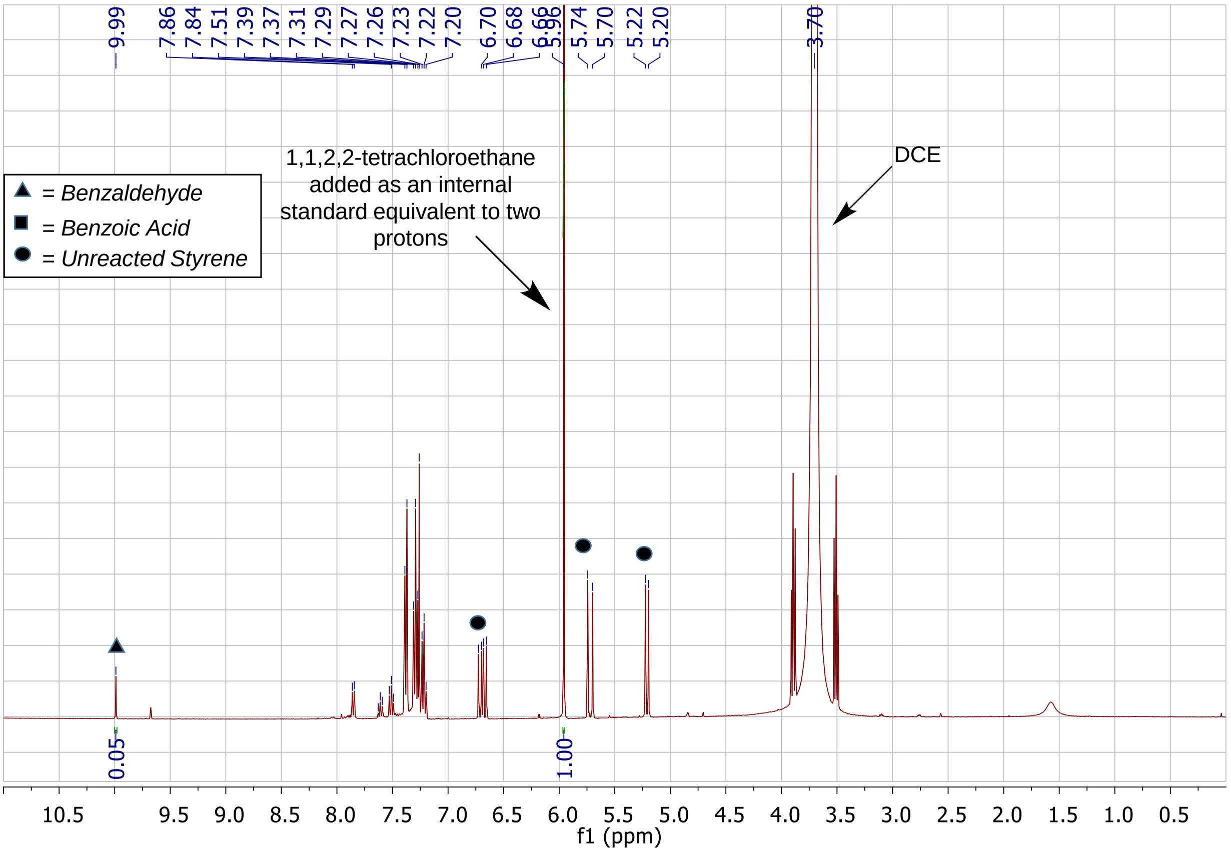

Figure S75: ^{1}H NMR spectrum of the styrene oxidation reaction in $CDCl_3$ using $Cs_3Bi_2Br_9$ as a photocatalyst in DCE as a solvent with a 50 W blue LED as the light source.

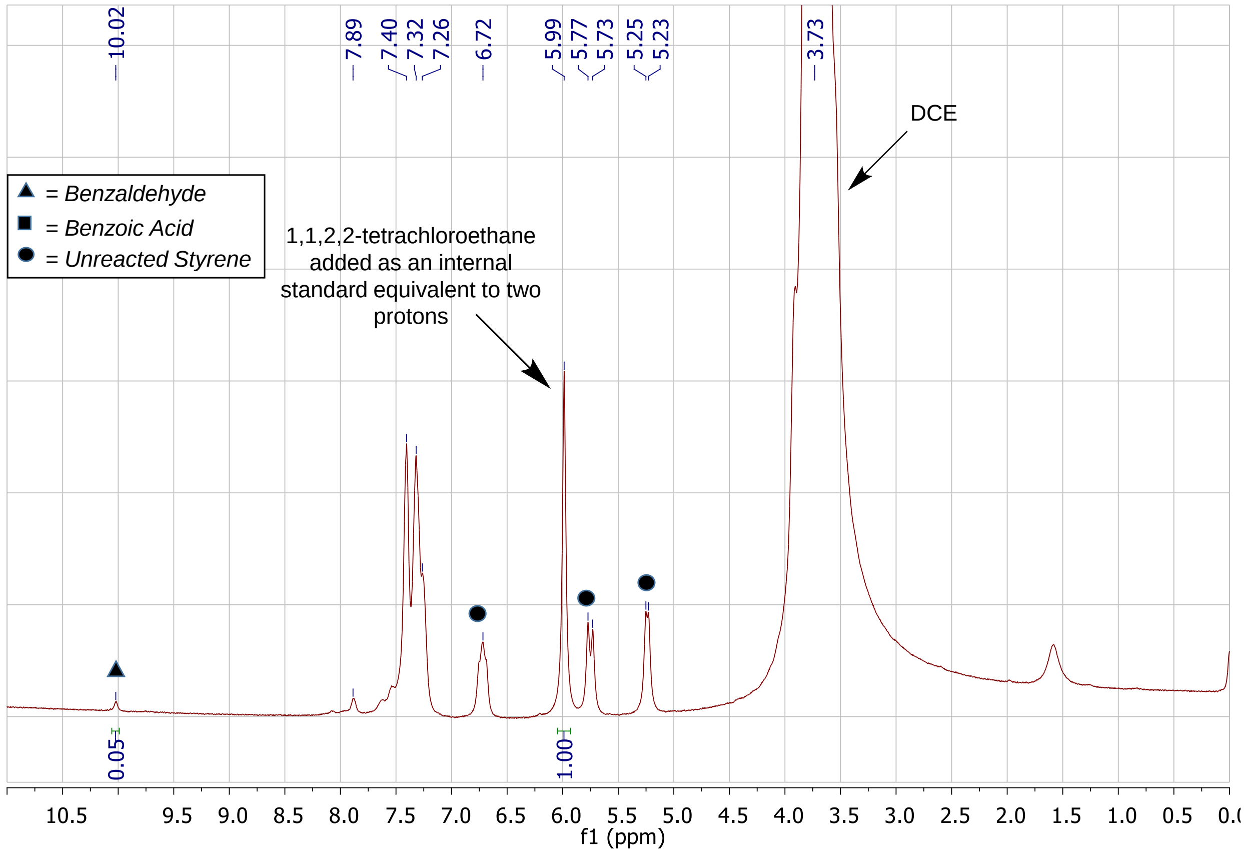

Figure S76: ^{1}H NMR spectrum of the styrene oxidation reaction in $CDCl_3$ using $Cs_4Cu_{0.5}Mn_{0.5}Sb_2Cl_{12}$ as a photocatalyst in DCE as a solvent with a 50 W blue LED as the light source.

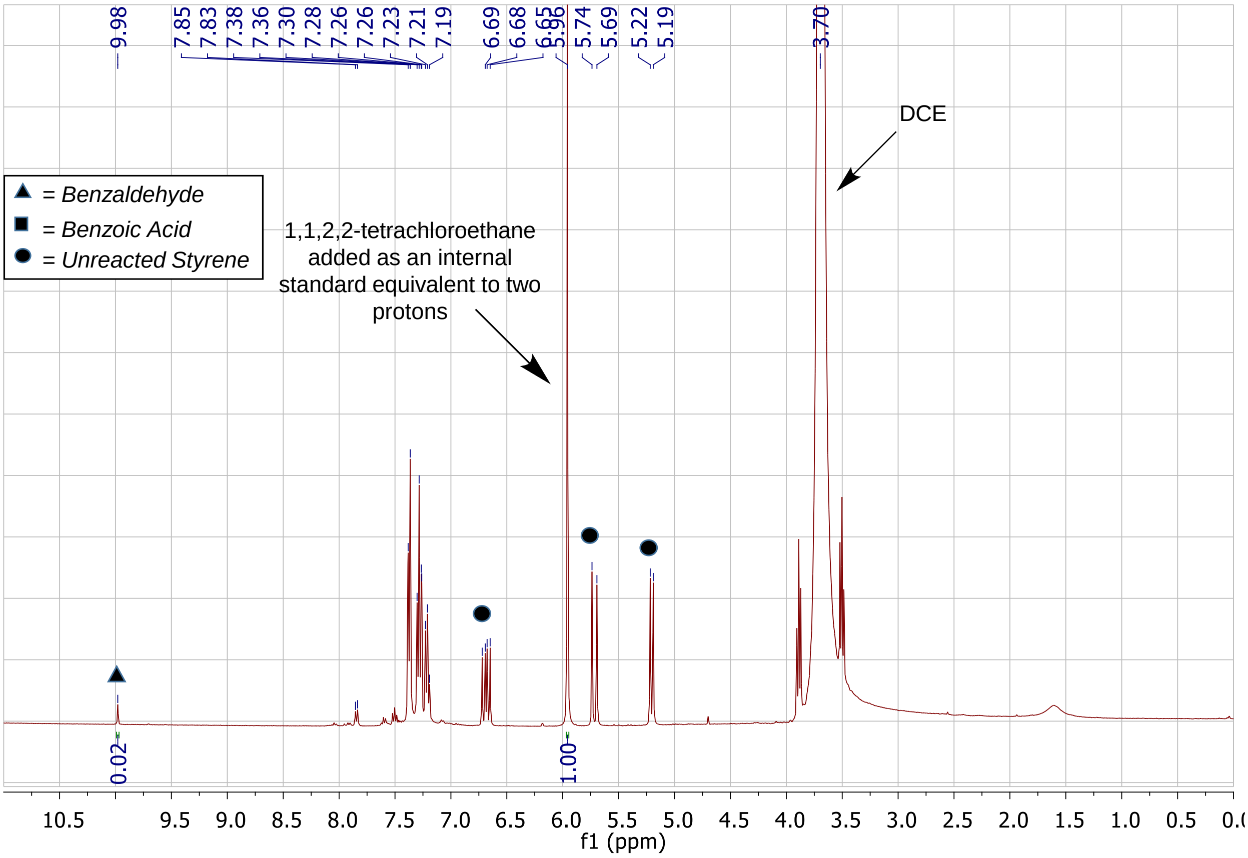

Figure S77: ^{1}H NMR spectrum of the styrene oxidation reaction in $CDCl_3$ using $Cs_4Cu_{0.4}Mn_{0.6}Sb_2Cl_{12}$ as a photocatalyst in DCE as a solvent with a 50 W blue LED as the light source.

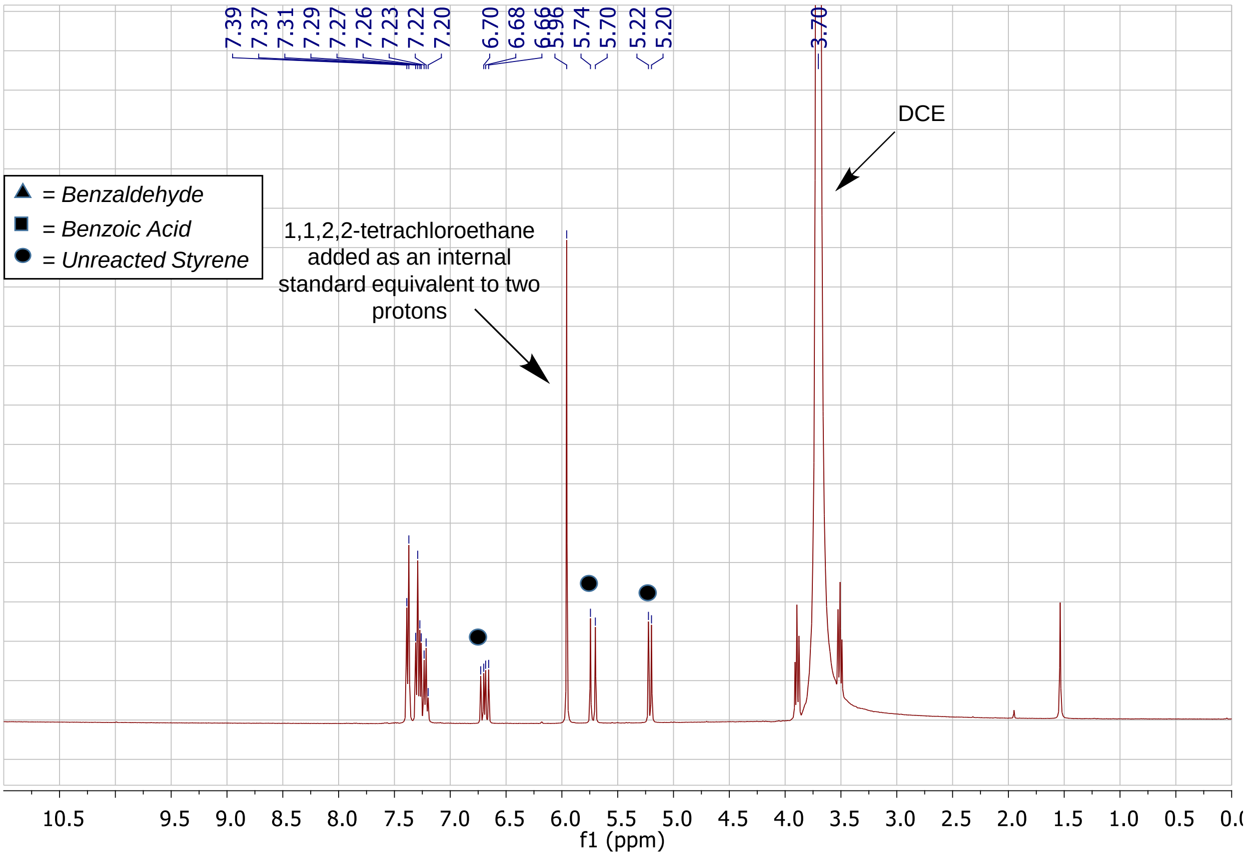

Figure S78: ^{1}H NMR spectrum of the styrene oxidation reaction in $CDCl_3$ using $Cs_4Cu_{0.8}Mn_{0.2}Sb_2Cl_{12}$ as a photocatalyst in DCE as a solvent with a 50 W blue LED as the light source.

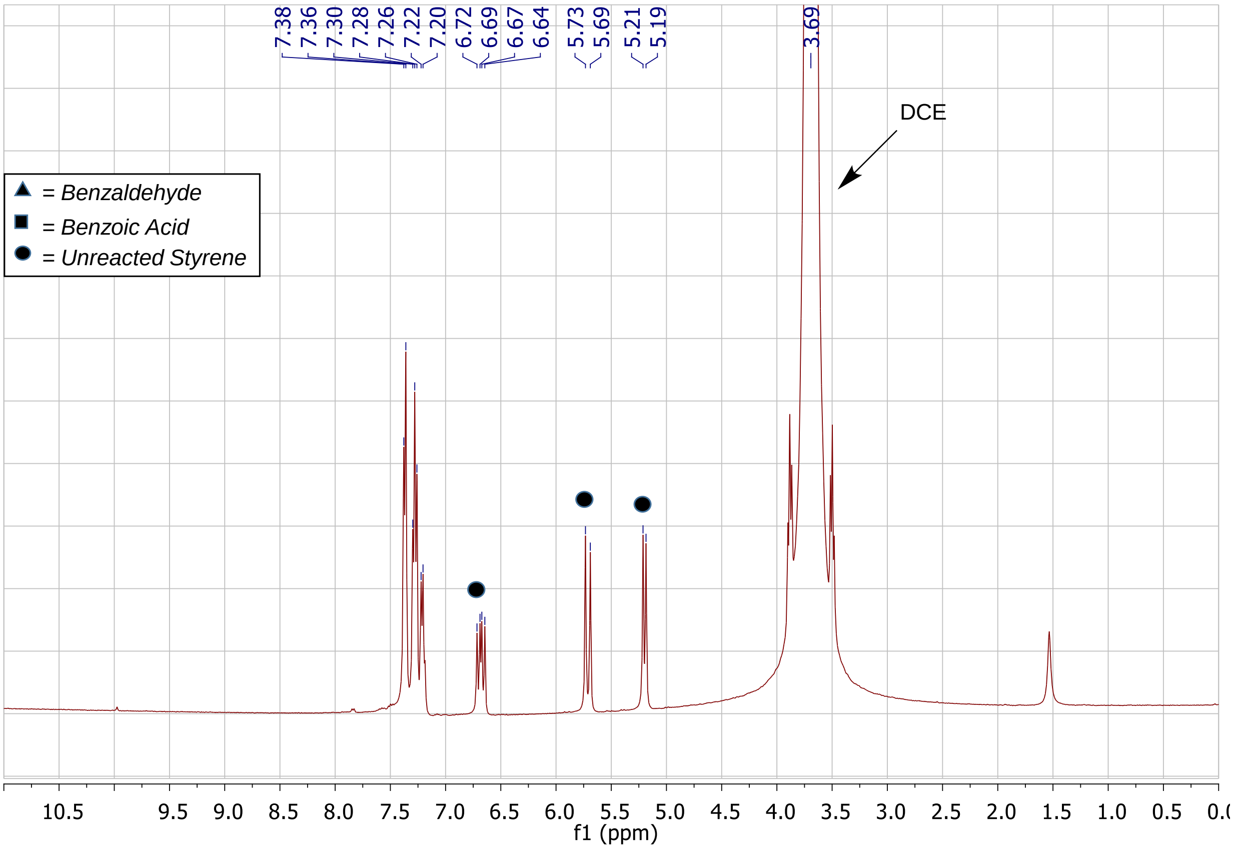

Figure S79: ^{1}H NMR spectrum of the styrene oxidation reaction in $CDCl_3$ using $Cs_4Cu_{0.2}Mn_{0.8}Sb_2Cl_{12}$ as a photocatalyst in DCE as a solvent with a 50 W blue LED as the light source.

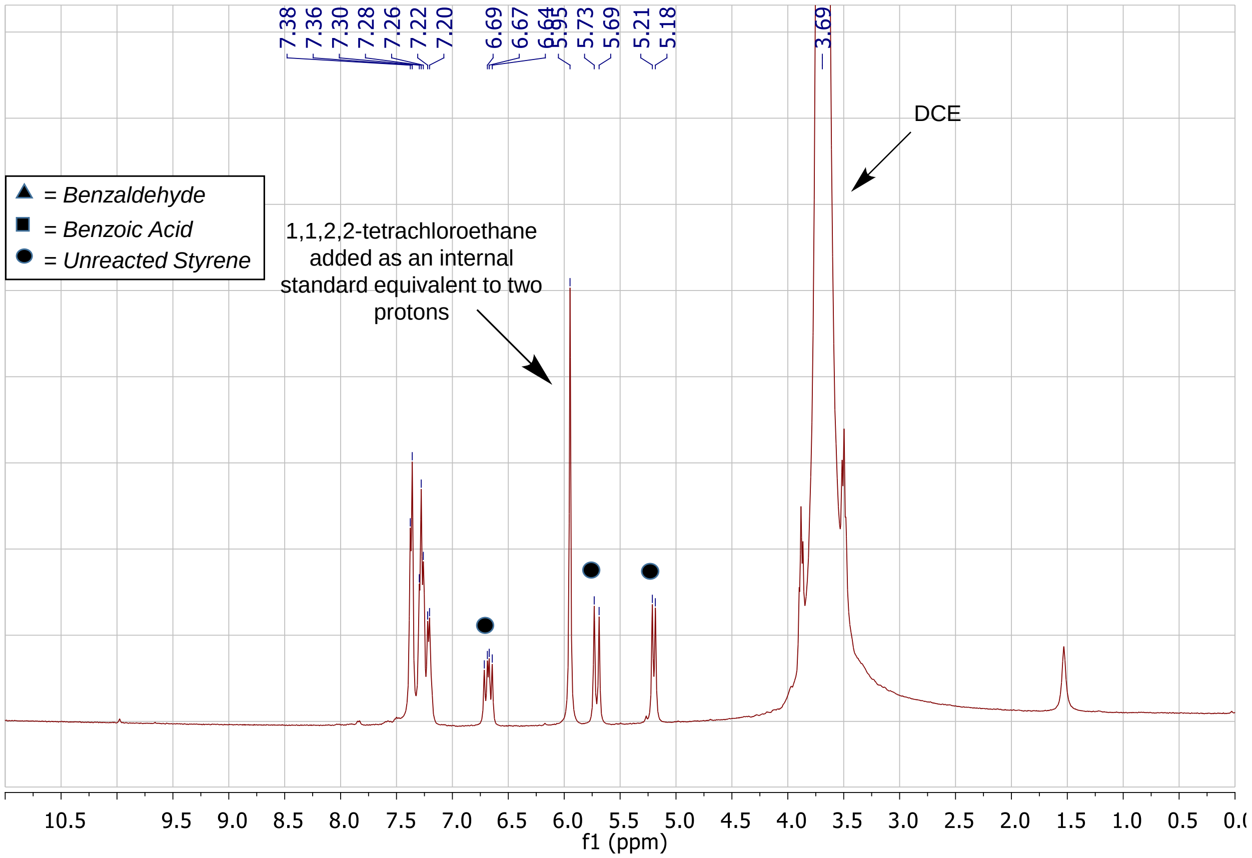

Figure S80: ^{1}H NMR spectrum of the styrene oxidation reaction in $CDCl_3$ using $Cs_4Cu_{0.6}Mn_{0.4}Sb_2Cl_{12}$ as a photocatalyst in DCE as a solvent with a 50 W blue LED as the light source.

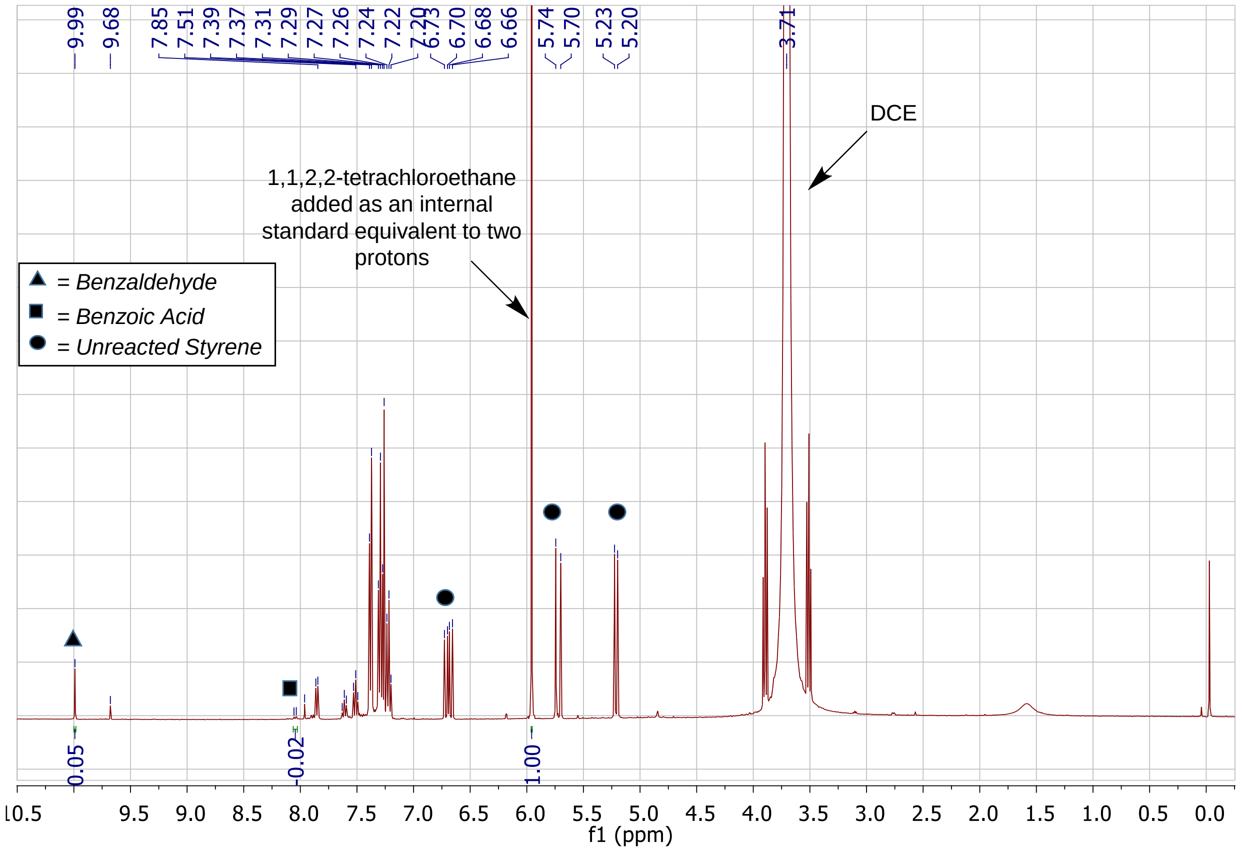

Figure S81: ^{1}H NMR spectrum of the styrene oxidation reaction in $CDCl_3$ using $Cs_2NaInCl_6$ as a photocatalyst in DCE as a solvent with a 50 W blue LED as the light source.

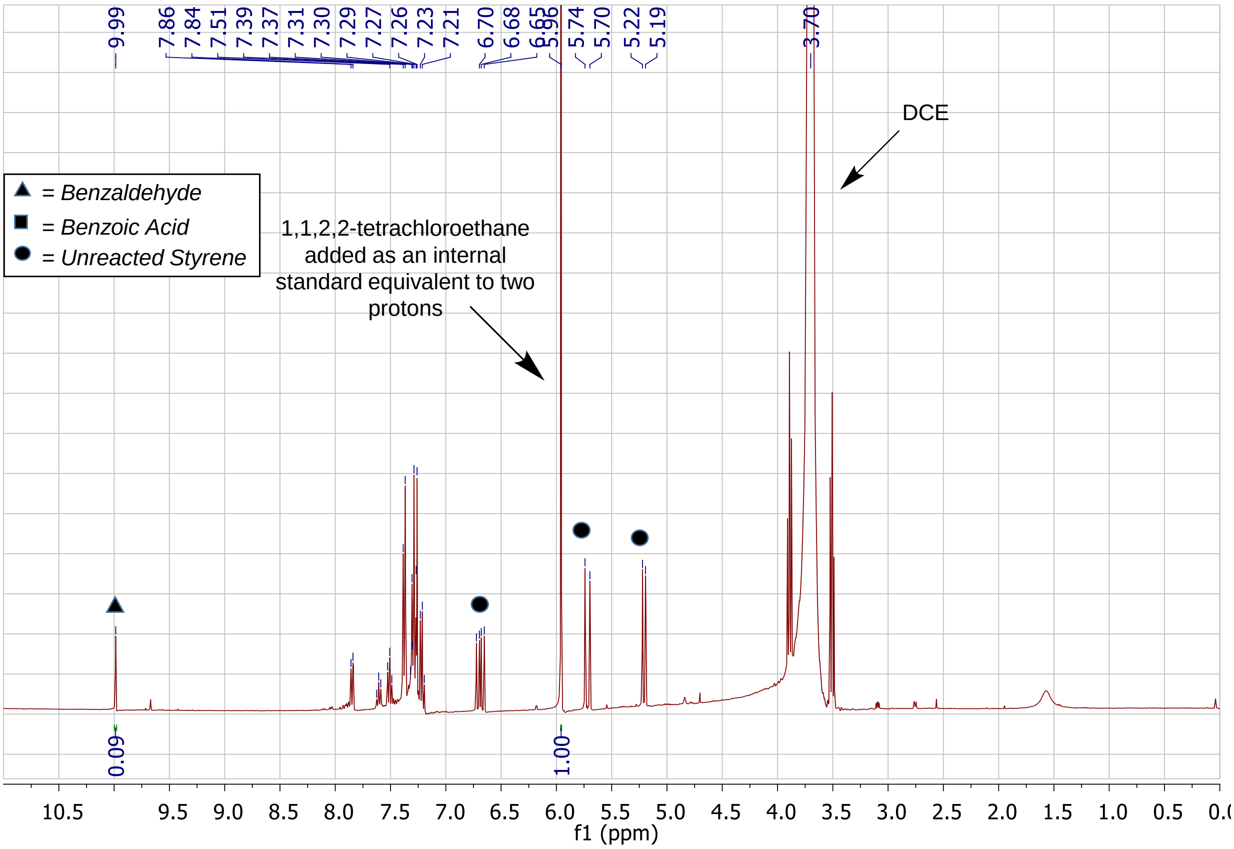

Figure S82: ^{1}H NMR spectrum of the styrene oxidation reaction in $CDCl_3$ using $Cs_2AgInCl_6$ as a photocatalyst in DCE as a solvent with a 50 W blue LED as the light source.

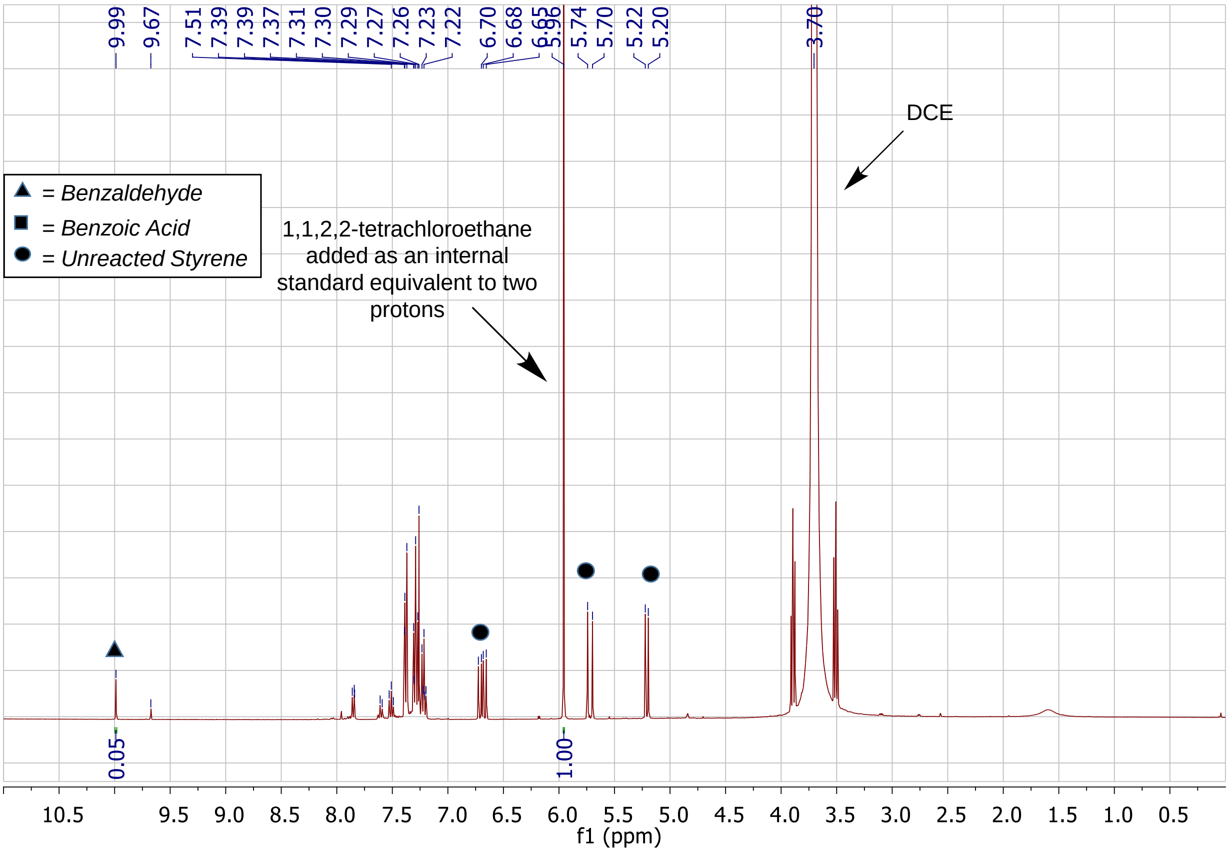

Figure S83: ^{1}H NMR spectrum of the styrene oxidation reaction in $CDCl_3$ using $Cs_2Ag_{0.2}Na_{0.8}InCl_6$ as a photocatalyst in DCE as a solvent with a 50 W blue LED as the light source.

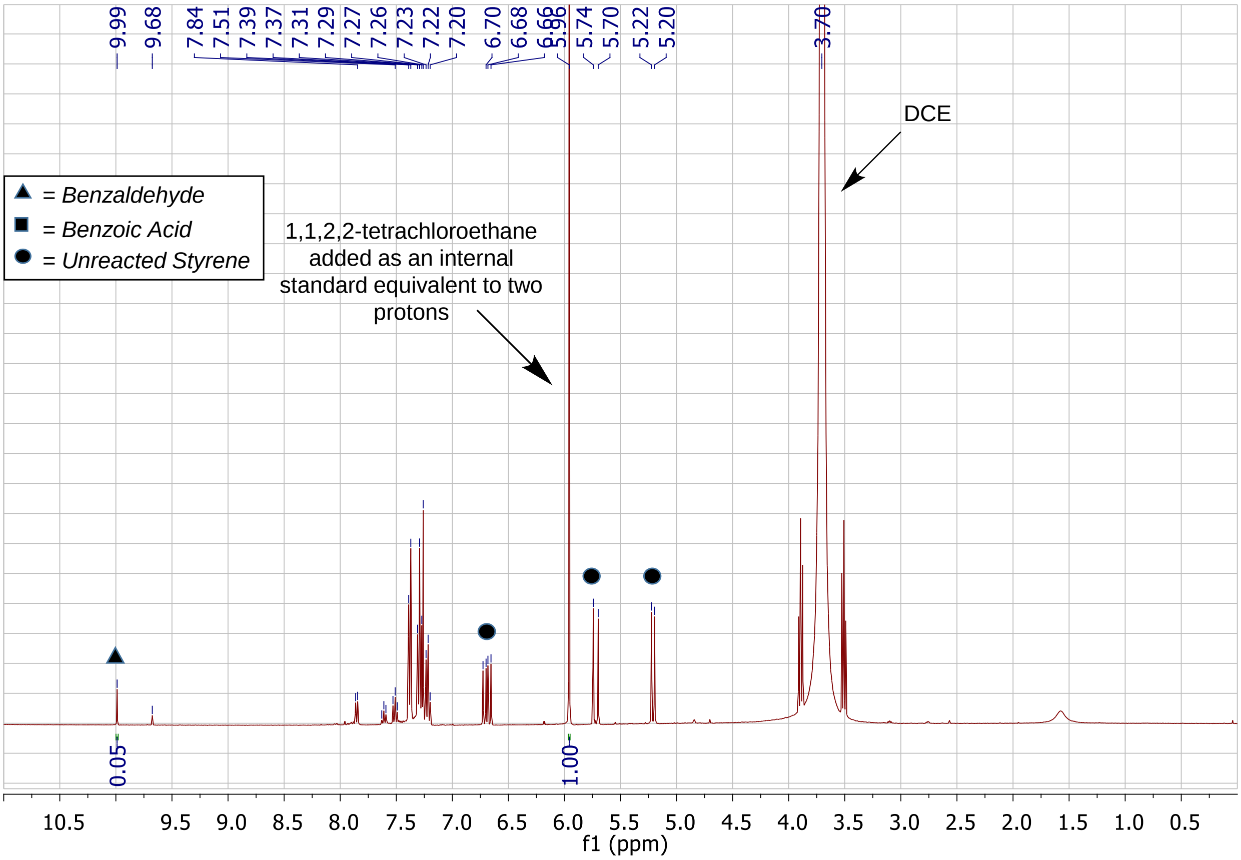

Figure S84: ^{1}H NMR spectrum of the styrene oxidation reaction in $CDCl_3$ using $Cs_2Ag_{0.8}Na_{0.2}InCl_6$ as a photocatalyst in DCE as a solvent with a 50 W blue LED as the light source.

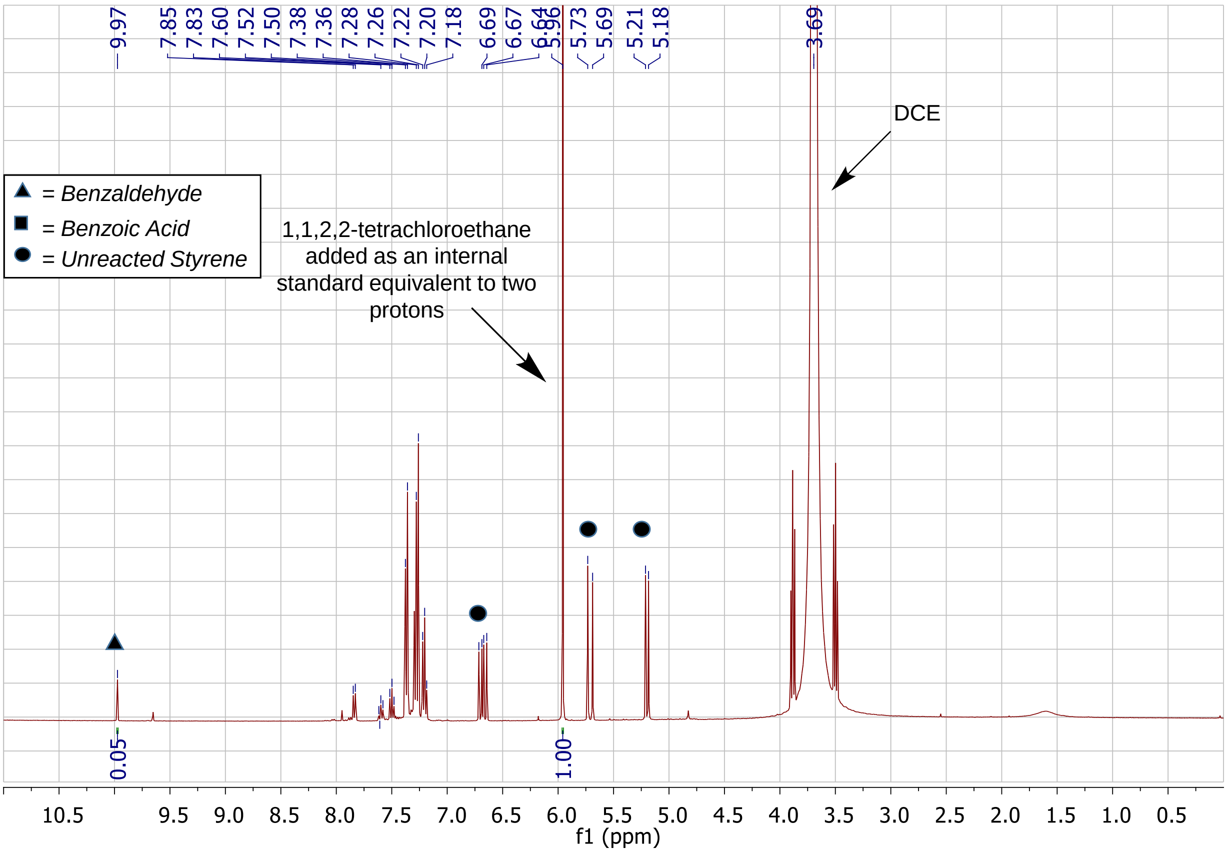

Figure S85: ^{1}H NMR spectrum of the styrene oxidation reaction in $CDCl_3$ using $Cs_2Ag_{0.6}Na_{0.4}InCl_6$ as a photocatalyst in DCE as a solvent with a 50 W blue LED as the light source.

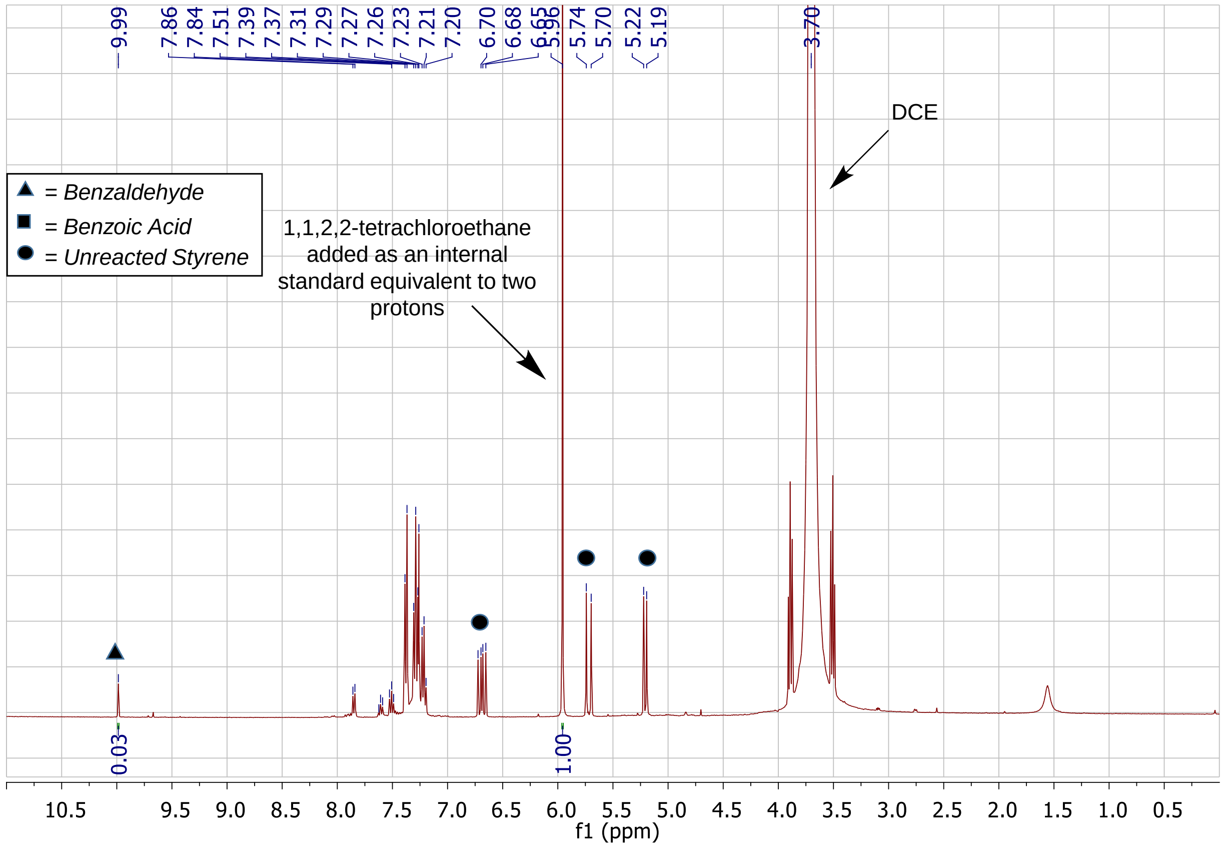

Figure S86: ^{1}H NMR spectrum of the styrene oxidation reaction in $CDCl_3$ using $Cs_2Ag_{0.4}Na_{0.6}InCl_6$ as a photocatalyst in DCE as a solvent with a 50 W blue LED as the light source.

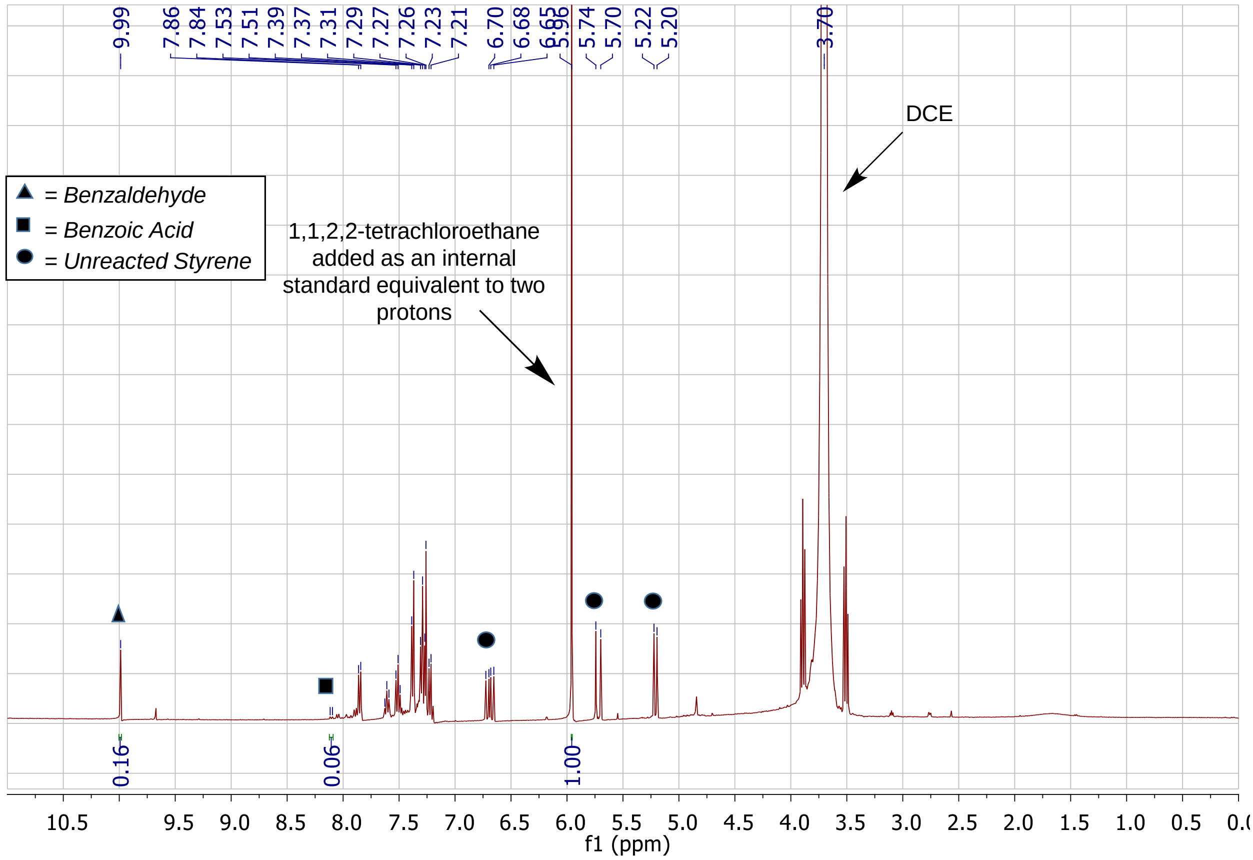

Figure S87: ^{1}H NMR spectrum of the styrene oxidation reaction in $CDCl_3$ using $Cs_2NaBiCl_6$ as a photocatalyst in DCE as a solvent with a 50 W blue LED as the light source.

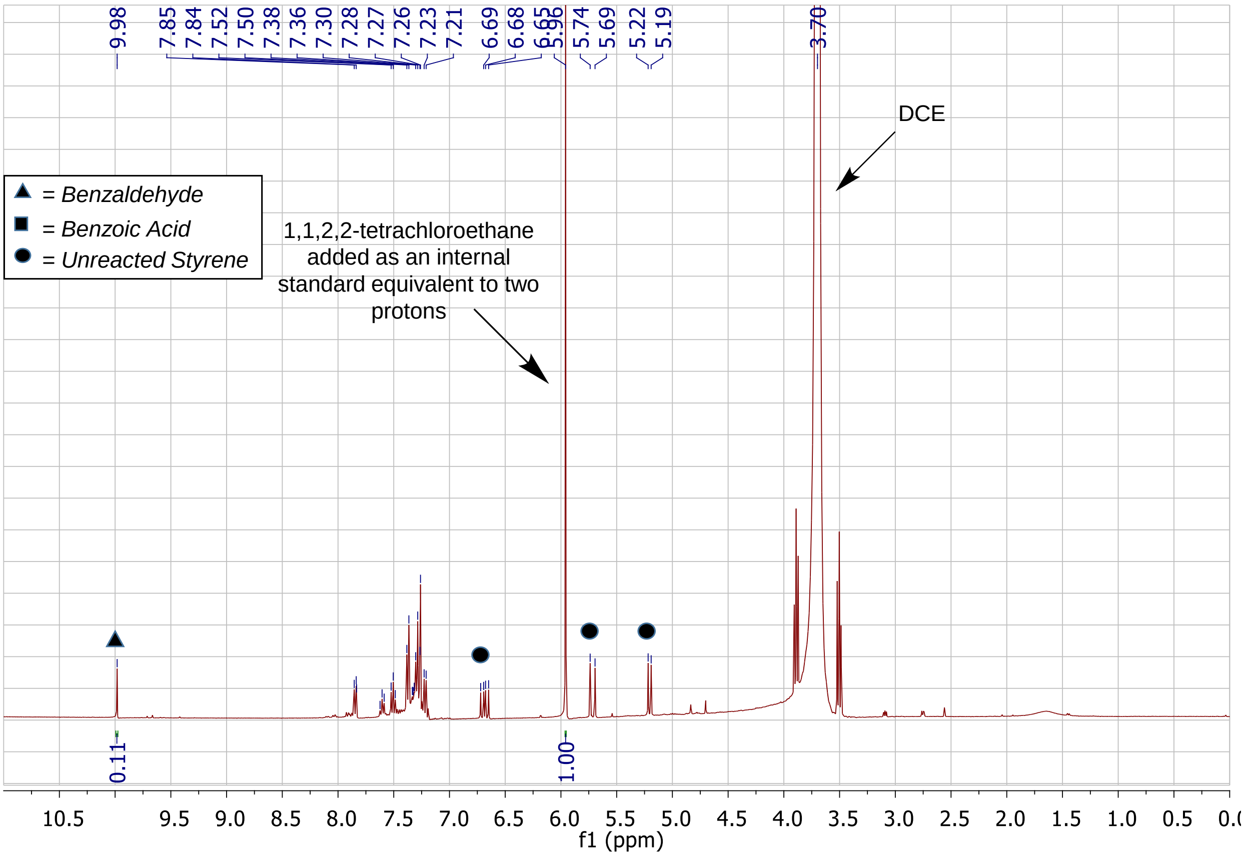

Figure S88: ^{1}H NMR spectrum of the styrene oxidation reaction in $CDCl_3$ using $Cs_2Ag_{0.5}Na_{0.5}BiCl_6$ as a photocatalyst in DCE as a solvent with a 50 W blue LED as the light source.

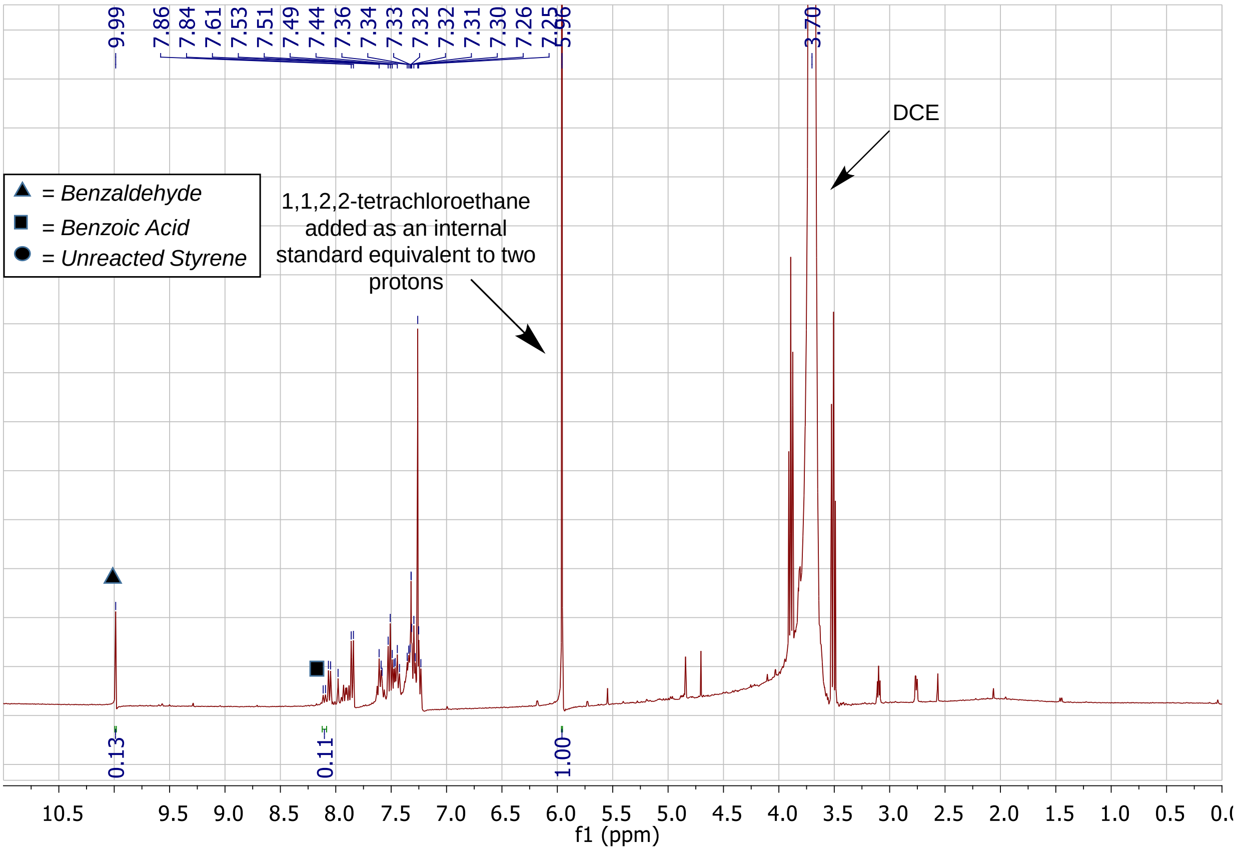

Figure S89: ^{1}H NMR spectrum of the styrene oxidation reaction in $CDCl_3$ using $Cs_2Ag_{0.9}Na_{0.1}BiCl_6$ as a photocatalyst in DCE as a solvent with a 50 W blue LED as the light source.

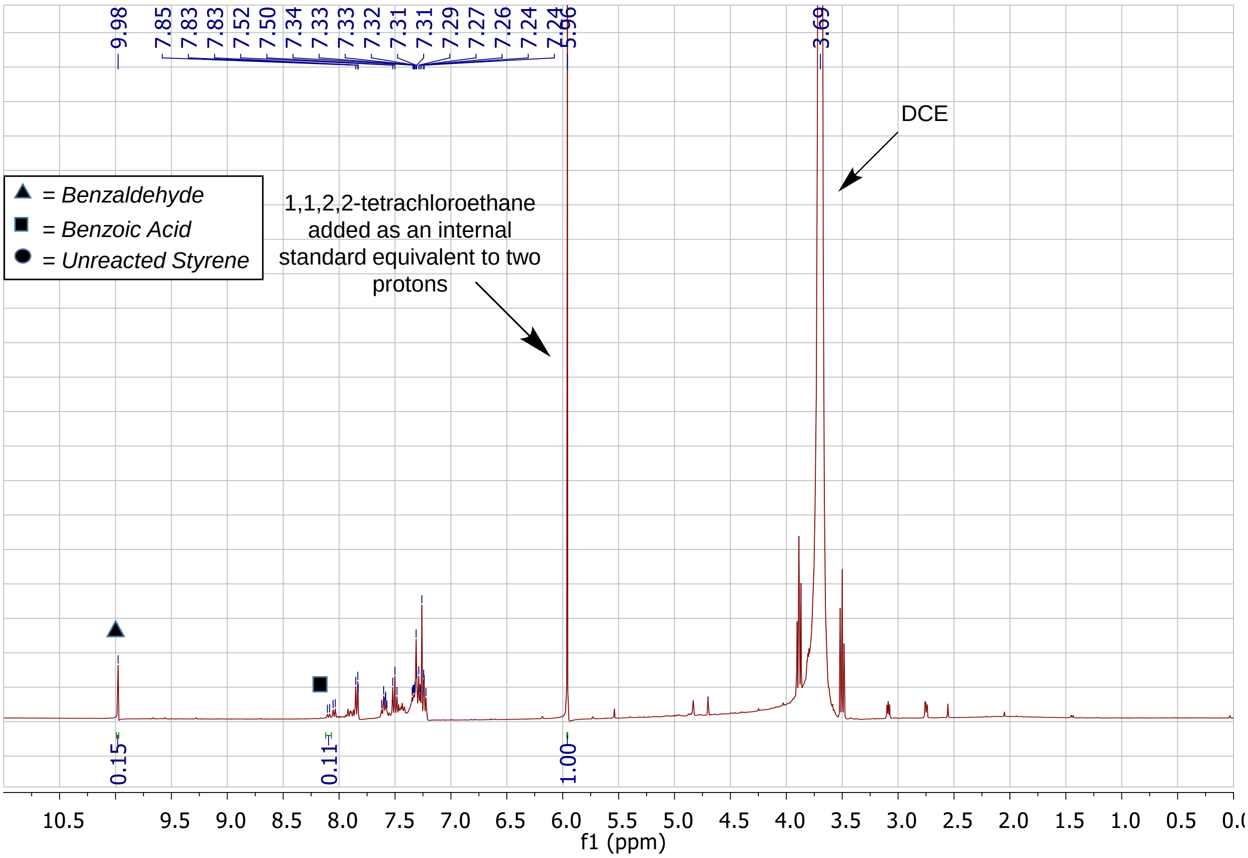

Figure S90: ^{1}H NMR spectrum of the styrene oxidation reaction in $CDCl_3$ using $Cs_2Ag_{0.8}Na_{0.2}BiCl_6$ as a photocatalyst in DCE as a solvent with a 50 W blue LED as the light source.

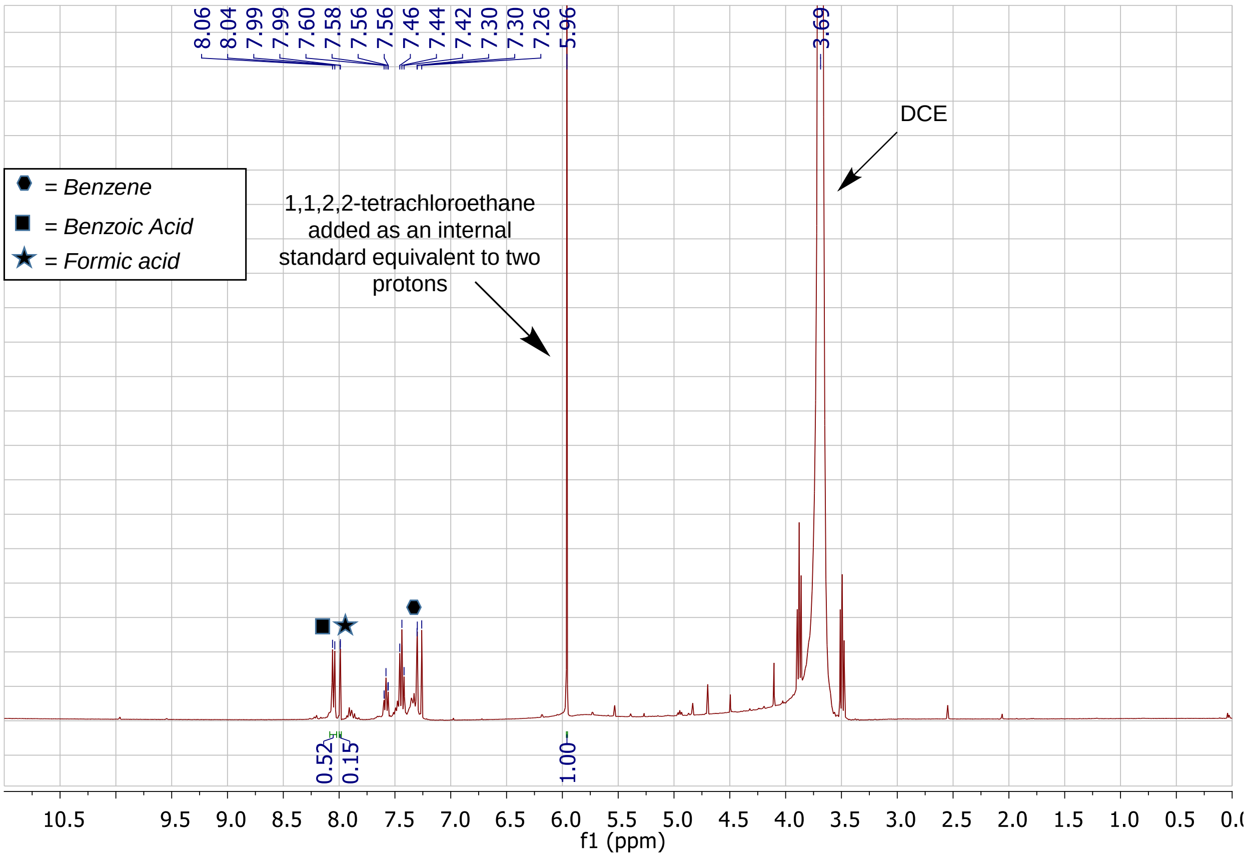

Figure S91: ^{1}H NMR spectrum of the styrene oxidation reaction in $CDCl_3$ using $Cs_2Ag_{0.95}Na_{0.05}BiCl_6$ as a photocatalyst in DCE as a solvent with a 50 W blue LED as the light source.

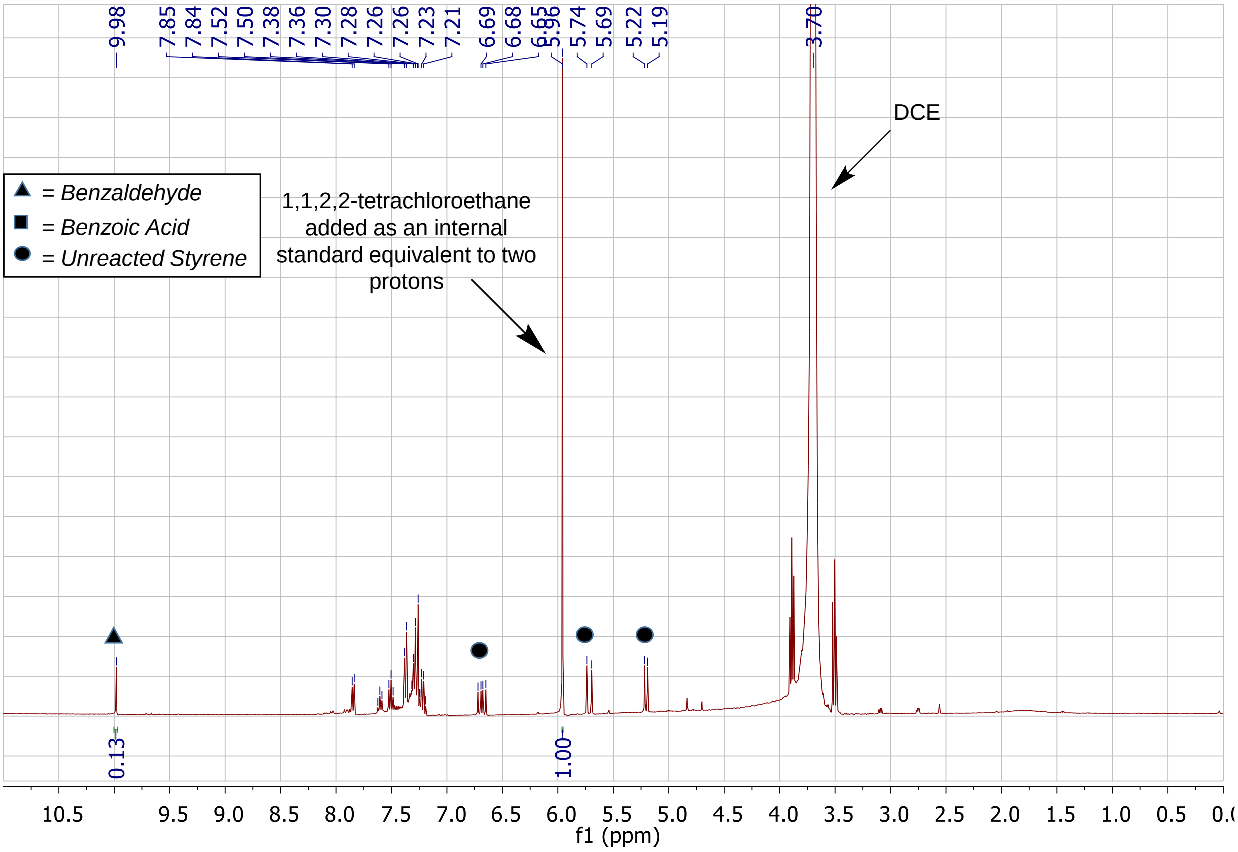

Figure S92: ^{1}H NMR spectrum of the styrene oxidation reaction in $CDCl_3$ using $Cs_2Ag_{0.6}Na_{0.4}BiCl_6$ as a photocatalyst in DCE as a solvent with a 50 W blue LED as the light source.

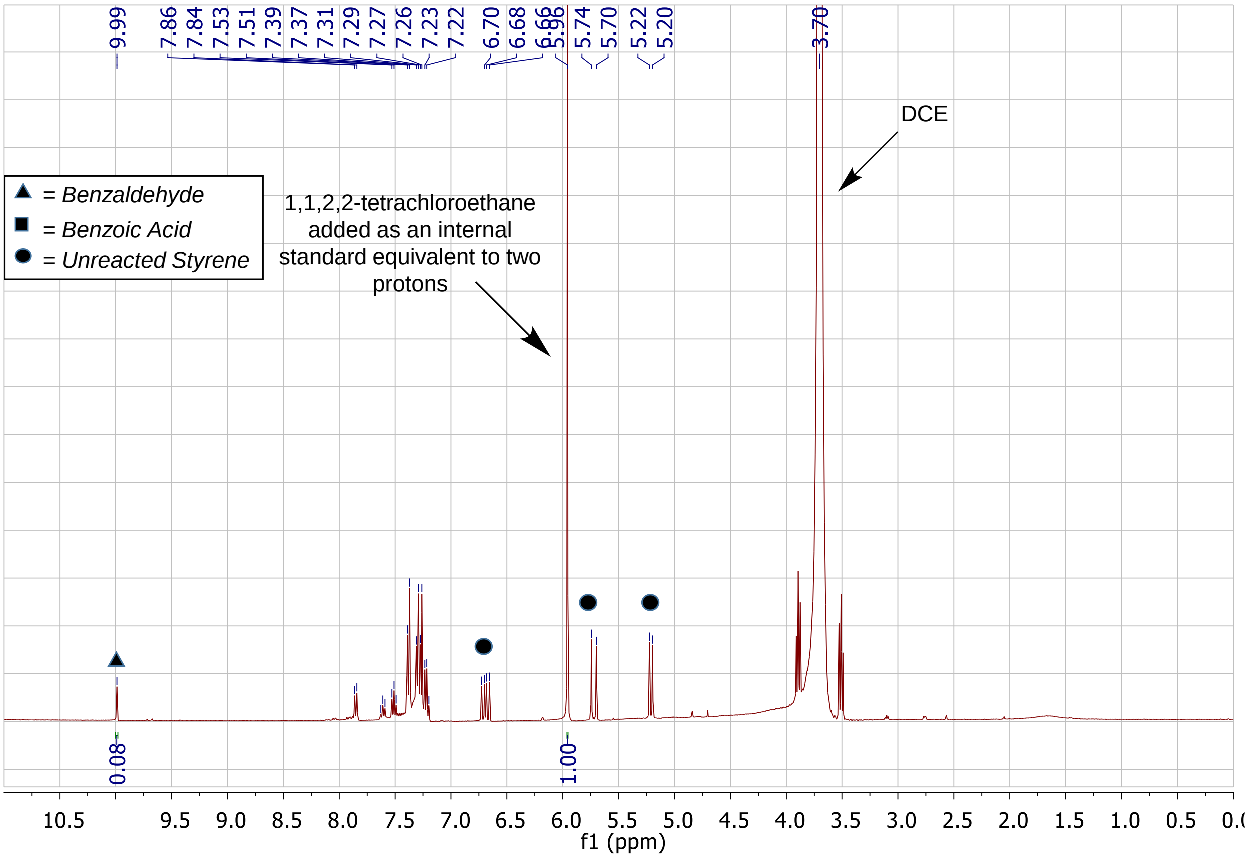

Figure S93: ^{1}H NMR spectrum of the styrene oxidation reaction in $CDCl_3$ using $Cs_2Ag_{0.4}Na_{0.6}BiCl_6$ as a photocatalyst in DCE as a solvent with a 50 W blue LED as the light source.

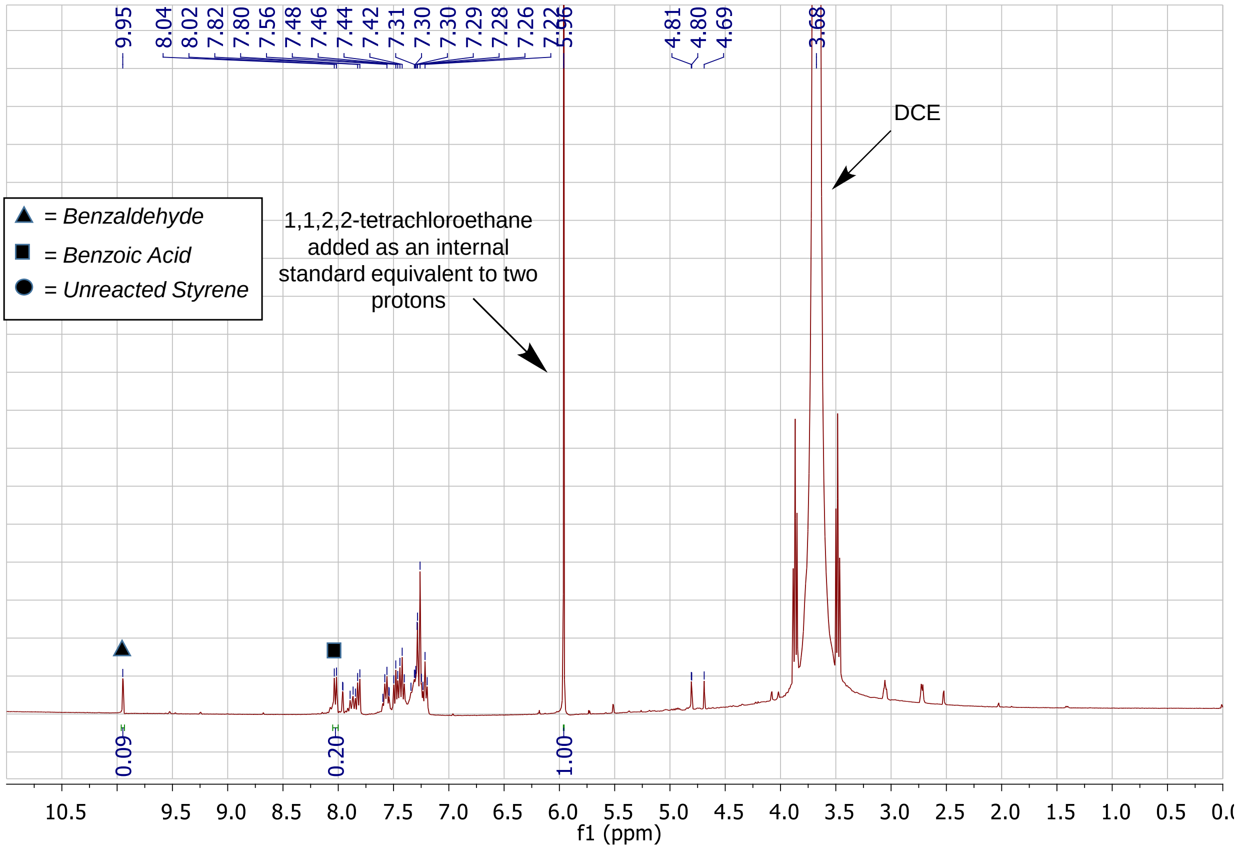

Figure S94: ^{1}H NMR spectrum of the styrene oxidation reaction in $CDCl_3$ using $Cs_2AgBiCl_6$ as a photocatalyst in DCE as a solvent with a 50 W blue LED as the light source.

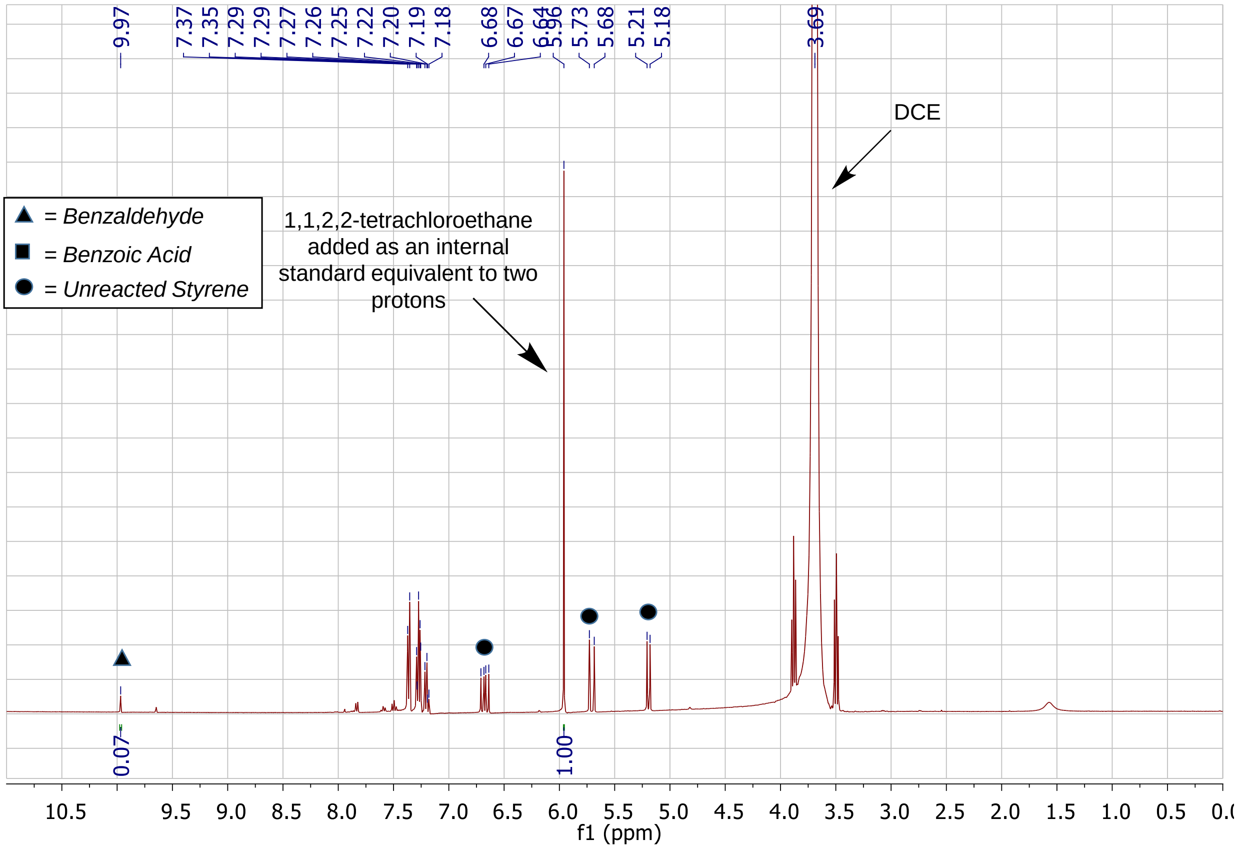

Figure S95: ^{1}H NMR spectrum of the styrene oxidation reaction in $CDCl_3$ using $Cs_2Ag_{0.05}Na_{0.95}BiCl_6$ as a photocatalyst in DCE as a solvent with a 50 W blue LED as the light source.

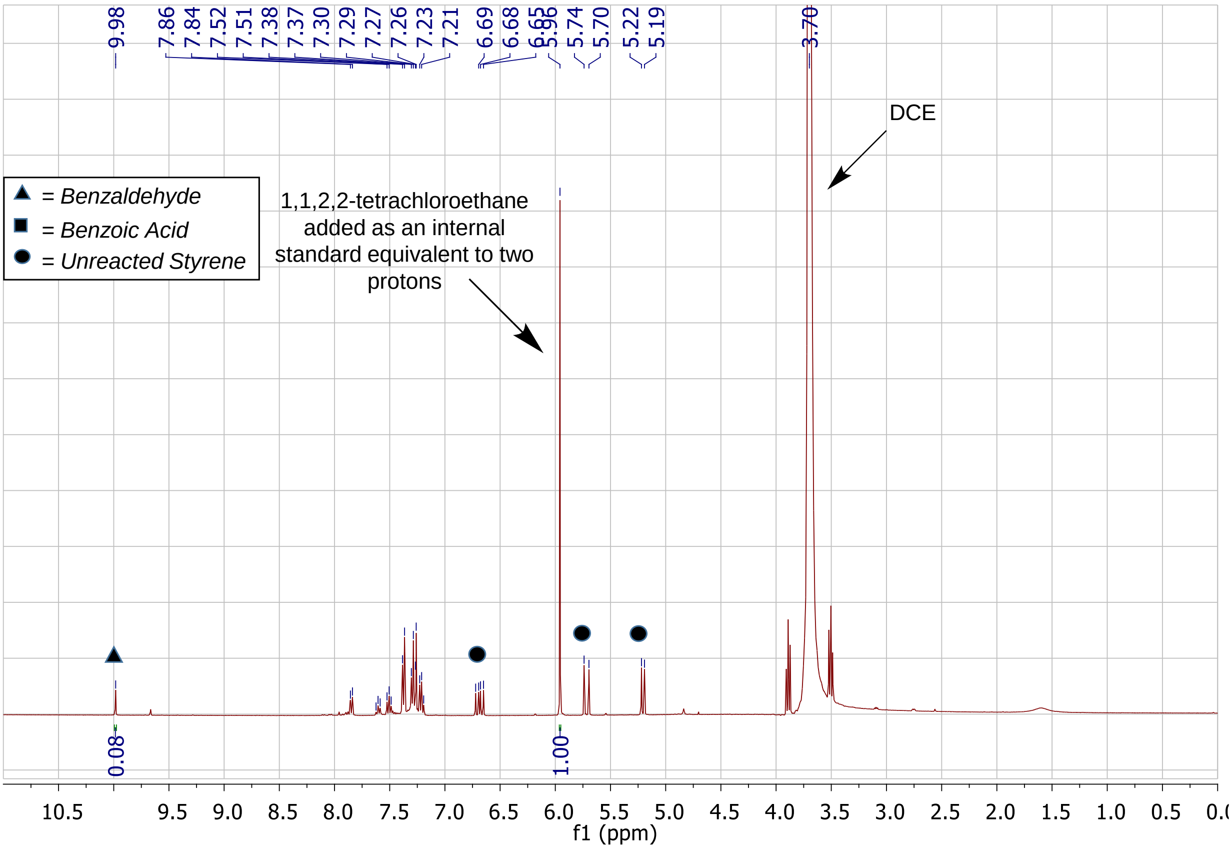

Figure S96: ^{1}H NMR spectrum of the styrene oxidation reaction in $CDCl_3$ using $Cs_2Ag_{0.2}Na_{0.8}BiCl_6$ as a photocatalyst in DCE as a solvent with a 50 W blue LED as the light source.

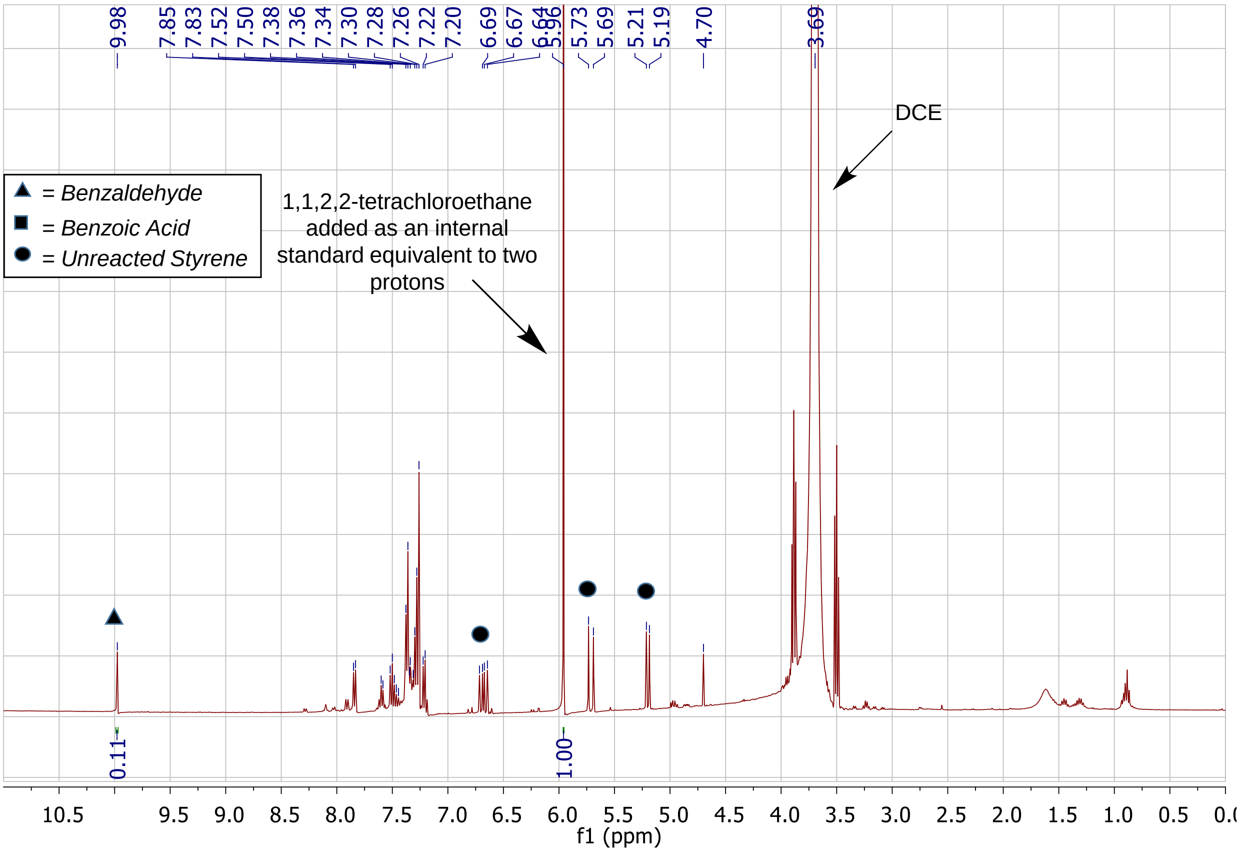

Figure S97: ^{1}H NMR spectrum of the styrene oxidation reaction in $CDCl_3$ using $BA_4AgInCl_8$ as a photocatalyst in DCE as a solvent with a 50 W blue LED as the light source.

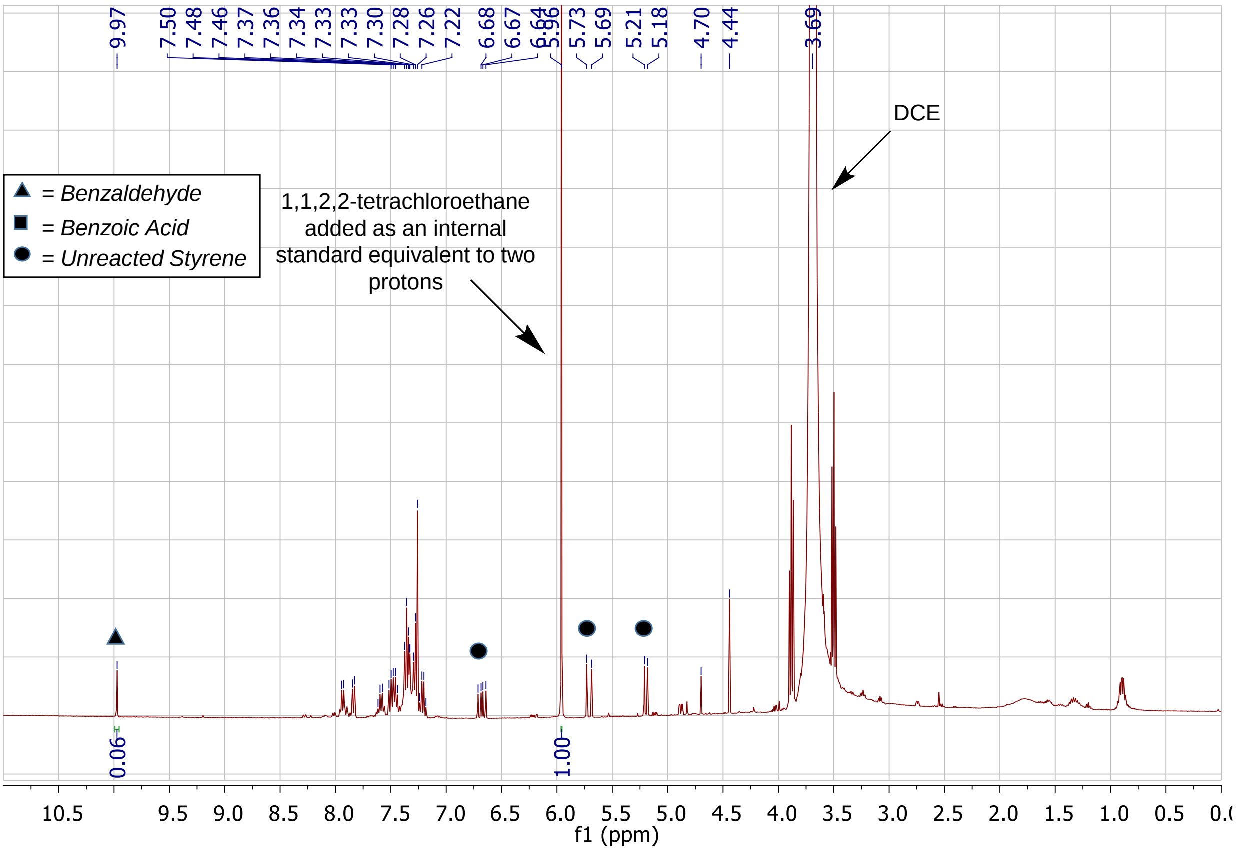

Figure S98: ^{1}H NMR spectrum of the styrene oxidation reaction in $CDCl_3$ using $BA_4AgBiBr_8$ as a photocatalyst in DCE as a solvent with a 50 W blue LED as the light source.

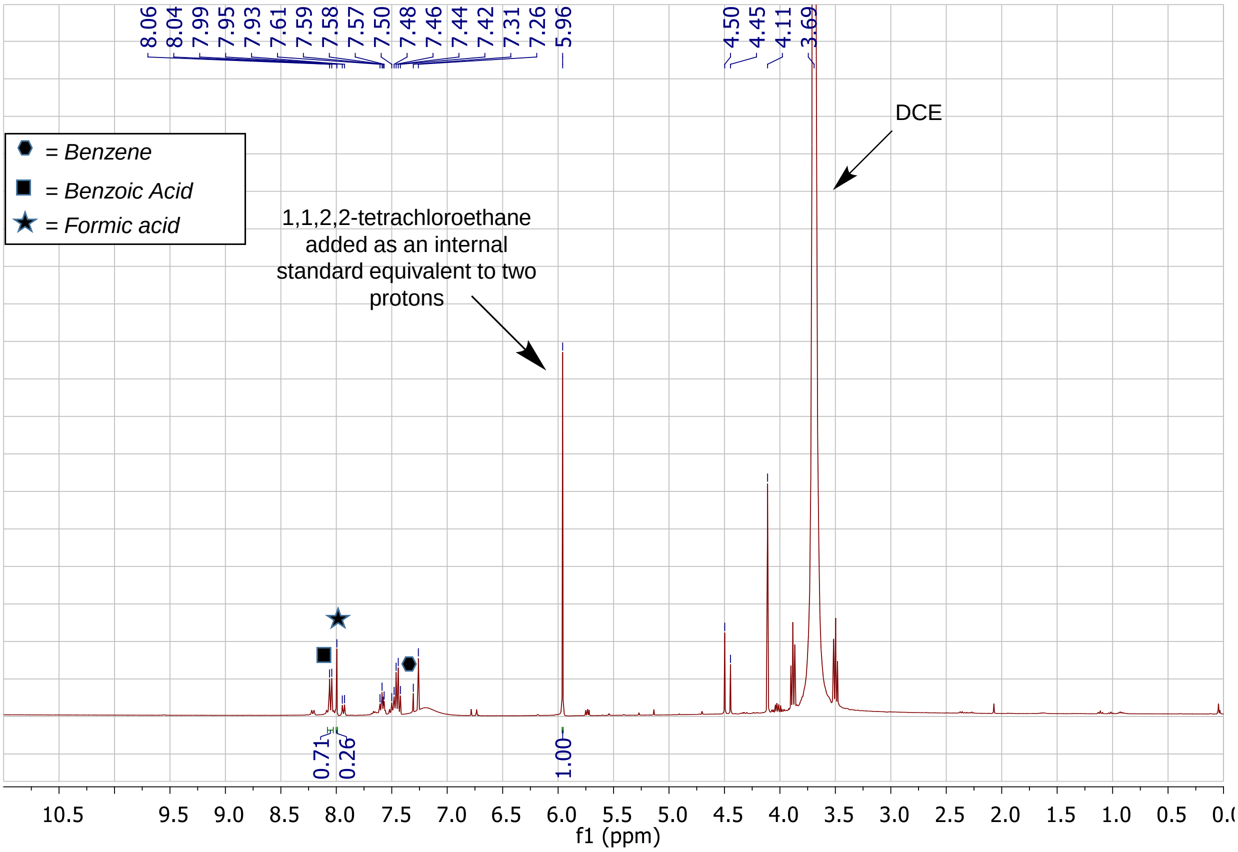

Figure S99: ^{1}H NMR spectrum of the styrene oxidation reaction in $CDCl_3$ using $BA_2CsAgBiBr_7$ as a photocatalyst in DCE as a solvent with a 50 W blue LED as the light source.

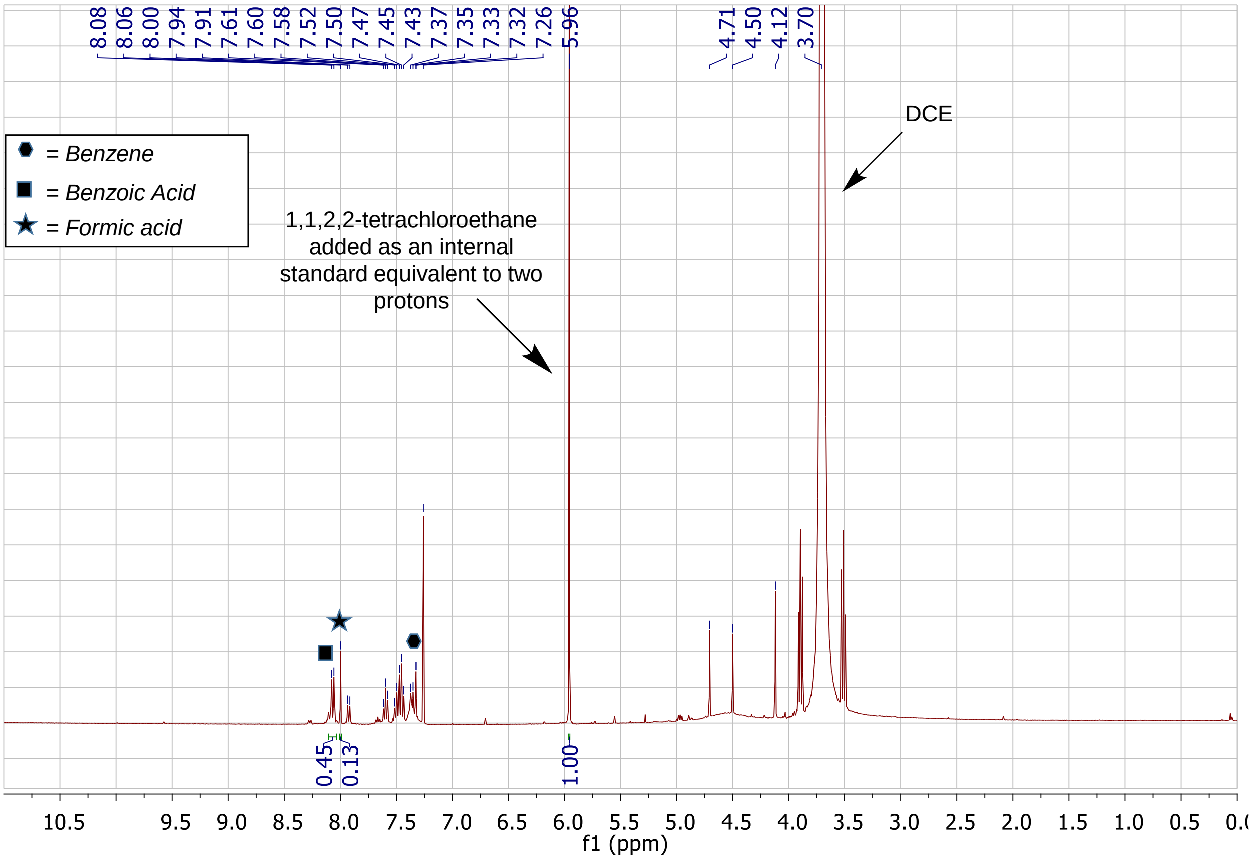

Figure S100: ^{1}H NMR spectrum of the styrene oxidation reaction in $CDCl_3$ using $Cs_2Ag_{0.95}Na_{0.05}BiCl_6$ as a photocatalyst in DCE as a solvent with a 50 W blue LED as the light source.

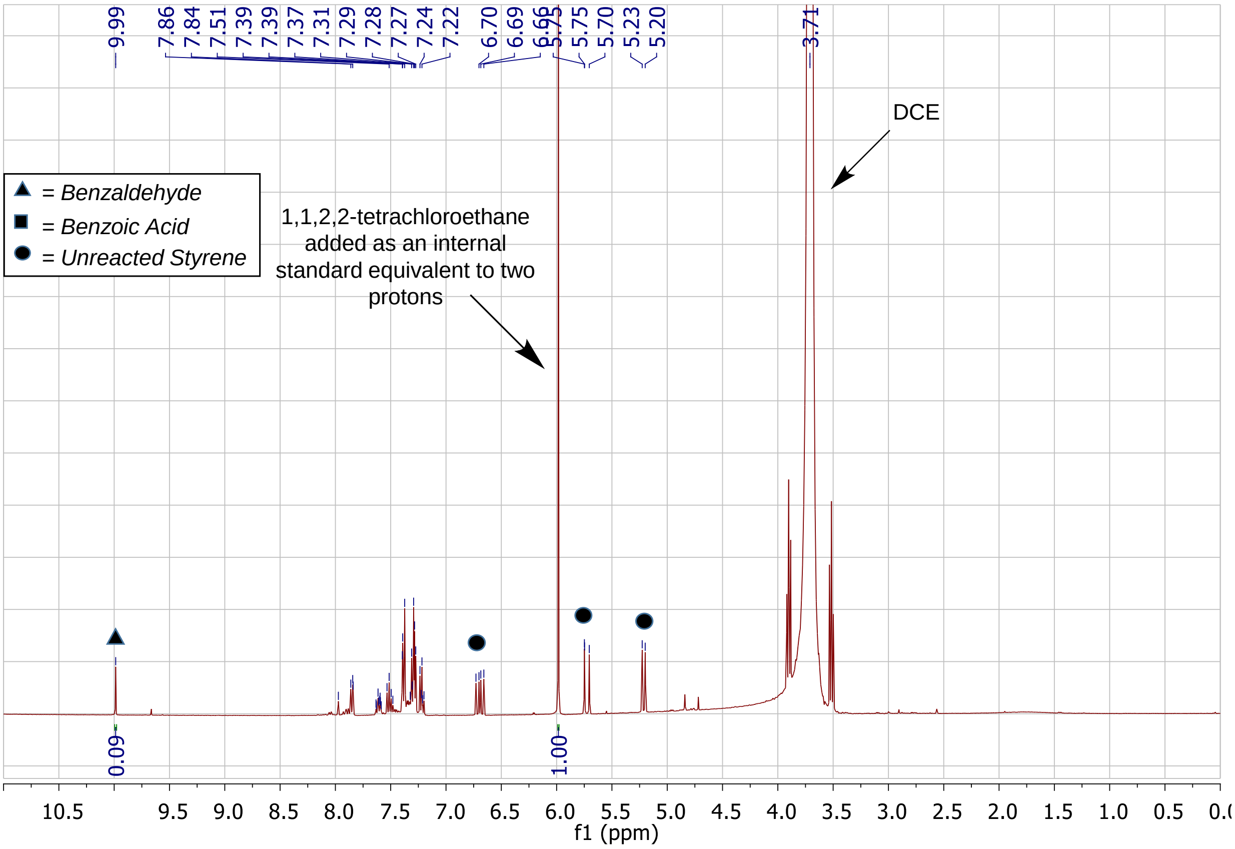

Figure S101: ^{1}H NMR spectrum of the styrene oxidation reaction in $CDCl_3$ using $MA_3Bi_2Cl_9$ as a photocatalyst in DCE as a solvent with a 50 W blue LED as the light source.

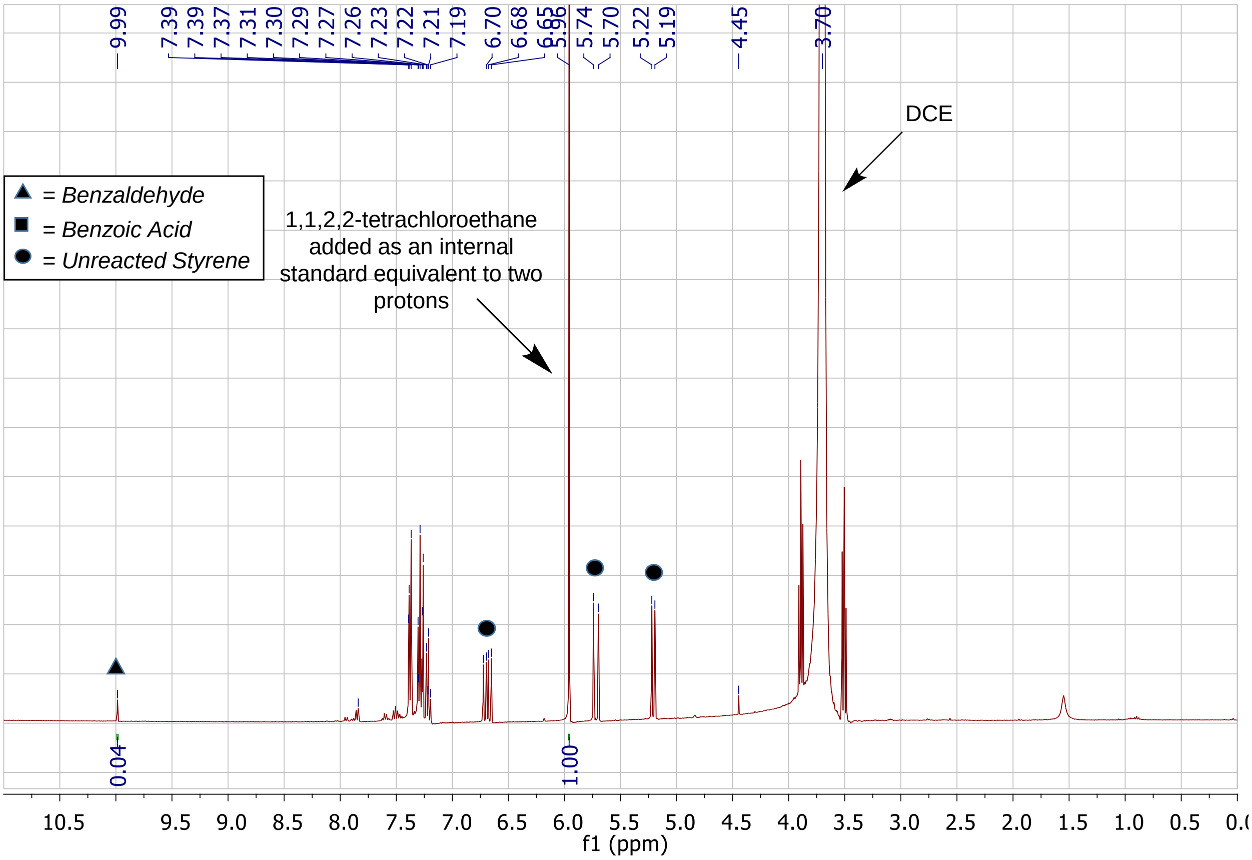

Figure S102: ^{1}H NMR spectrum of the styrene oxidation reaction in $CDCl_3$ using $PA_4AgBiBr_8$ as a photocatalyst in DCE as a solvent with a 50 W blue LED as the light source.

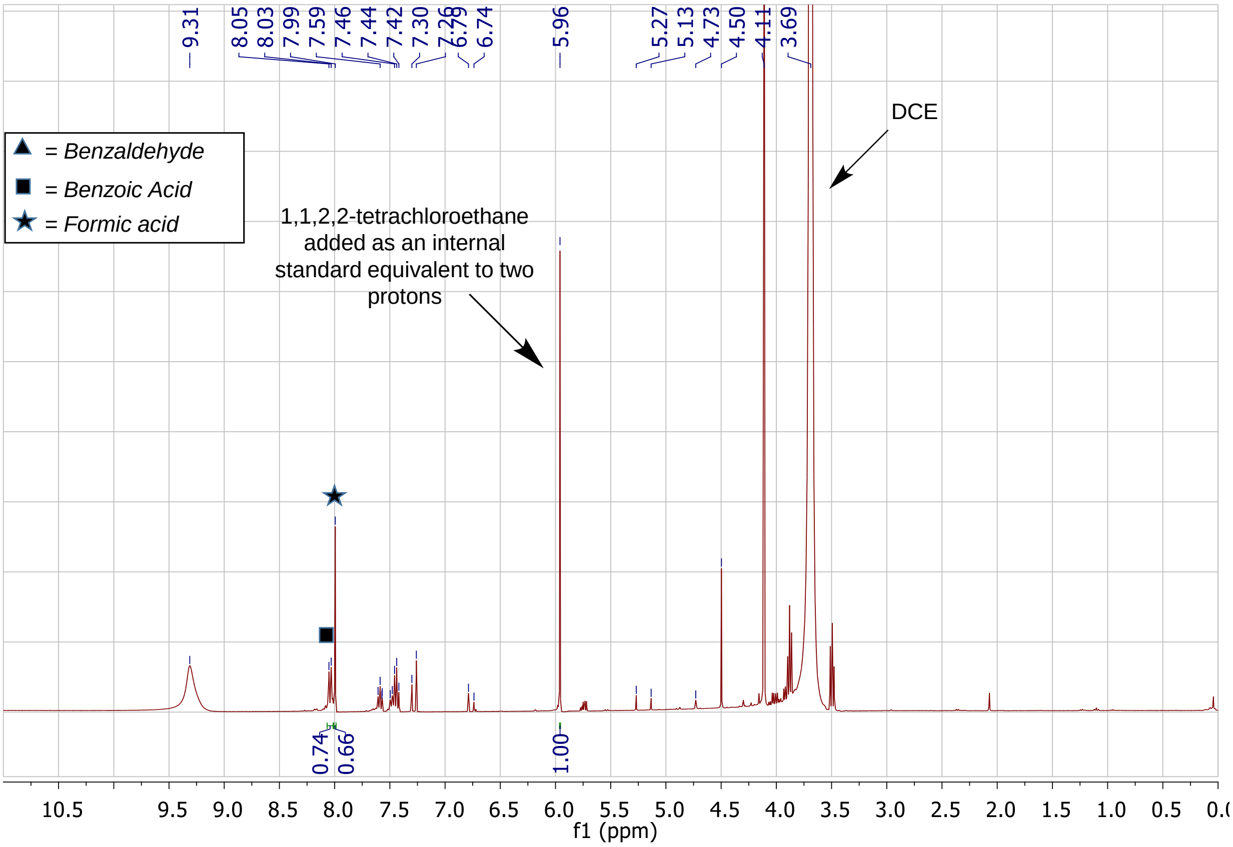

Figure S103: ^{1}H NMR spectrum of the styrene oxidation reaction in $CDCl_3$ using $PA_2CsAgBiBr_7$ as a photocatalyst in DCE as a solvent with a 50 W blue LED as the light source.

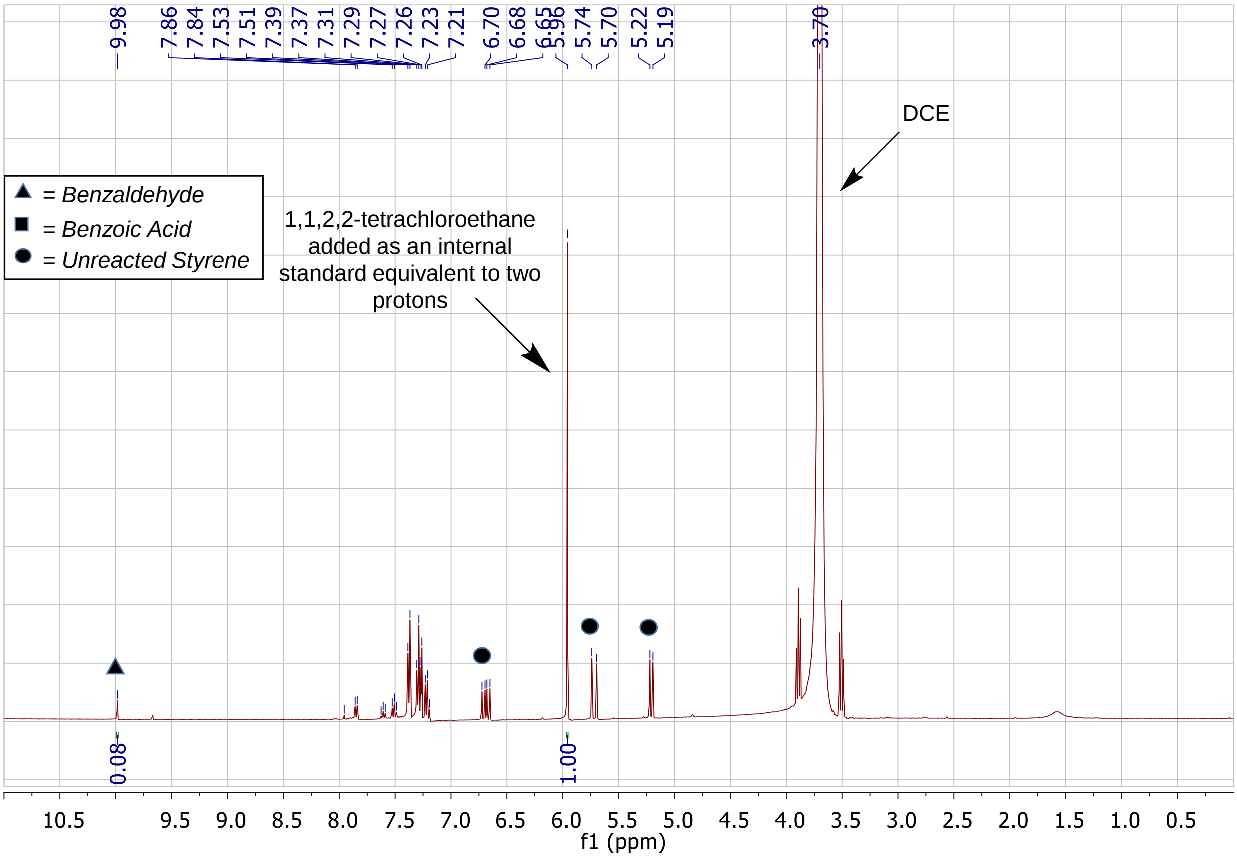

Figure S104: ^{1}H NMR spectrum of the styrene oxidation reaction in $CDCl_3$ using Cs_2KInCl_6 as a photocatalyst in DCE as a solvent with a 50 W blue LED as the light source.

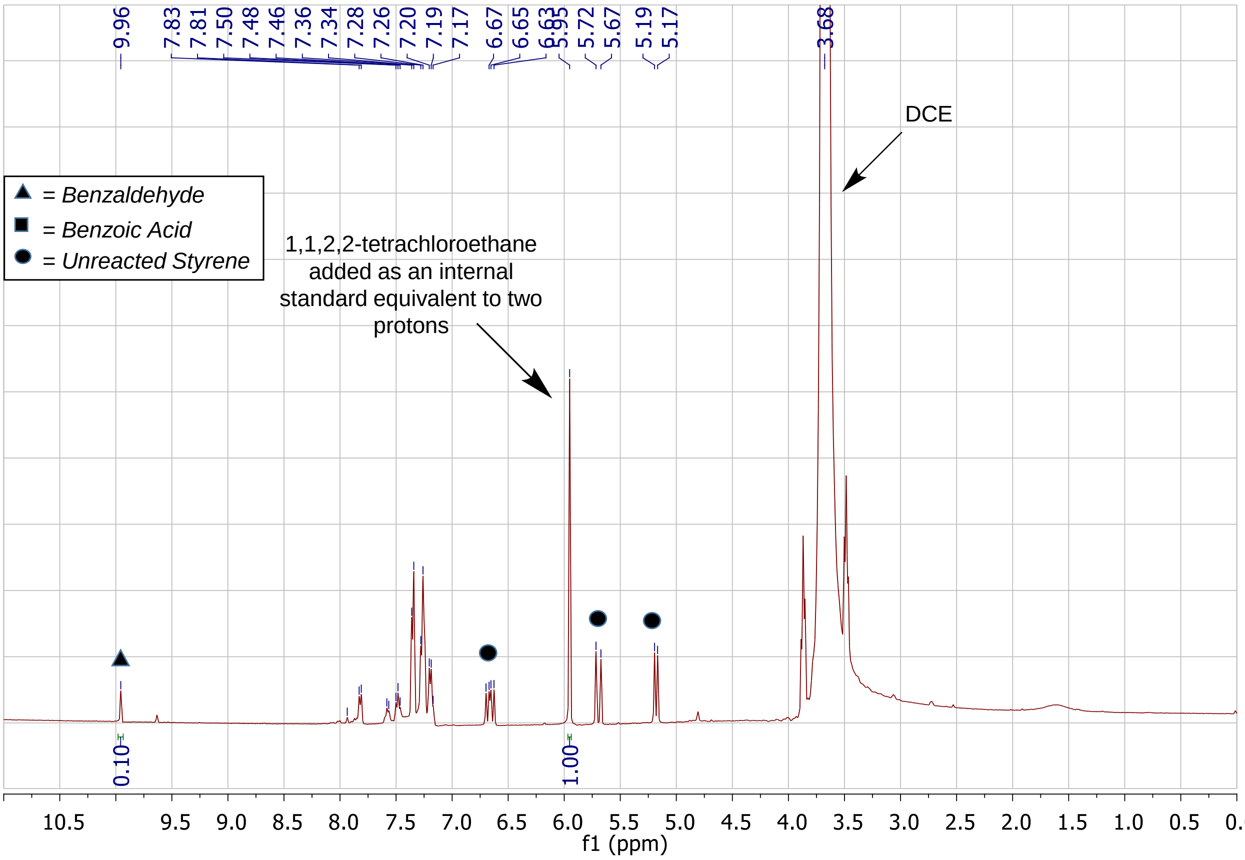

Figure S105: ^{1}H NMR spectrum of the styrene oxidation reaction in $CDCl_3$ using $Cs_2Ag_{0.1}Na_{0.9}BiCl_6$ as a photocatalyst in DCE as a solvent with a 50 W blue LED as the light source.

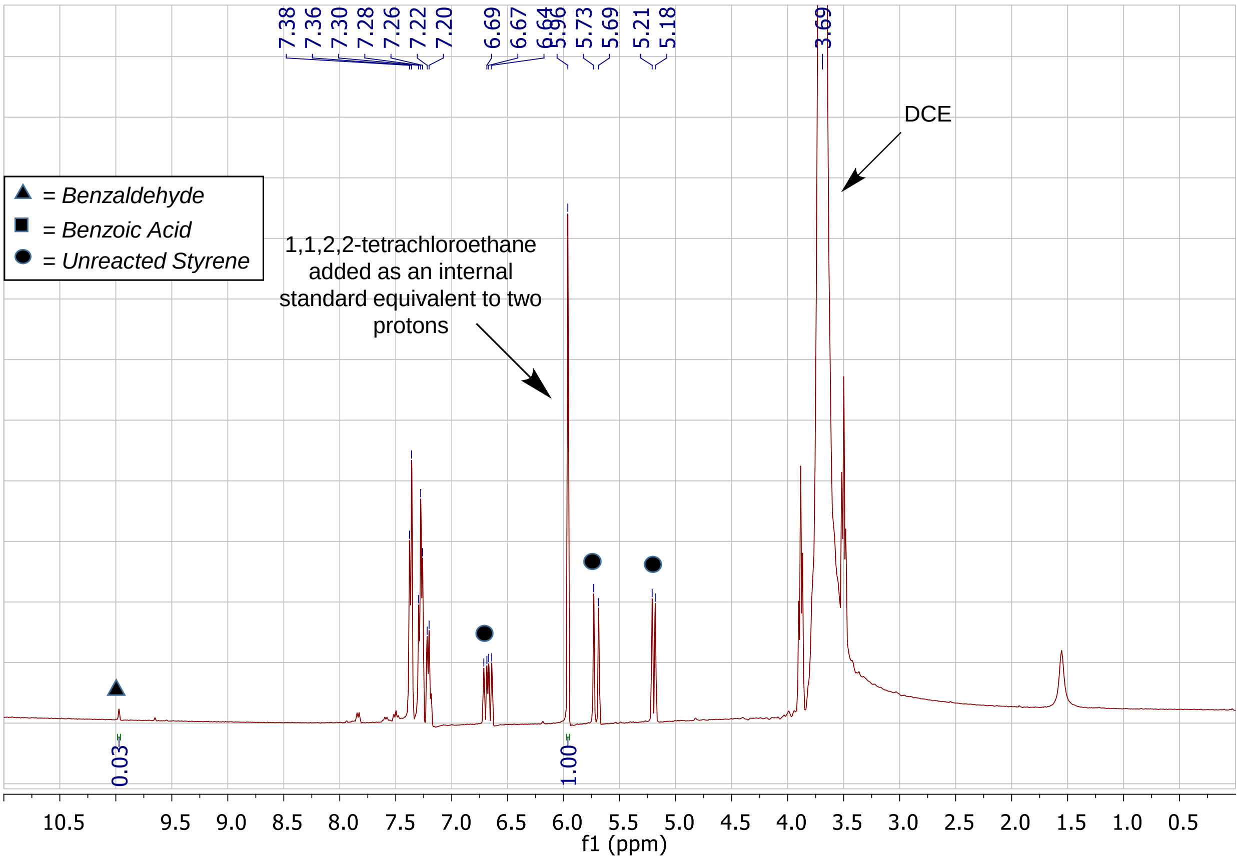

Figure S106: ^{1}H NMR spectrum of the styrene oxidation reaction in $CDCl_3$ using $FASnCl_3$ as a photocatalyst in DCE as a solvent with a 50 W blue LED as the light source.

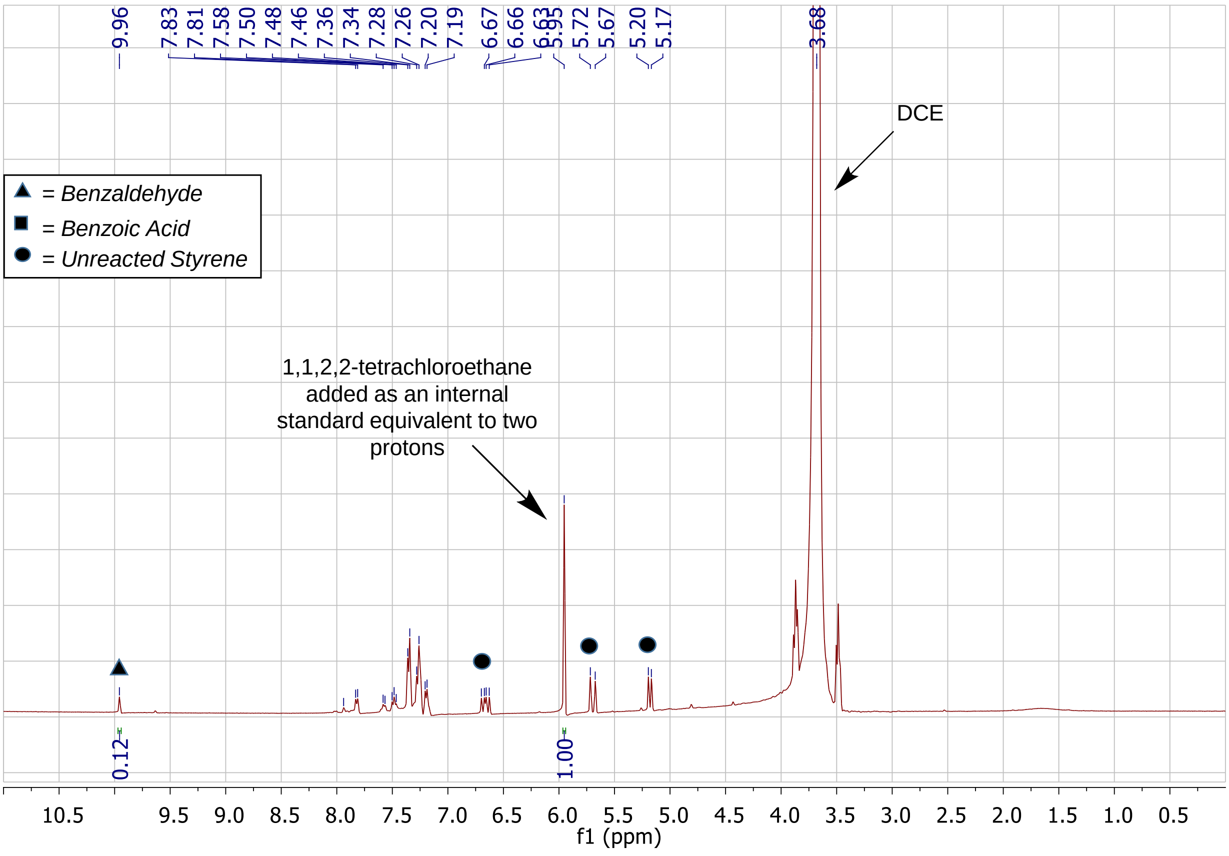

Figure S107: ^{1}H NMR spectrum of the styrene oxidation reaction in $CDCl_3$ using $FASnBr_3$ as a photocatalyst in DCE as a solvent with a 50 W blue LED as the light source.

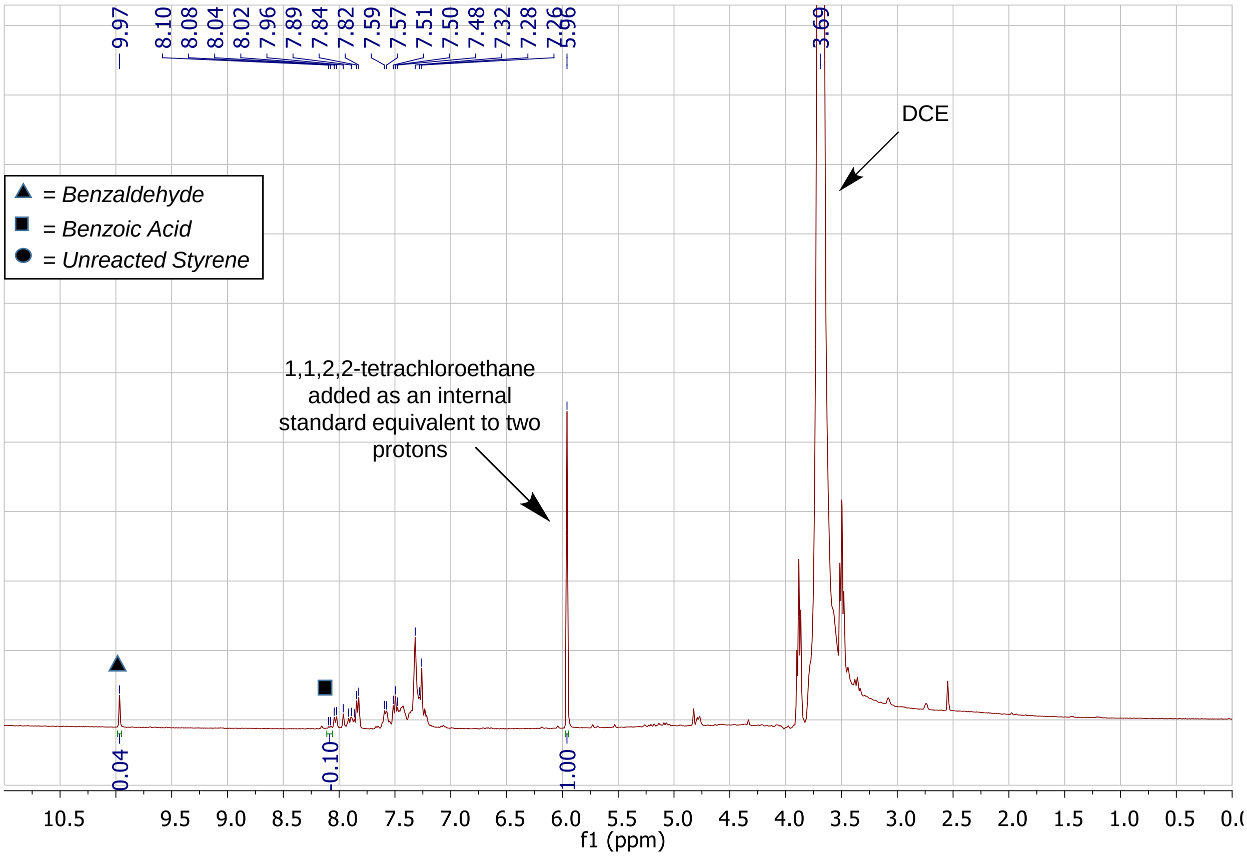

Figure S108: ^{1}H NMR spectrum of the styrene oxidation reaction in $CDCl_3$ using $FASnI_3$ as a photocatalyst in DCE as a solvent with a 50 W blue LED as the light source.

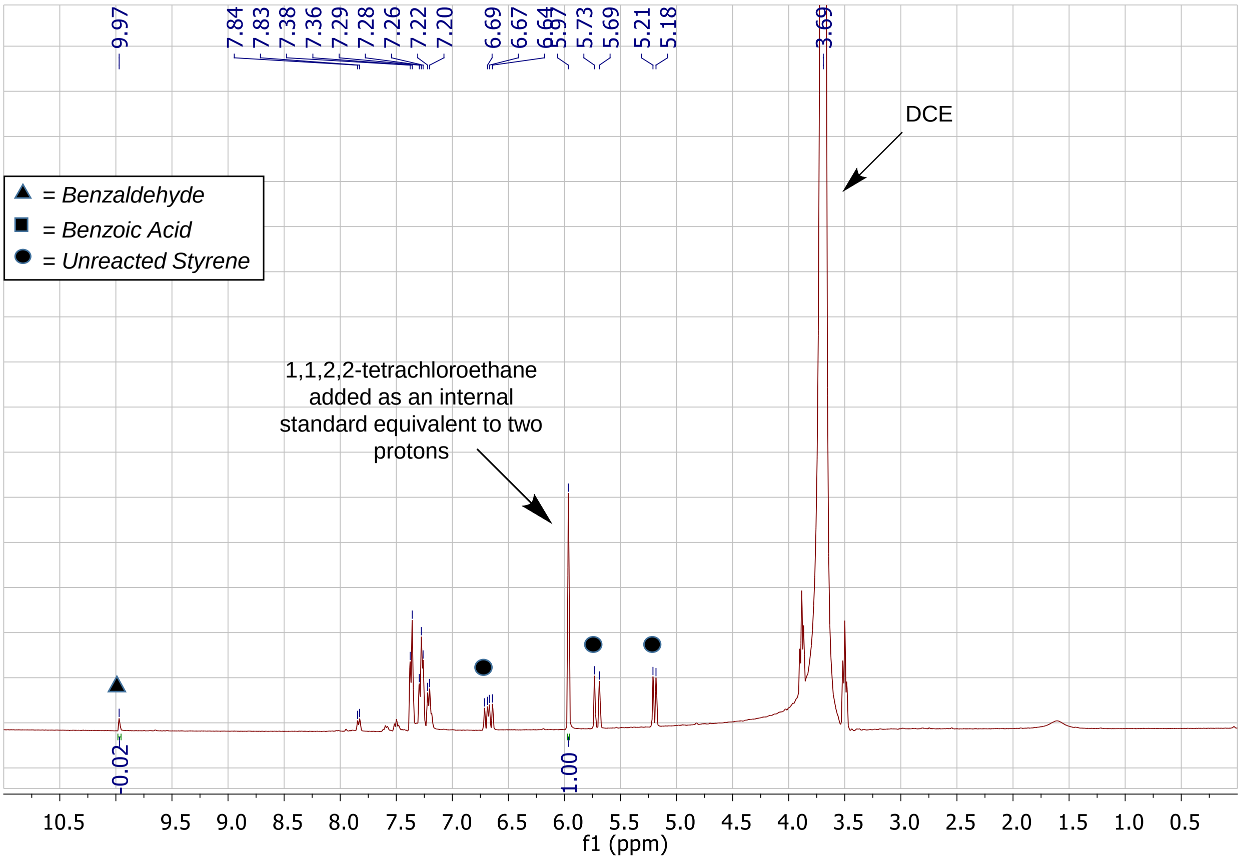

Figure S109: ^{1}H NMR spectrum of the styrene oxidation reaction in $CDCl_3$ using $MASnCl_3$ as a photocatalyst in DCE as a solvent with a 50 W blue LED as the light source.

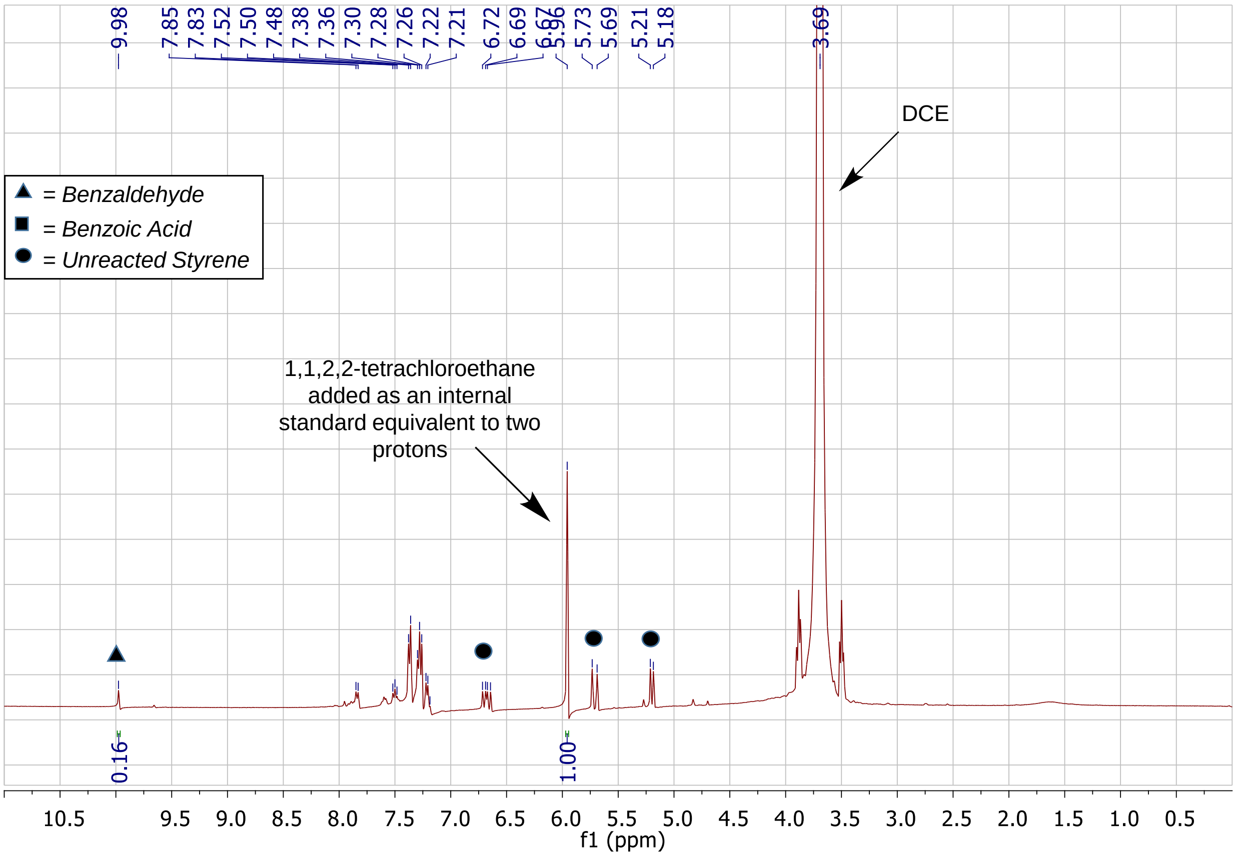

Figure S110: ^{1}H NMR spectrum of the styrene oxidation reaction in $CDCl_3$ using $Rb_2AgBiCl_6$ as a photocatalyst in DCE as a solvent with a 50 W blue LED as the light source.

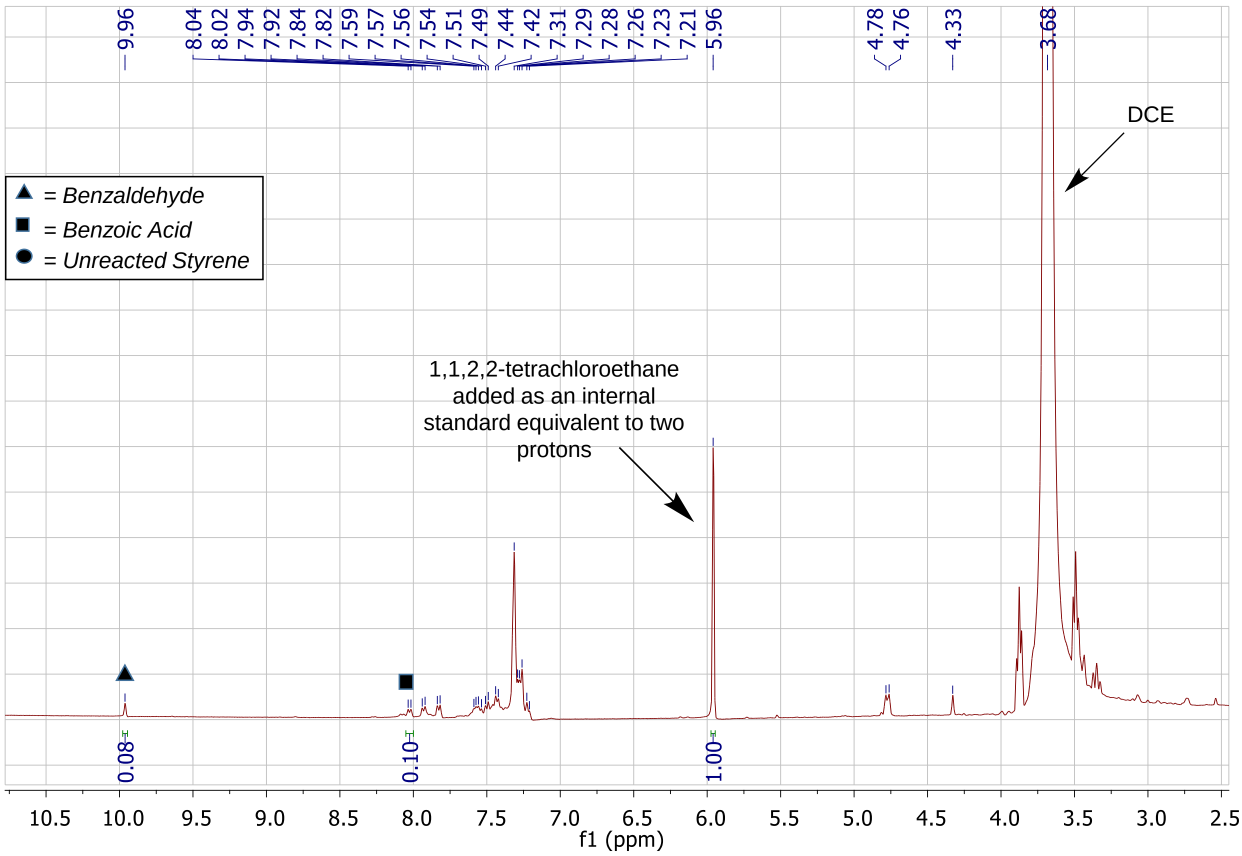

Figure S111: ^{1}H NMR spectrum of the styrene oxidation reaction in $CDCl_3$ using $MA_3Bi_2I_9$ as a photocatalyst in DCE as a solvent with a 50 W blue LED as the light source.

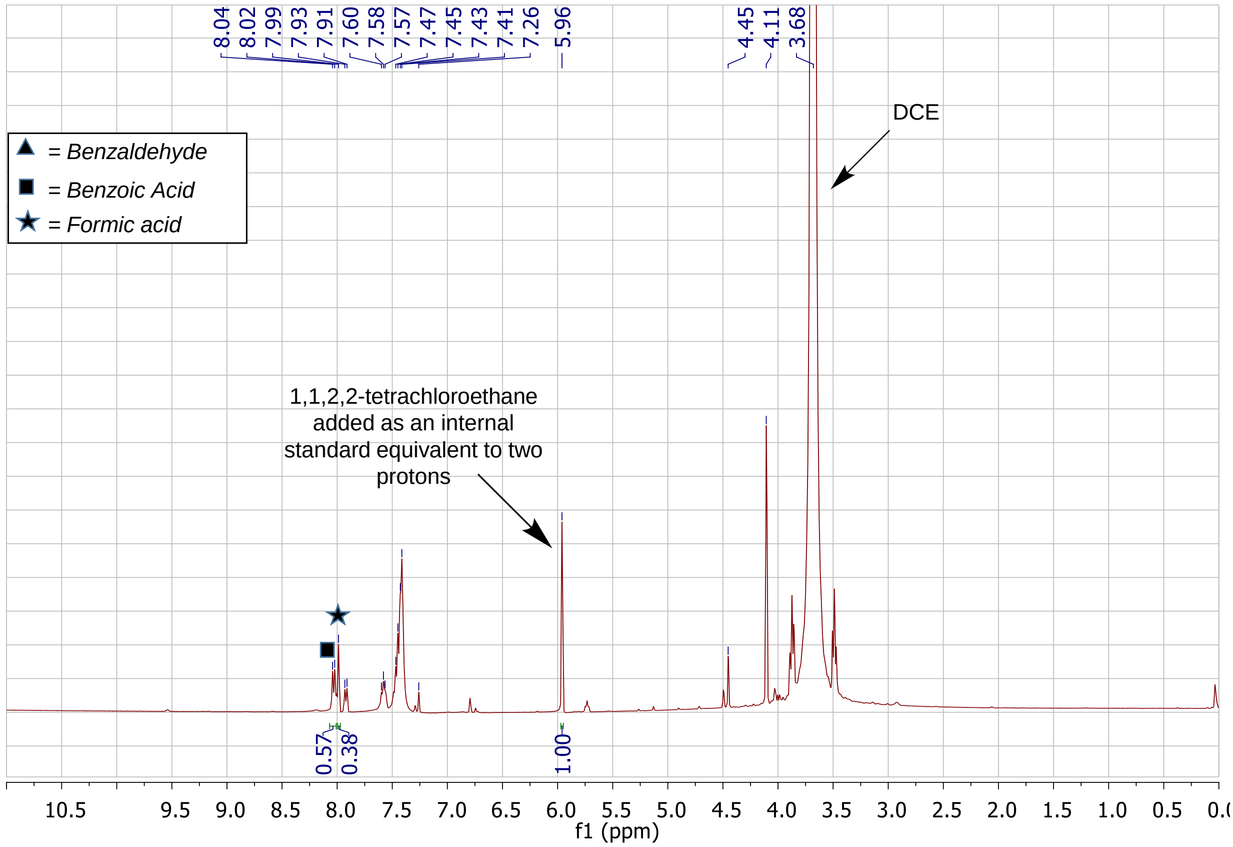

Figure S112: ^{1}H NMR spectrum of the styrene oxidation reaction in $CDCl_3$ using $MA_3Sb_2Br_9$ as a photocatalyst in DCE as a solvent with a 50 W blue LED as the light source.

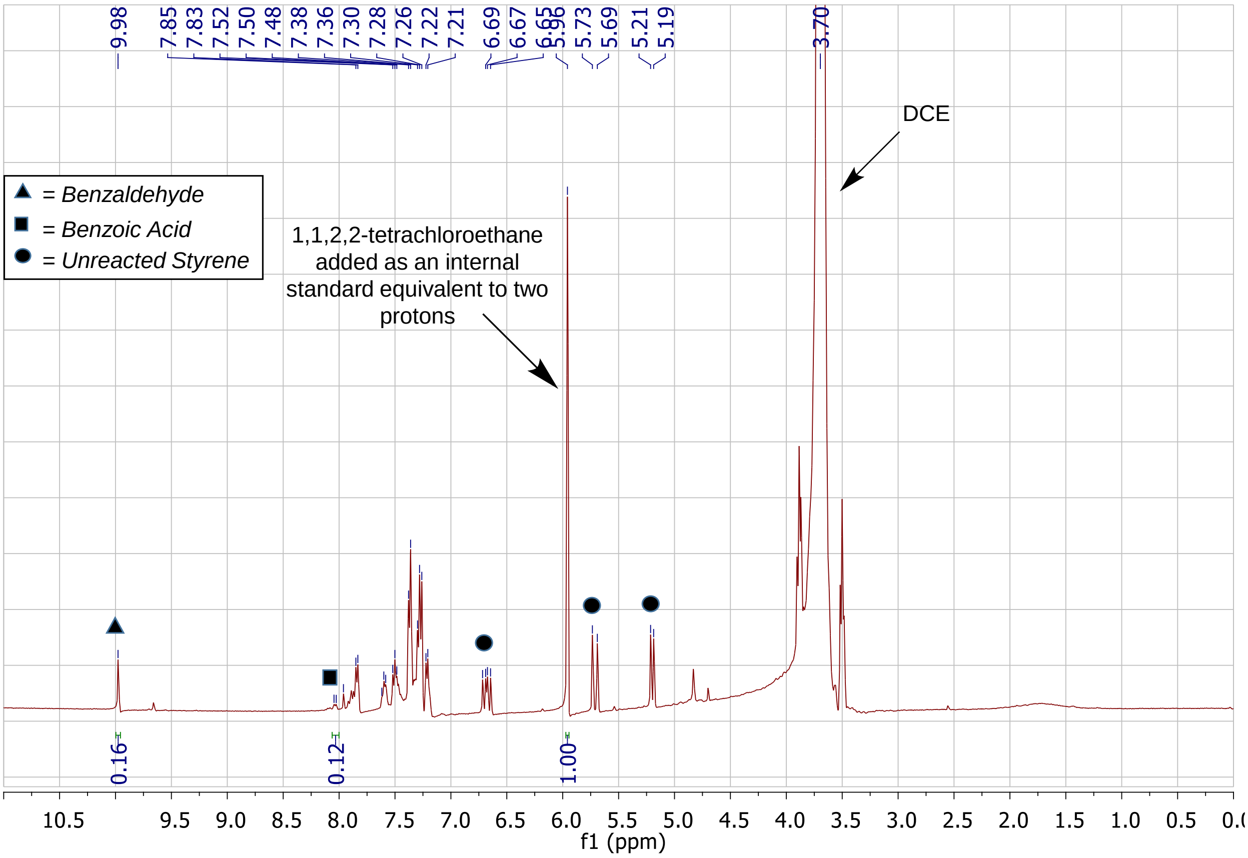

Figure S113: ^{1}H NMR spectrum of the styrene oxidation reaction in $CDCl_3$ using $FA_3Bi_2Cl_9$ as a photocatalyst in DCE as a solvent with a 50 W blue LED as the light source.

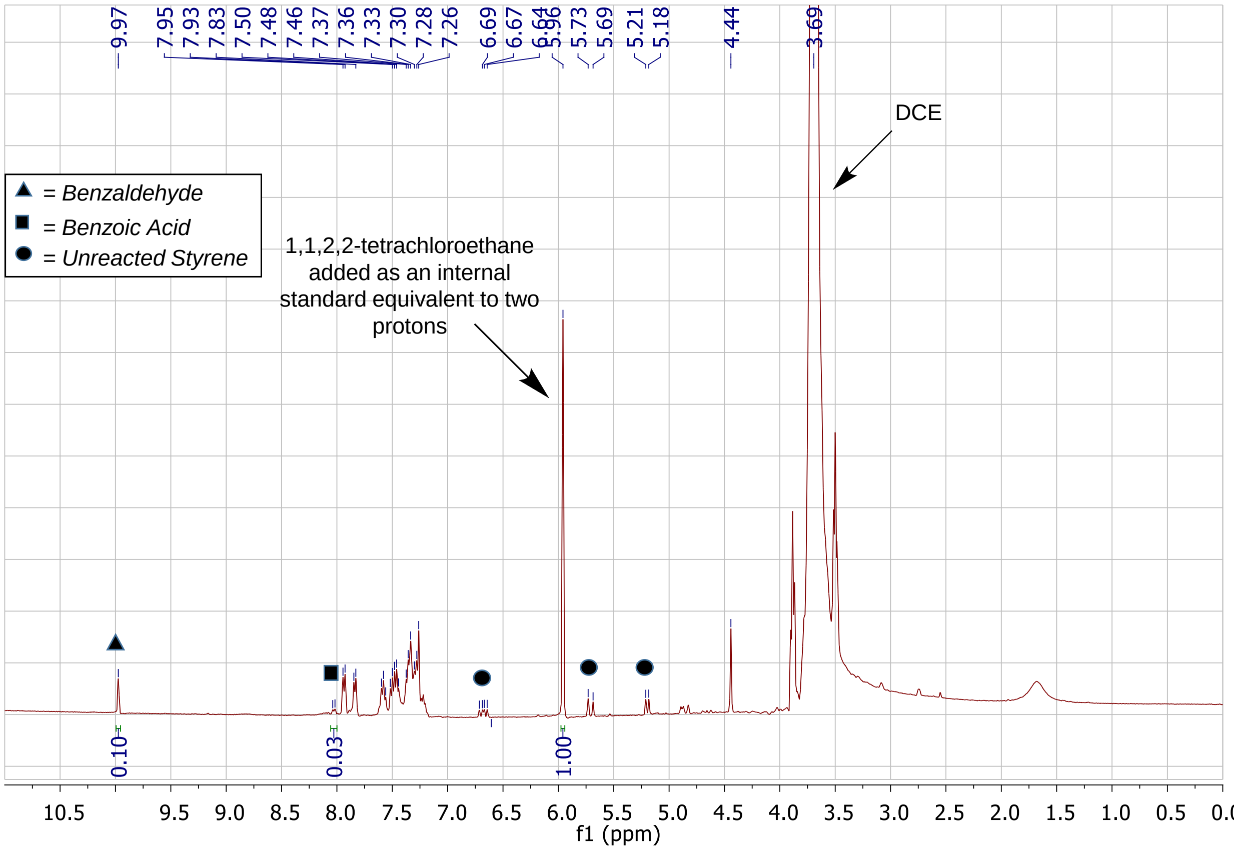

Figure S114: ^{1}H NMR spectrum of the styrene oxidation reaction in $CDCl_3$ using $FA_3Bi_2Br_9$ as a photocatalyst in DCE as a solvent with a 50 W blue LED as the light source.

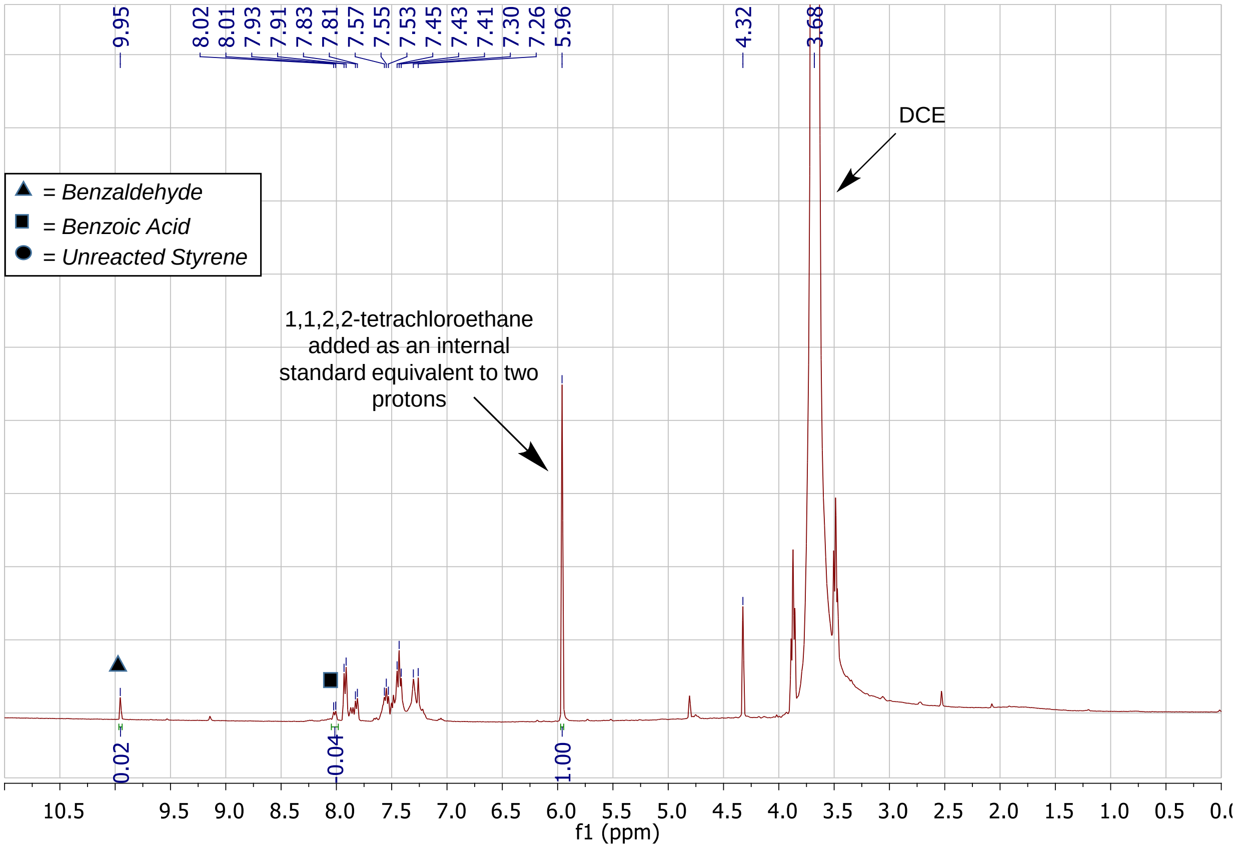

Figure S115: ^{1}H NMR spectrum of the styrene oxidation reaction in $CDCl_3$ using $FA_3Bi_2I_9$ as a photocatalyst in DCE as a solvent with a 50 W blue LED as the light source.

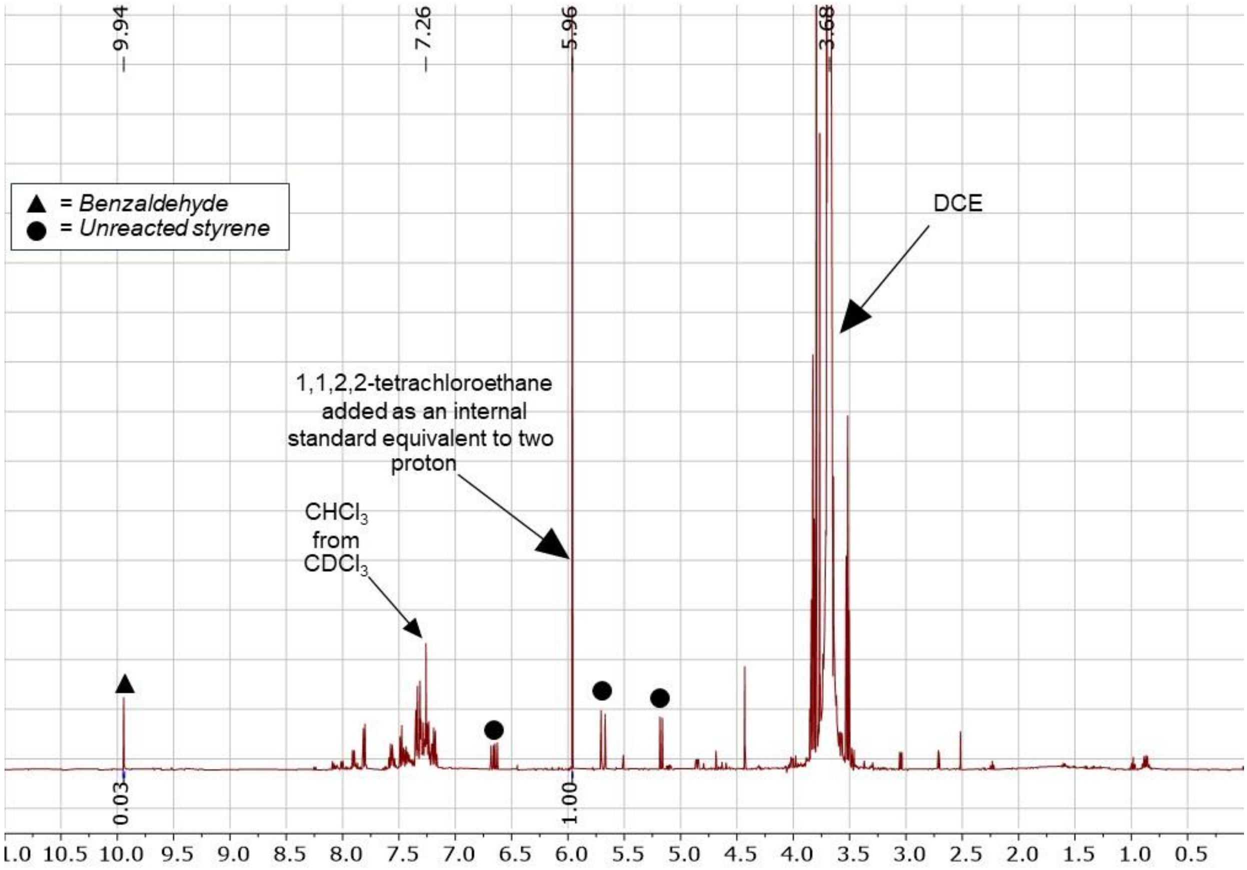

Figure S116: ^{1}H NMR spectrum of the styrene oxidation reaction in $CDCl_3$ using $PA_2CsAg_{0.95}Na_{0.05}BiBr_7$ as a photocatalyst in DCE as a solvent with a 50 W blue LED as the light source.

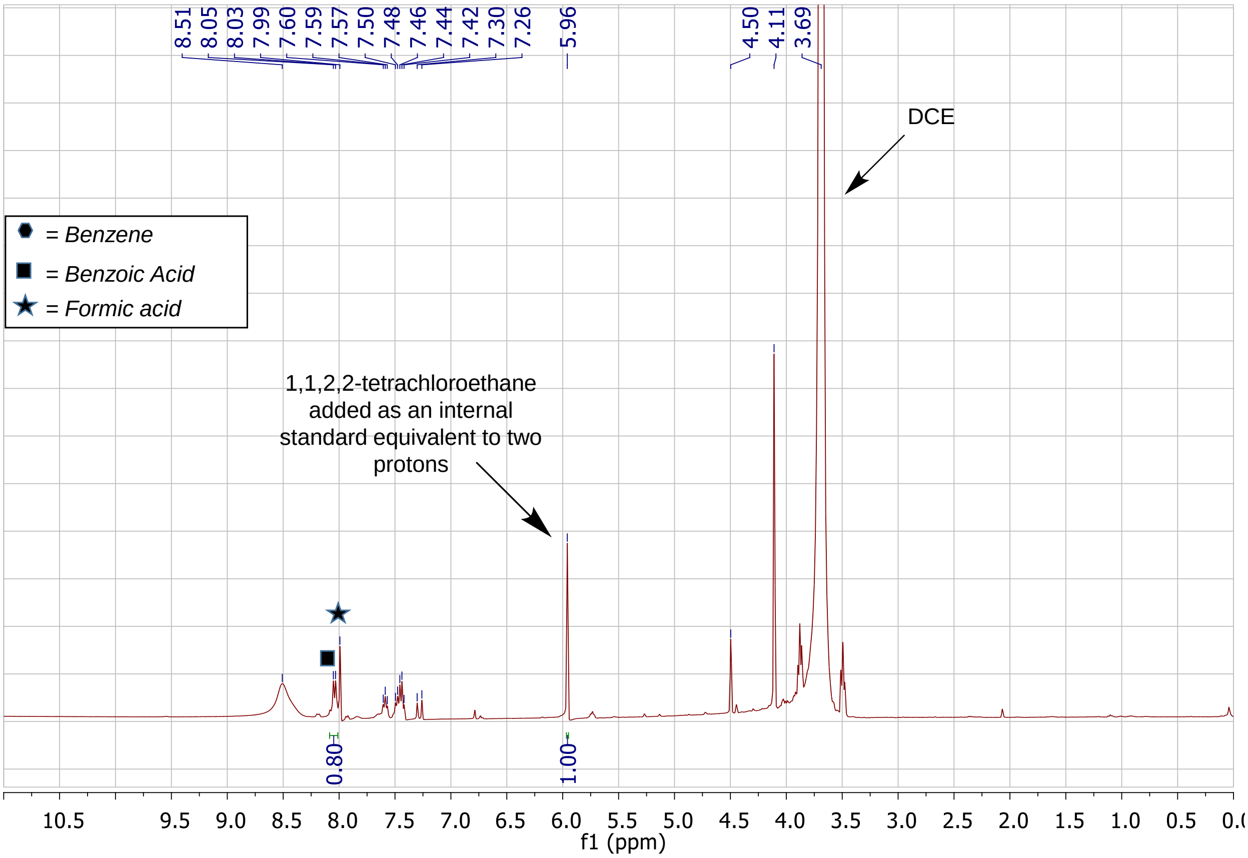

Figure S117: ^{1}H NMR spectrum of the styrene oxidation reaction in $CDCl_3$ using $BA_2CsAg_{0.95}Na_{0.05}BiBr_7$ as a photocatalyst in DCE as a solvent with a 50 W blue LED as the light source.

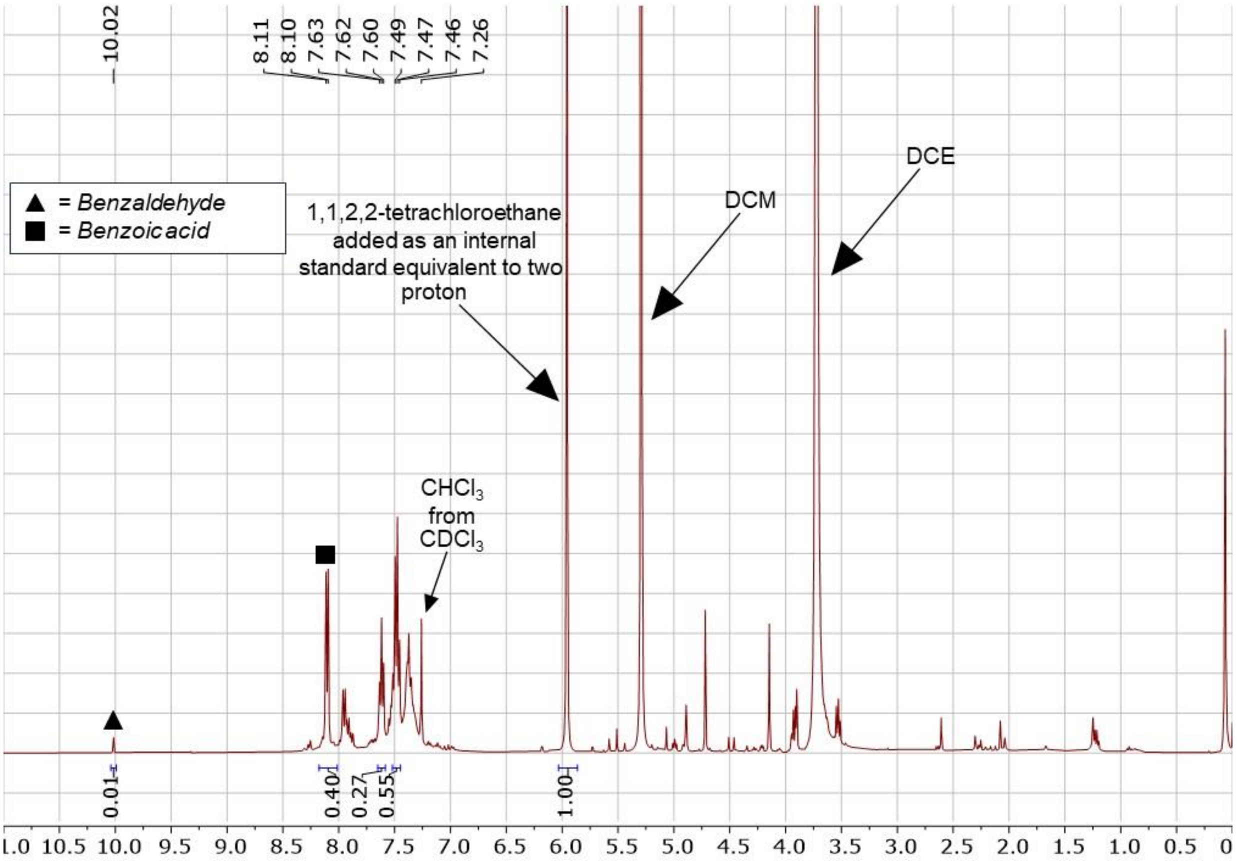

Figure S118: ^{1}H NMR spectrum of the styrene oxidation reaction in $CDCl_3$ using $BA_2CsAg_{0.95}Na_{0.05}BiBr_7$, which had been collected by centrifugation after one round of reaction and reused in a second round as a photocatalyst, in DCE as a solvent with a 50 W blue LED as the light source.

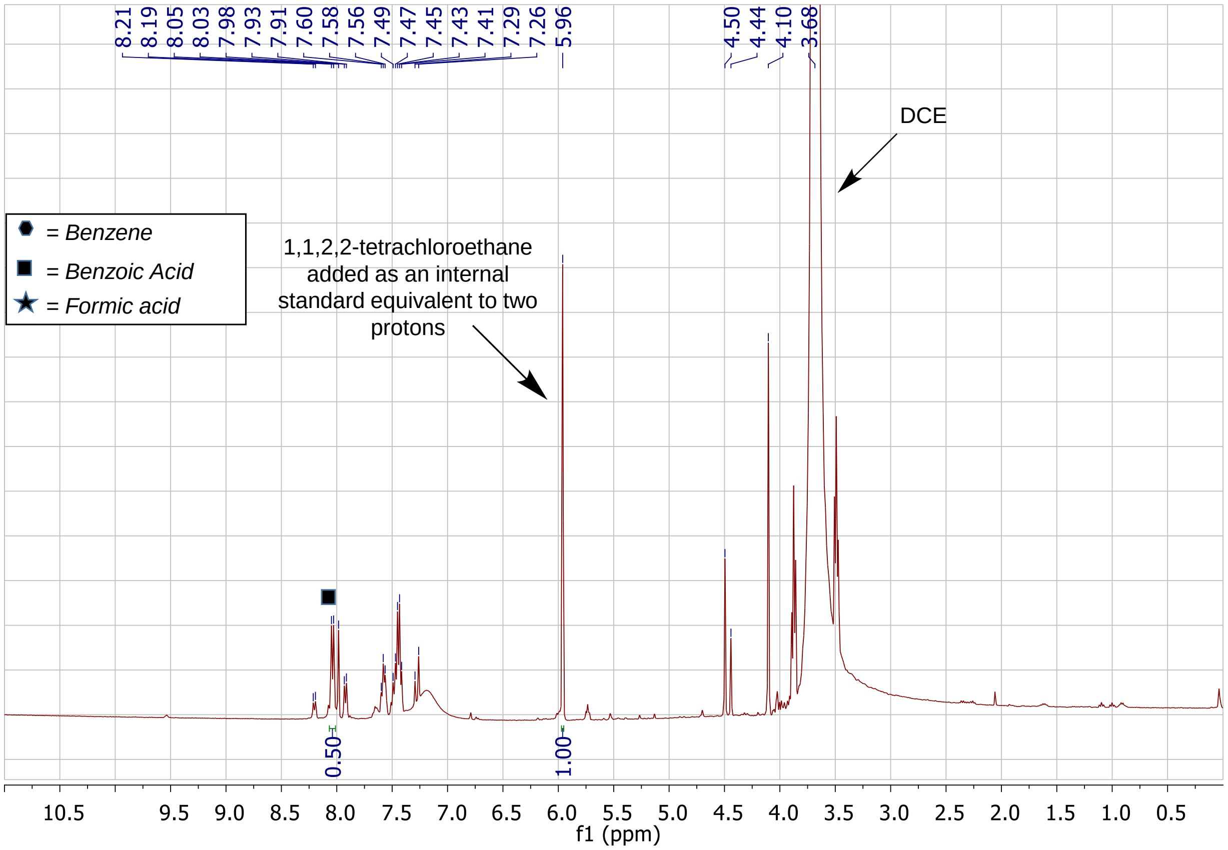

Figure S119: ^{1}H NMR spectrum of the styrene oxidation reaction in $CDCl_3$ using $BA_2CsAg_{0.9}Na_{0.1}BiBr_7$ as a photocatalyst in DCE as a solvent with a 50 W blue LED as the light source.

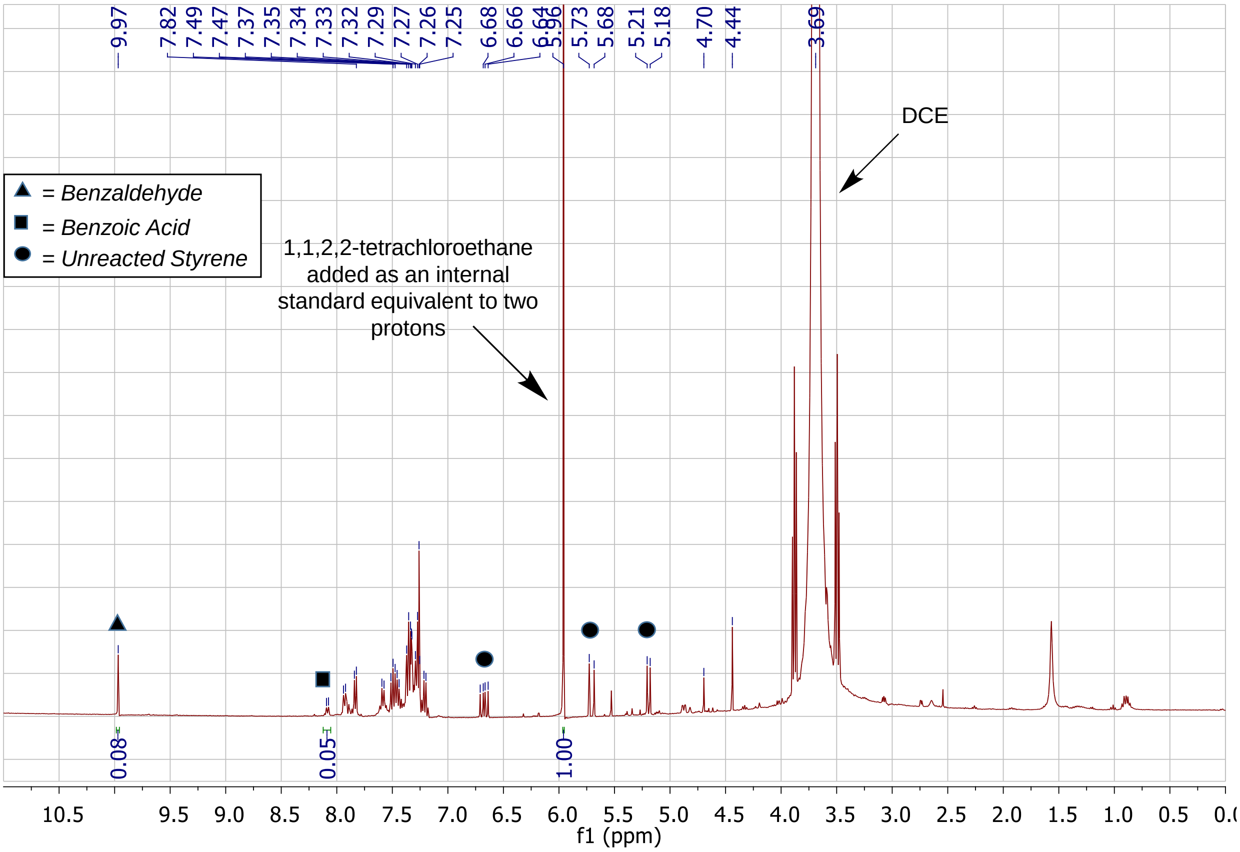

Figure S120: ^{1}H NMR spectrum of the styrene oxidation reaction in $CDCl_3$ using $BA_2CsAg_{0.25}Na_{0.75}BiBr_7$ as a photocatalyst in DCE as a solvent with a 50 W blue LED as the light source.

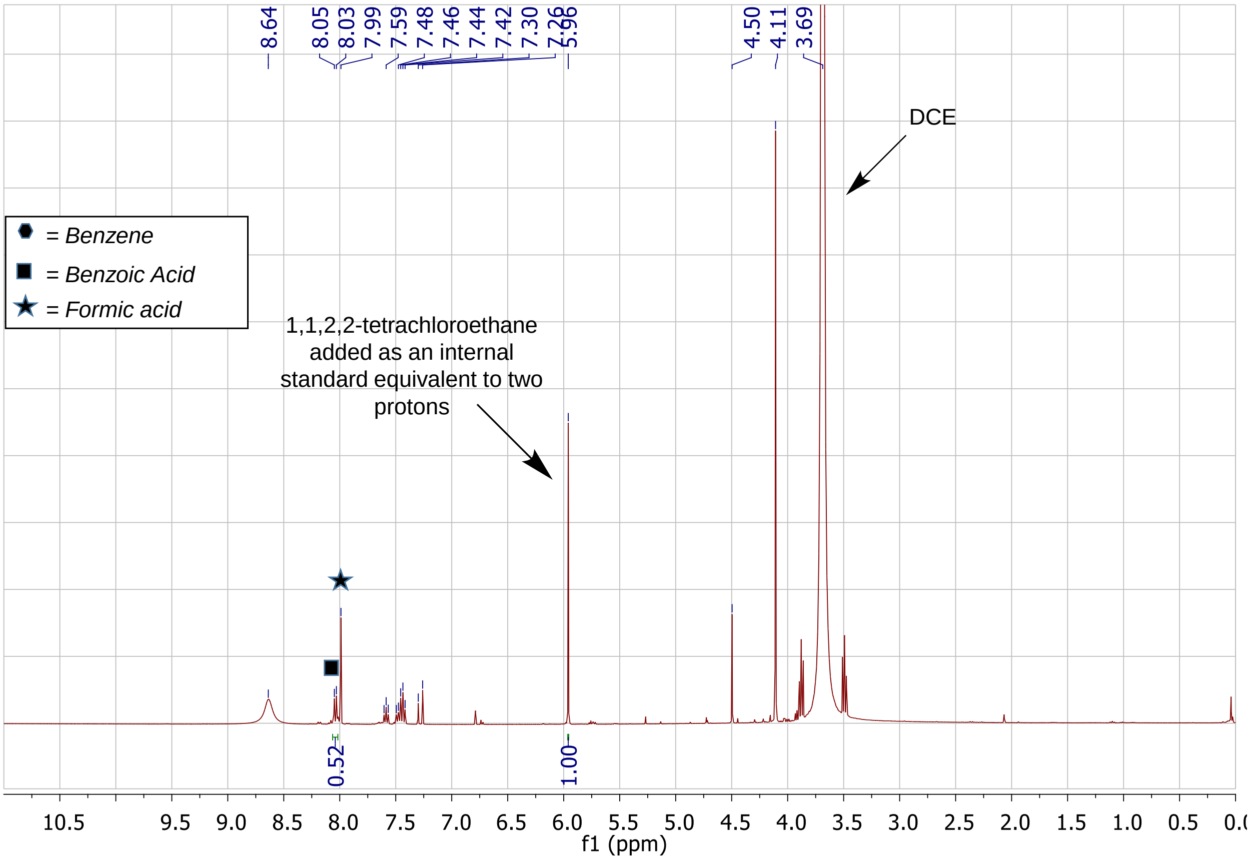

Figure S121: ^{1}H NMR spectrum of the styrene oxidation reaction in $CDCl_3$using $BA_2CsAg_{0.75}Na_{0.25}BiBr_7$ as a photocatalyst in DCE as a solvent with a 50 W blue LED as the light source.

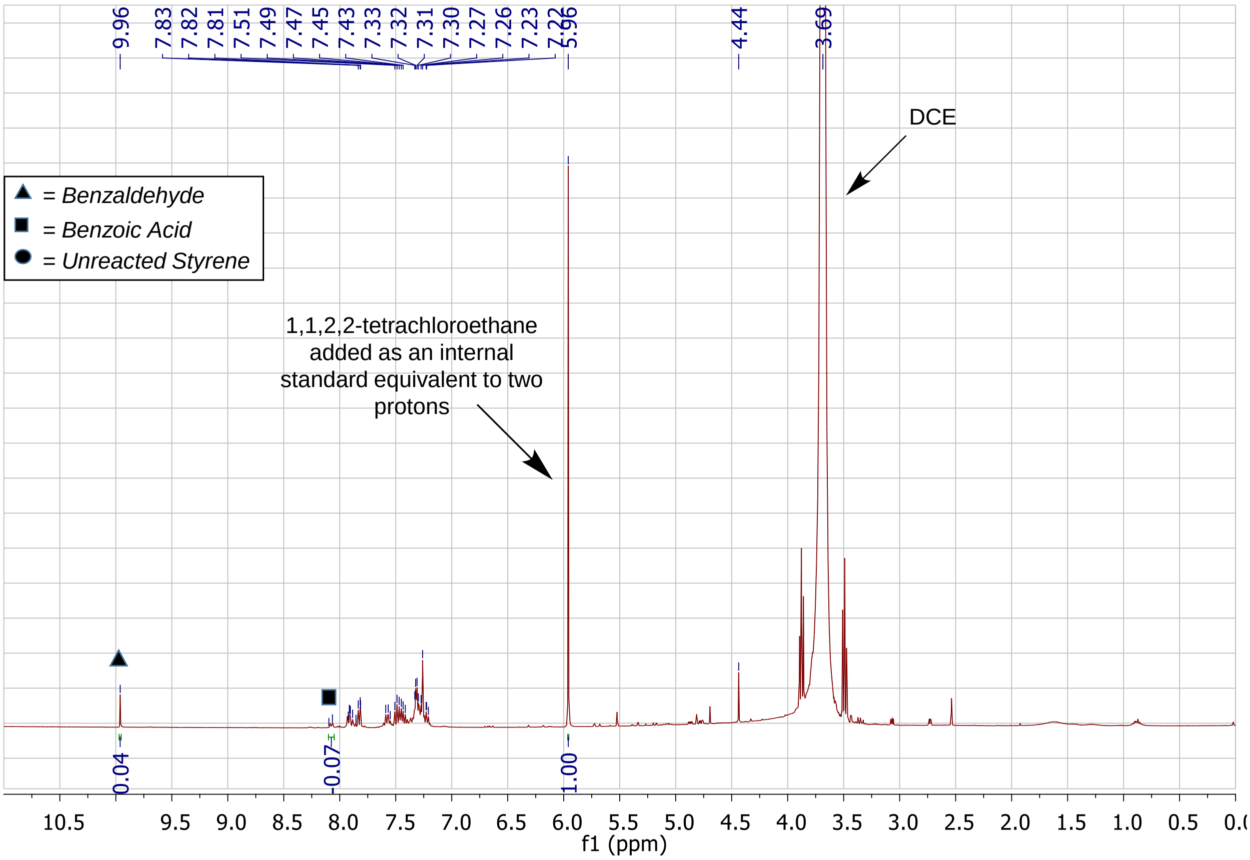

Figure S122: ^{1}H NMR spectrum of the styrene oxidation reaction in $CDCl_3$ using $BA_2CsAgBiBr_5I_2$ as a photocatalyst in DCE as a solvent with a 50 W blue LED as the light source.

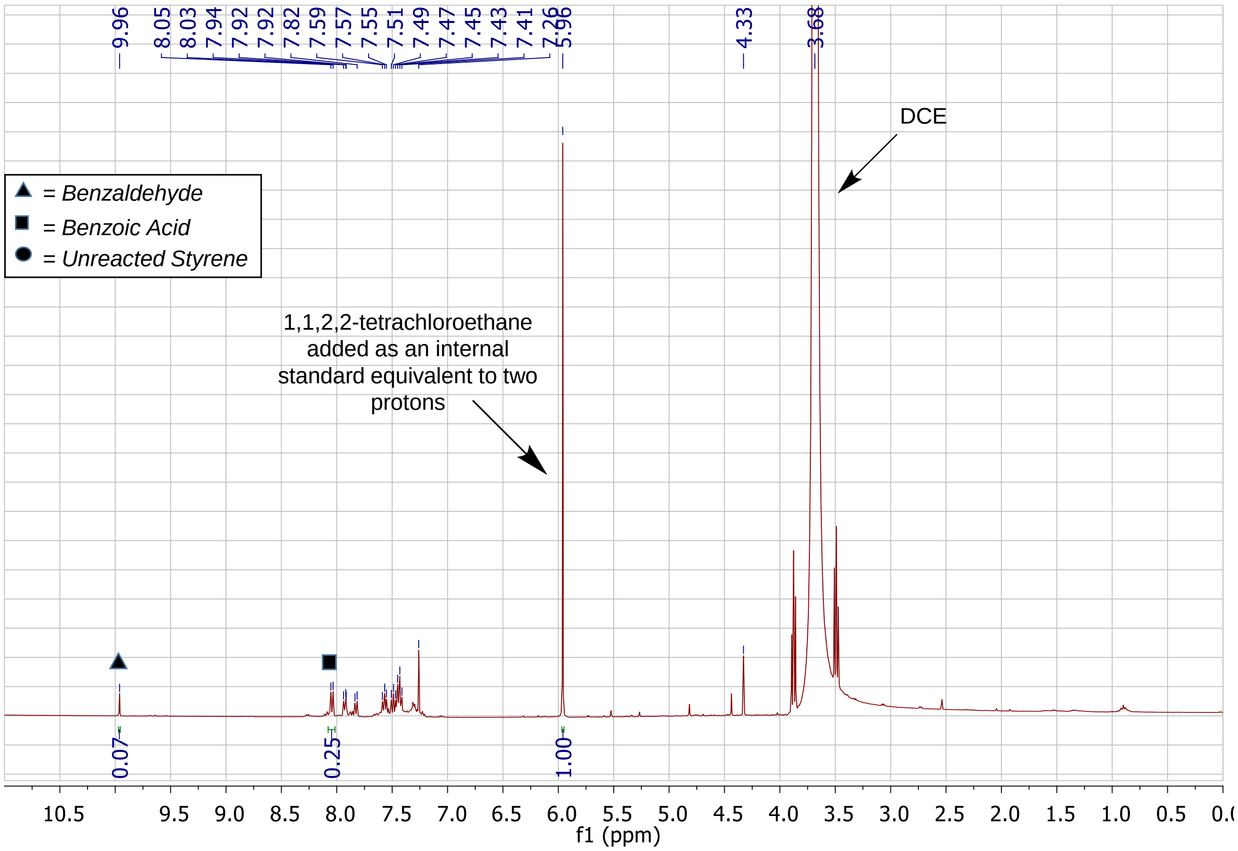

Figure S123: ^{1}H NMR spectrum of the styrene oxidation reaction in $CDCl_3$ using $BA_2CsAgBiBr_2I_5$ as a photocatalyst in DCE as a solvent with a 50 W blue LED as the light source.

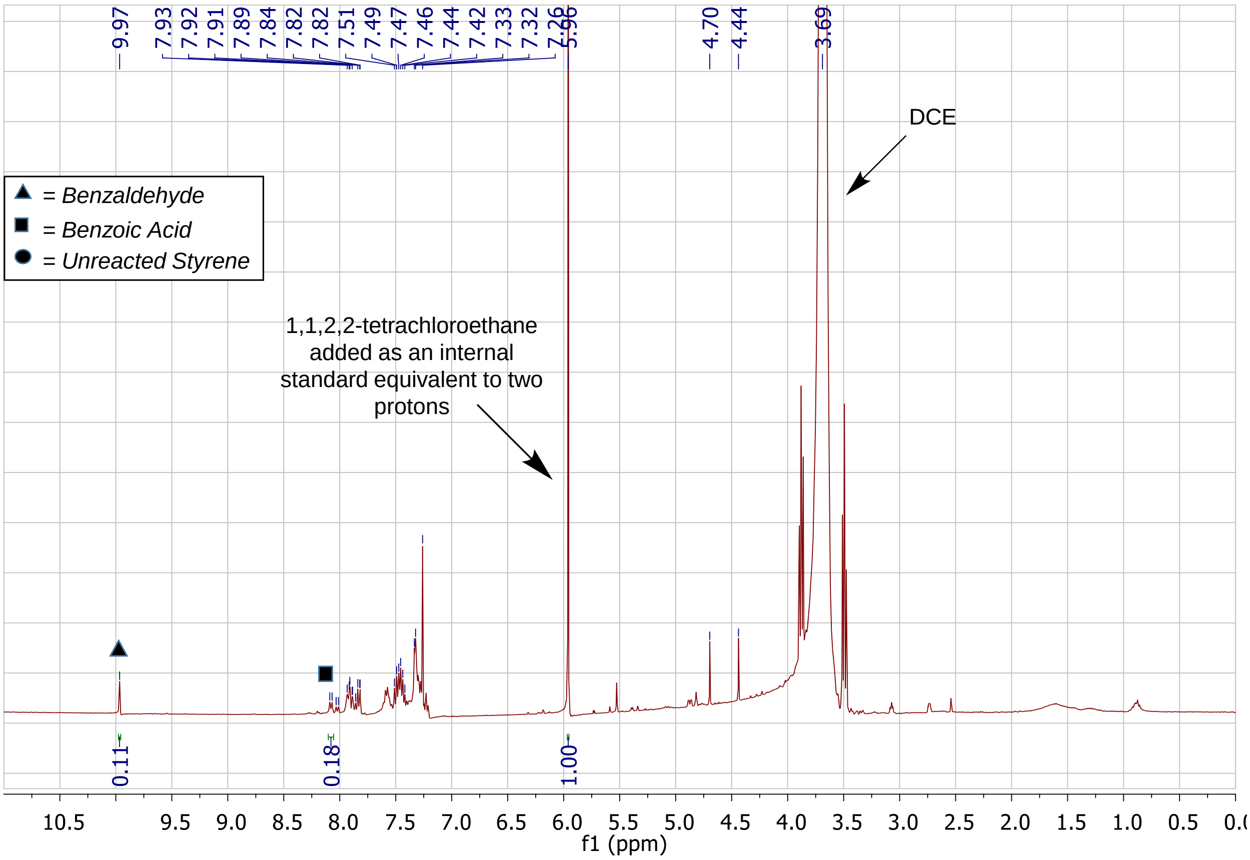

Figure S124: ^{1}H NMR spectrum of the styrene oxidation reaction in $CDCl_3$ using $BA_2CsAg_{0.95}Na_{0.05}BiBr_6I$ as a photocatalyst in DCE as a solvent with a 50 W blue LED as the light source.

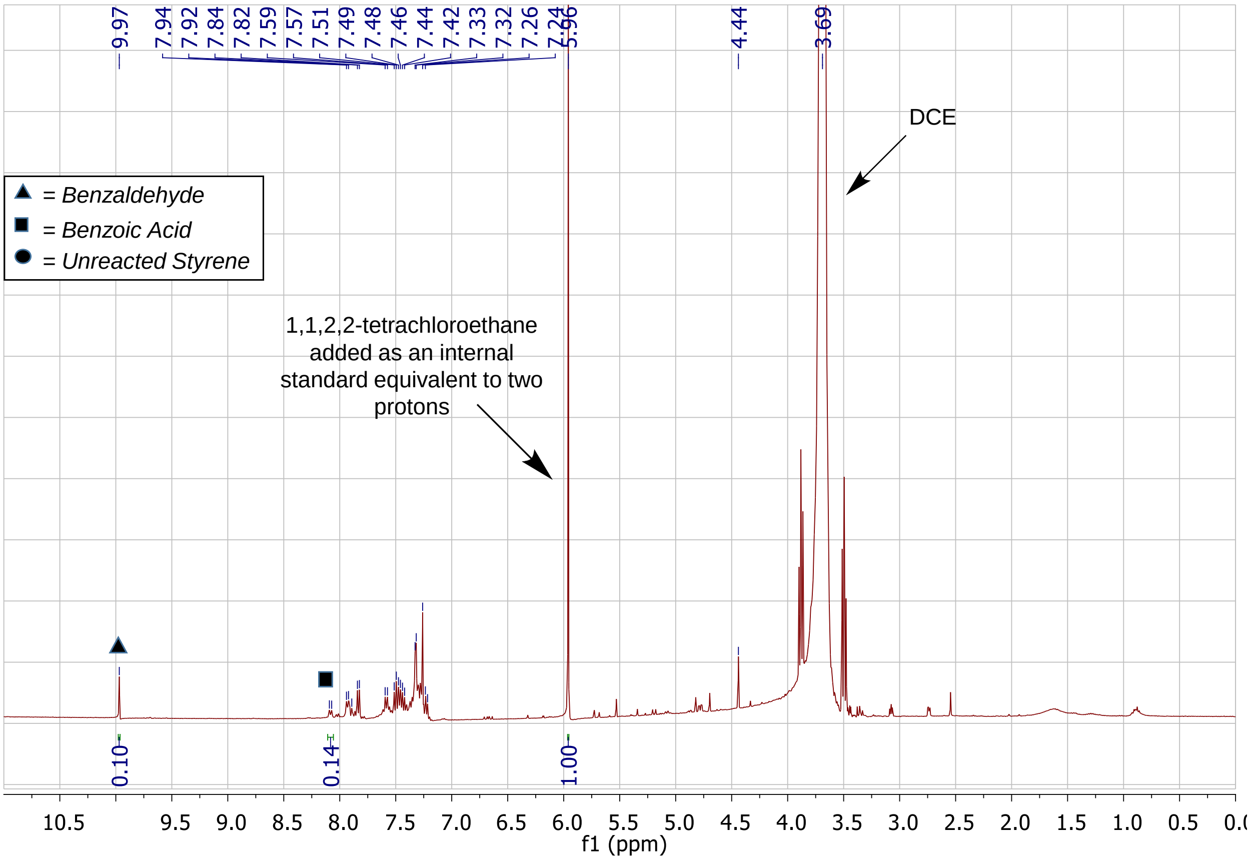

Figure S125: ^{1}H NMR spectrum of the styrene oxidation reaction in $CDCl_3$ using $BA_2CsAgBiBr_4I_3$ as a photocatalyst in DCE as a solvent with a 50 W blue LED as the light source.

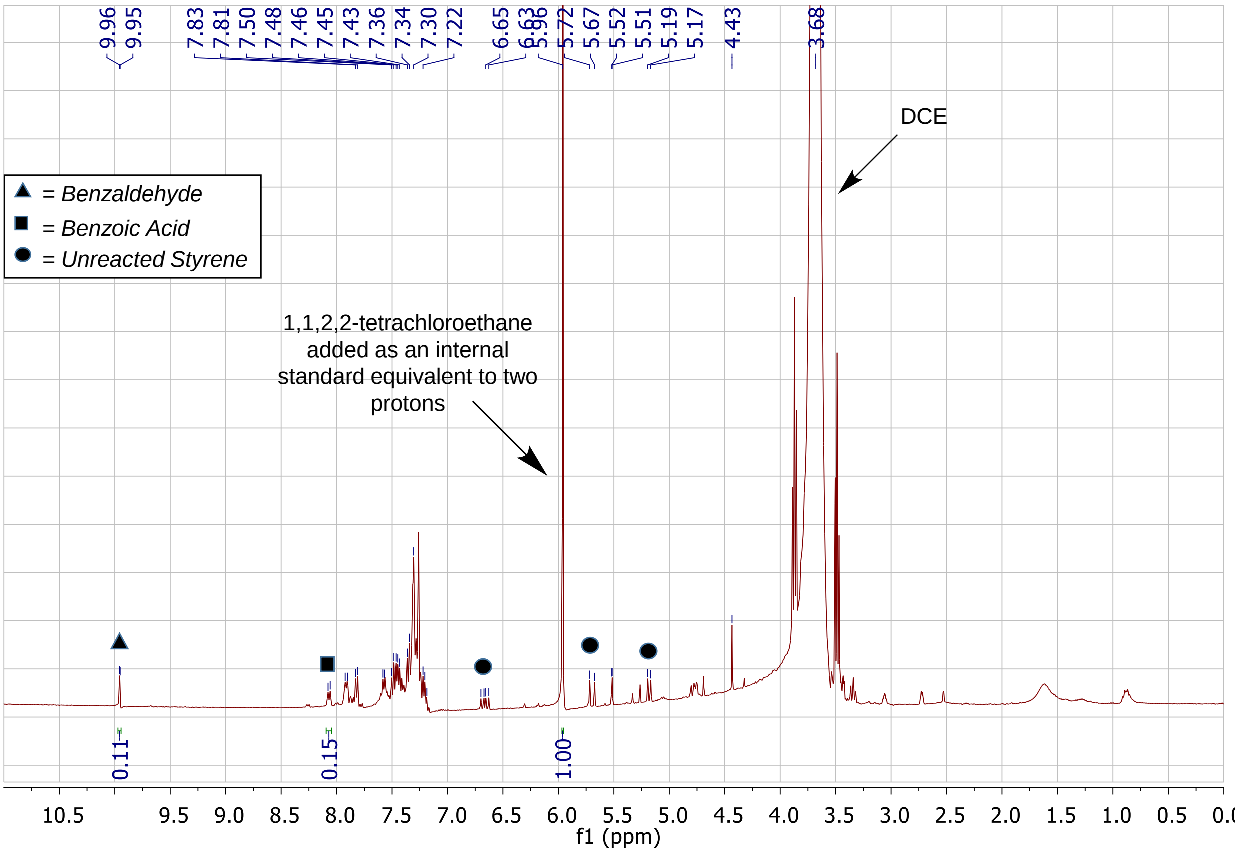

Figure S126: ^{1}H NMR spectrum of the styrene oxidation reaction in $CDCl_3$ using $BA_2CsAgBiBr_3I_4$ as a photocatalyst in DCE as a solvent with a 50 W blue LED as the light source.

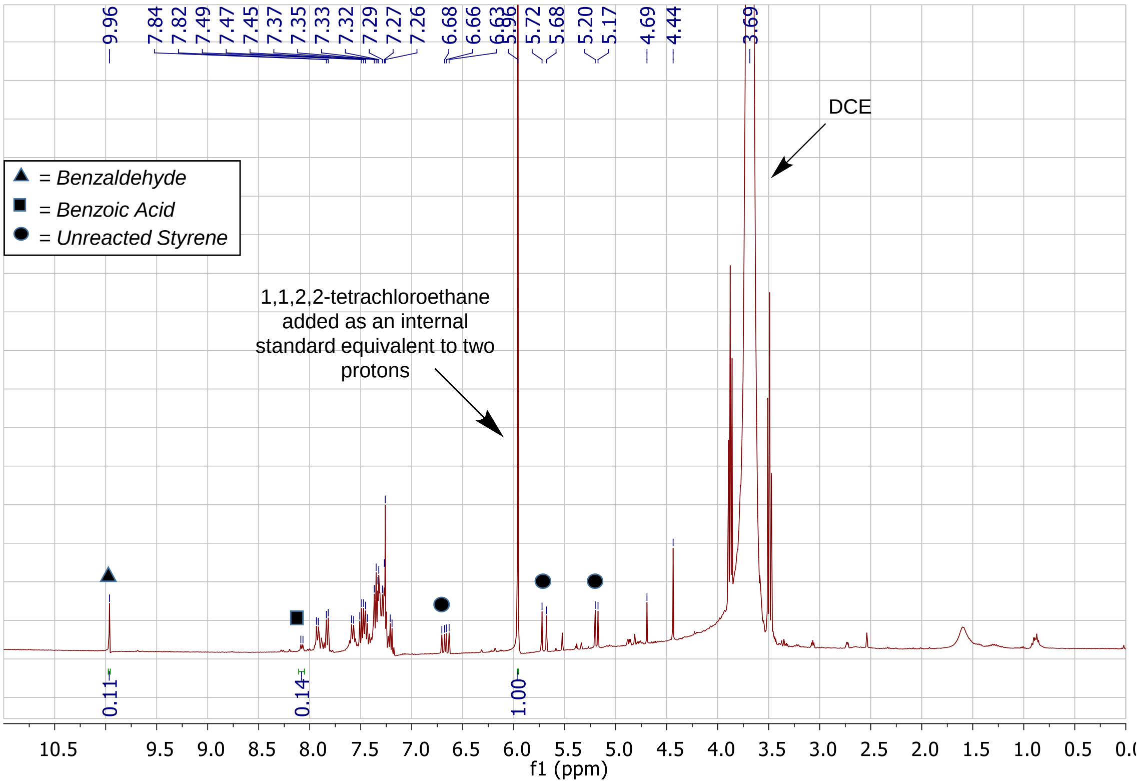

Figure S127: ^{1}H NMR spectrum of the styrene oxidation reaction in $CDCl_3$ using $BA_2CsAgBiBr_6I$ as a photocatalyst in DCE as a solvent with a 50 W blue LED as the light source.

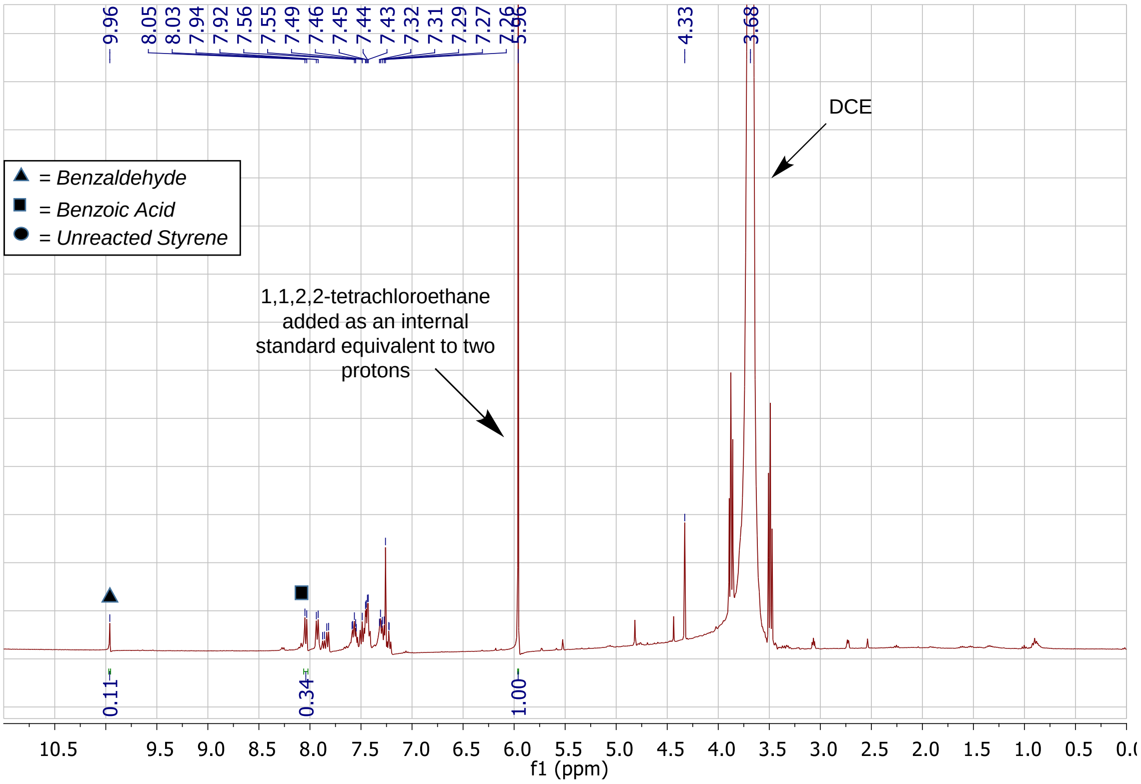

Figure S128: ^{1}H NMR spectrum of the styrene oxidation reaction in $CDCl_3$ using $BA_2CsAgBiBrI_6$ as a photocatalyst in DCE as a solvent with a 50 W blue LED as the light source.

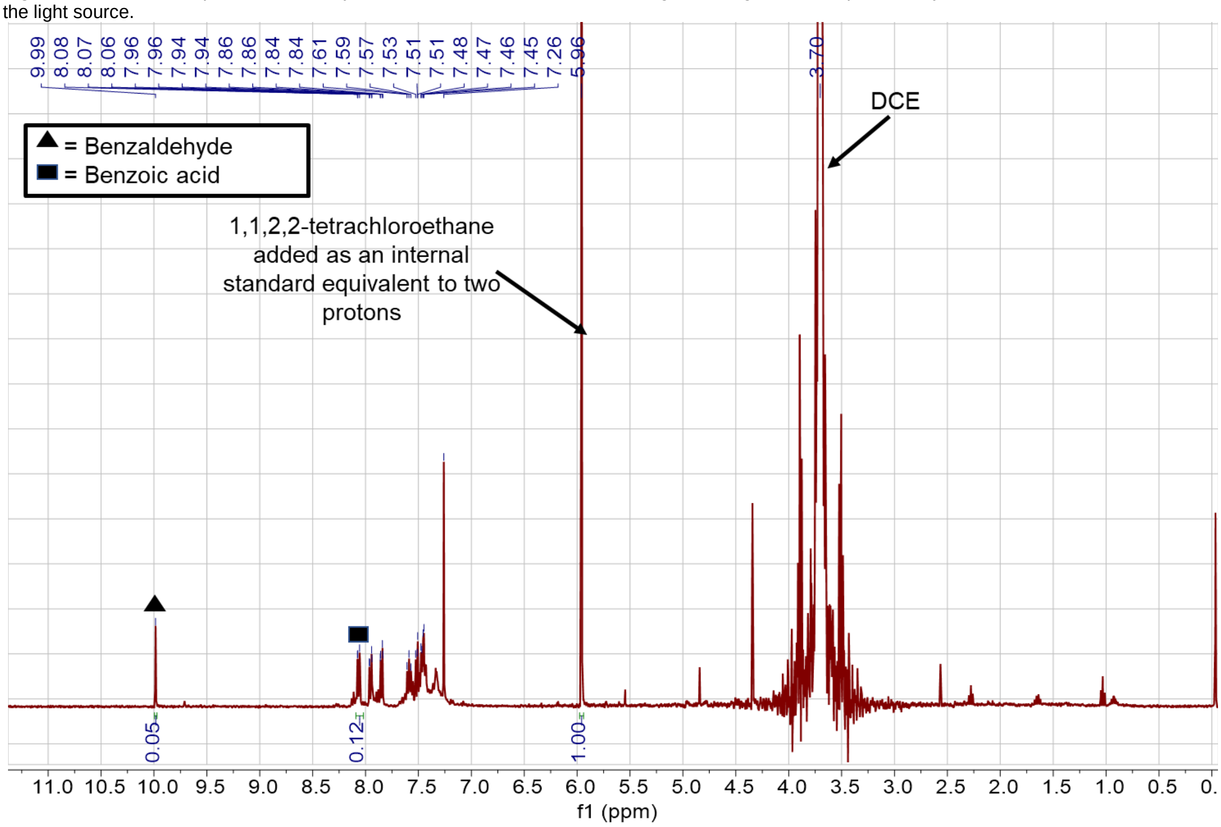

Figure S129: ^{1}H NMR spectrum of the styrene oxidation reaction in $CDCl_3$ using $BA_2CsAgBiI_7$ as a photocatalyst in DCE as a solvent with a 50 W blue LED as the light source.

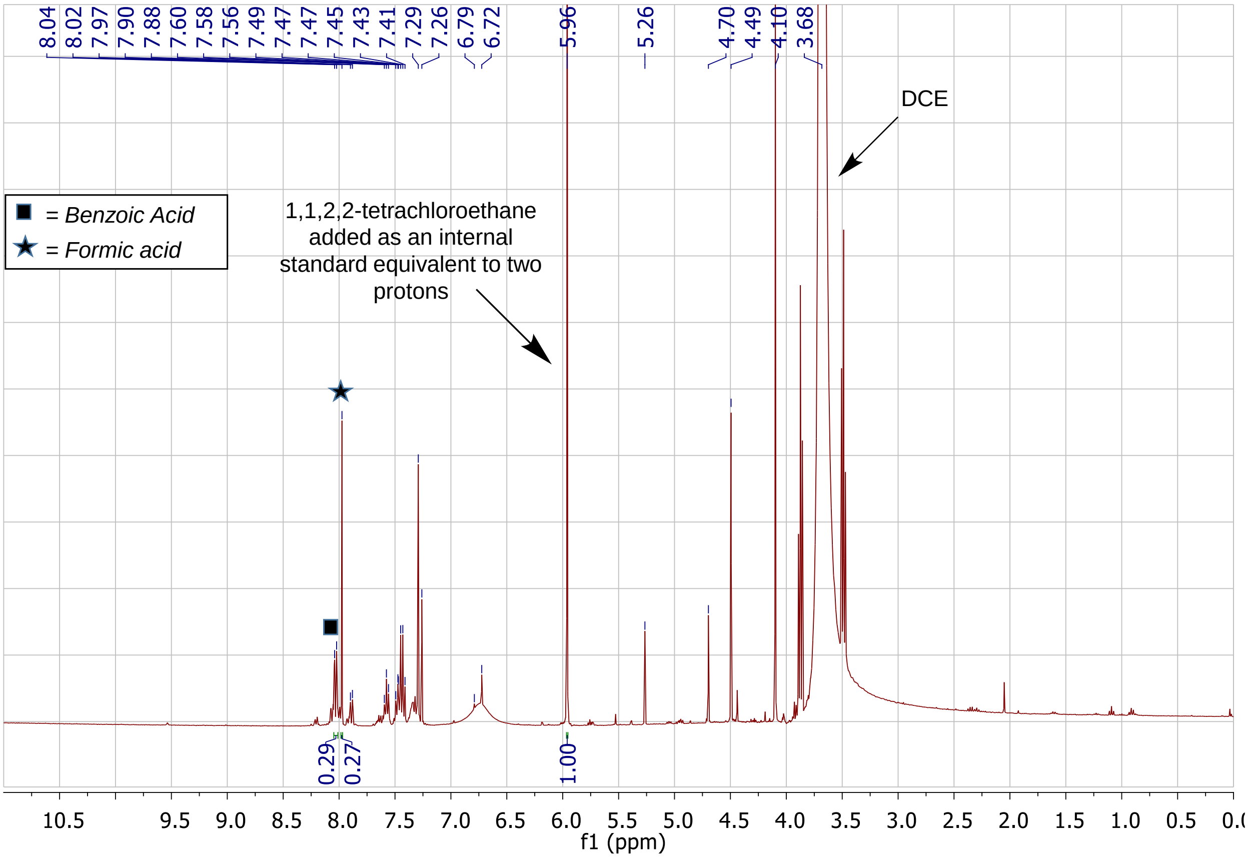

Figure S130: ^{1}H NMR spectrum of the styrene oxidation reaction in $CDCl_3$ using $BA_2CsAg_{0.95}Na_{0.05}BiCl_6Br$ as a photocatalyst in DCE as a solvent with a 50 W blue LED as the light source.

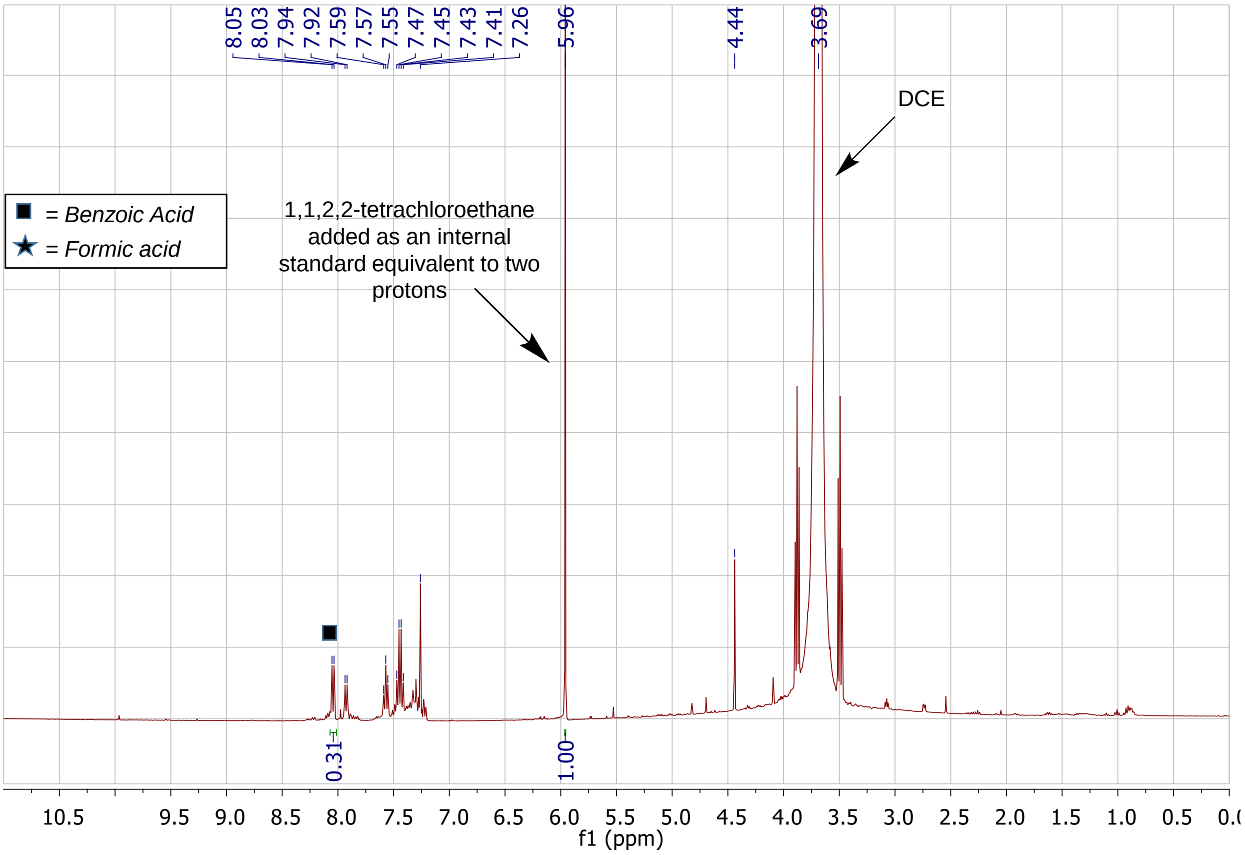

Figure S131: ^{1}H NMR spectrum of the styrene oxidation reaction in $CDCl_3$ using $BA_2CsAg_{0.95}K_{0.05}BiBr_7$ as a photocatalyst in DCE as a solvent with a 50 W blue LED as the light source.

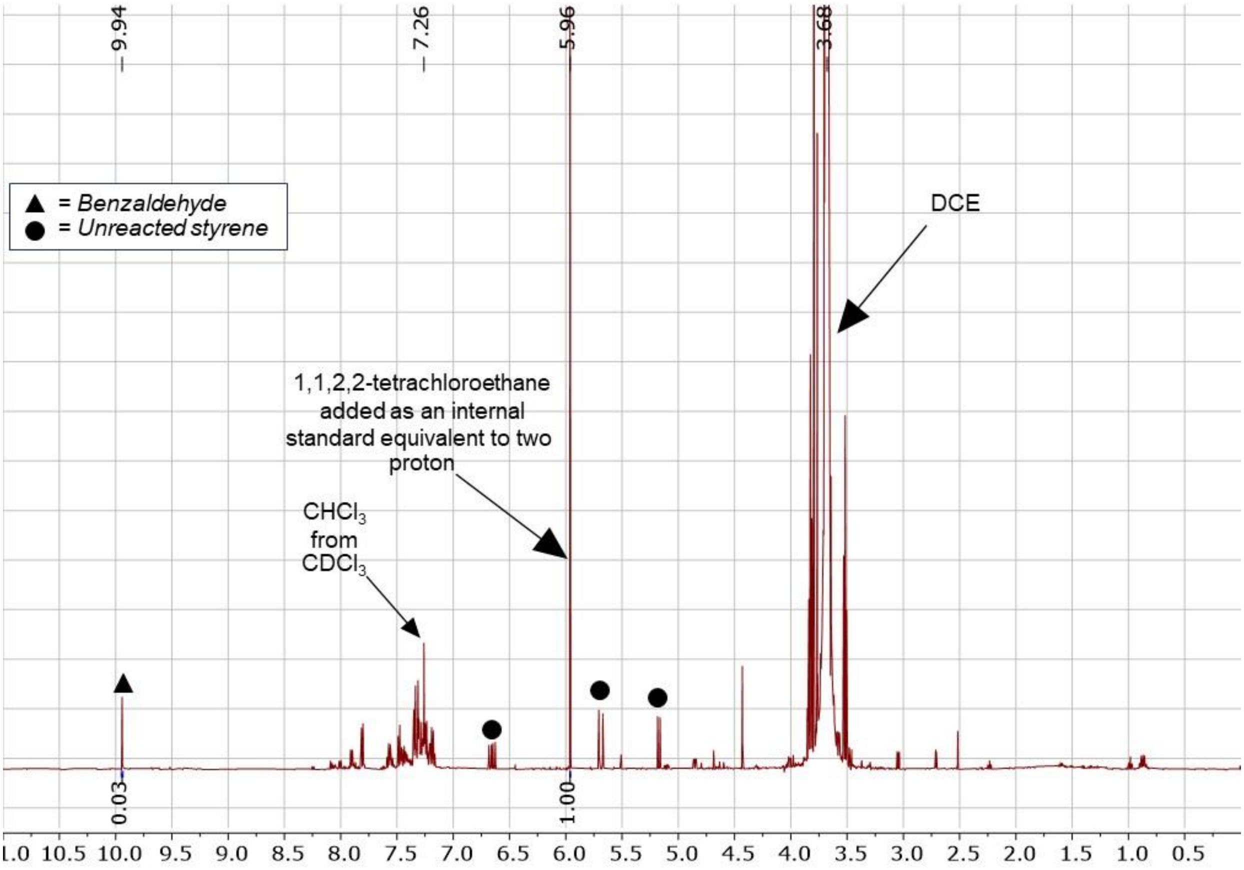

Figure S132: ^{1}H NMR spectrum of the styrene oxidation reaction in $CDCl_3$ using $BA_2CsAg_{0.95}Cu_{0.05}BiBr_7$ as a photocatalyst in DCE as a solvent with a 50 W blue LED as the light source.

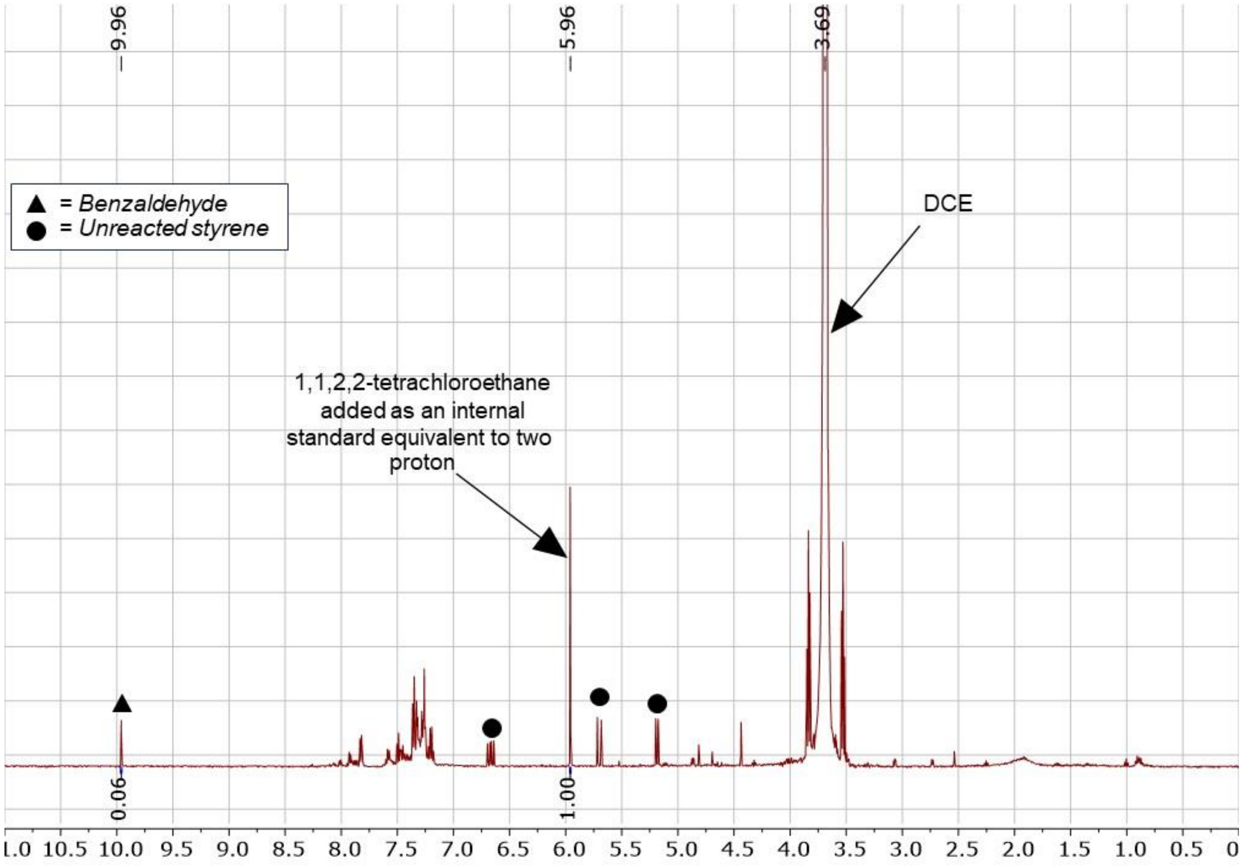

Figure S133: ^{1}H NMR spectrum of the styrene oxidation reaction in $CDCl_3$ using $BA_2CsAg_{0.95}Li_{0.05}BiBr_7$ as a photocatalyst in DCE as a solvent with a 50 W blue LED as the light source.

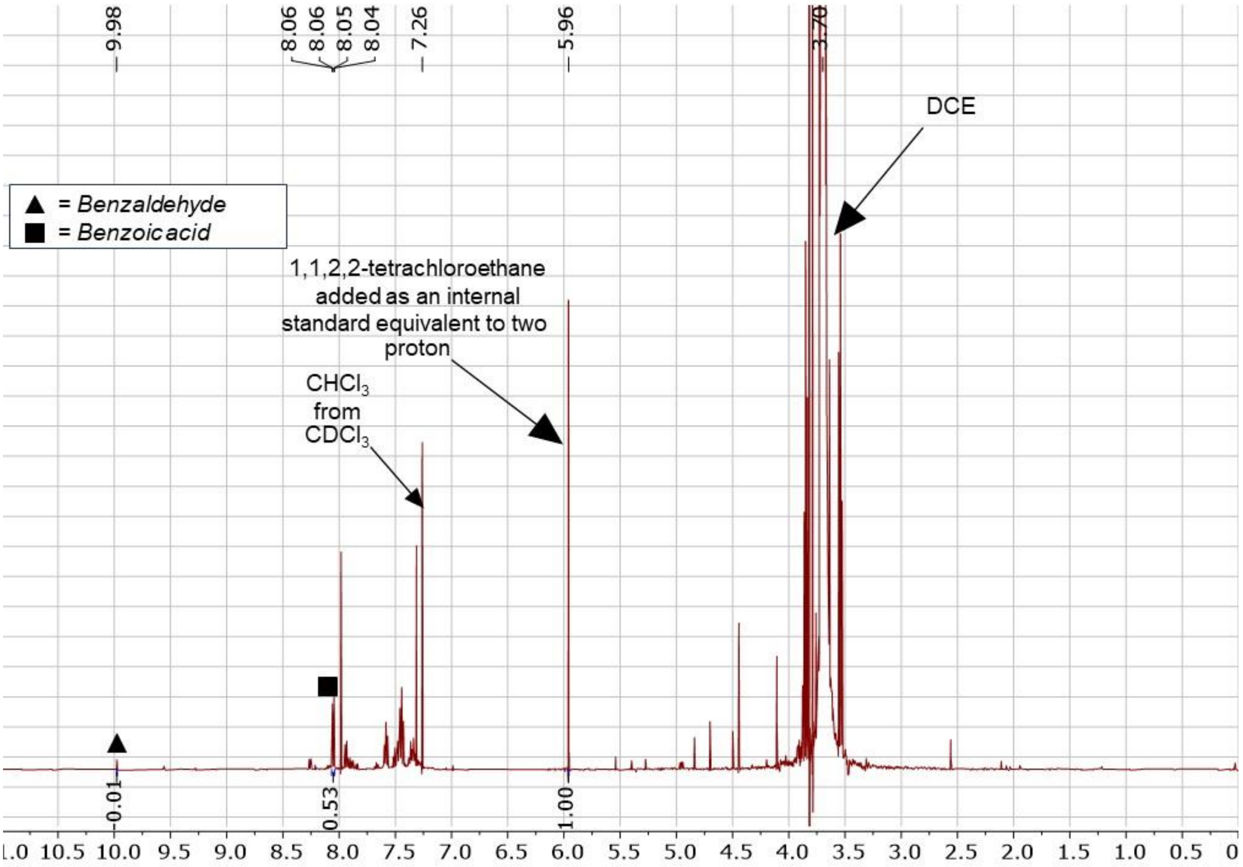

Figure S134: ^{1}H NMR spectrum of the styrene oxidation reaction in $CDCl_3$ using $PEA_2CsAgBiBr_7$ as a photocatalyst in DCE as a solvent with a 50 W blue LED as the light source.

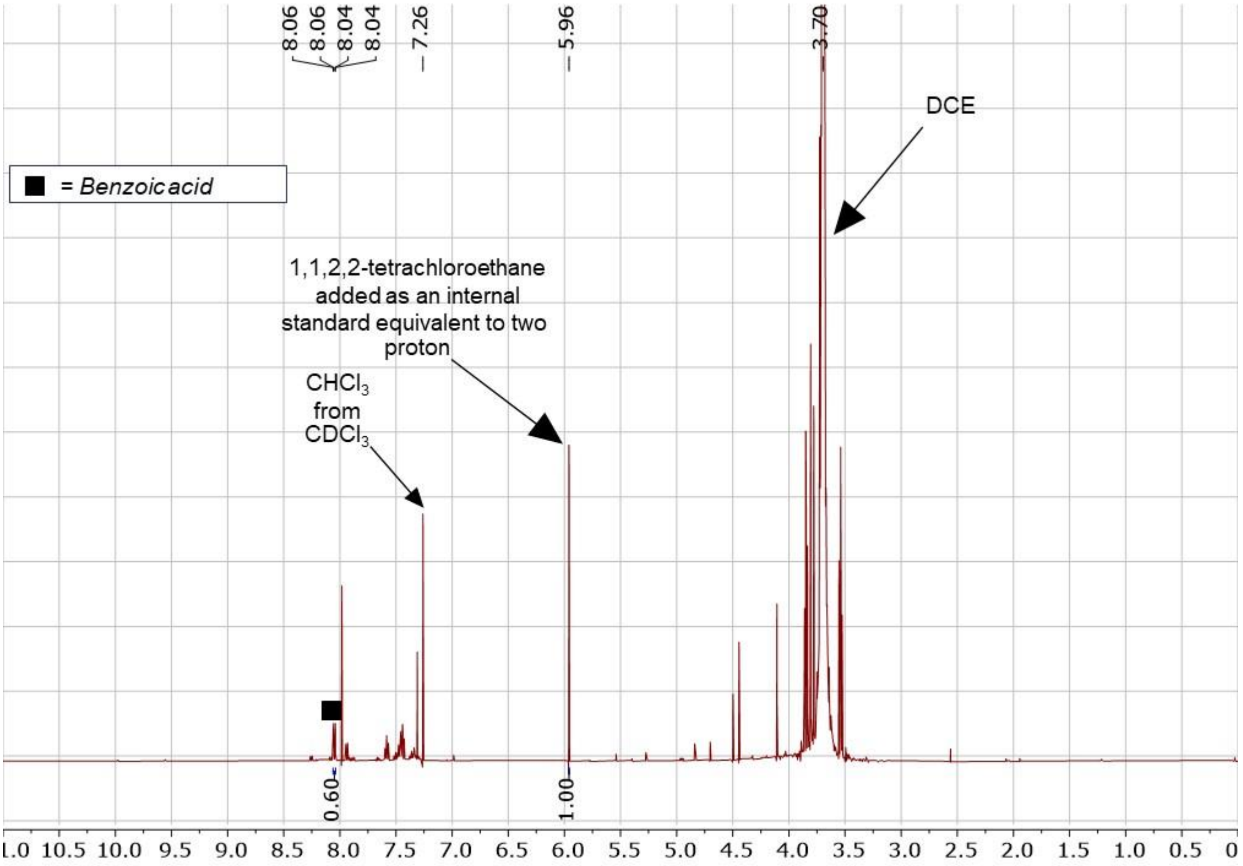

Figure S135: ^{1}H NMR spectrum of the styrene oxidation reaction in $CDCl_3$ using $PEA_2CsAg_{0.95}Na_{0.05}BiBr_7$ as a photocatalyst in DCE as a solvent with a 50 W blue LED as the light source.

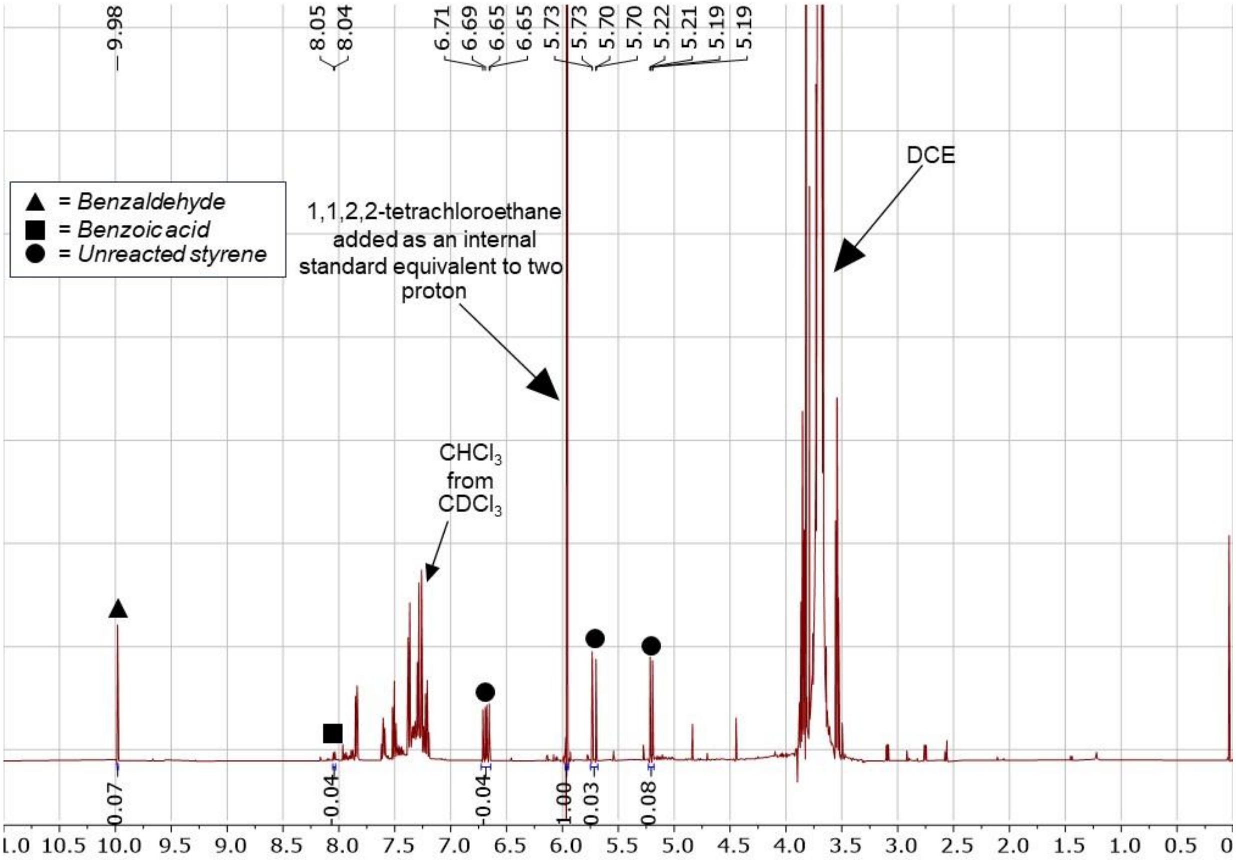

Figure S136: ^{1}H NMR spectrum of the styrene oxidation reaction in $CDCl_3$ using $PEA_2CsAg_{0.95}Na_{0.05}BiBr_7$, which had been collected by centrifugation after one round of reaction and reused in a second round as a photocatalyst, in DCE as a solvent with a 50 W blue LED as the light source.

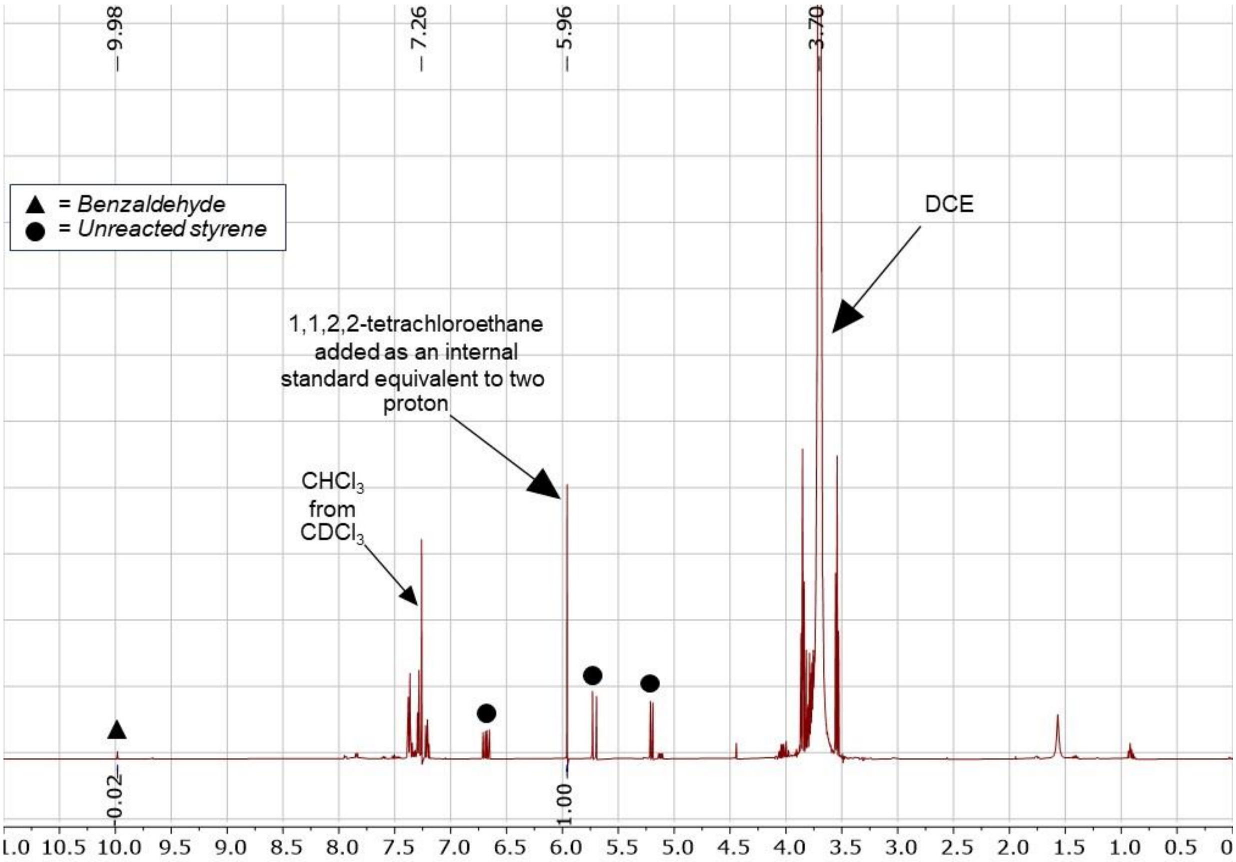

Figure S137: ^{1}H NMR spectrum of the styrene oxidation reaction in $CDCl_3$ using $BA_2CsAgSbBr_7$ as a photocatalyst in DCE as a solvent with a 50 W blue LED as the light source.

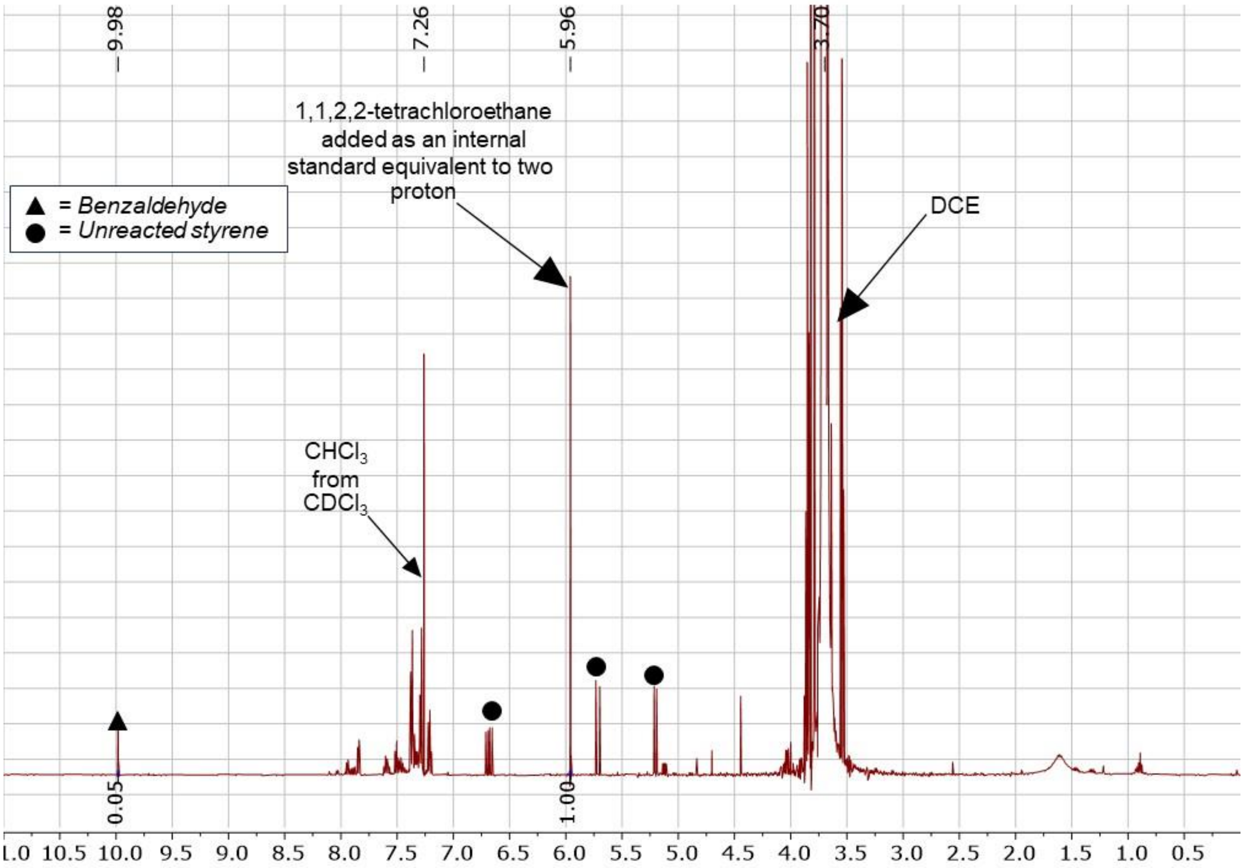

Figure S138: ^{1}H NMR spectrum of the styrene oxidation reaction in $CDCl_3$ using $BA_2CsAg_{0.95}Na_{0.05}SbBr_7$ as a photocatalyst in DCE as a solvent with a 50 W blue LED as the light source.

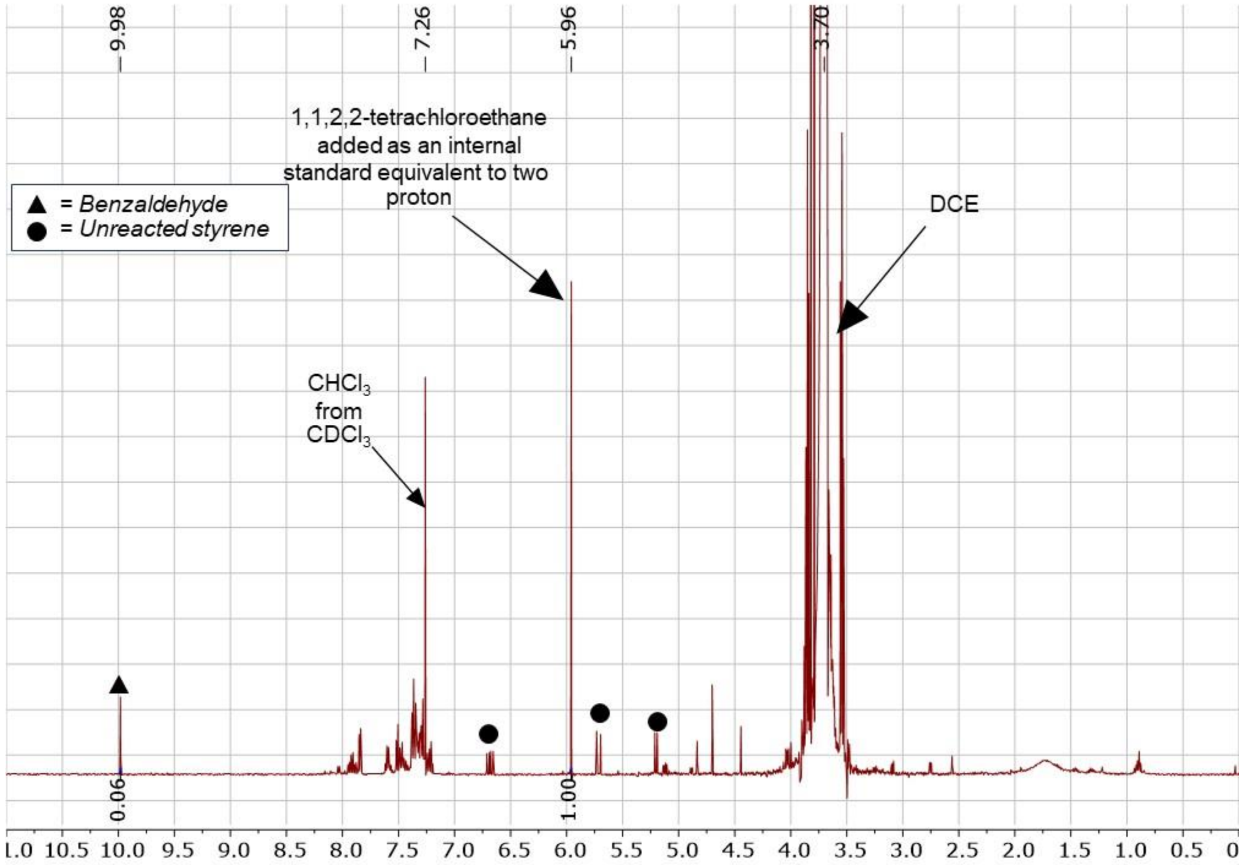

Figure S139: ^{1}H NMR spectrum of the styrene oxidation reaction in $CDCl_3$ using $BA_2CsAg_{0.9}Na_{0.1}SbBr_7$ as a photocatalyst in DCE as a solvent with a 50 W blue LED as the light source.

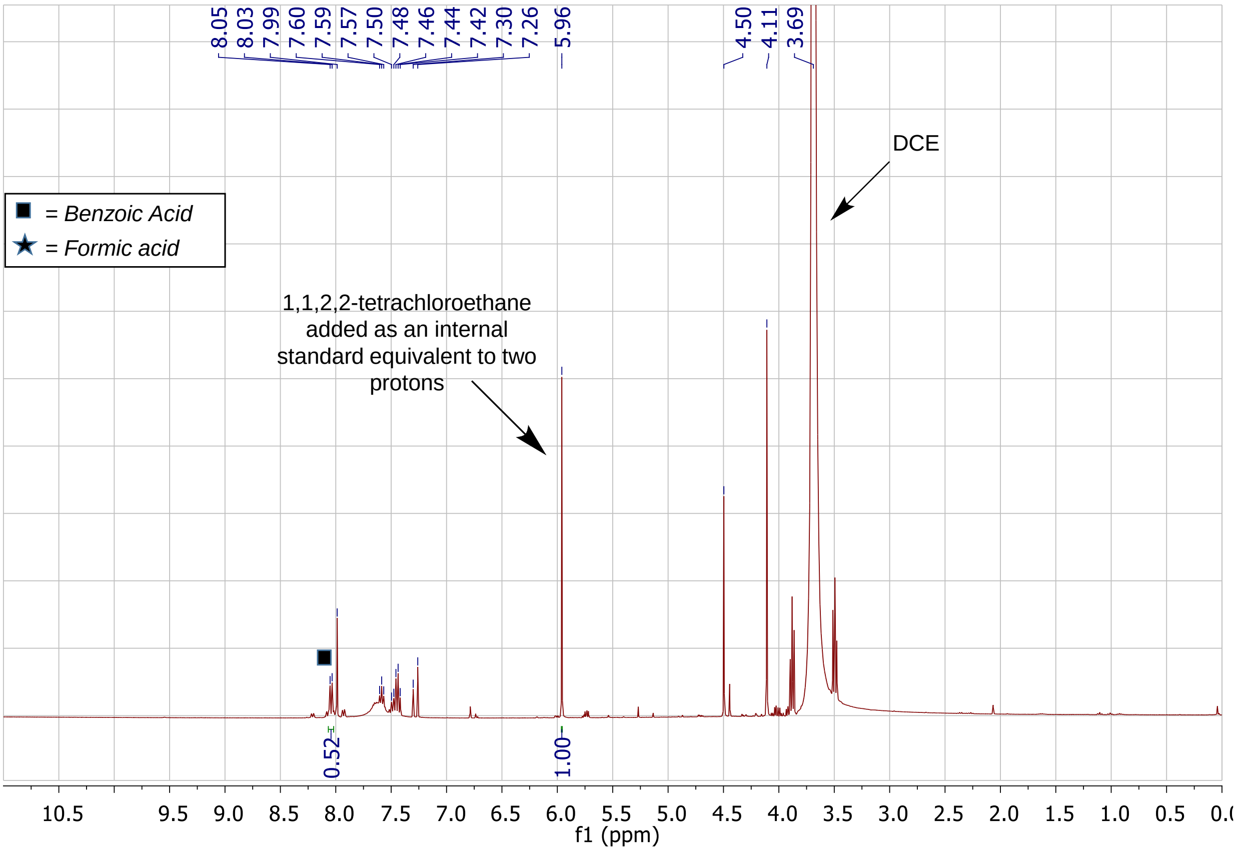

Figure S140: ^{1}H NMR spectrum of the styrene oxidation reaction in $CDCl_3$ using $BA_{1.5}Cs_{1.5}Ag_{0.95}Na_{0.05}BiBr_7$ as a photocatalyst in DCE as a solvent with a 50 W blue LED as the light source.

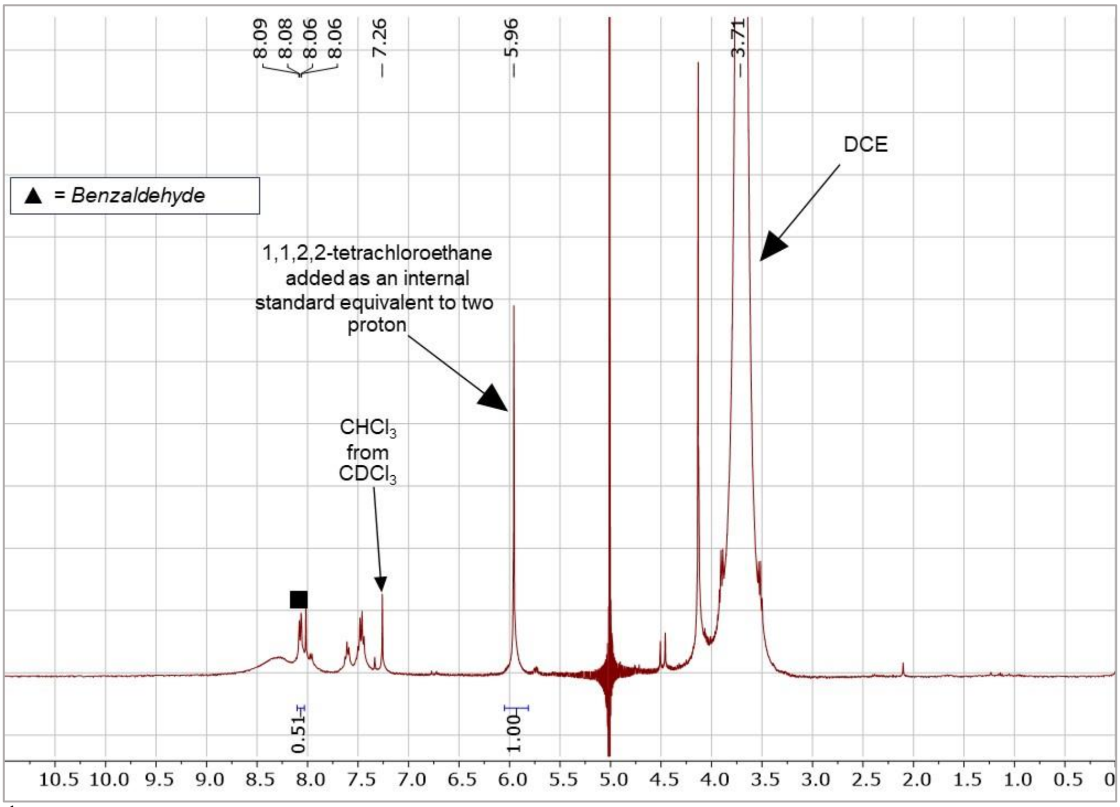

Figure S141: ^{1}H NMR spectrum of the mechanistic study of styrene oxidation reaction in $CDCl_3$ in $BA_2CsAg_{0.95}Na_{0.05}BiBr_7$ as a photocatalyst, ammonium oxalate as a hole scavenger, DCE as a solvent, with a 50 W blue LED as the light source.

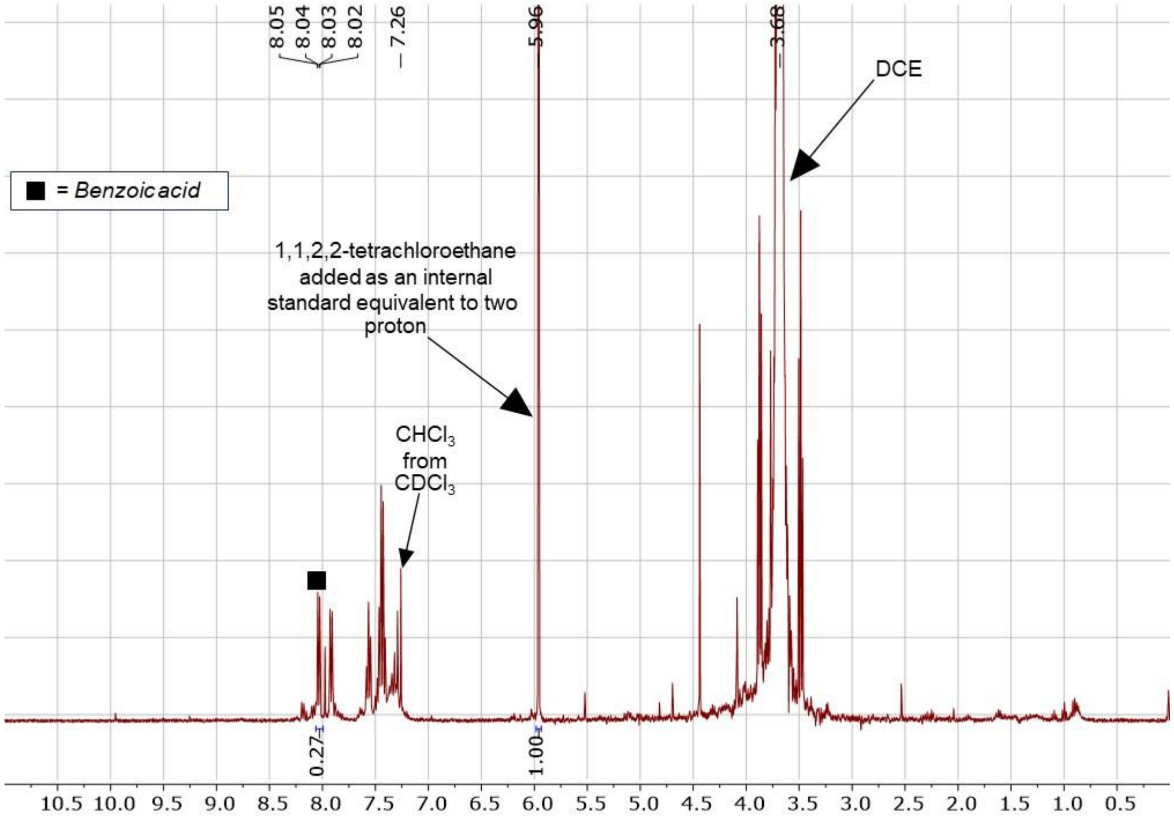

Figure S142: ^{1}H NMR spectrum of the mechanistic study of styrene oxidation reaction in $CDCl_3$ using $BA_2CsAg_{0.95}Na_{0.05}BiBr_7$ as a photocatalyst, potassium persulfate as an electron scavenger, DCE as a solvent, with a 50 W blue LED as the light source.

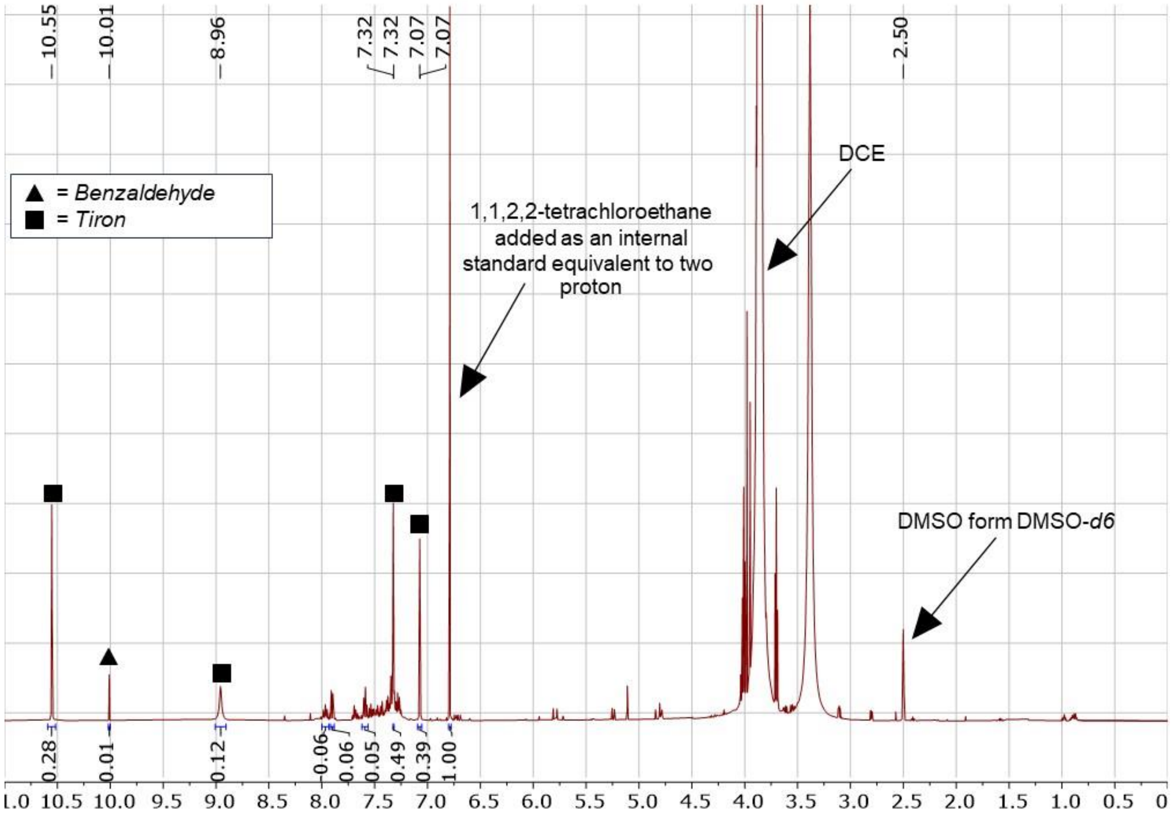

Figure S143: ^{1}H NMR spectrum of the mechanistic study of styrene oxidation reaction in DMSO-*d6* using $BA_2CsAg_{0.95}Na_{0.05}BiBr_7$ as a photocatalyst, Tiron as a superoxide scavenger, DCE as a solvent, with a 50 W blue LED as the light source.

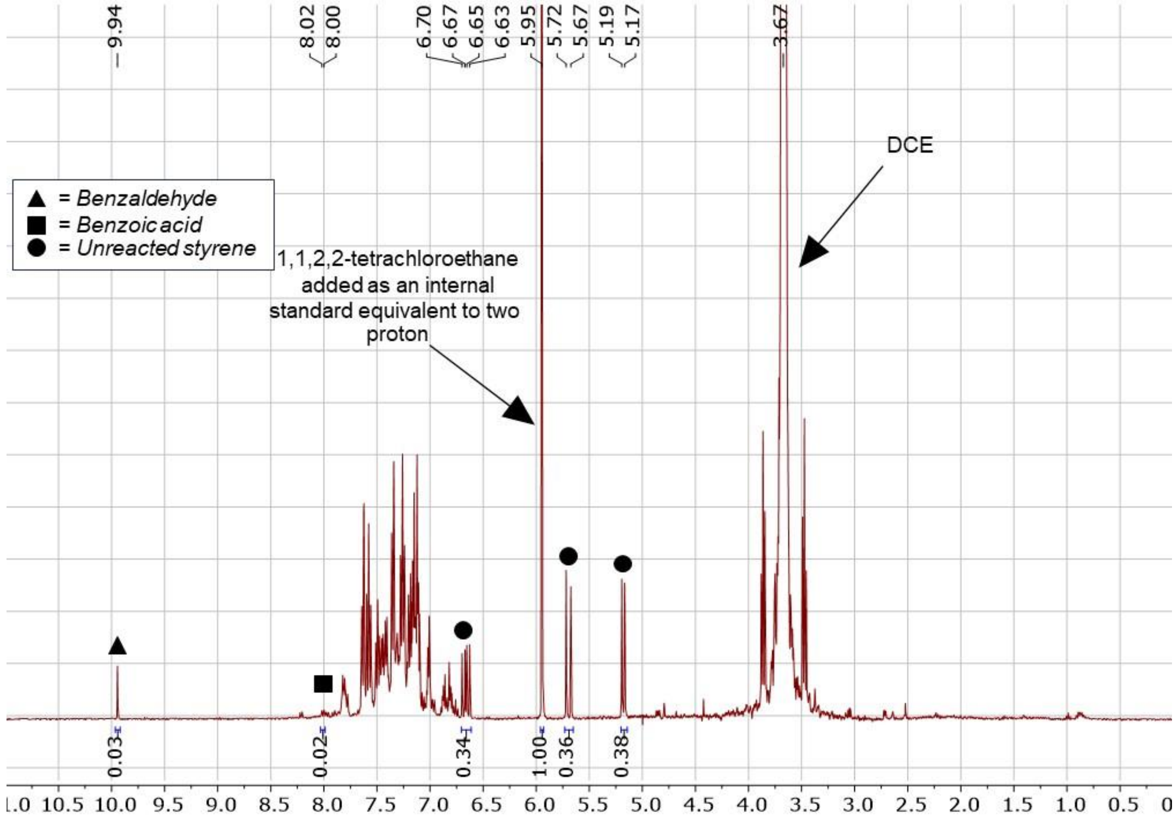

Figure S144: ^{1}H NMR spectrum of the mechanistic study of styrene oxidation reaction in $CDCl_3$ using $BA_2CsAg_{0.95}Na_{0.05}BiBr_7$ as a photocatalyst, 9,10-diphenylanthracene as a singlet oxygen scavenger, DCE as a solvent, with a 50 W blue LED as the light source.

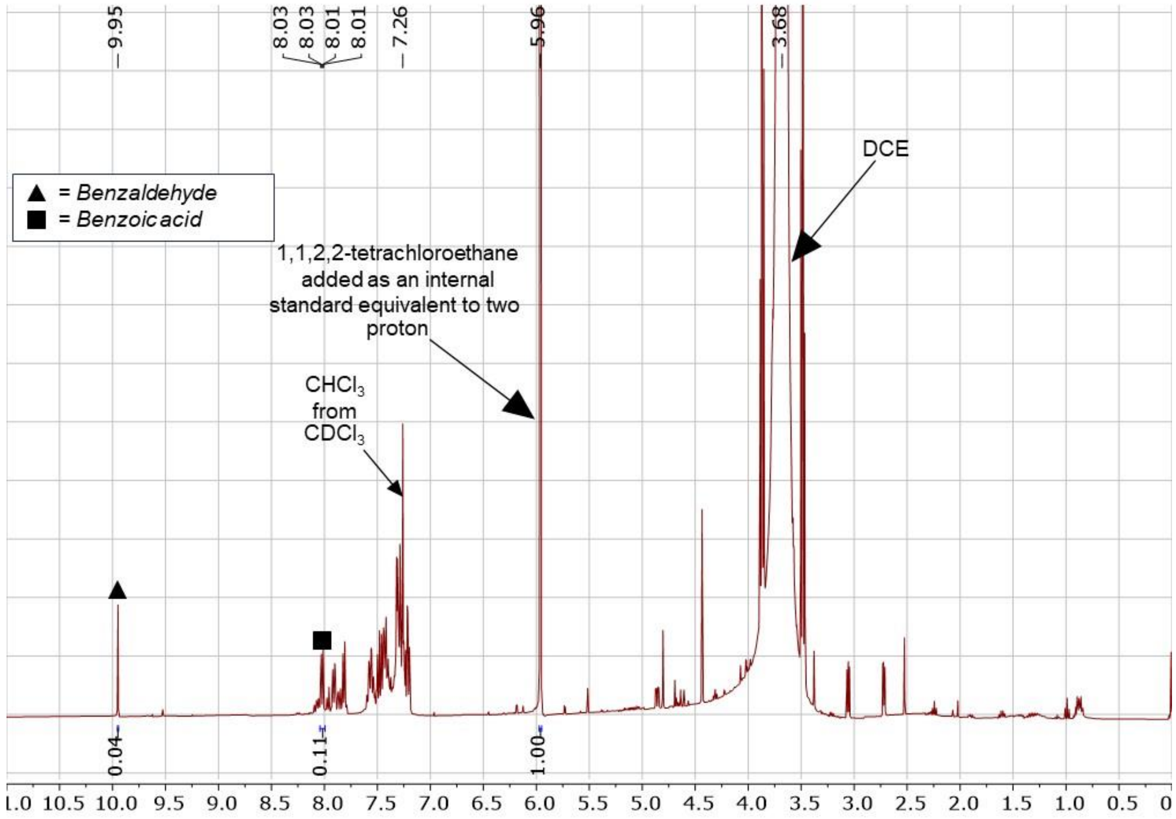

Figure S145: ^{1}H NMR spectrum of the mechanistic study of styrene oxidation reaction in $CDCl_3$ using $BA_2CsAg_{0.95}Na_{0.05}BiBr_7$ as a photocatalyst, DCE as a solvent, with a 50 W blue LED as the light source. The reaction was irradiated for one day and stirred in the dark for the next two days.

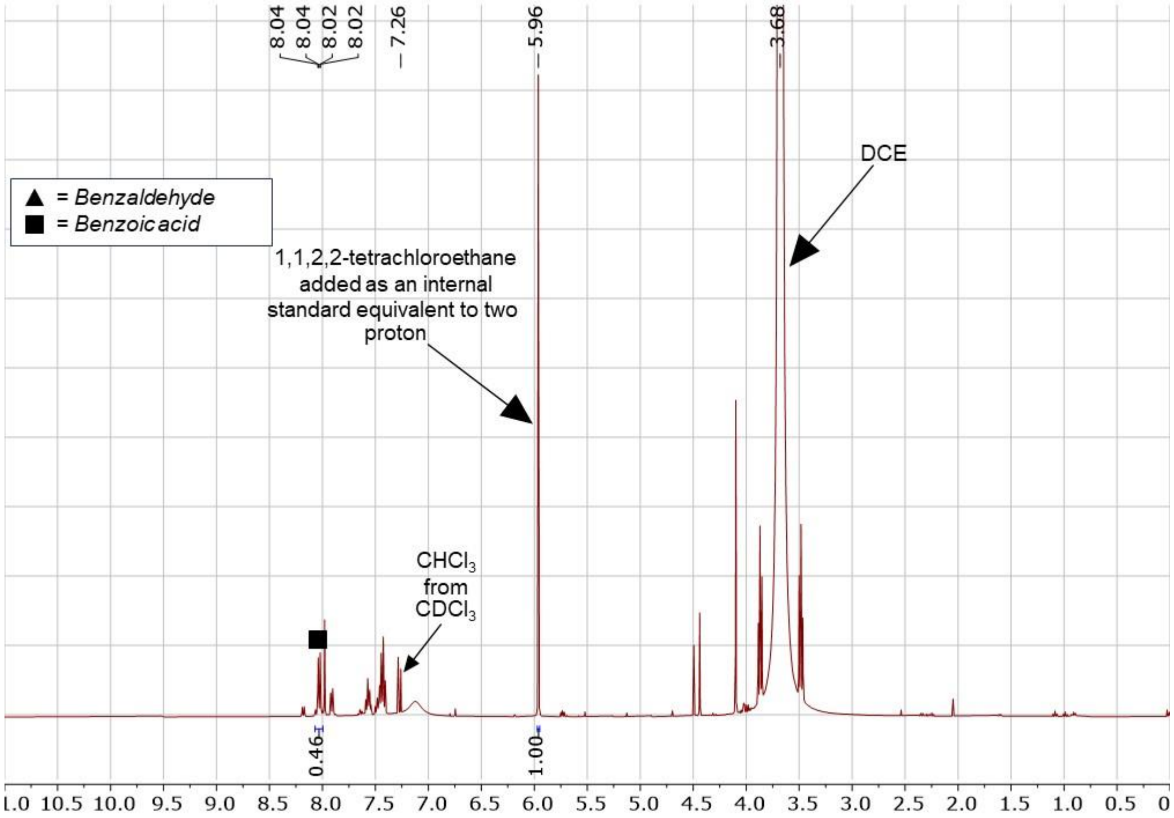

Figure S146: ^{1}H NMR spectrum of the mechanistic study of styrene oxidation reaction in $CDCl_3$ using $BA_2CsAg_{0.95}Na_{0.05}BiBr_7$ as a photocatalyst, DCE as a solvent, with a 50 W blue LED as the light source. The reaction was irradiated for two days and stirred in the dark for the next one days.

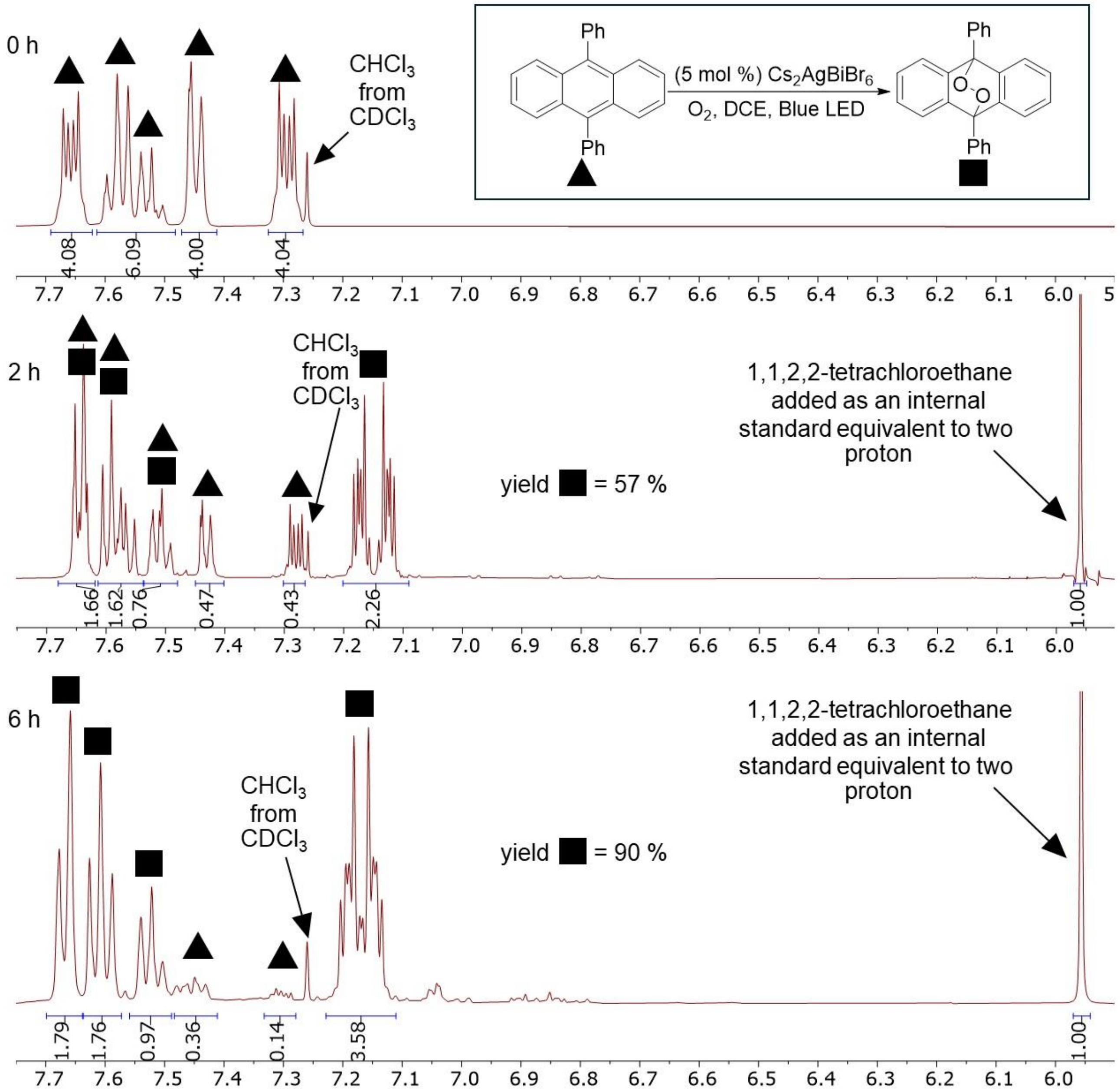

Figure S147: ^1H NMR spectra of the reactions using 9,10-diphenylanthracene to trap $^1O_2$ to provide 9,10-diphenyl-9,10-epidioxyanthracene in $CDCl_3$, which represents the $^1O_2$ producing ability of the photoexcited $Cs_2AgBiBr_6$.

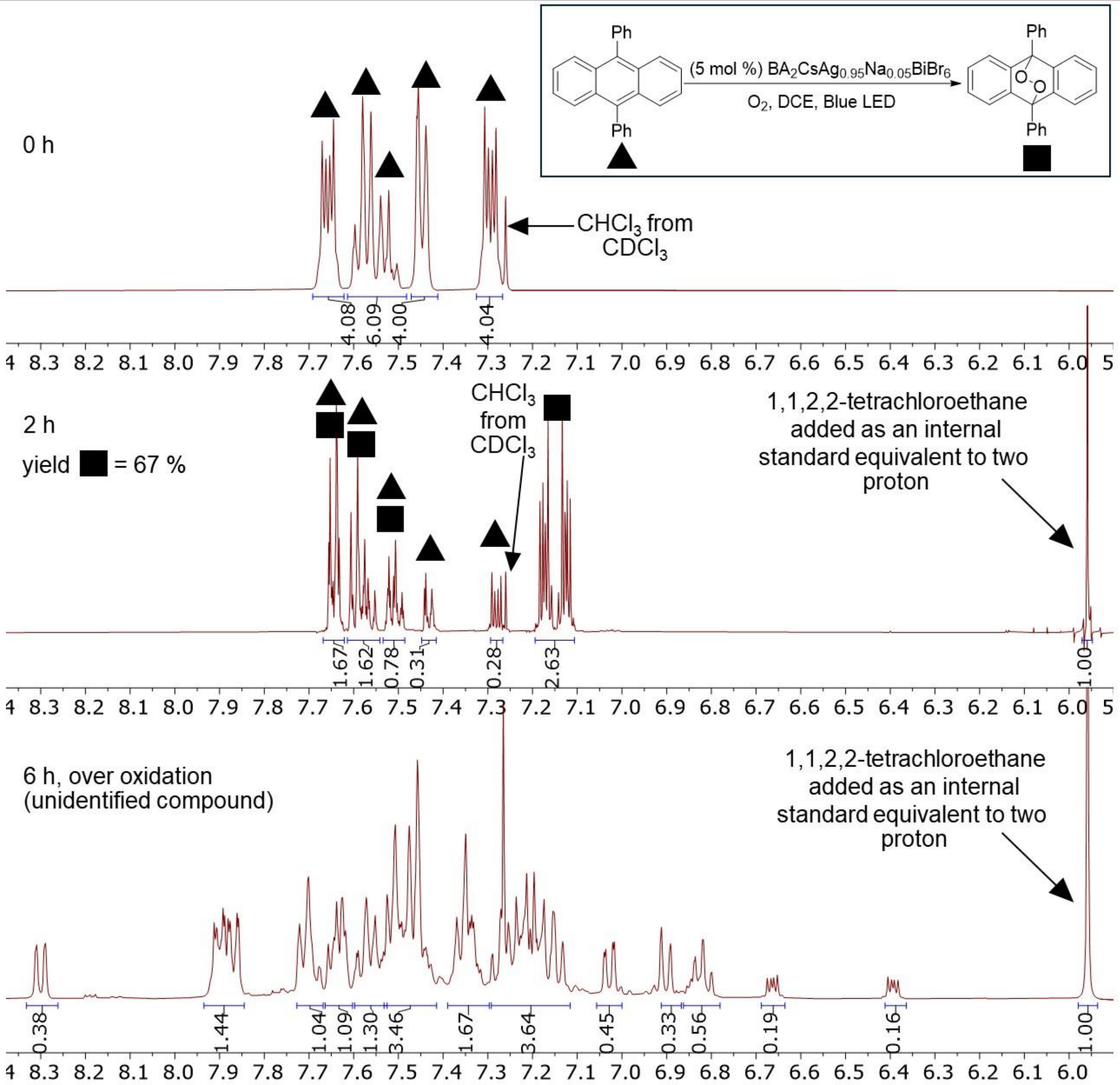

Figure S148: ^{1}H NMR spectra of the reactions using 9,10-diphenylanthracene to trap $^1O_2$ to provide 9,10-diphenyl-9,10-epidioxyanthracene in $CDCl_3$, which represents the $^1O_2$ producing ability of the photoexcited $BA_2CsAg_{0.95}Na_{0.05}BiBr_7$.

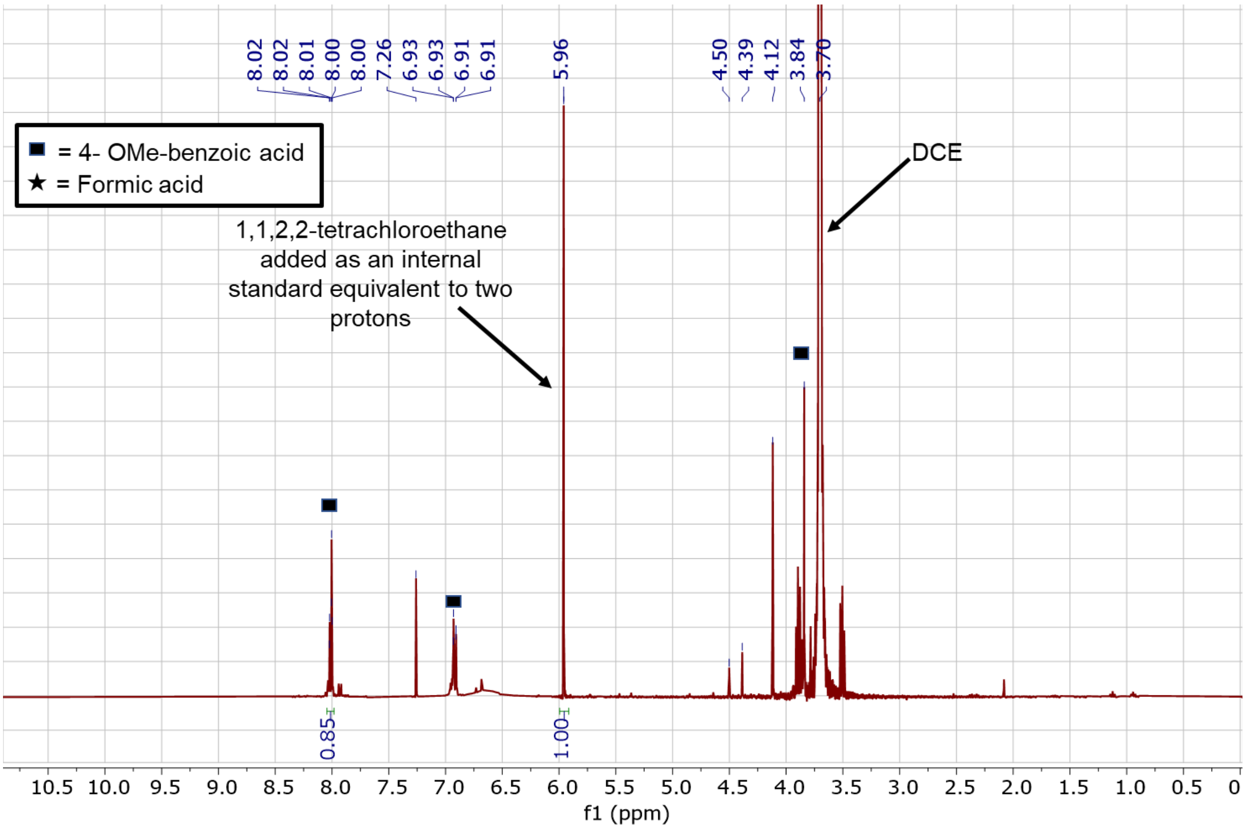

Figure S149: ^{1}H NMR spectrum of the 4-methoxystyrene oxidation reaction in $CDCl_3$ using $BA_2CsAg_{0.95}Na_{0.05}BiBr_7$ as a photocatalyst in DCE as a solvent with a 50 W blue LED as the light source.

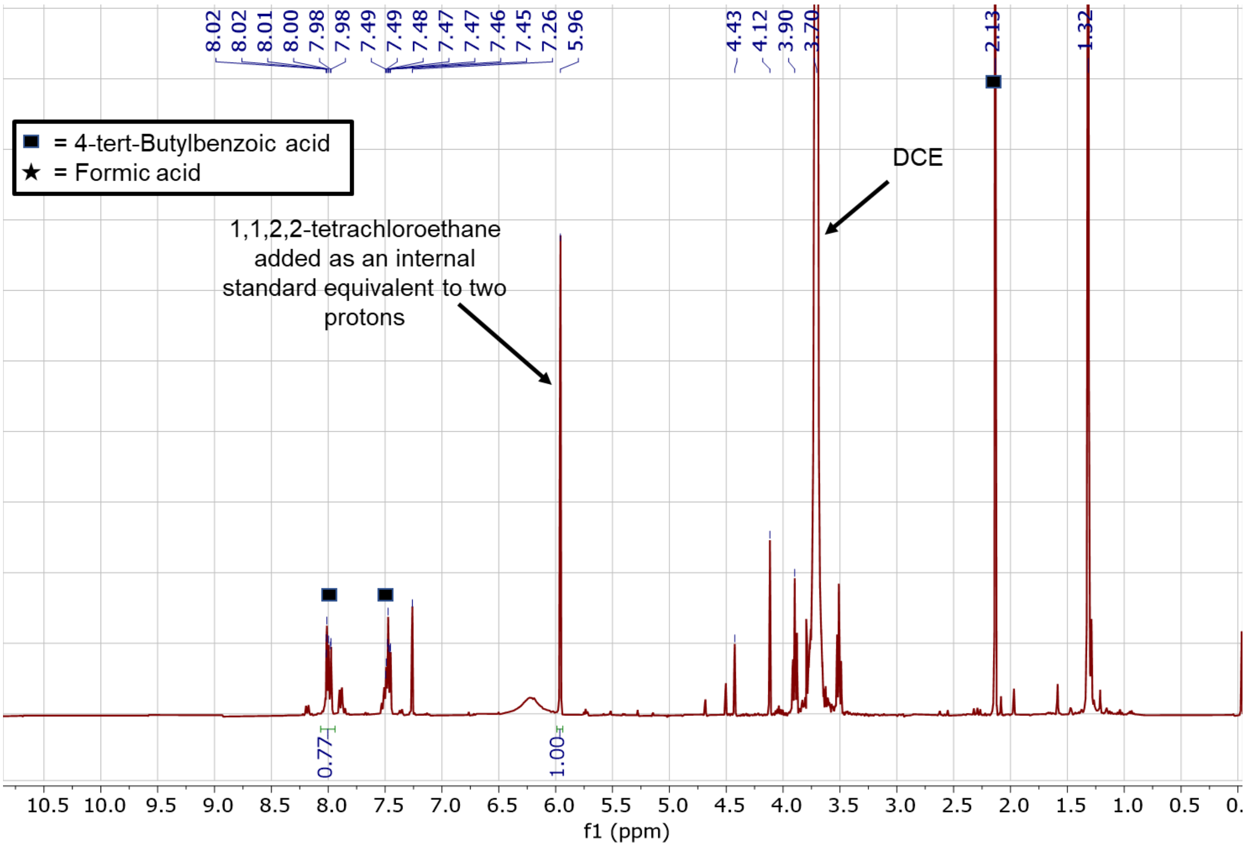

Figure S150: ^{1}H NMR spectrum of the 4-*tert*-butylstyrene oxidation reaction in $CDCl_3$ using $BA_2CsAg_{0.95}Na_{0.05}BiBr_7$ as a photocatalyst in DCE as a solvent with a 50 W blue LED as the light source.

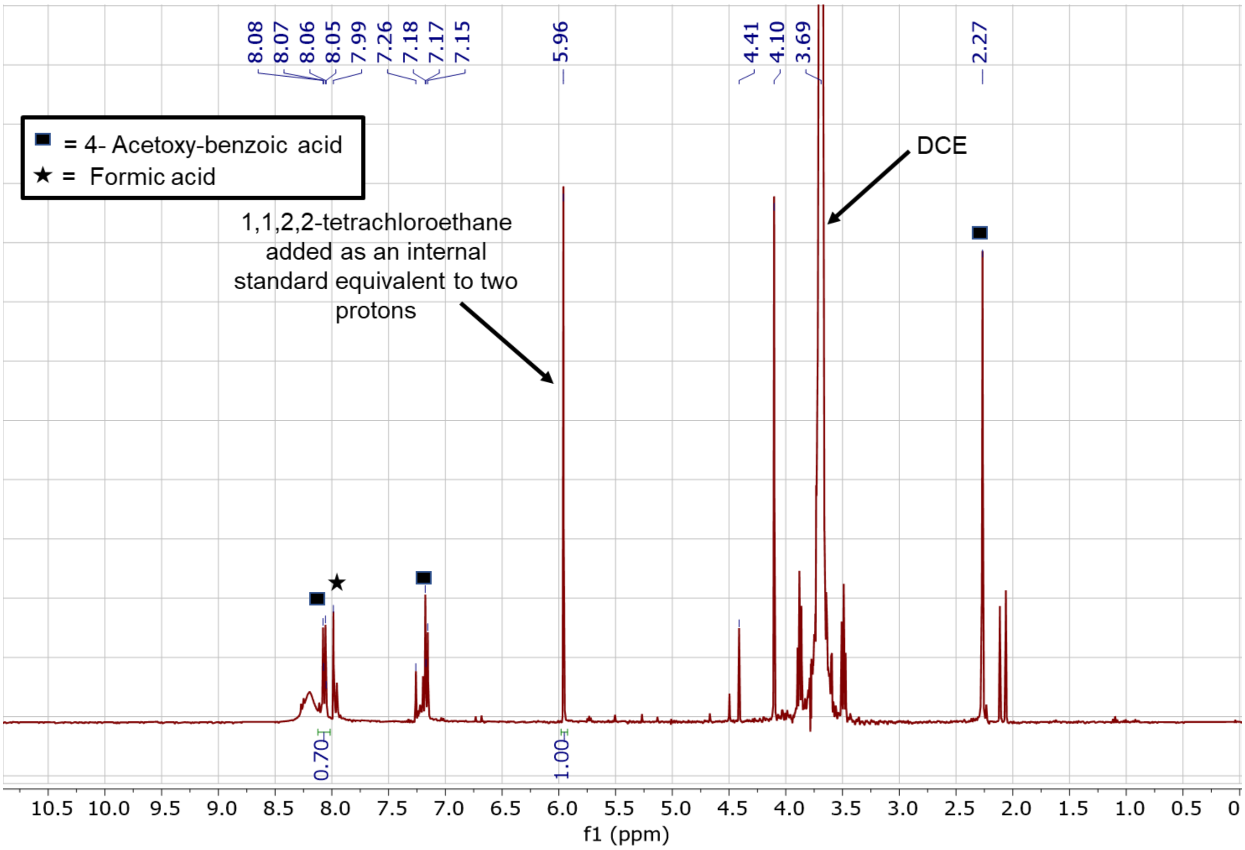

Figure S151: ^{1}H NMR spectrum of the 4-acetoxystyrene oxidation reaction in $CDCl_3$ using $BA_2CsAg_{0.95}Na_{0.05}BiBr_7$ as a photocatalyst in DCE as a solvent with a 50 W blue LED as the light source.

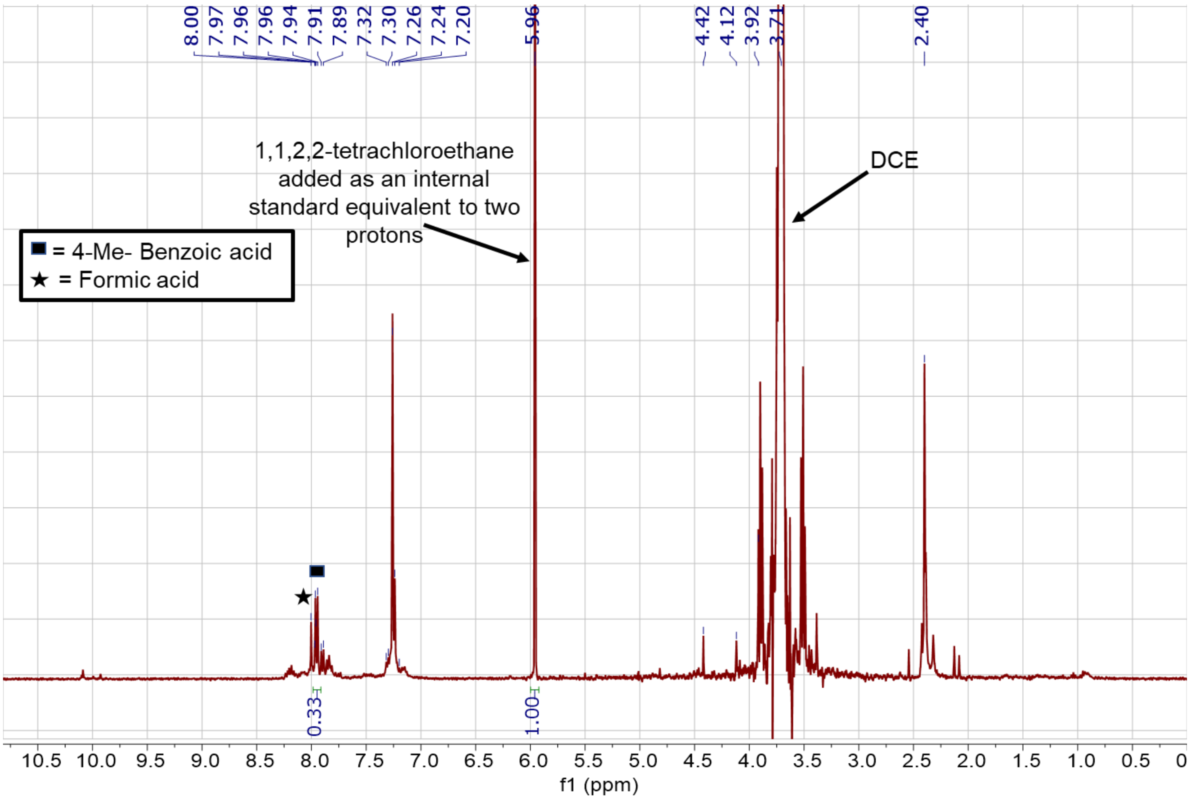

Figure S152: ^{1}H NMR spectrum of the 4-methylstyrene oxidation reaction in $CDCl_3$ using $BA_2CsAg_{0.95}Na_{0.05}BiBr_7$ as a photocatalyst in DCE as a solvent with a 50 W blue LED as the light source.

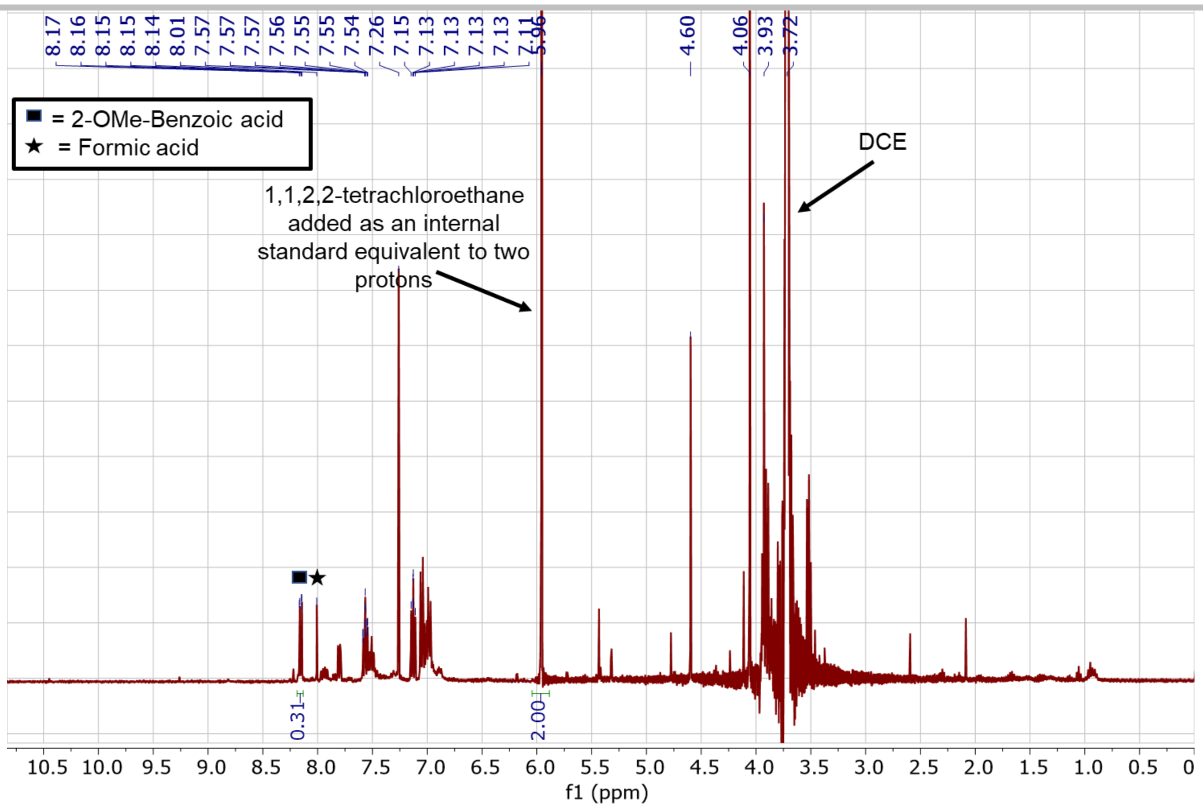

Figure S153: ^{1}H NMR spectrum of the 2-methoxystyrene oxidation reaction in $CDCl_3$ using $BA_2CsAg_{0.95}Na_{0.05}BiBr_7$ as a photocatalyst in DCE as a solvent with a 50 W blue LED as the light source.

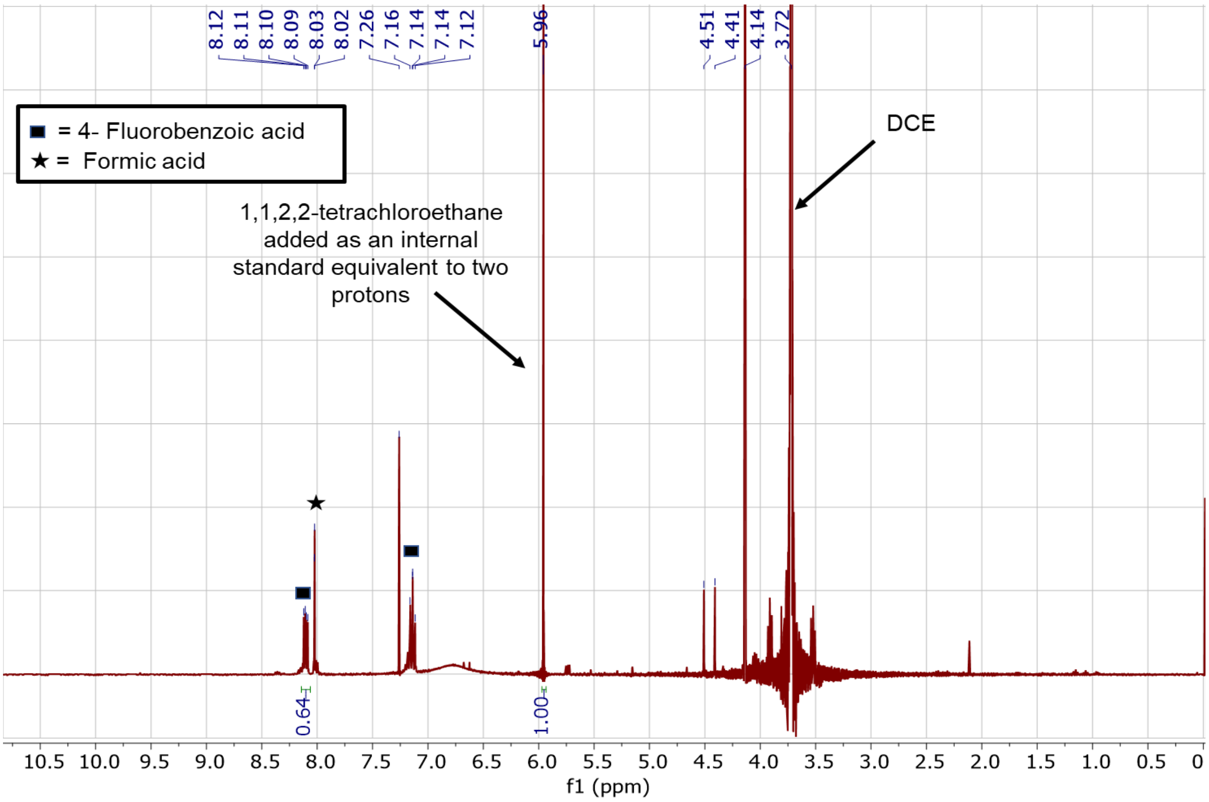

Figure S154: ^{1}H NMR spectrum of the 4-fluorostyrene oxidation reaction in $CDCl_3$ using $BA_2CsAg_{0.95}Na_{0.05}BiBr_7$ as a photocatalyst in DCE as a solvent with a 50 W blue LED as the light source.

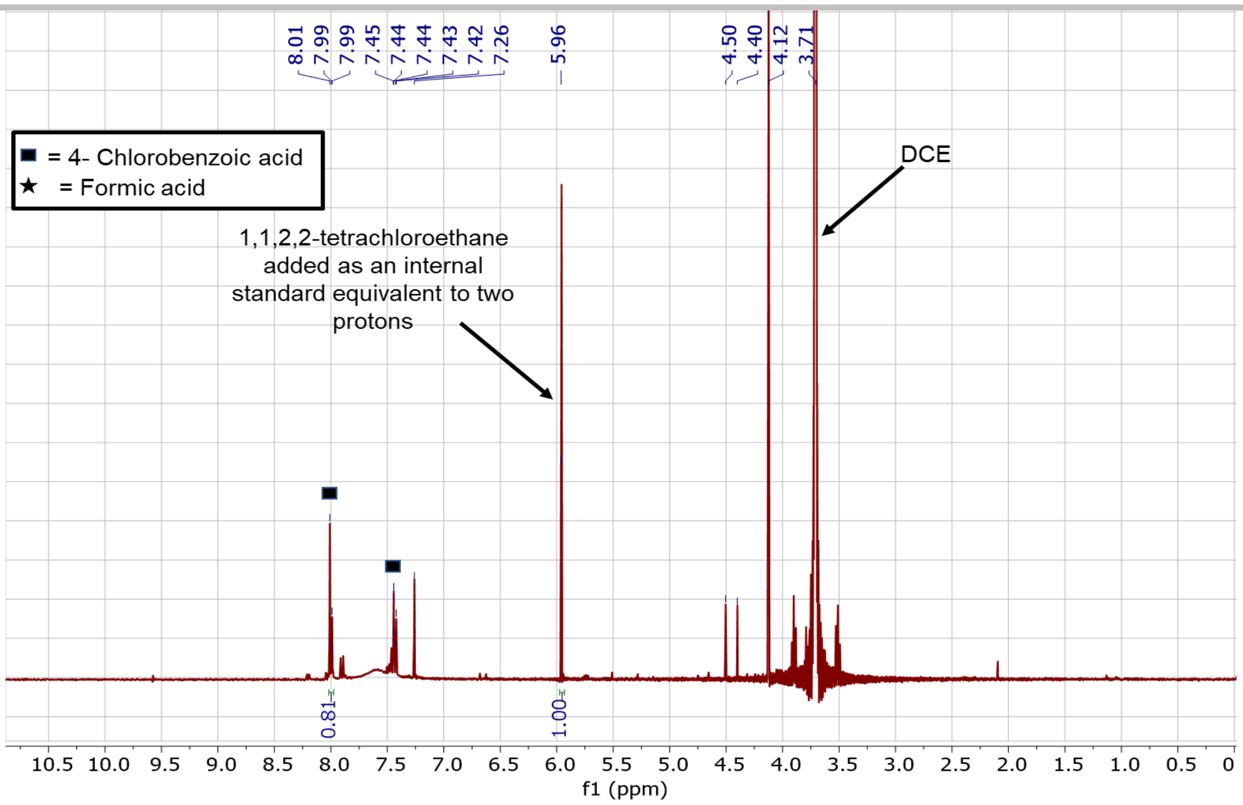

Figure S155: ^{1}H NMR spectrum of the 4-chlorostyrene oxidation reaction in $CDCl_3$ using $BA_2CsAg_{0.95}Na_{0.05}BiBr_7$ as a photocatalyst in DCE as a solvent with a 50 W blue LED as the light source.

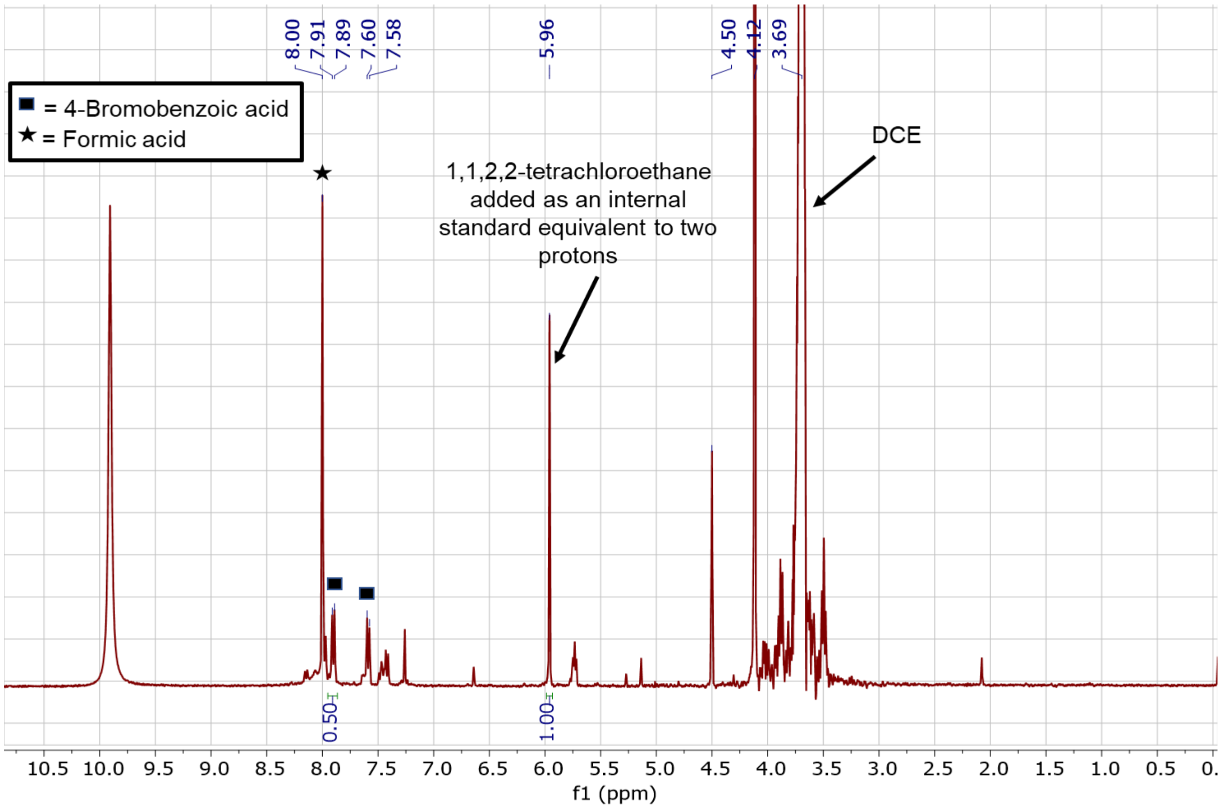

Figure S156: ^{1}H NMR spectrum of the 4-bromostyrene oxidation reaction in $CDCl_3$ using $BA_2CsAg_{0.95}Na_{0.05}BiBr_7$ as a photocatalyst in DCE as a solvent with a 50 W blue LED as the light source.

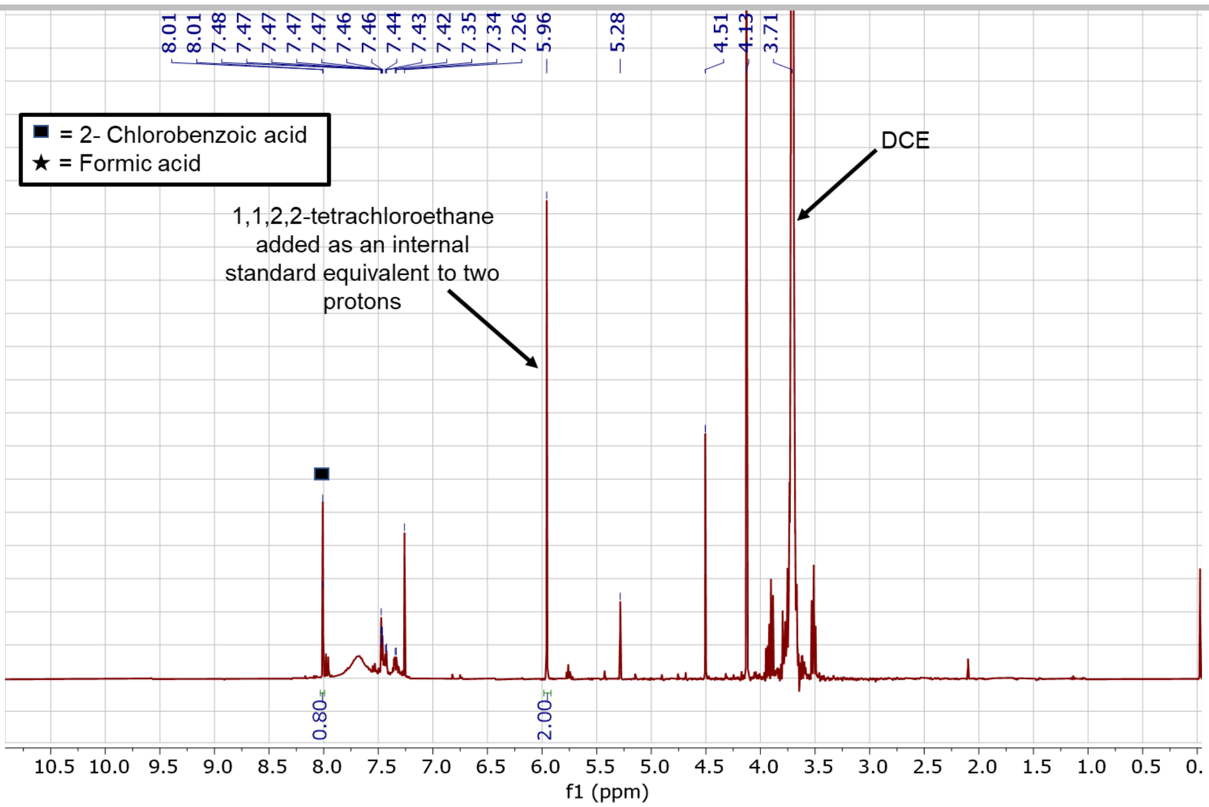

Figure S157: ^{1}H NMR spectrum of the 2-chlorostyrene oxidation reaction in $CDCl_3$ using $BA_2CsAg_{0.95}Na_{0.05}BiBr_7$ as a photocatalyst in DCE as a solvent with a 50 W blue LED as the light source.

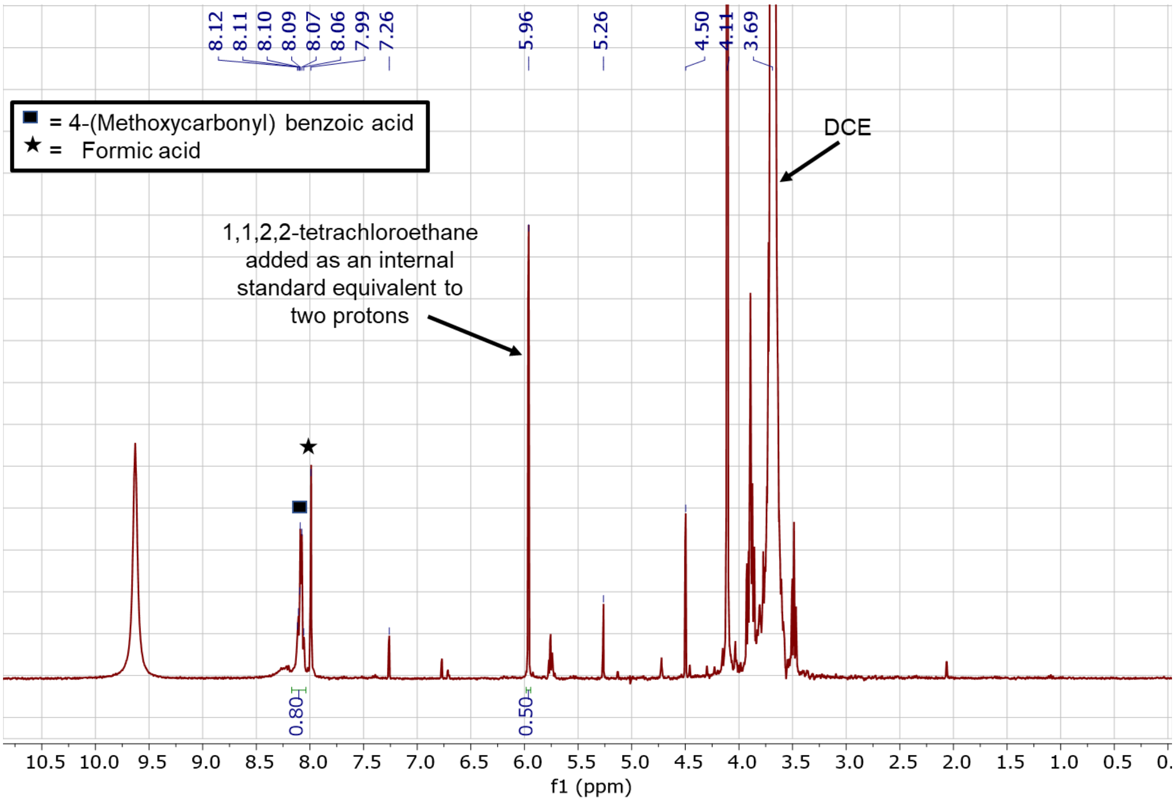

Figure S158: ^{1}H NMR spectrum of the methyl-4-vinylbenzoate oxidation reaction in $CDCl_3$ using $BA_2CsAg_{0.95}Na_{0.05}BiBr_7$ as a photocatalyst in DCE as a solvent with a 50 W blue LED as the light source.

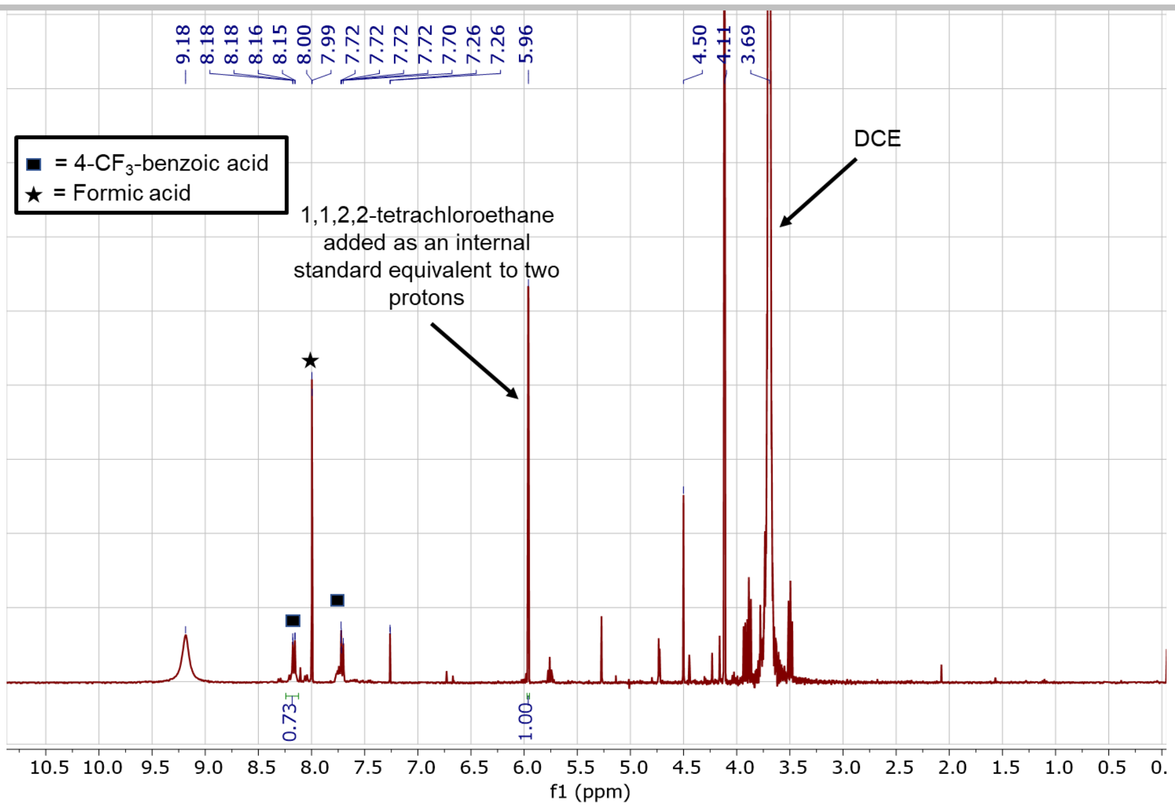

Figure S159: ^{1}H NMR spectrum of the 4-(trifluoromethyl)styrene oxidation reaction in $CDCl_3$ using $BA_2CsAg_{0.95}Na_{0.05}BiBr_7$ as a photocatalyst in DCE as a solvent with a 50 W blue LED as the light source.

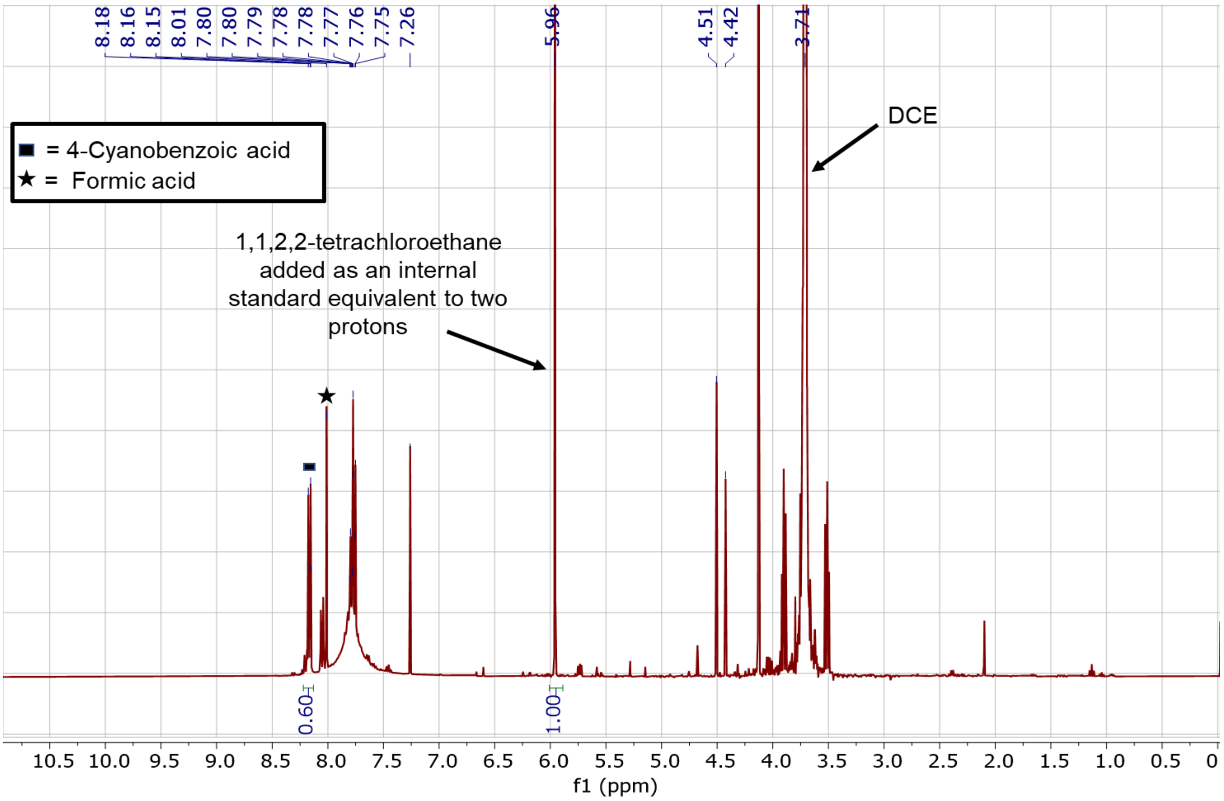

Figure S160: ^{1}H NMR spectrum of the 4-cyanostyrene oxidation reaction in $CDCl_3$ using $BA_2CsAg_{0.95}Na_{0.05}BiBr_7$ as a photocatalyst in DCE as a solvent with a 50 W blue LED as the light source.

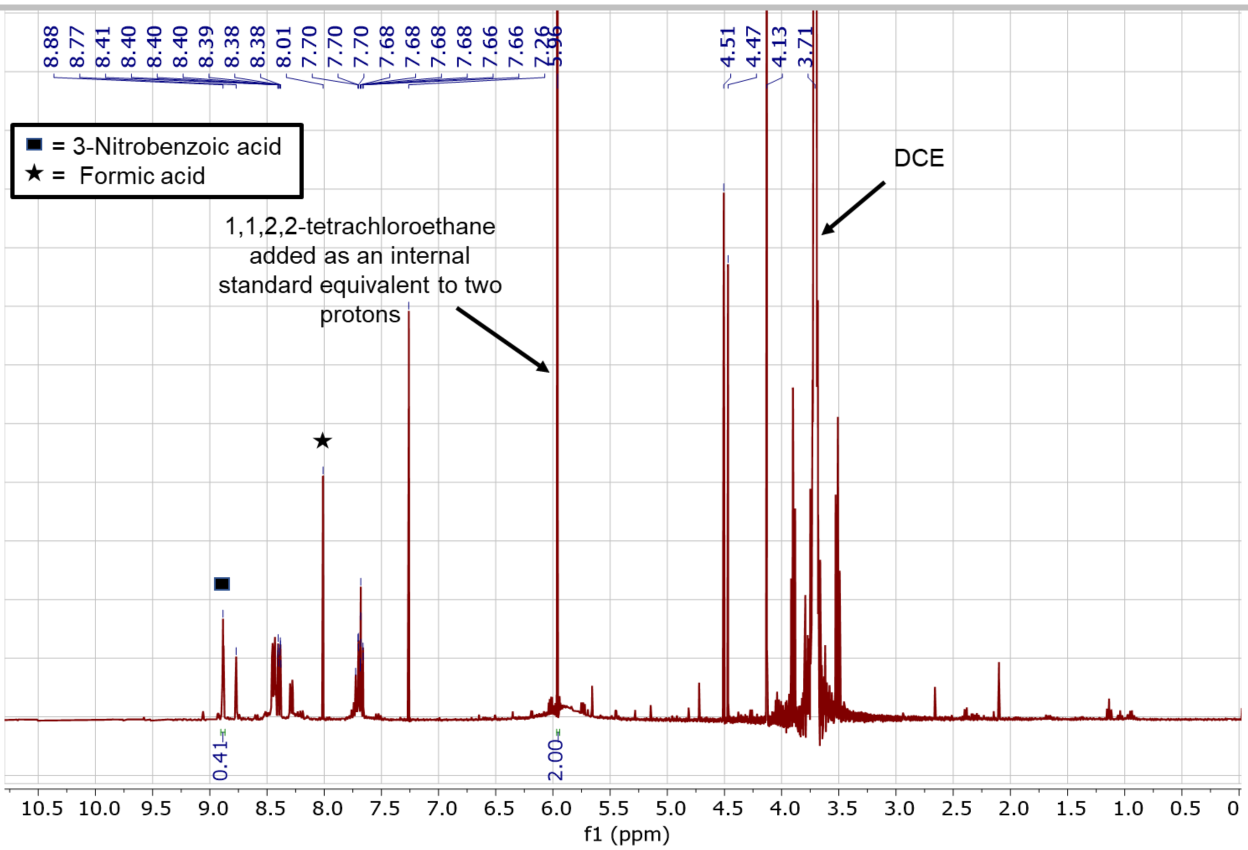

Figure S161: ^{1}H NMR spectrum of the 4-nitrostyrene oxidation reaction in $CDCl_3$ using $BA_2CsAg_{0.95}Na_{0.05}BiBr_7$ as a photocatalyst in DCE as a solvent with a 50 W blue LED as the light source.

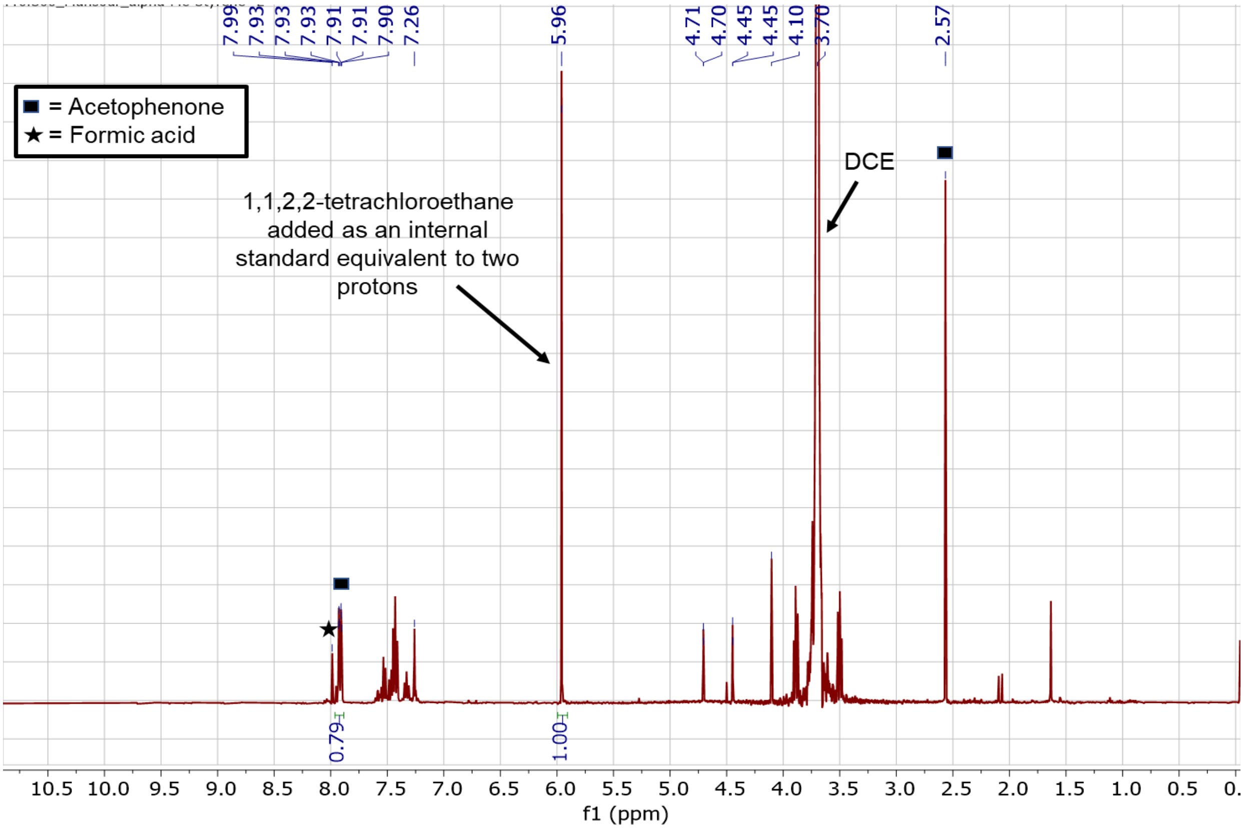

Figure S162: ^{1}H NMR spectrum of the α-methylstyrene oxidation reaction in $CDCl_3$ using $BA_2CsAg_{0.95}Na_{0.05}BiBr_7$ as a photocatalyst in DCE as a solvent with a 50 W blue LED as the light source.

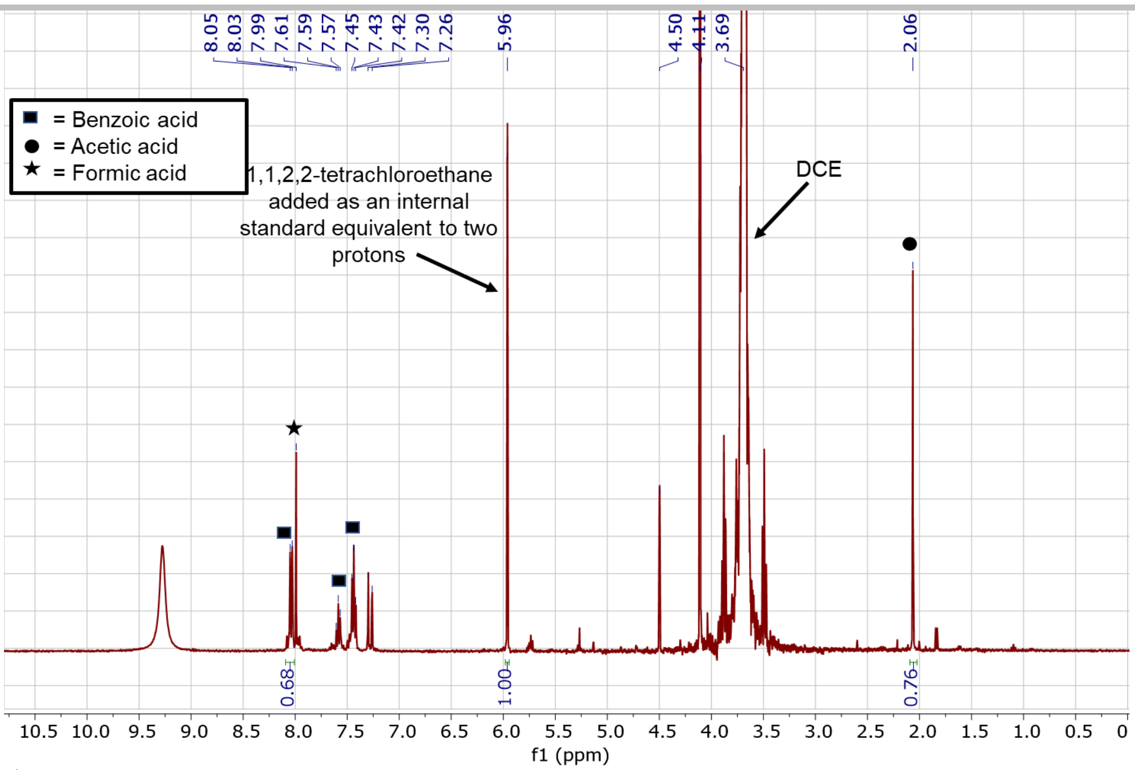

Figure S163: ^{1}H NMR spectrum of the *trans*-β-methylstyrene oxidation reaction in $CDCl_3$ using $BA_2CsAg_{0.95}Na_{0.05}BiBr_7$ as a photocatalyst in DCE as a solvent with a 50 W blue LED as the light source.

Calculations to obtain NMR yield of acetic acid (**4p**) from photocatalyzed *trans*-β-methylstyrene (**1p**) oxidation reaction
Integration of 1,1,2,2-tetrachloroethane = 1.00 (two protons); integration of methyl group of acetic acid = 0.76 (three protons).
Yield of **4p** = $(0.76 \times \frac{2}{3}) \times 100\% = 51\%$.

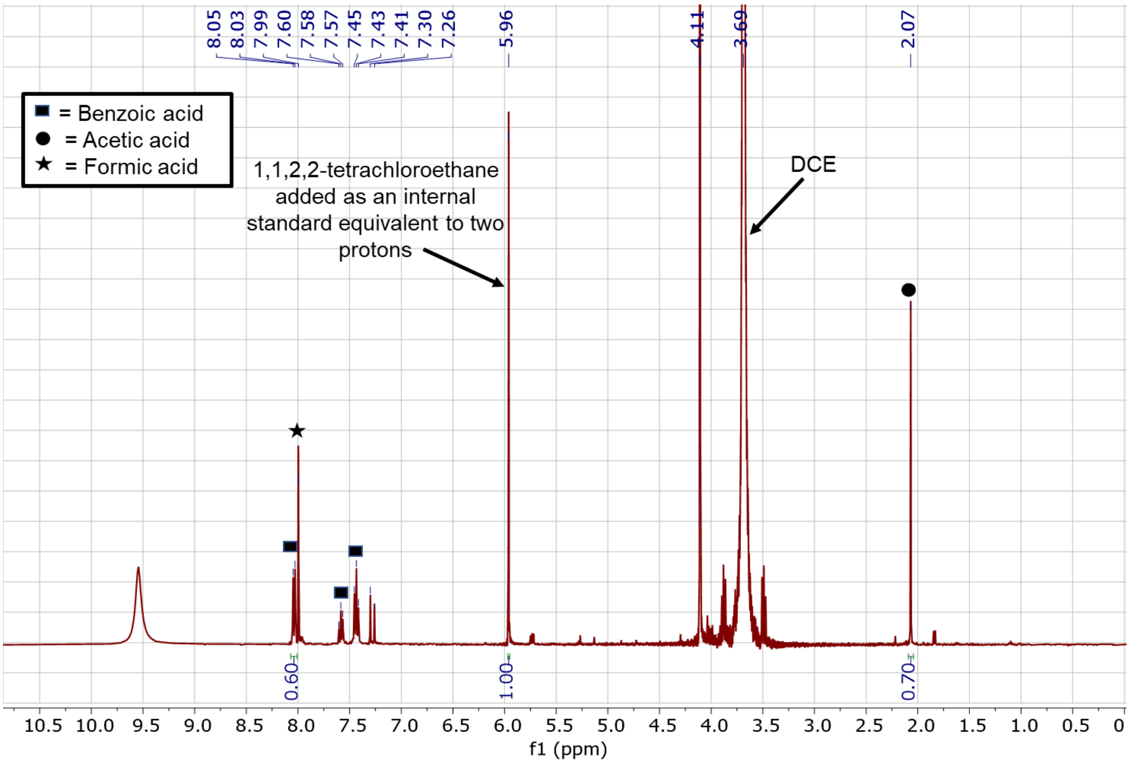

Figure S164: ^{1}H NMR spectrum of the *cis*-β-methylstyrene oxidation reaction in $CDCl_3$ using $BA_2CsAg_{0.95}Na_{0.05}BiBr_7$ as a photocatalyst in DCE as a solvent with a 50 W blue LED as the light source.

Calculations to obtain NMR yield of **4p** from photocatalyzed *cis*-β-methylstyrene (**1q**) oxidation reaction

Integration of 1,1,2,2-tetrachloroethane = 1.00 (two protons); integration of methyl group of acetic acid = 0.70 (three protons).

Yield of **4p** = $\left(0.70 \times \frac{2}{3}\right) \times 100\% = 46\%$.

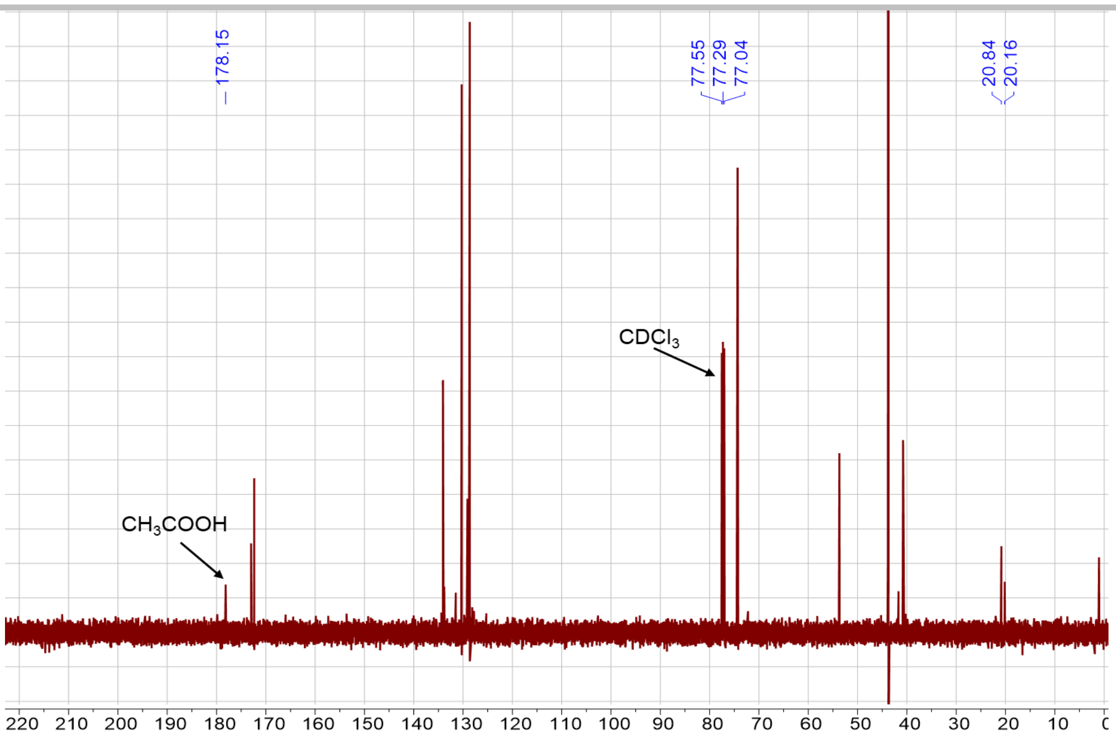

Figure S165: $^{13}C\{^{1}H\}$ NMR spectrum of the *cis*-β-methylstyrene oxidation reaction in $CDCl_3$ using $BA_2CsAg_{0.95}Na_{0.05}BiBr_7$ as a photocatalyst in DCE as a solvent with a 50 W blue LED as the light source.

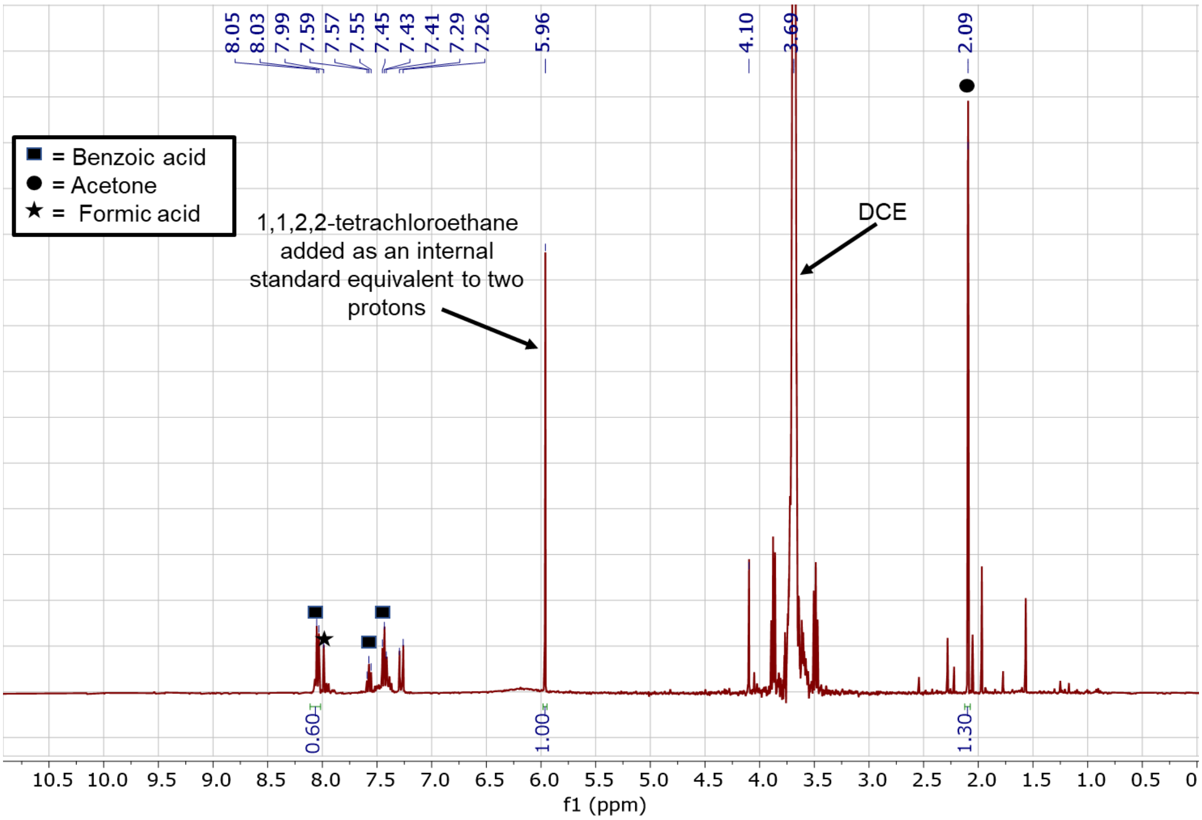

Figure S166: ^{1}H NMR spectrum of the β-dimethylstyrene oxidation reaction in $CDCl_3$ using $BA_2CsAg_{0.95}Na_{0.05}BiBr_7$ as a photocatalyst in DCE as a solvent with a 50 W blue LED as the light source.

Calculations to obtain NMR yield of acetone (**4r**) of the photocatalyzed β-dimethylstyrene (**1r**) oxidation reaction

Integration of 1,1,2,2-tetrachloroethane = 1.00 (two protons); integration of di-methyl group of acetone = 1.30 (six protons).

Yield of **4r** = $\left(1.30 \times \frac{2}{6}\right) \times 100\% = 43\%$.

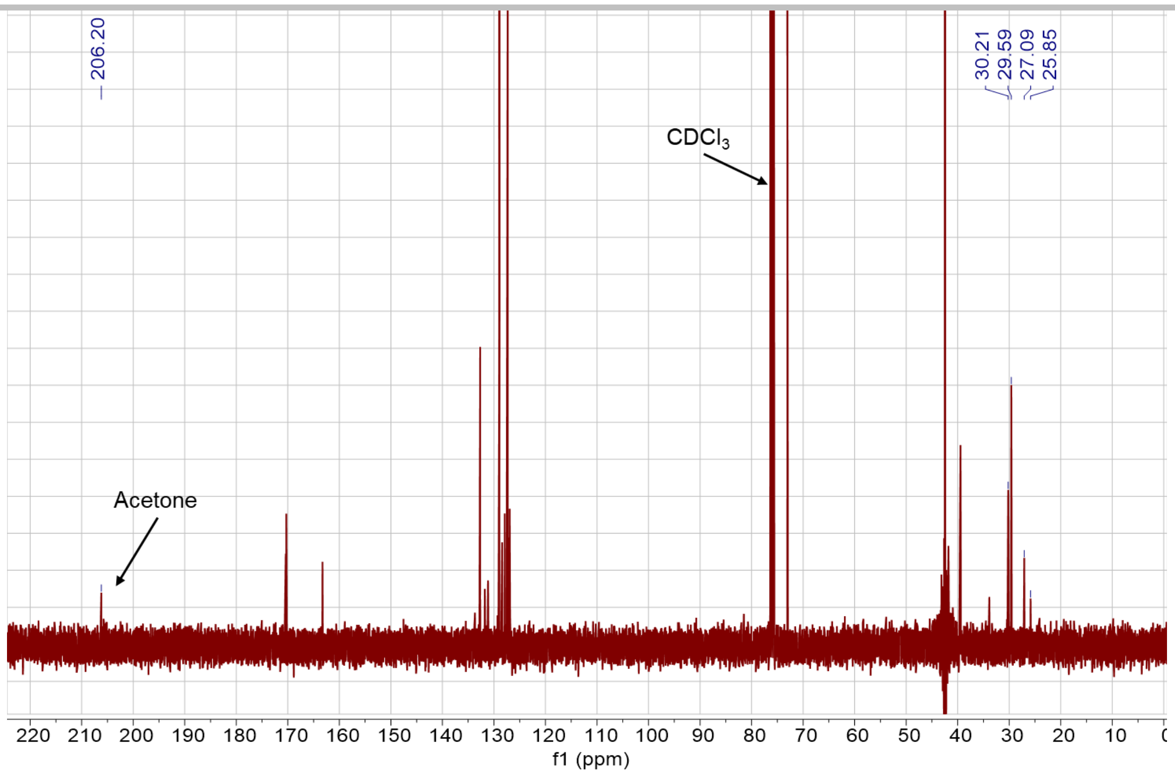

Figure S167: $^{13}C\{^1H\}$ NMR spectrum of the β-dimethylstyrene oxidation reaction in $CDCl_3$ using $BA_2CsAg_{0.95}Na_{0.05}BiBr_7$ as a photocatalyst in DCE as a solvent with a 50 W blue LED as the light source.

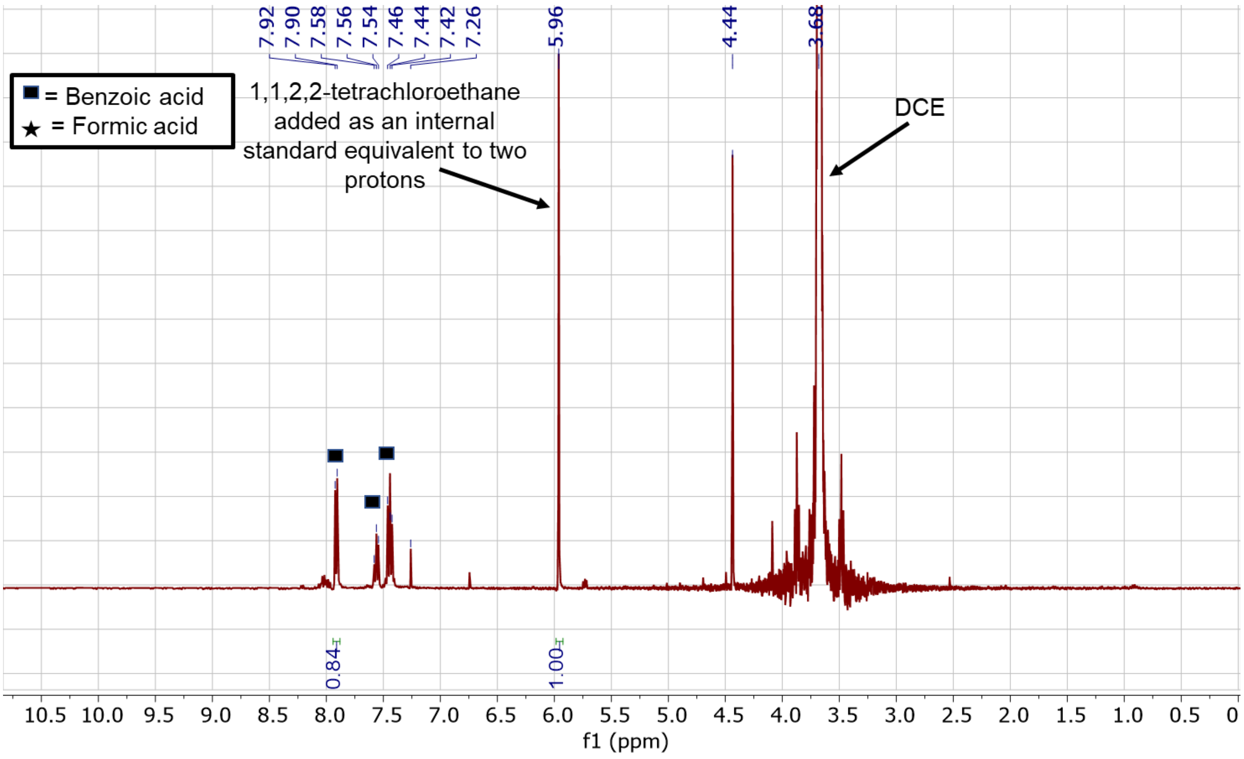

Figure S168: ^1H NMR spectrum of the α-bromostyrene oxidation reaction in $CDCl_3$ using $BA_2CsAg_{0.95}Na_{0.05}BiBr_7$ as a photocatalyst in DCE as a solvent with a 50 W blue LED as the light source.

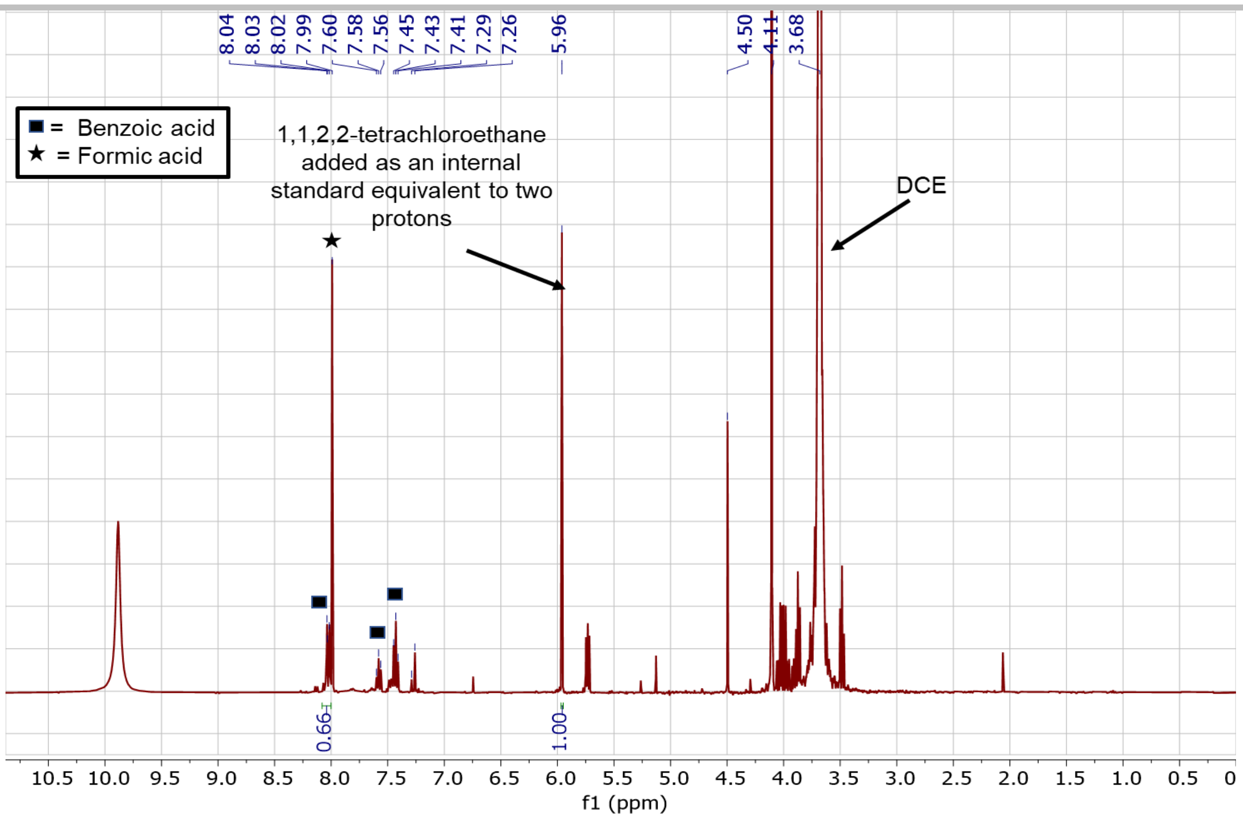

Figure S169: ^{1}H NMR spectrum of the β -bromostyrene oxidation reaction in $CDCl_3$ using $BA_2CsAg_{0.95}Na_{0.05}BiBr_7$ as a photocatalyst in DCE as a solvent with a 50 W blue LED as the light source.

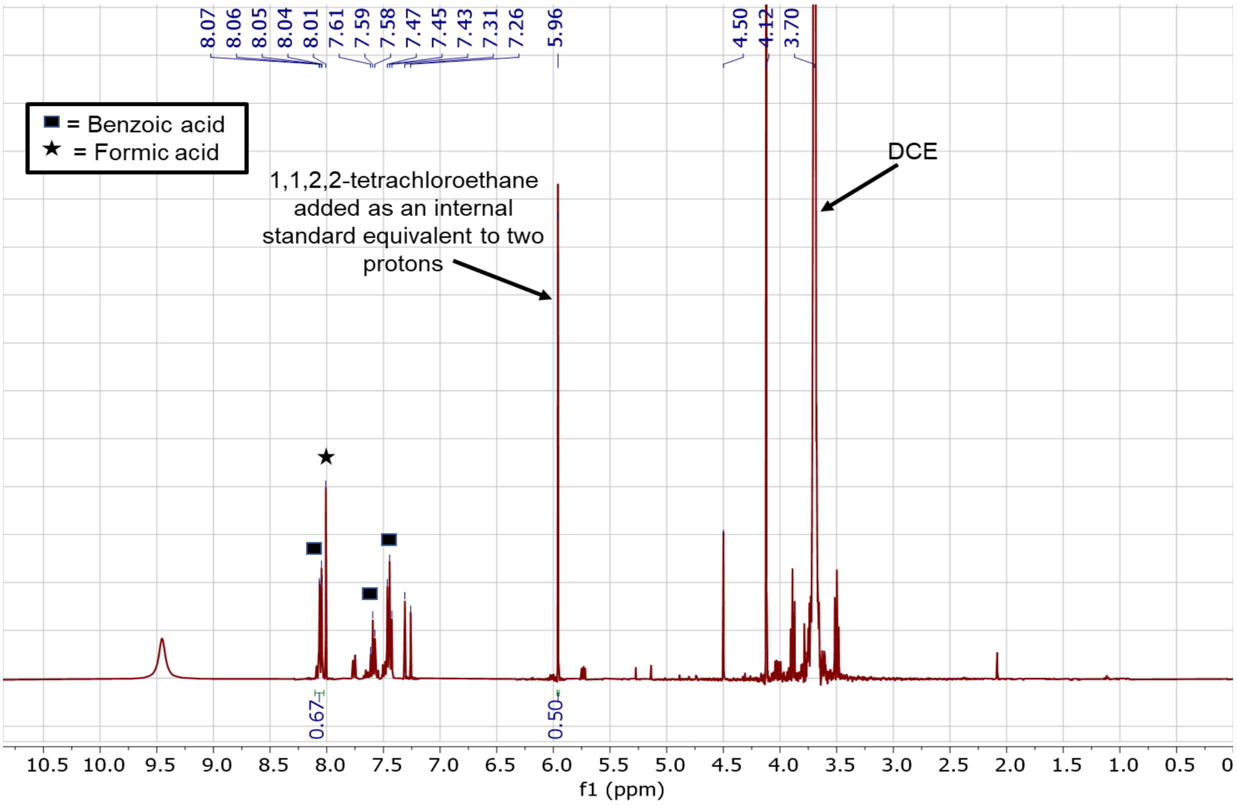

Figure S170: ^{1}H NMR spectrum of the *trans*-stilbene oxidation reaction in $CDCl_3$ using $BA_2CsAg_{0.95}Na_{0.05}BiBr_7$ as a photocatalyst in DCE as a solvent with a 50 W blue LED as the light source.

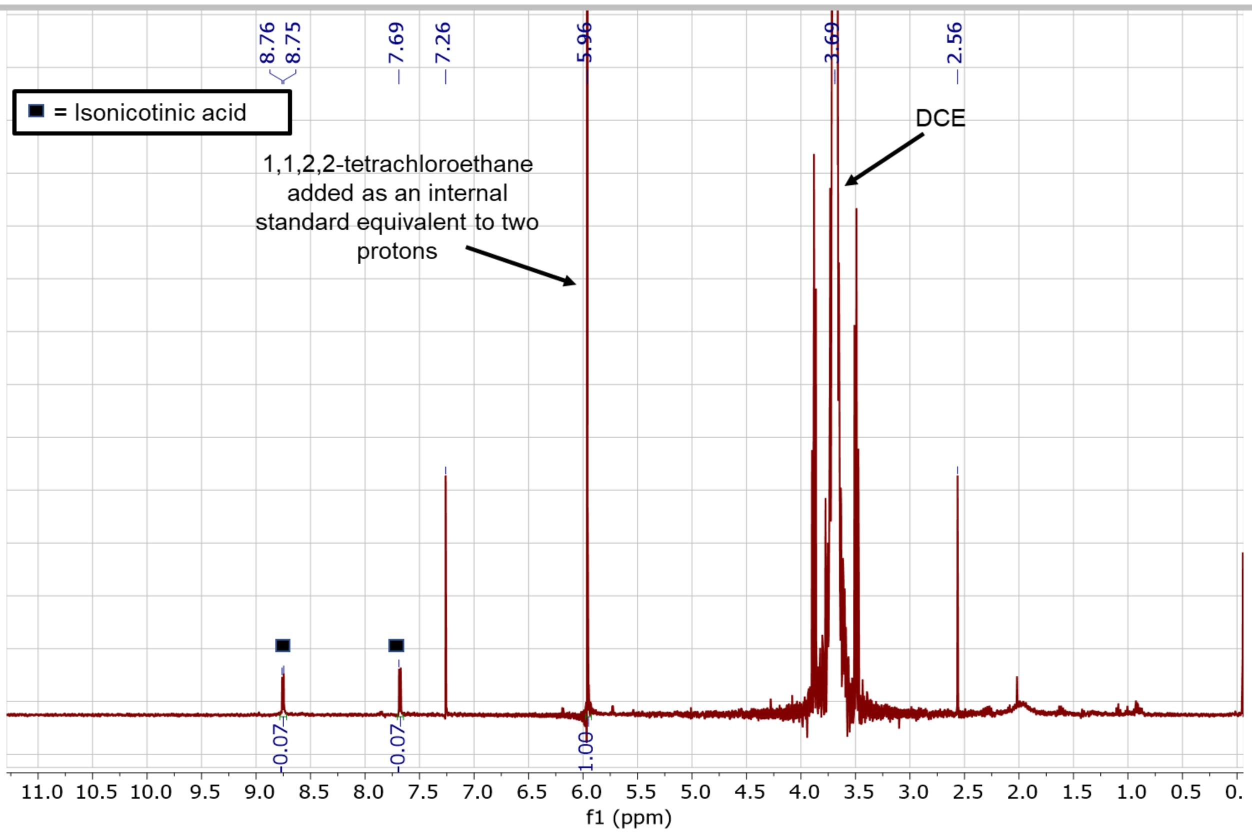

Figure S171: ^{1}H NMR spectrum of the 4-vinyl pyridine oxidation reaction in $CDCl_3$ using $BA_2CsAg_{0.95}Na_{0.05}BiBr_7$ as a photocatalyst in DCE as a solvent with a 50 W blue LED as the light source.

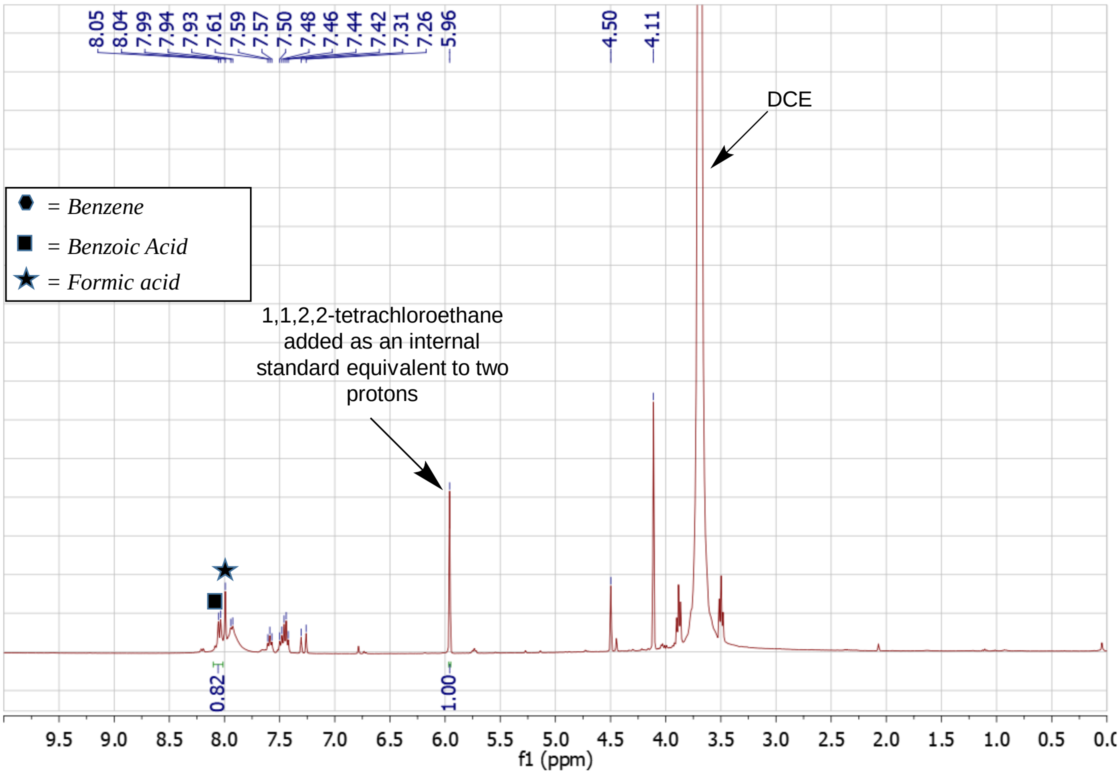

Figure S172: ^{1}H NMR spectrum of the styrene oxidation reaction in $CDCl_3$ using $BA_2CsAg_{0.95}Na_{0.05}BiBr_7$ as a photocatalyst in DCE as a solvent with a 50 W blue LED as the light source after 90 hours.

Benzoic acid (**3a**)

^{1}H NMR (400 MHz, CHLOROFORM-*D*) δ 7.49 (t, *J* = 7.6 Hz, 2 H), 7.58 – 7.67 (m, 1 H), 8.14 (d, *J* = 8.5 Hz, 2 H).

^{13}C{^{1}H} NMR (100 MHz, CHLOROFORM-*D*) δ 127.3, 128.1, 129.0, 132.6, 170.9.

HRMS (ESI-QTOF, m/z) calculated for $[C_7H_5O_2]^-$ *m/z* = 121.0137, found 121.0143.

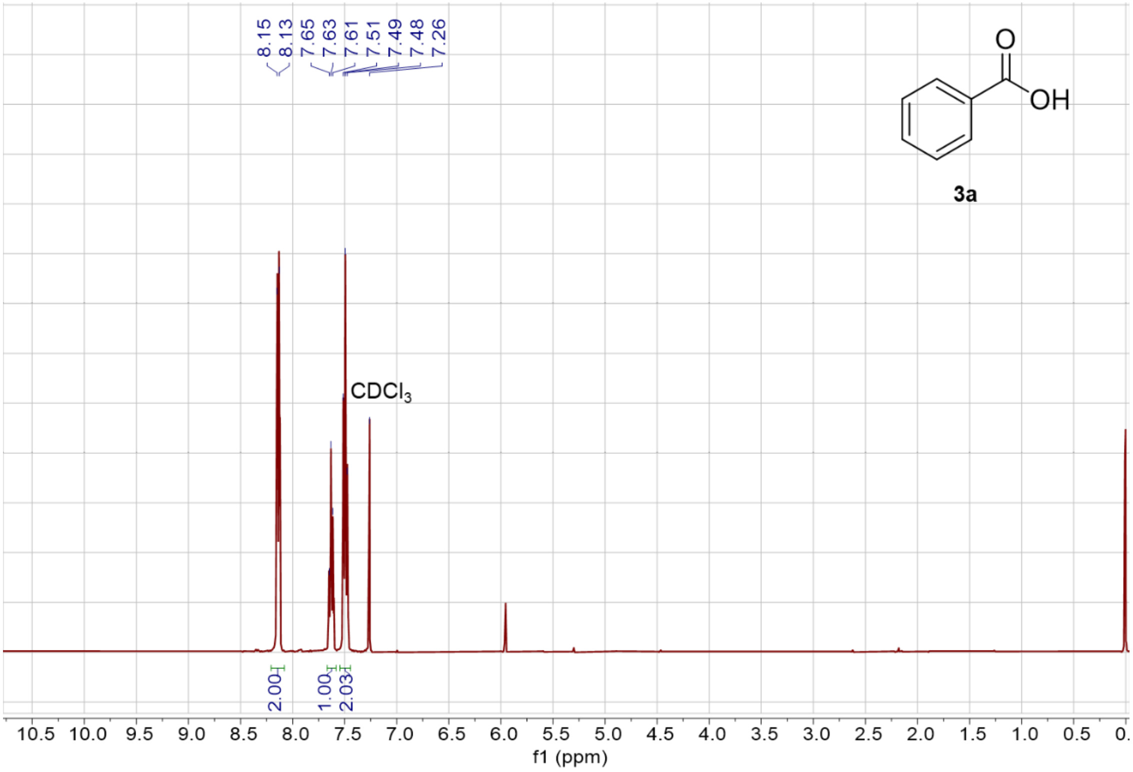

Figure S173: ^{1}H NMR spectrum of the isolated **3a** in $CDCl_3$ as the solvent.

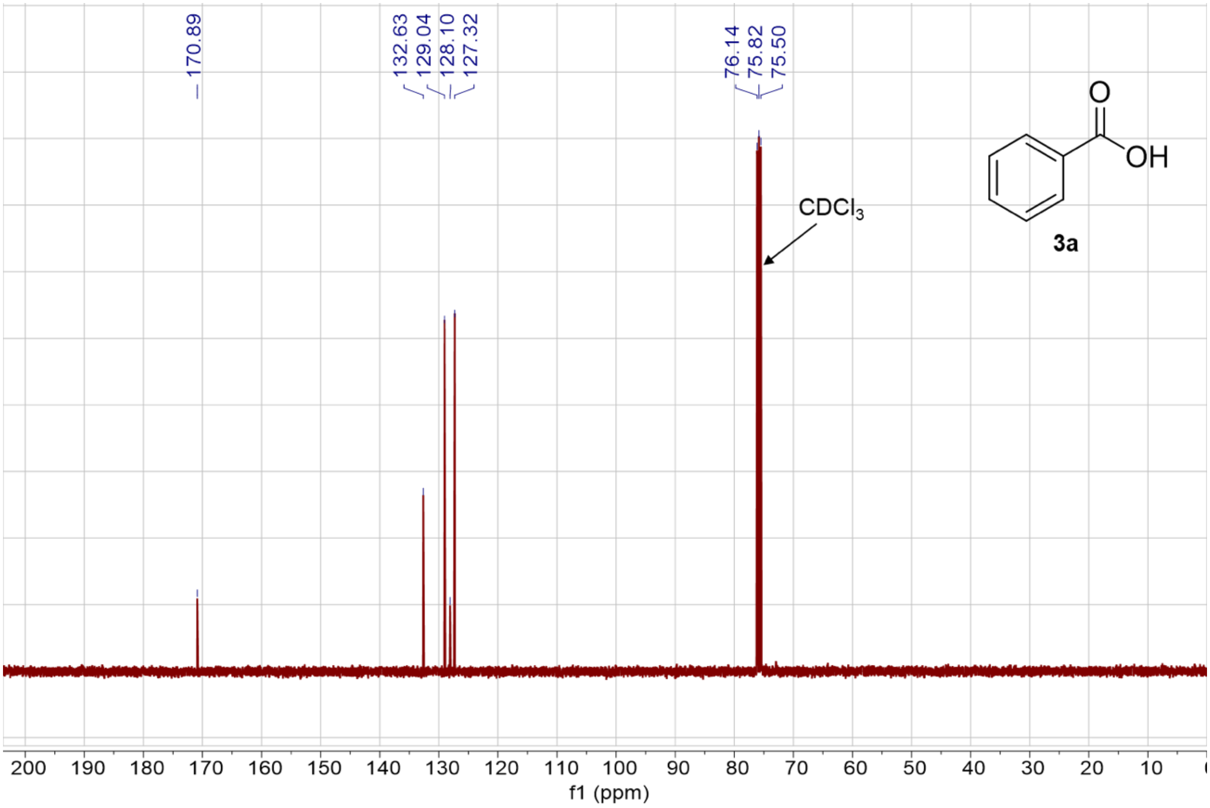

Figure S174: ^{13}C{^{1}H} NMR spectrum of the isolated **3a** in $CDCl_3$ as the solvent.

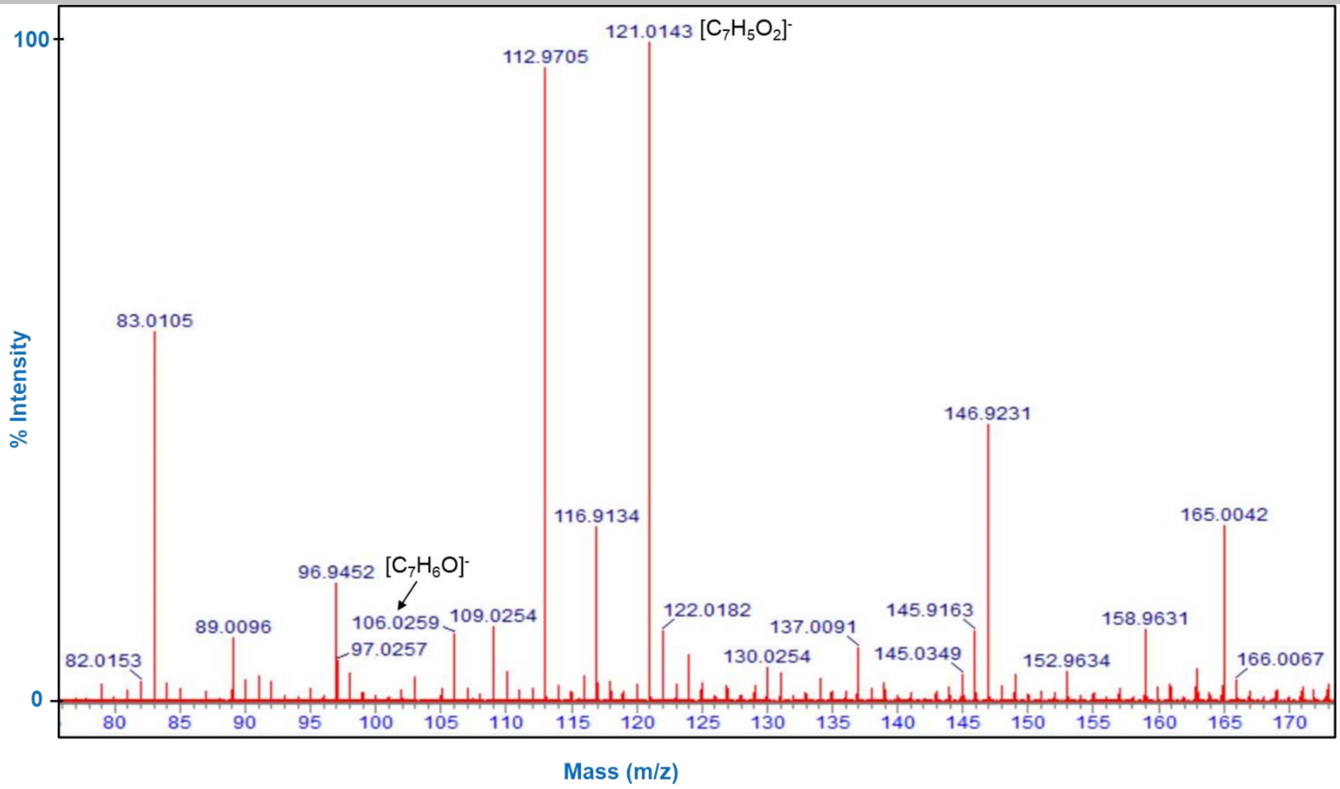

Figure S175: High-resolution mass spectrum (HRMS) of the isolated **3a**.

4-methoxybenzoic acid (**3b**)
^{1}H NMR (400 MHz, CHLOROFORM-*D*) δ 3.88 (s, 3 H), 6.95 (d, *J* = 8.9 Hz, 2 H), 8.07 (d, *J* = 8.9 Hz, 2 H).
^{13}C{^{1}H} NMR (100 MHz, CHLOROFORM-*D*) δ 54.2, 112.5, 120.3, 131.1, 162.8, 169.6.
HRMS (ESI-QTOF, m/z) calculated for $[C_8H_7O_3]^-$ *m/z* = 151.0243, found 151.0249.

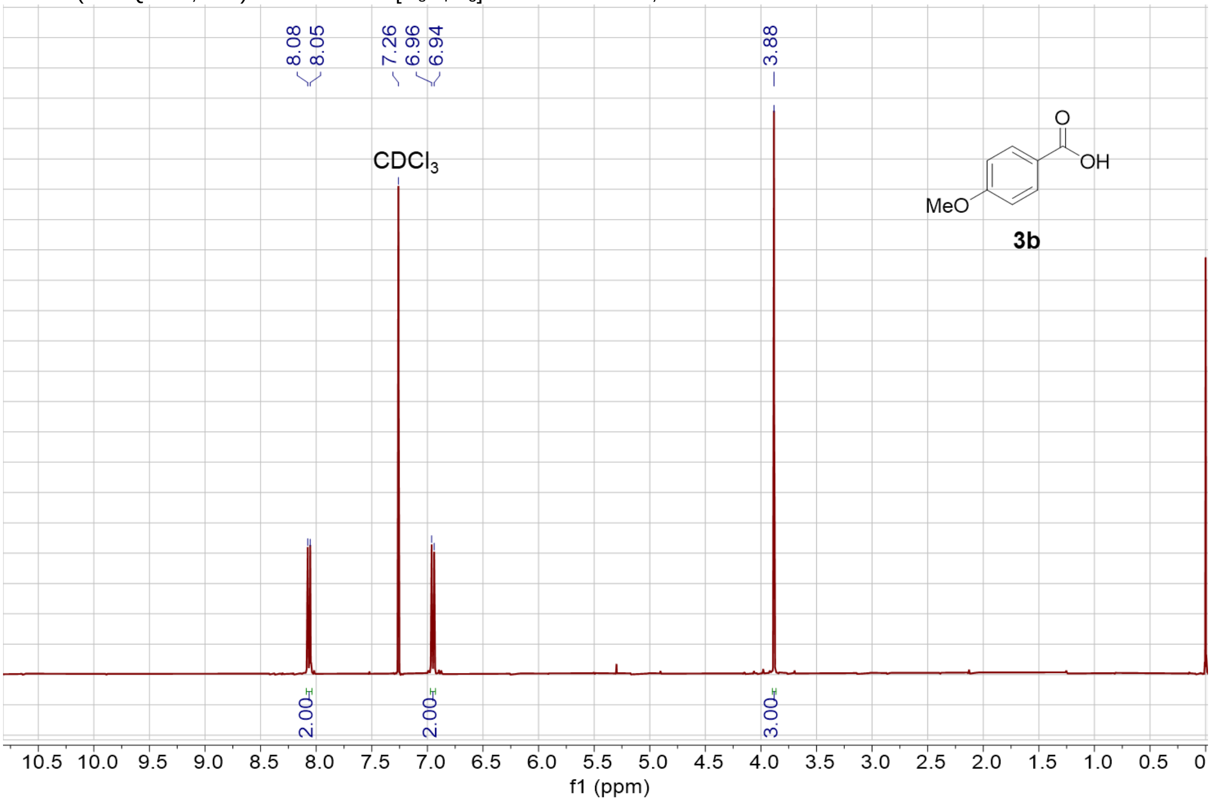

Figure S176: ^{1}H NMR spectrum of the isolated **3b** in $CDCl_3$ as the solvent.

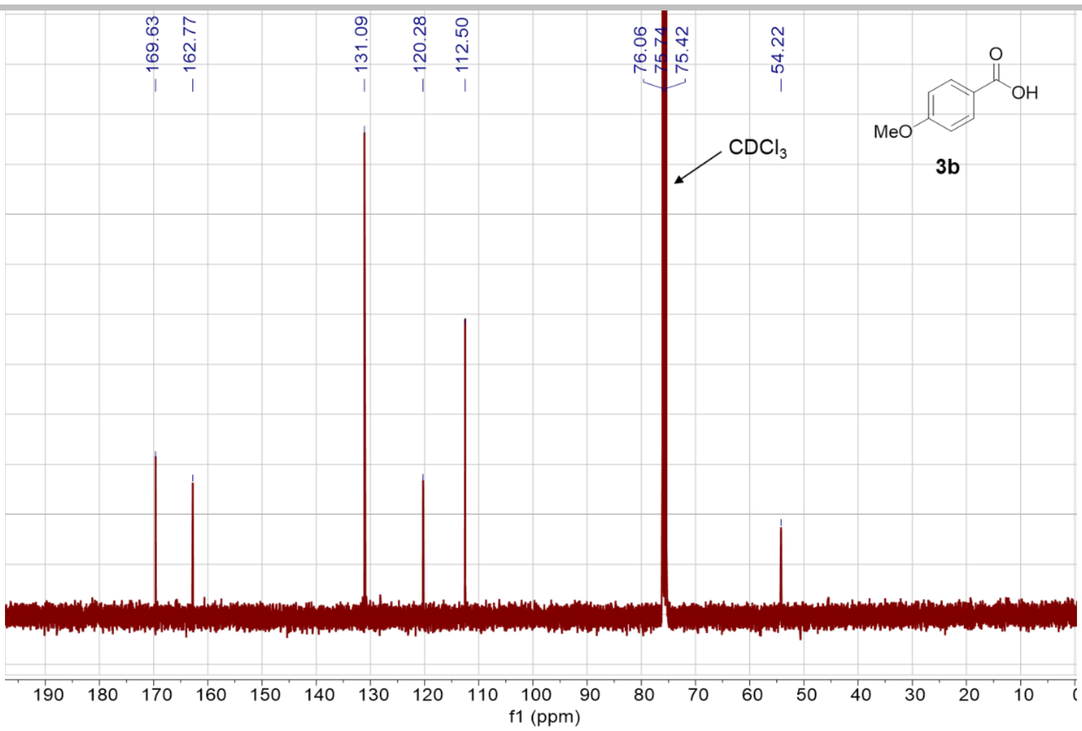

Figure S177: $^{13}C\{^{1}H\}$ NMR spectrum of the isolated **3b** in $CDCl_3$ as the solvent.

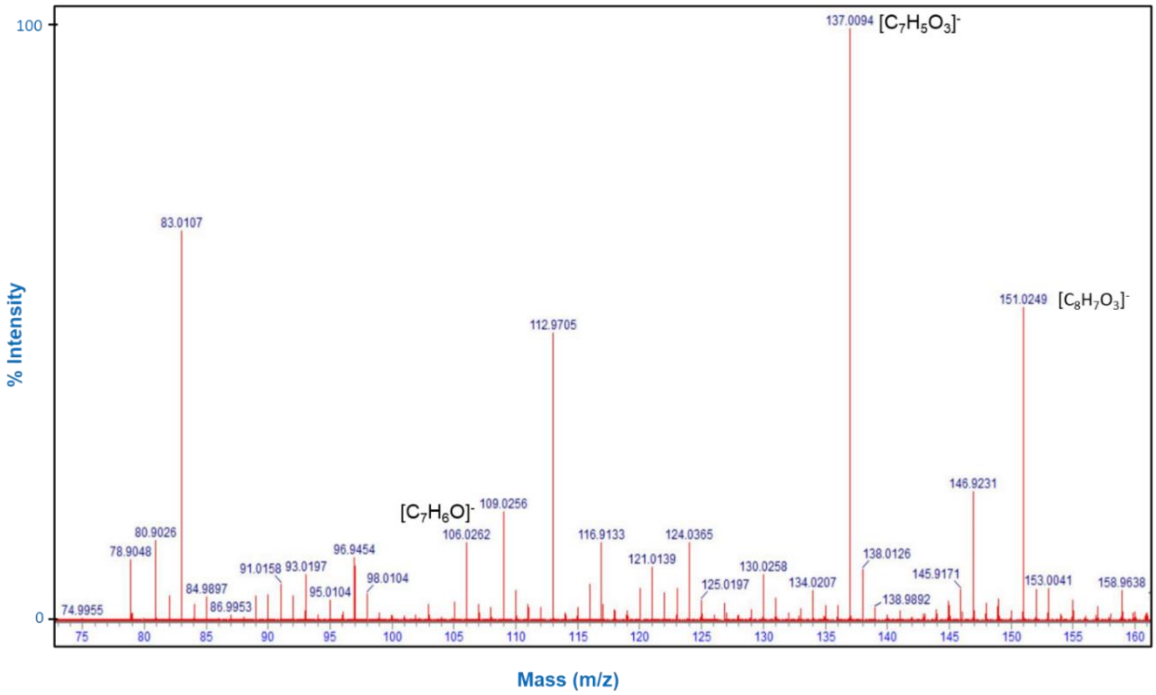

Figure S178: HRMS of the isolated **3b**.

4-*tert*-butylbenzoic acid (**3c**)

^{1}H NMR (400 MHz, CHLOROFORM-*D*) δ 1.35 (s, 9 H), 7.49 (d, *J* = 8.5 Hz, 2 H), 8.04 (d, *J* = 8.6 Hz, 2 H).

^{13}C{^{1}H} NMR (100 MHz, CHLOROFORM-*D*) δ 29.9, 34.0, 124.3, 125.2, 129.0, 156.5, 170.5.

HRMS (ESI-QTOF, m/z) calculated for $[C_{11}H_{13}O_2]^-$ *m/z* = 177.0763, found 177.0771.

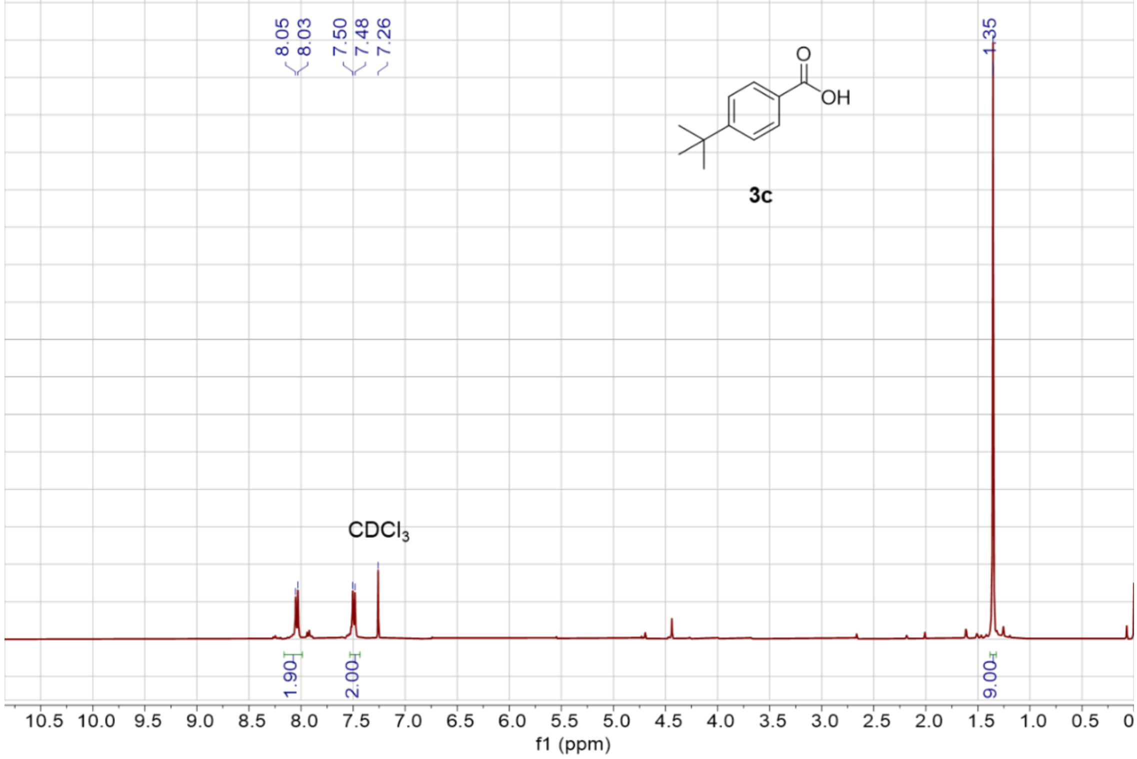

Figure S179: ^{1}H NMR spectrum of the isolated **3c** in $CDCl_3$ as the solvent.

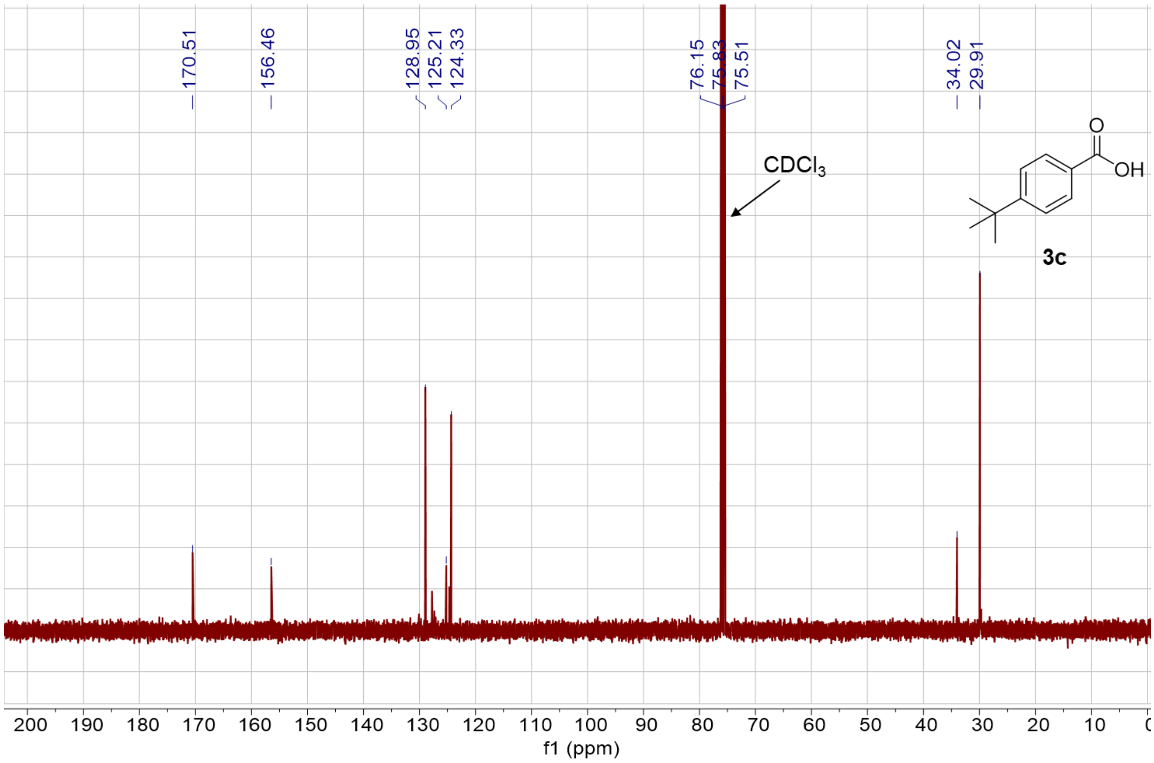

Figure S180: ^{13}C{^{1}H} NMR spectrum of the isolated **3c** in $CDCl_3$ as the solvent.

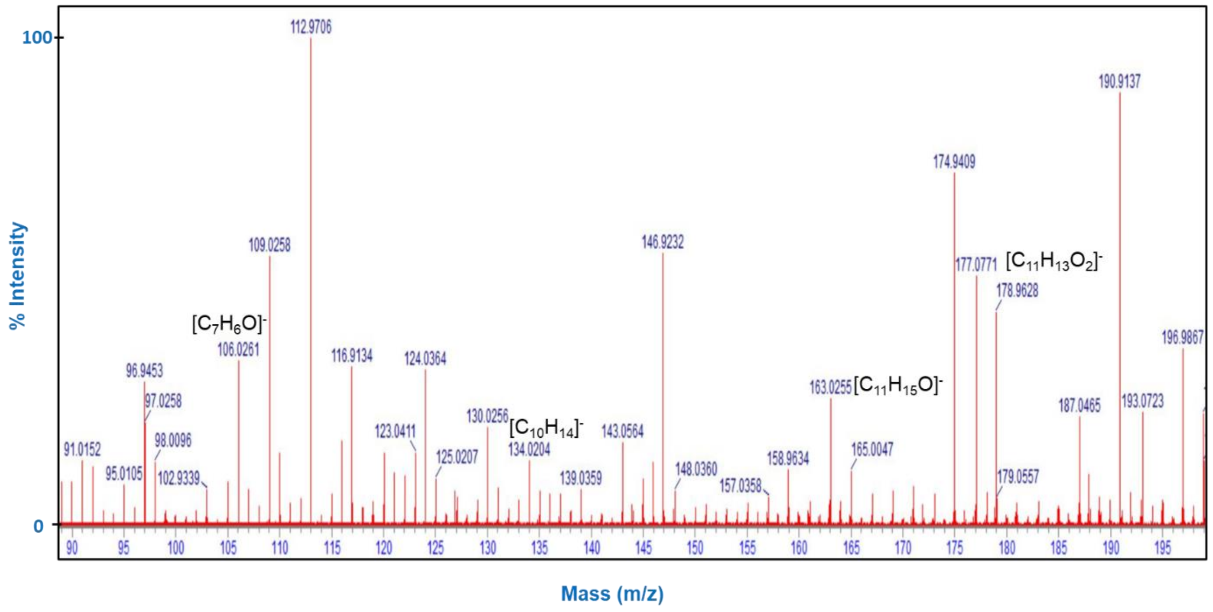

Figure S181: HRMS of the isolated **3c**.

4-acetoxybenzoic acid (**3d**)

^{1}H NMR (400 MHz, CHLOROFORM-*D*) δ 2.34 (s, 3 H), 7.21 (d, *J* = 8.6 Hz, 2 H), 8.14 (d, *J* = 8.6 Hz, 2 H).

^{13}C{^{1}H} NMR (100 MHz, CHLOROFORM-*D*) δ 20.0, 120.6, 125.5, 130.7, 153.8, 167.7, 169.3.

HRMS (ESI-QTOF, m/z) calculated for $[C_9H_7O_4]^-$ *m/z* = 179.0245, found 179.0204.

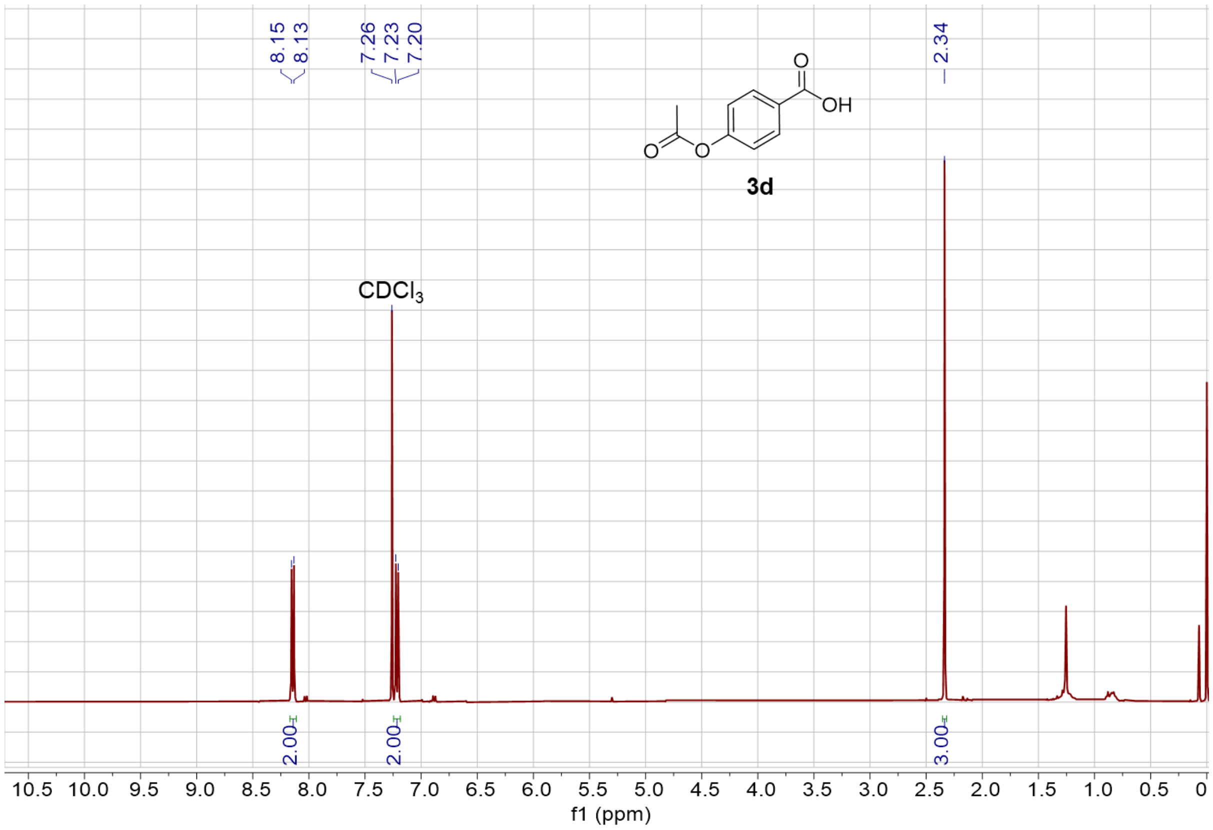

Figure S182: ^{1}H NMR spectrum of the isolated **3d** in $CDCl_3$ as the solvent.

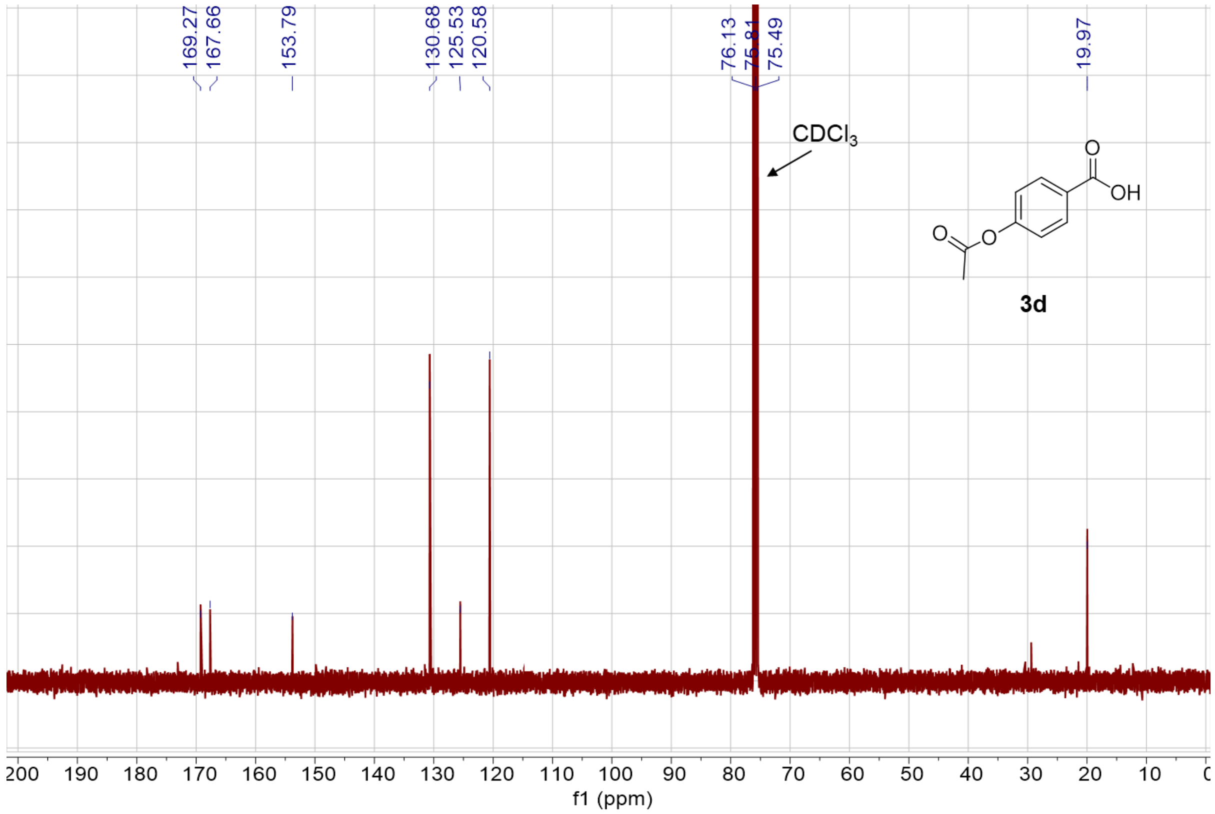

Figure S183: $^{13}C\{^{1}H\}$ NMR spectrum of the isolated **3d** in $CDCl_3$ as the solvent.

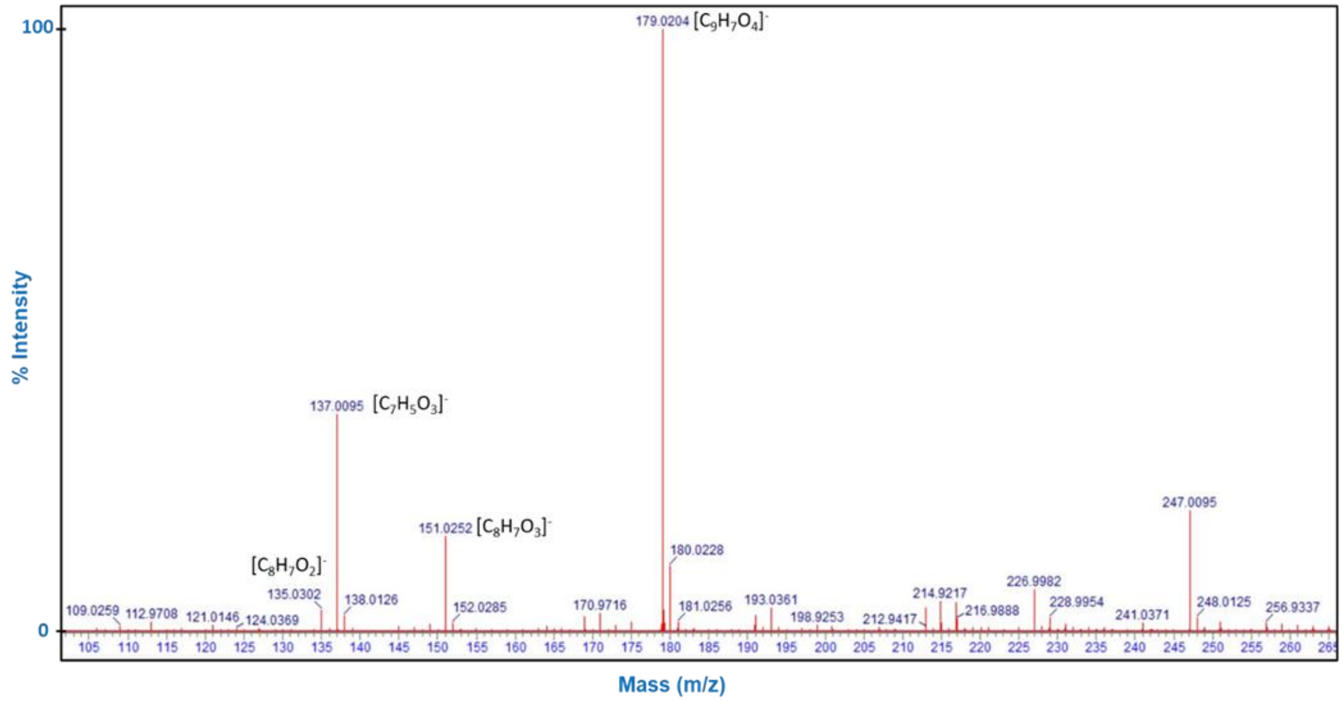

Figure S184: HRMS of the isolated **3d**.

4-methylbenzoic acid (**3e**)

^{1}H NMR (400 MHz, CHLOROFORM-*D*) δ 2.43 (s, 3 H), 7.28 (d, *J* = 7.3 Hz, 2 H), 8.00 (d, *J* = 8.0 Hz, 2 H).

^{13}C{^{1}H} NMR (100 MHz, CHLOROFORM-*D*) δ 20.6, 127.1, 128.0, 129.1, 143.5, 170.8.

HRMS (ESI-QTOF, m/z) calculated for $[C_8H_7O_2]^-$ *m/z* = 135.0293, found 135.0295.

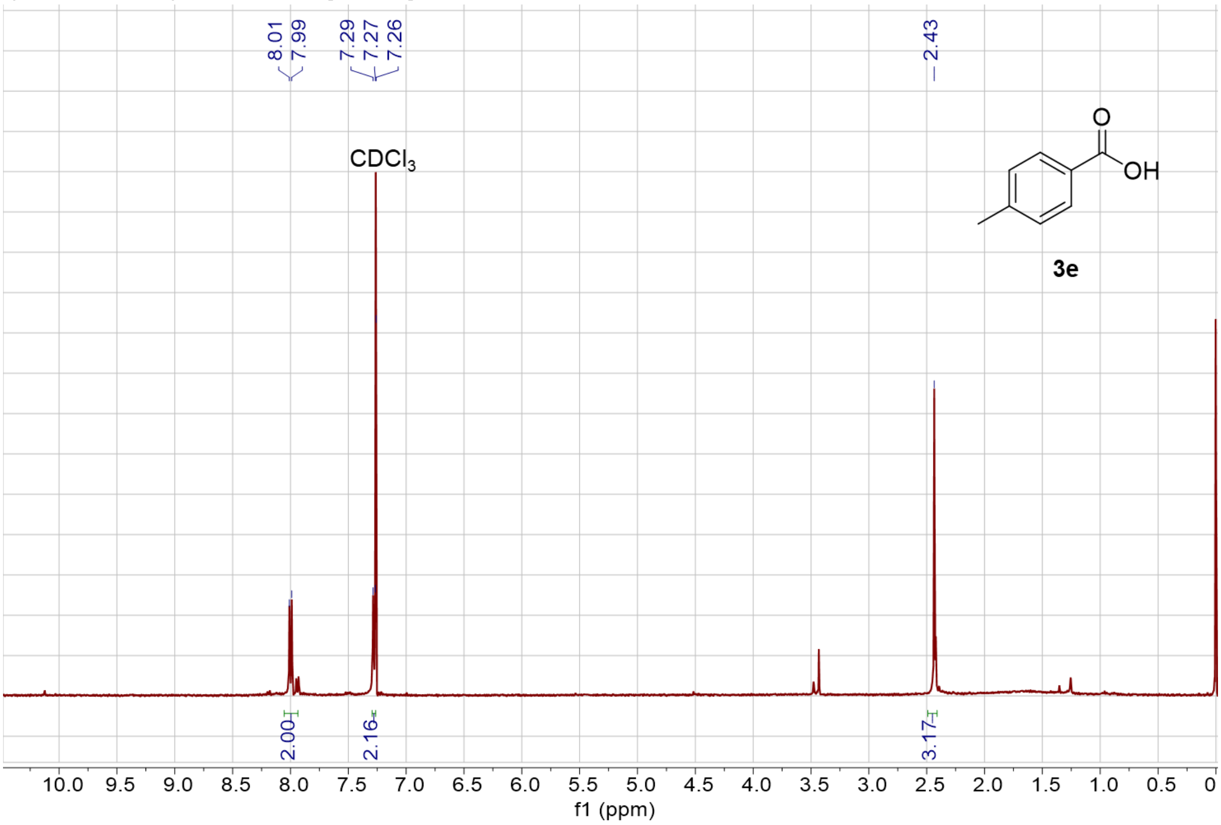

Figure S185: ^{1}H NMR spectrum of the isolated **3e** in $CDCl_3$ as the solvent.

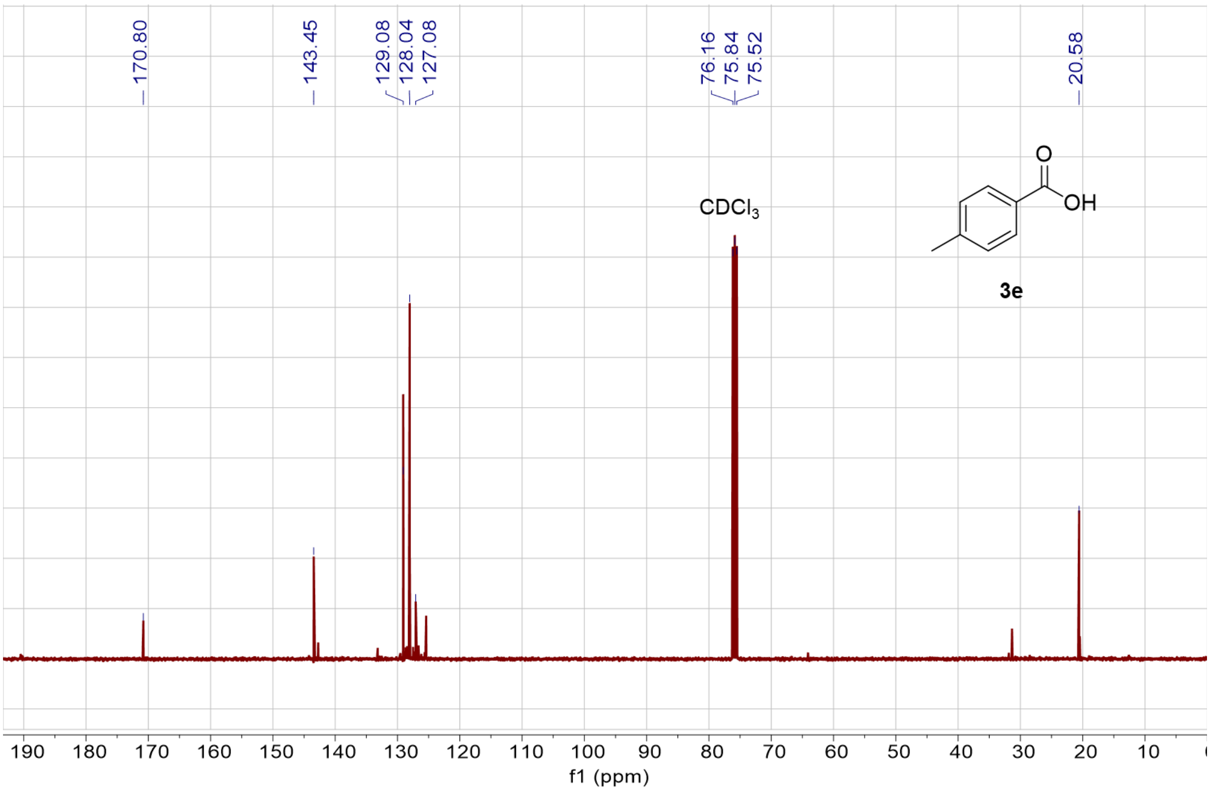

Figure S186: ^{13}C{^{1}H} NMR spectrum of the isolated **3e** in $CDCl_3$ as the solvent.

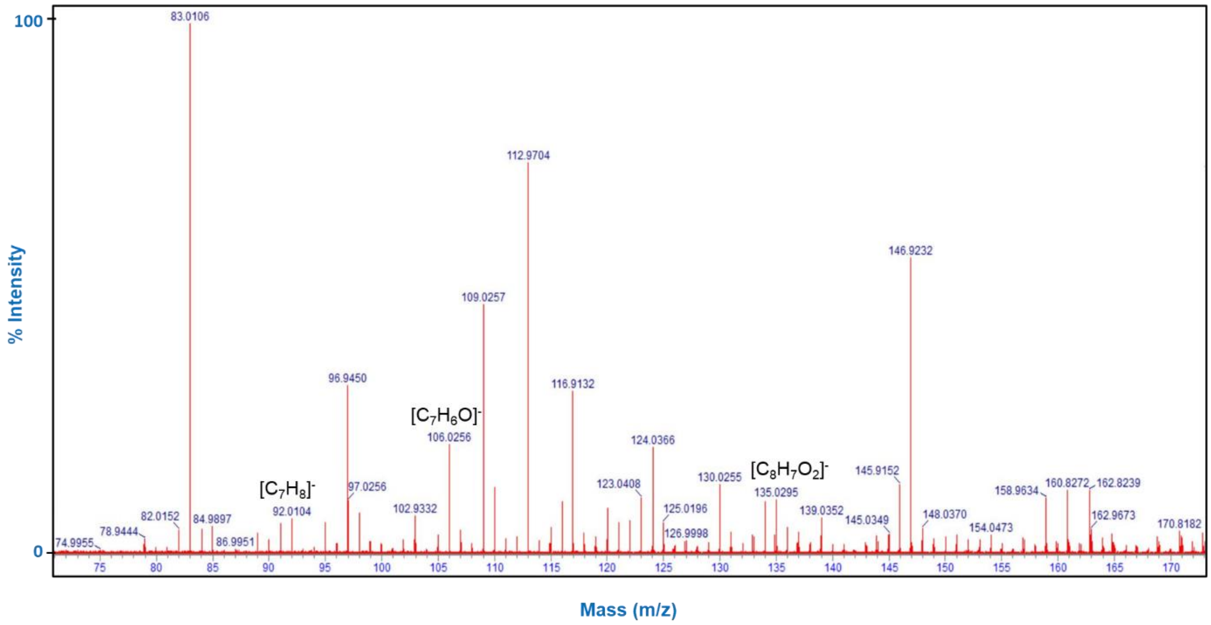

Figure S187: HRMS of the isolated **3e**.

2-methoxybenzoic acid (**3f**)

^{1}H NMR (400 MHz, CHLOROFORM-*D*) δ 4.08 (s, 3 H), 7.06 (d, *J* = 8.5 Hz, 1 H), 7.11 – 7.17 (m, 1 H), 7.54 – 7.61 (m, 1 H), 8.18 (d, *J* = 7.8 Hz, 1 H).

^{13}C{^{1}H} NMR (100 MHz, CHLOROFORM-*D*) δ 55.5, 110.5, 116.4, 121.1, 132.7, 134.0, 156.9, 164.4.

HRMS (ESI-QTOF, m/z) calculated for $[C_8H_7O_3]^-$ *m/z* = 151.0243, found 151.0251.

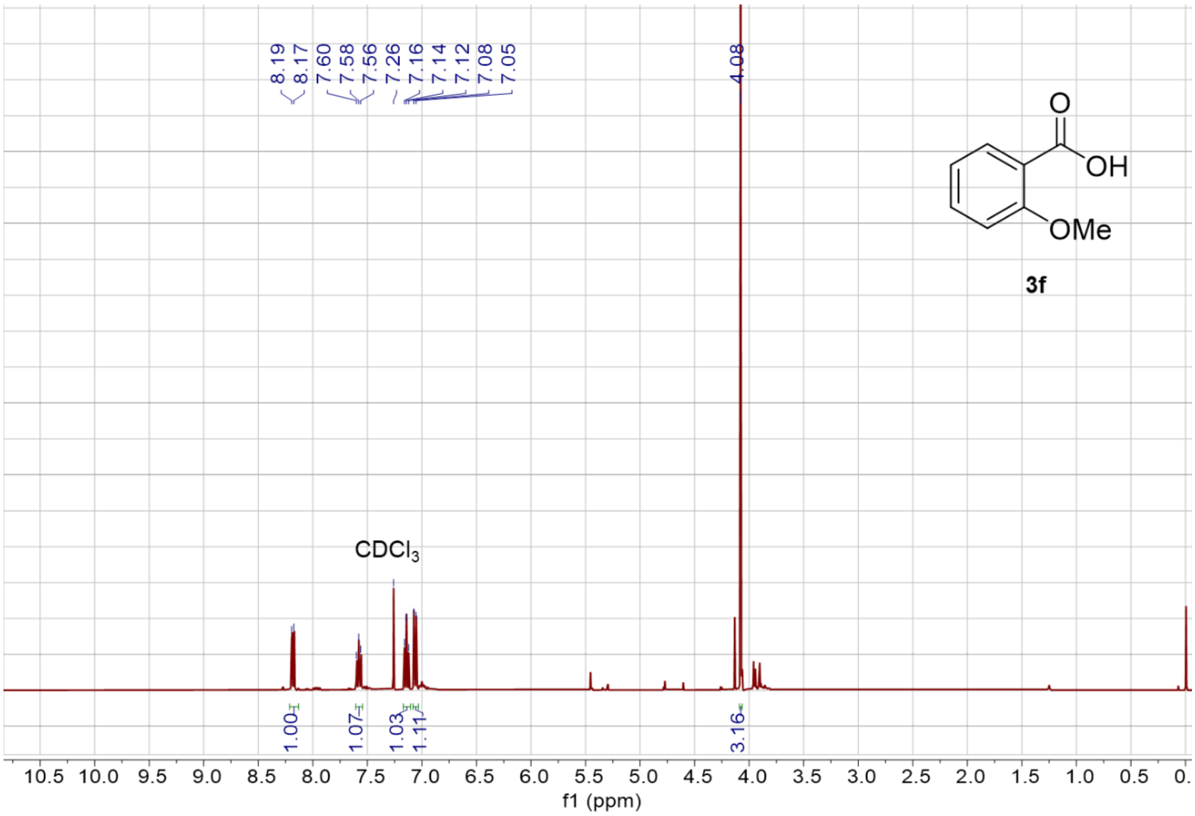

Figure S188: ^{1}H NMR spectrum of the isolated **3f** in $CDCl_3$ as the solvent.

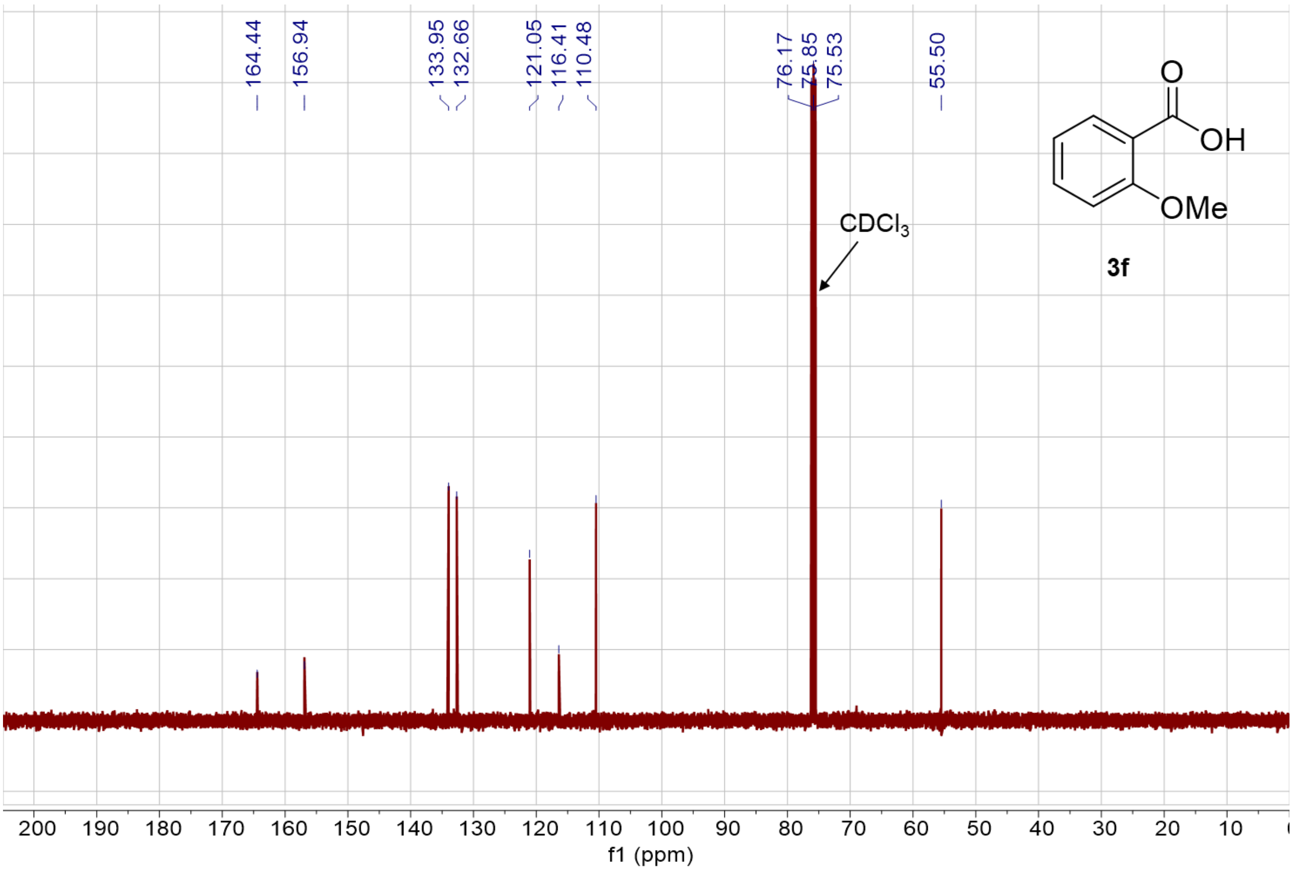

Figure S189: $^{13}C\{^{1}H\}$ NMR spectrum of the isolated **3f** in $CDCl_3$ as the solvent.

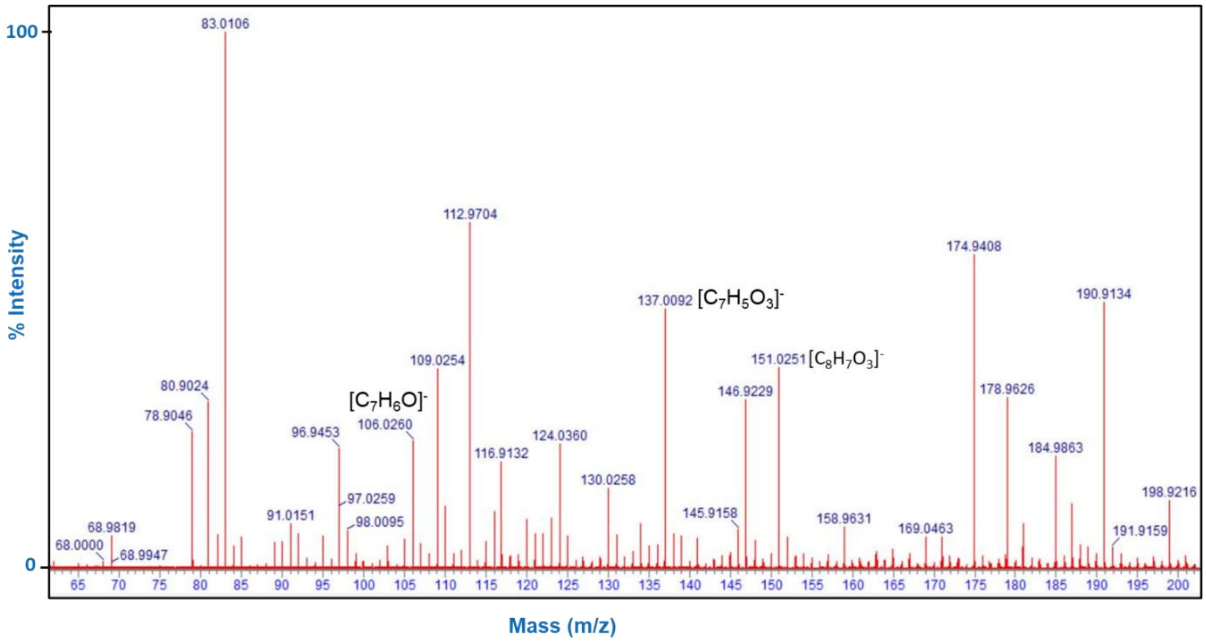

Figure S190: HRMS of the isolated **3f**.

4-fluorobenzoic acid (**3g**)

^{1}H NMR (400 MHz, CHLOROFORM-*D*) δ 7.15 (t, *J* = 8.6 Hz, 2 H), 8.14 (dd, *J* = 8.9, 5.4 Hz, 2 H).

^{13}C{^{1}H} NMR (100 MHz, CHLOROFORM-*D*) δ 114.6 (d, ^2J C-F coupling = 22.1 Hz), 124.3, 131.7 (d, ^3J C-F coupling = 9.5 Hz), 165.2 (d, ^1J C-F coupling = 255.1 Hz), 169.1.

^{19}F{^{1}H} NMR (400 MHz, CHLOROFORM-*D*) δ -104.0.

HRMS (ESI-QTOF, m/z) calculated for $[C_7H_4FO_2]^-$ *m/z* = 139.0043, found 139.0047.

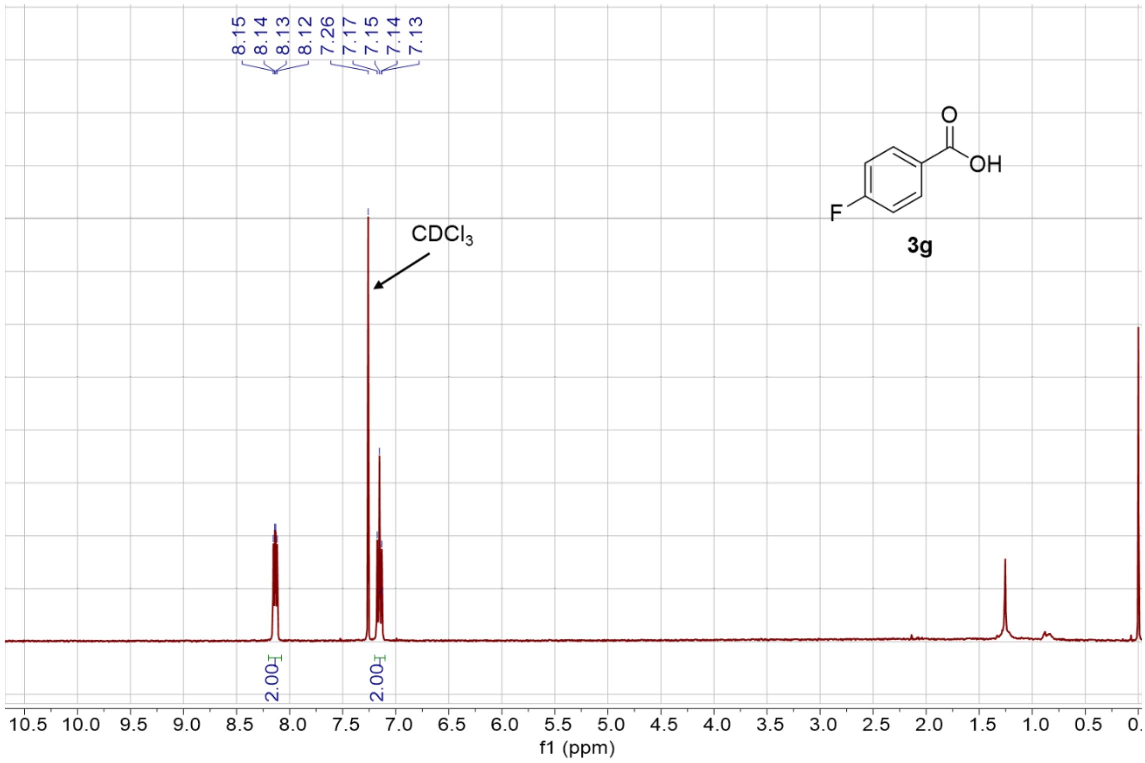

Figure S191: ^{1}H NMR spectrum of the isolated **3g** in $CDCl_3$ as the solvent.

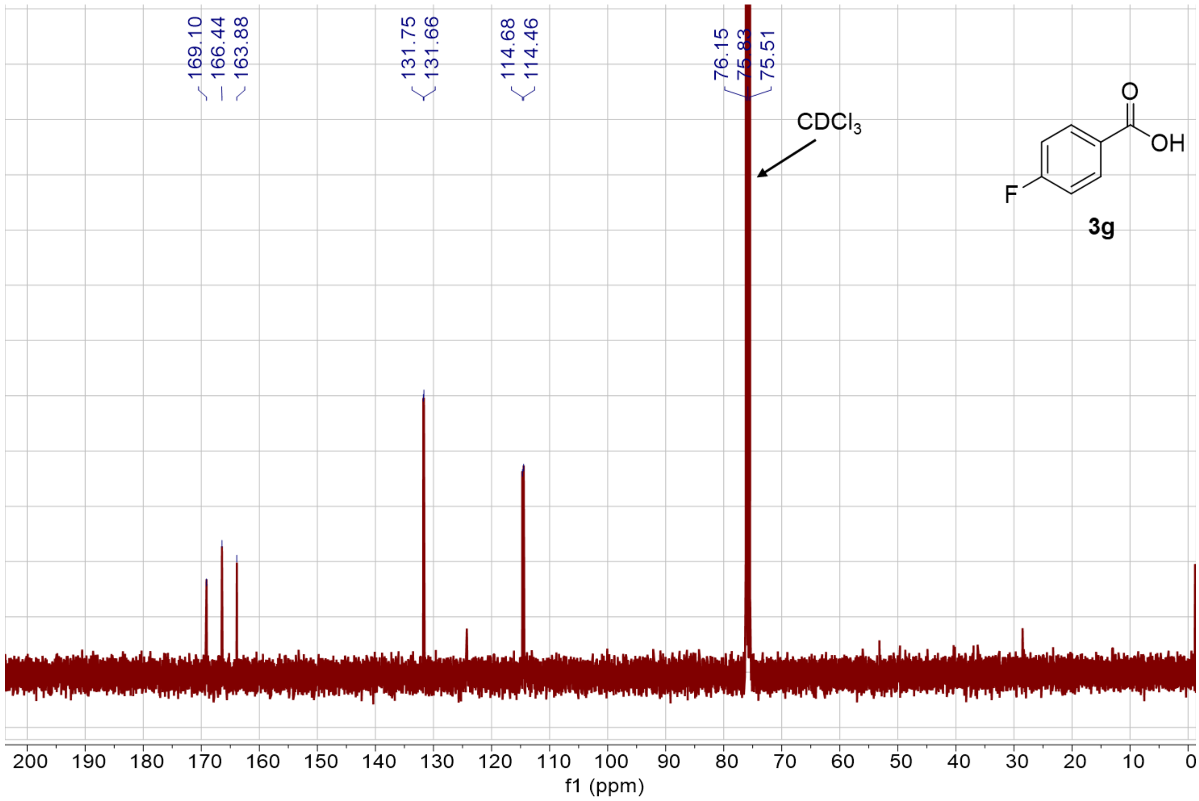

Figure S192: ^{13}C{^{1}H} NMR spectrum of the isolated **3g** in $CDCl_3$ as the solvent.

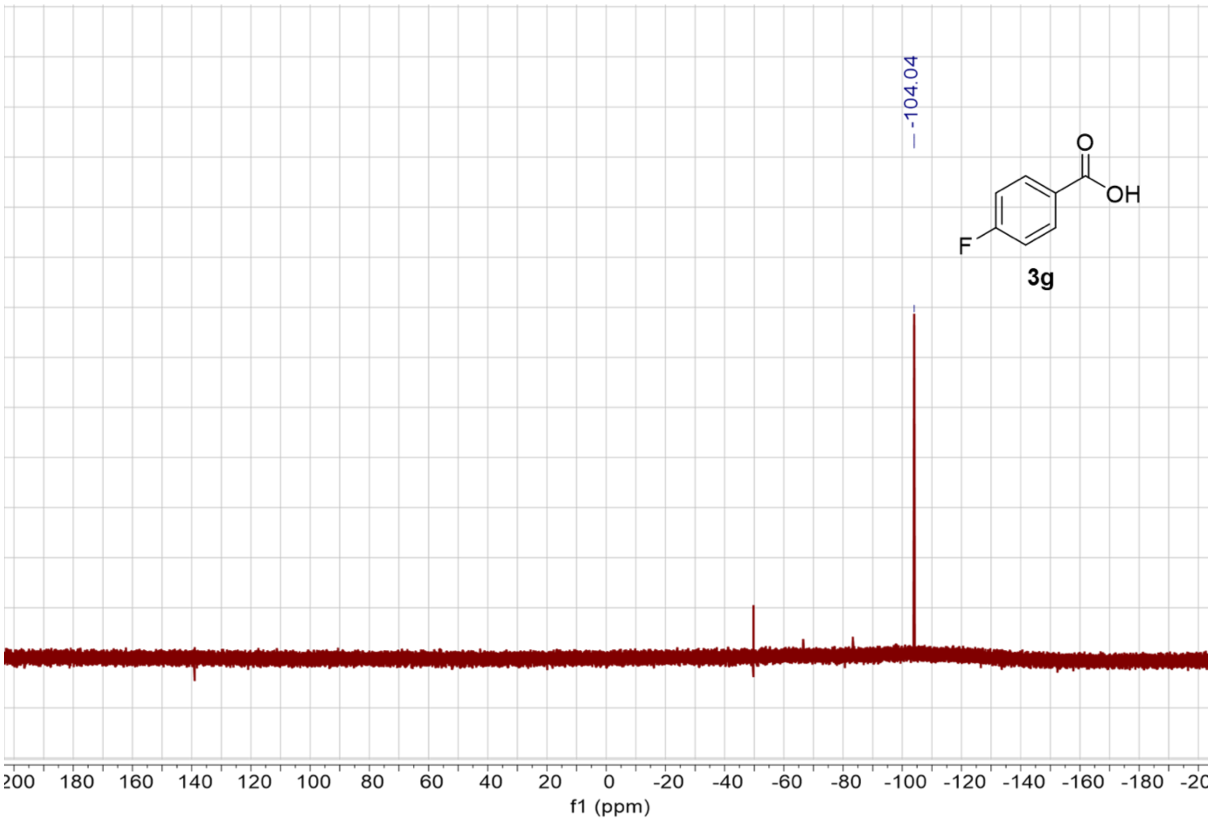

Figure S193: $^{19}F\{^1H\}$ NMR spectrum of the isolated **3g** in $CDCl_3$ as the solvent.

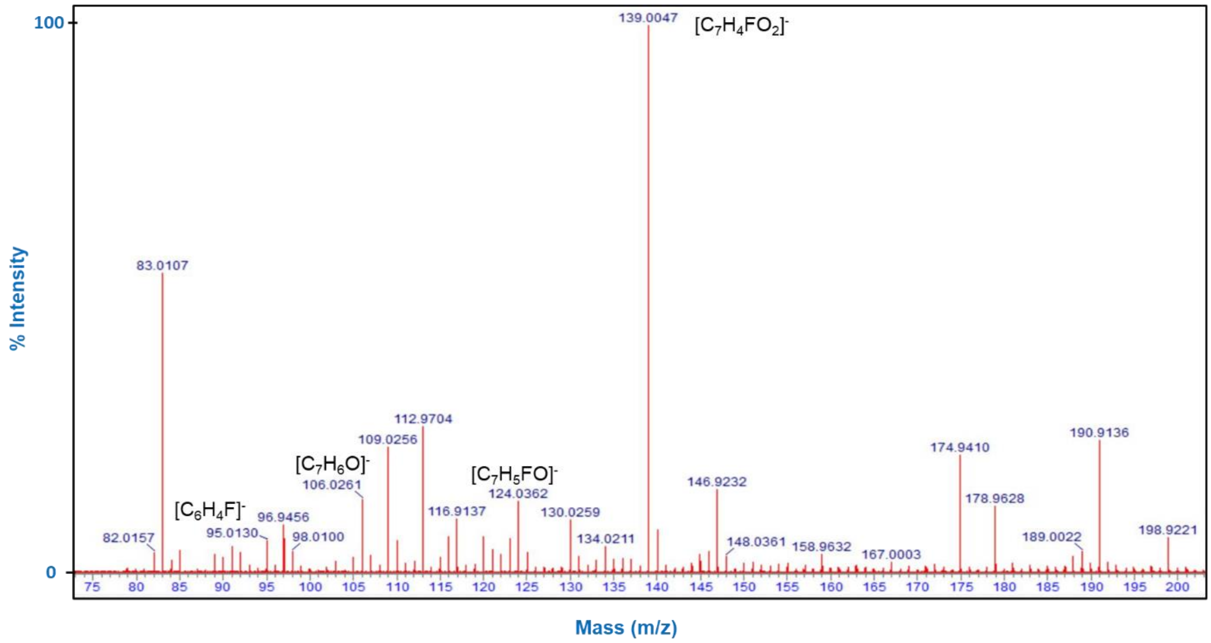

Figure S194: HRMS of the isolated **3g**.

4-chlorobenzoic acid (**3h**)
^{1}H NMR (400 MHz, CHLOROFORM-*D*) δ 7.46 (d, *J* = 8.6 Hz, 2 H), 8.04 (d, *J* = 8.6 Hz, 2 H).
^{13}C{^{1}H} NMR (100 MHz, CHLOROFORM-*D*) δ 126.3, 127.7, 130.3, 139.1, 168.5.
HRMS (ESI-QTOF, m/z) calculated for $[C_7H_4ClO_2]^-$ *m/z* = 154.9747, found 154.9754.

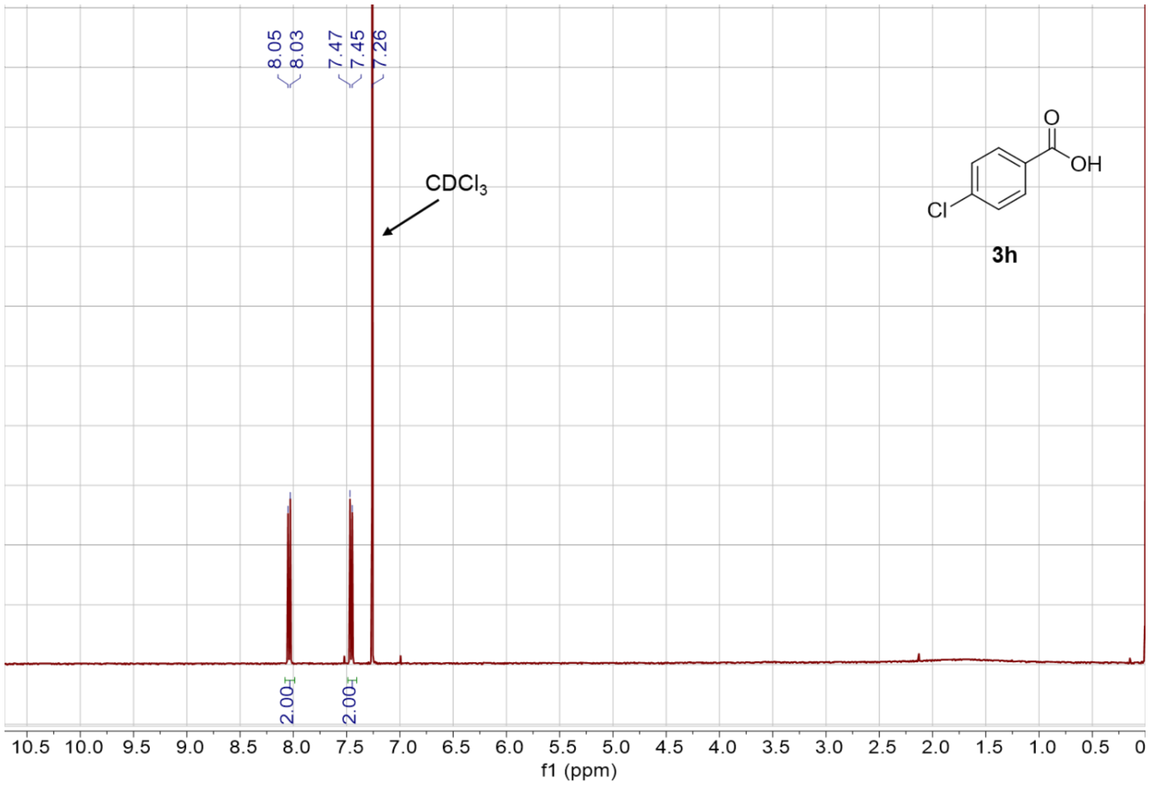

Figure S195: ^{1}H NMR spectrum of the isolated **3h** in $CDCl_3$ as the solvent.

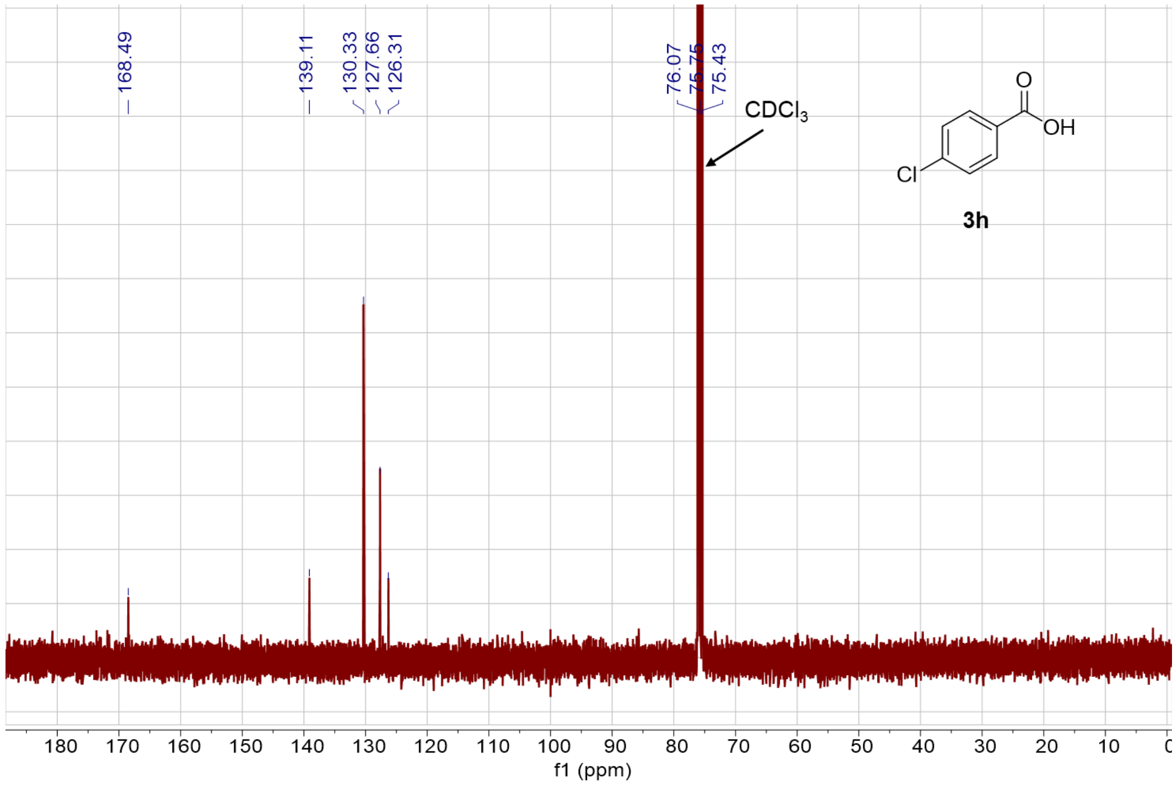

Figure S196: ^{13}C{^{1}H} NMR spectrum of the isolated **3h** in $CDCl_3$ as the solvent.

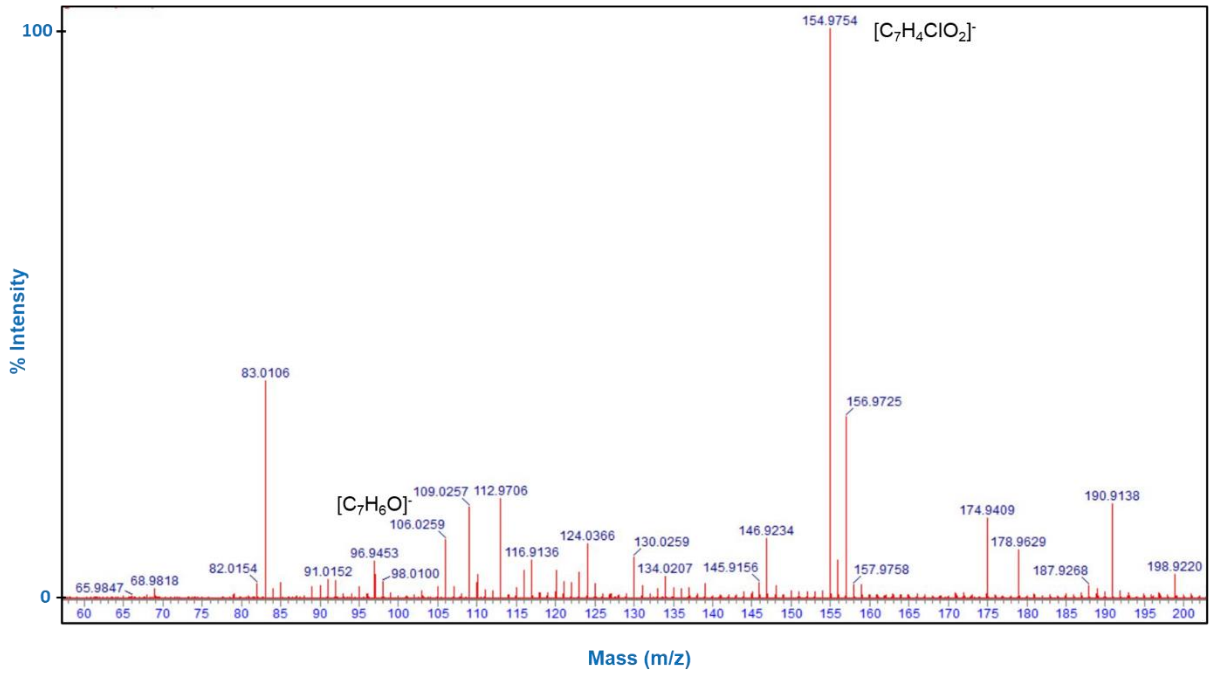

Figure S197: HRMS of the isolated **3h**.

4-bromobenzoic acid (**3i**)

^{1}H NMR (500 MHz, CHLOROFORM-*D*) δ 7.62 (d, *J* = 8.6 Hz, 1H), 7.96 (d, *J* = 8.6 Hz, 1H).

^{13}C{^{1}H} NMR (125 MHz, DMSO-*D*$_6$) δ 127.0, 130.2, 131.5, 131.9, 166.8.

HRMS (ESI-QTOF, m/z) calculated for $[C_7H_4BrO_2]^-$ *m/z* = 198.9242, found 198.9257.

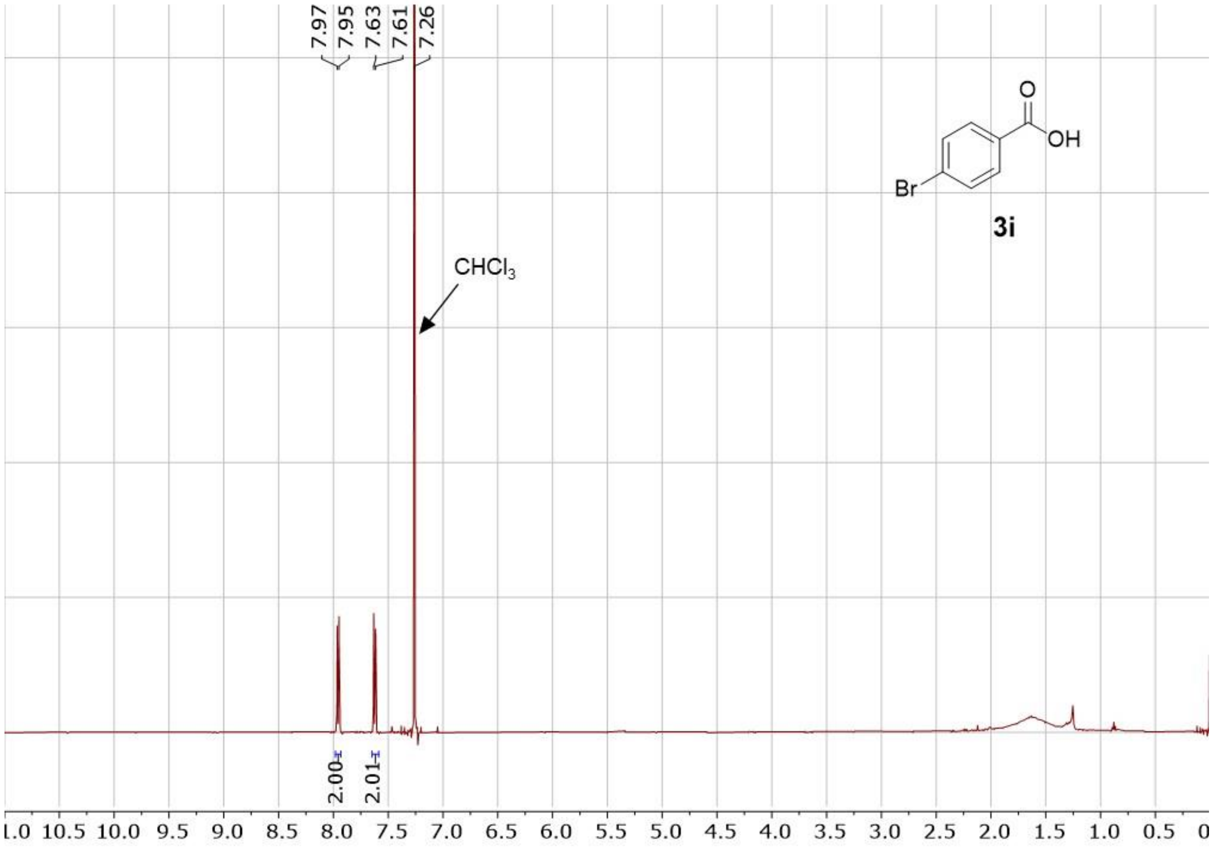

Figure S198: ^{1}H NMR spectrum of the isolated **3i** in $CDCl_3$ as the solvent.

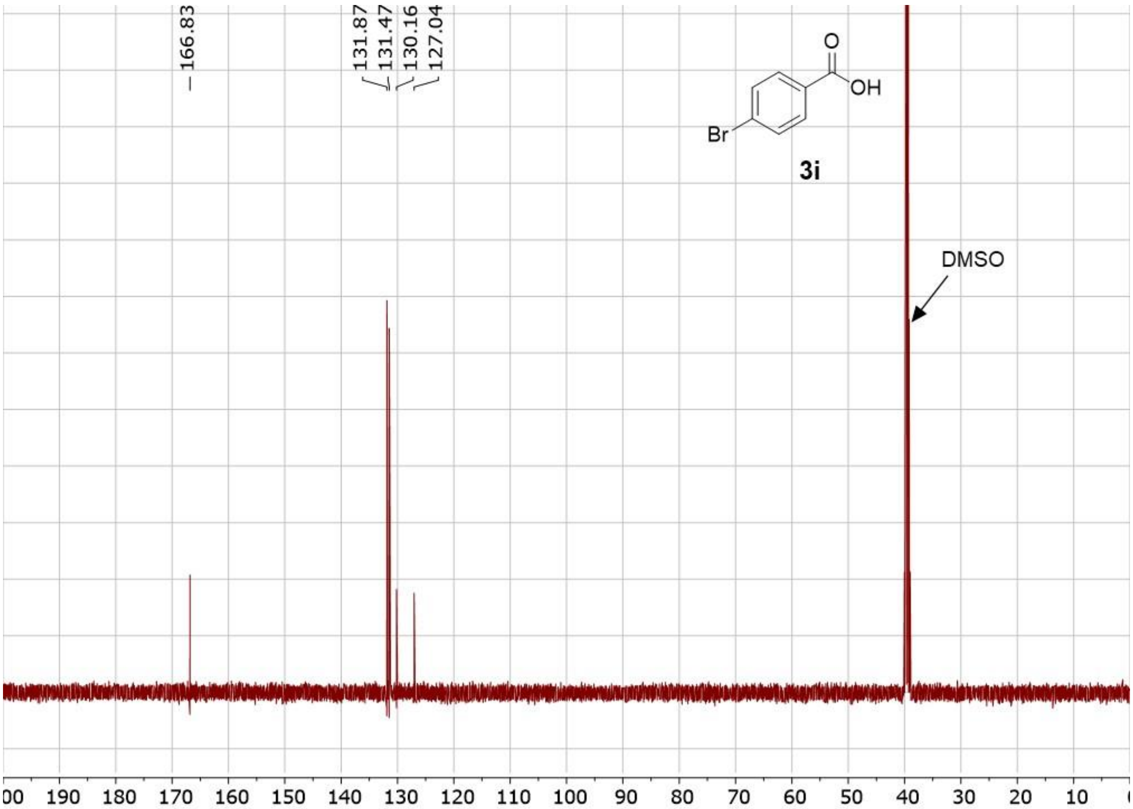

Figure S199: $^{13}C\{^1H\}$ NMR spectrum of the isolated **3i** in DMSO-d_6 as the solvent.

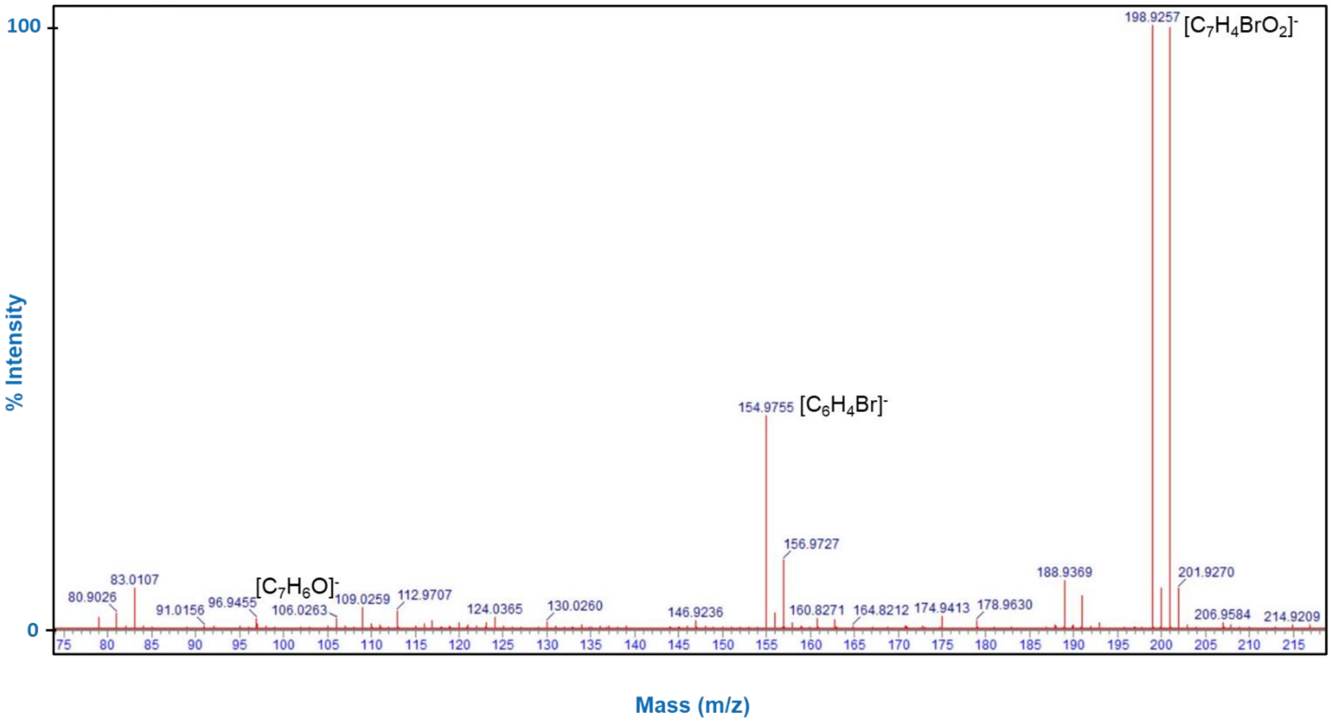

Figure S200: HRMS of the isolated **3i**.

2-chlorobenzoic acid (**3j**)

^{1}H NMR (400 MHz, CHLOROFORM-*D*) δ 7.36 (ddd, *J* = 8.3, 6.2, 2.4 Hz, 1 H), 7.42 – 7.56 (m, 2 H), 8.03 (d, *J* = 7.7 Hz, 1 H).

^{13}C{^{1}H} NMR (100 MHz, CHLOROFORM-*D*) δ 125.5, 127.2, 130.3, 131.2, 132.3, 133.5, 169.3.

HRMS (ESI-QTOF, m/z) calculated for $[C_7H_4ClO_2]^-$ *m/z* = 154.9747, found 154.9754.

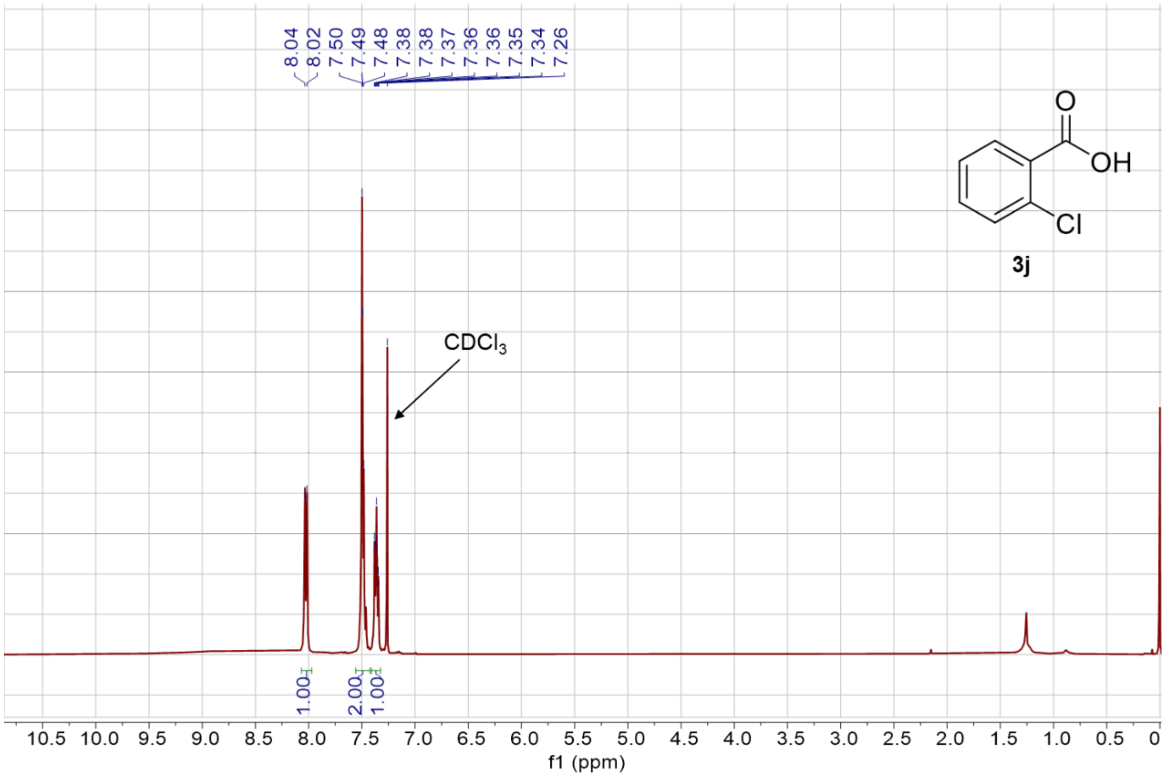

Figure S201: ^{1}H NMR spectrum of the isolated **3j** in $CDCl_3$ as the solvent.

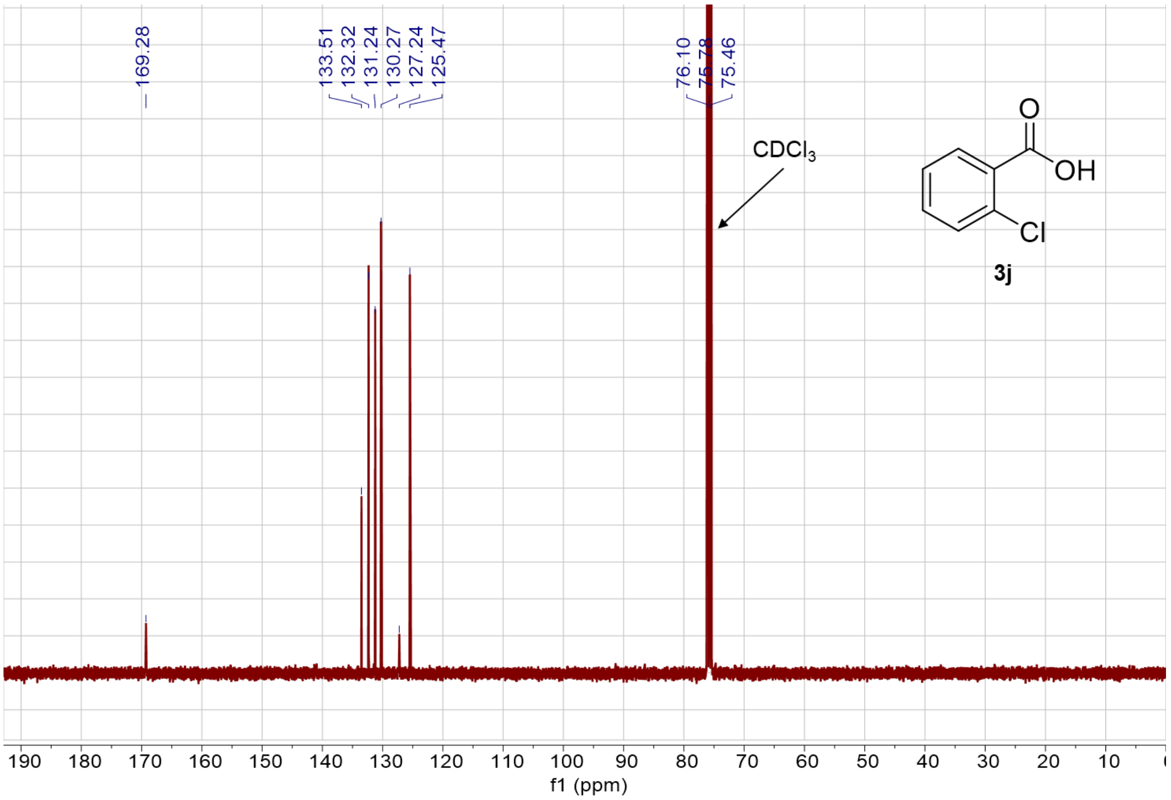

Figure S202: ^{13}C{^{1}H} NMR spectrum of the isolated **3j** in $CDCl_3$ as the solvent.

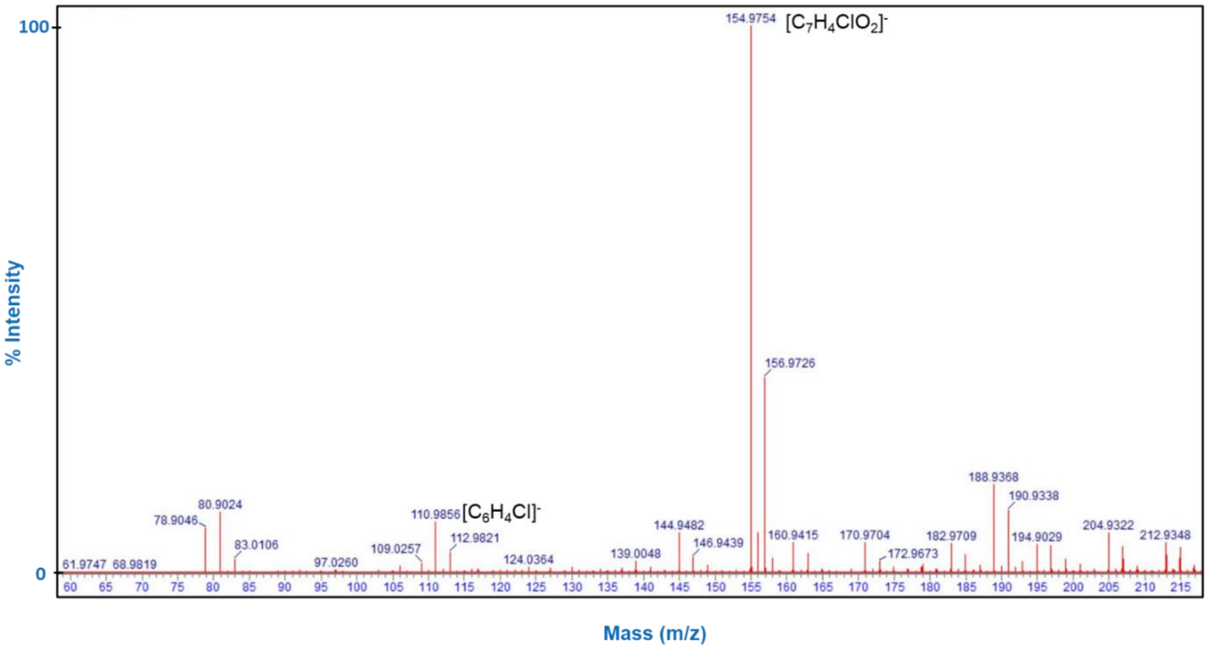

Figure S203: HRMS of the isolated **3j**.

4-(methoxycarbonyl)benzoic acid (**3k**)
^{1}H NMR (400 MHz, CHLOROFORM-*D*) δ 3.96 (s, 3 H), 8.42 – 7.97 (m, 4 H).
^{13}C{^{1}H} NMR (100 MHz, CHLOROFORM-*D*) δ 51.3, 128.5, 129.0, 131.7, 133.5, 165.0, 169.0.
HRMS (ESI-QTOF, m/z) calculated for $[C_9H_7O_4]^-$ *m/z* = 179.0344, found 179.0200.

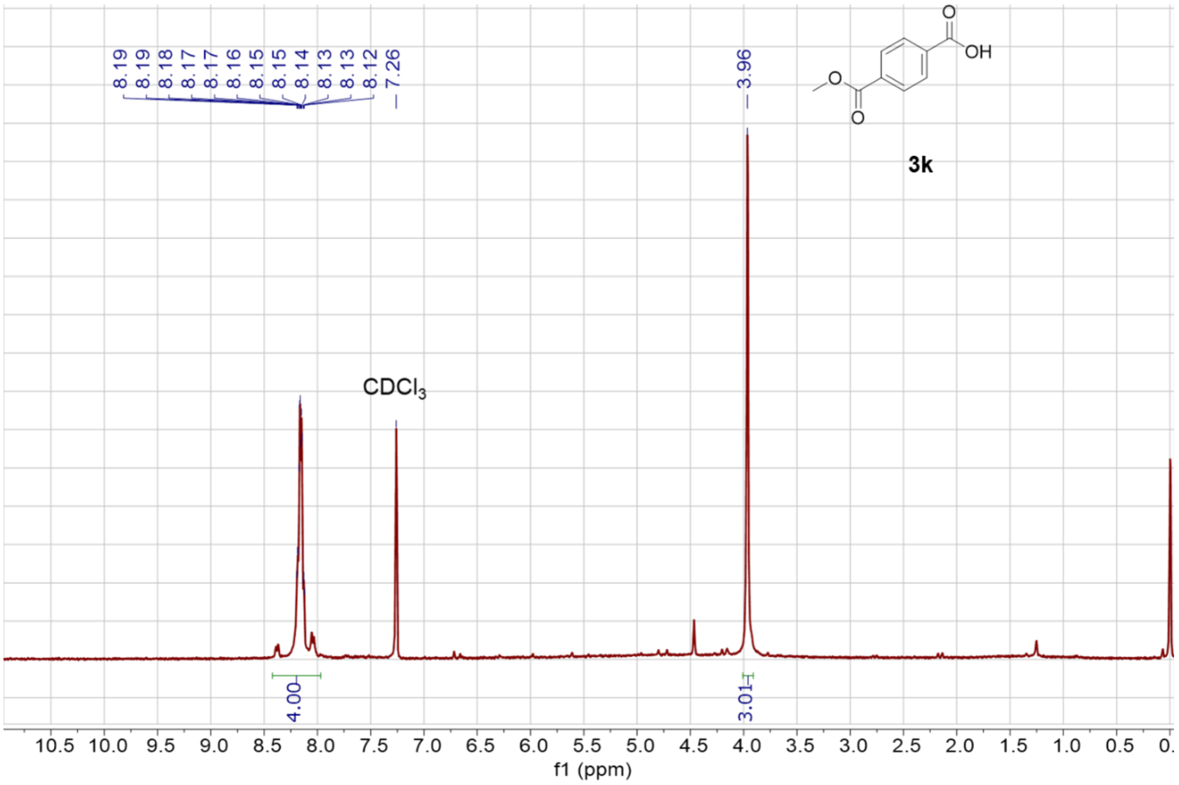

Figure S204: ^{1}H NMR spectrum of the isolated **3k** in $CDCl_3$ as the solvent.

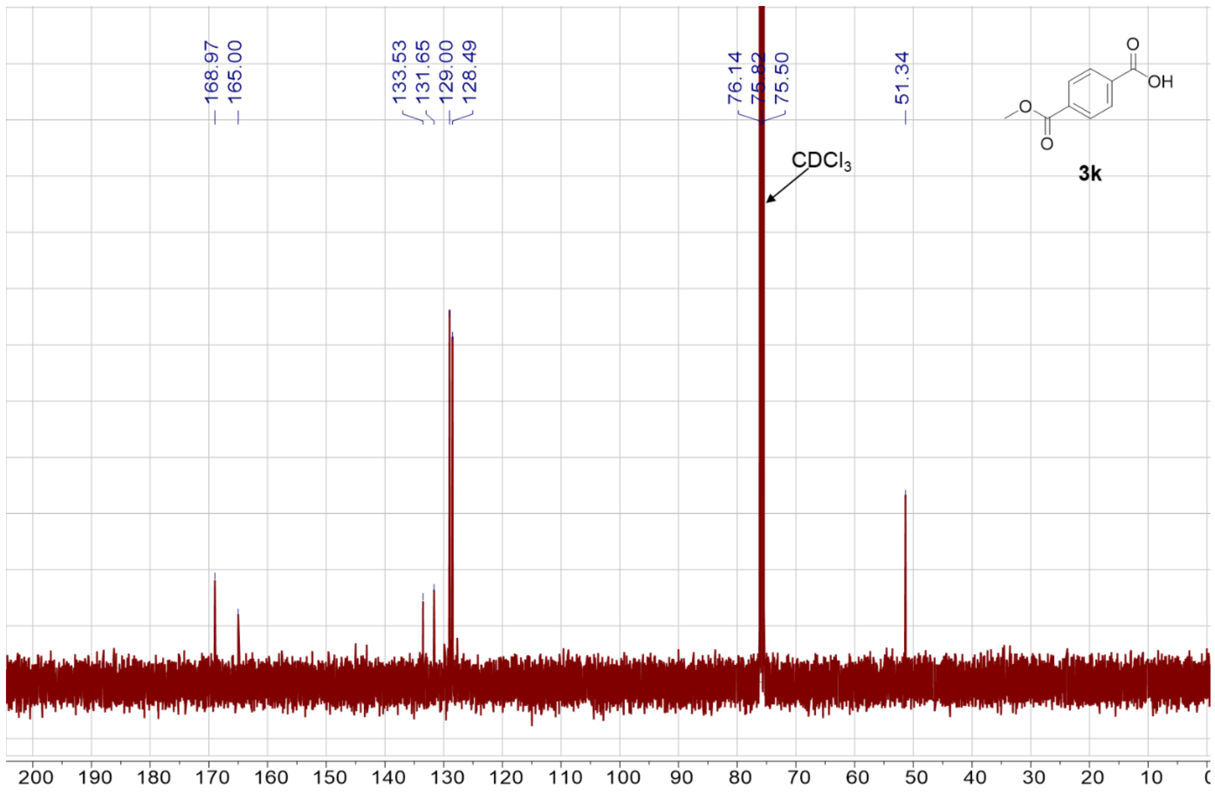

Figure S205: $^{13}C\{^{1}H\}$ NMR spectrum of the isolated **3k** in $CDCl_3$ as the solvent.

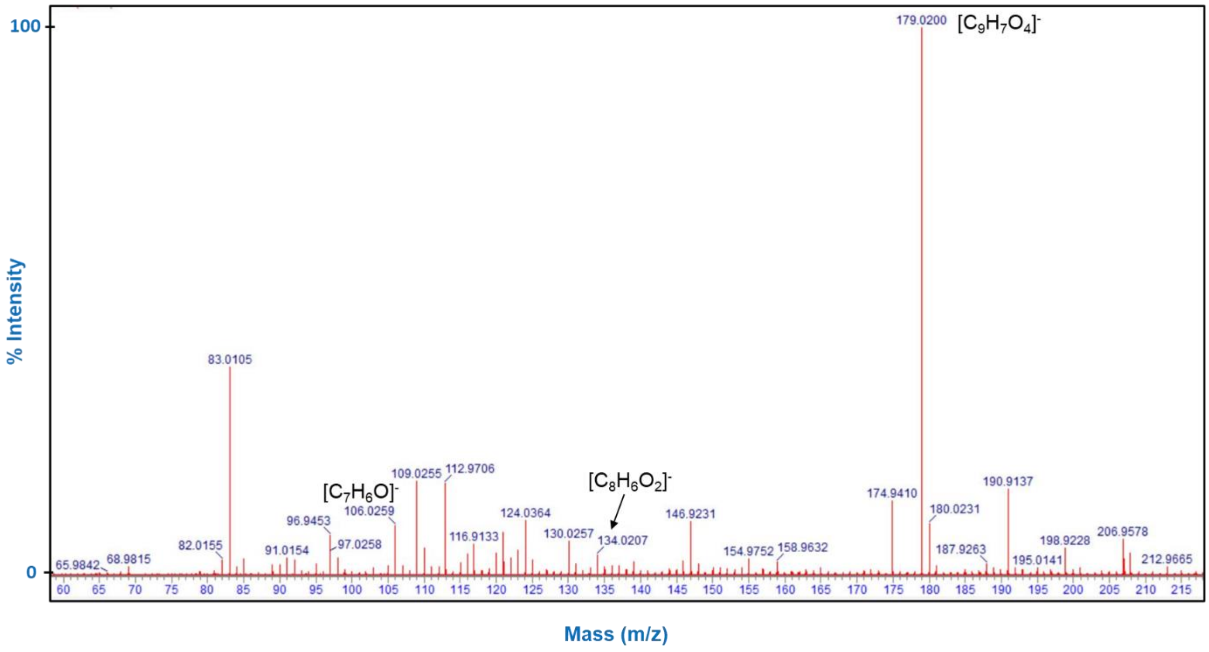

Figure S206: HRMS of the isolated **3k**.

4-(trifluoromethyl)benzoic acid (**3l**)
^{1}H NMR (400 MHz, CHLOROFORM-*D*) δ 7.76 (d, *J* = 7.1 Hz, 2 H), 8.23 (d, *J* = 7.9 Hz, 2 H).
$^{13}C\{^{1}H\}$ NMR (100 MHz, CHLOROFORM-*D*) δ 123.7, 124.4 (q, *J* = 3.7 Hz, CF_3), 129.5, 133.9, 134.3, 168.2.
$^{19}F\{^{1}H\}$ NMR (400 MHz, CHLOROFORM-*D*) δ -63.1.
HRMS (ESI-QTOF, m/z) calculated for $[C_8H_4F_3O_2]^-$ *m/z* = 188.9999, found 189.0020.

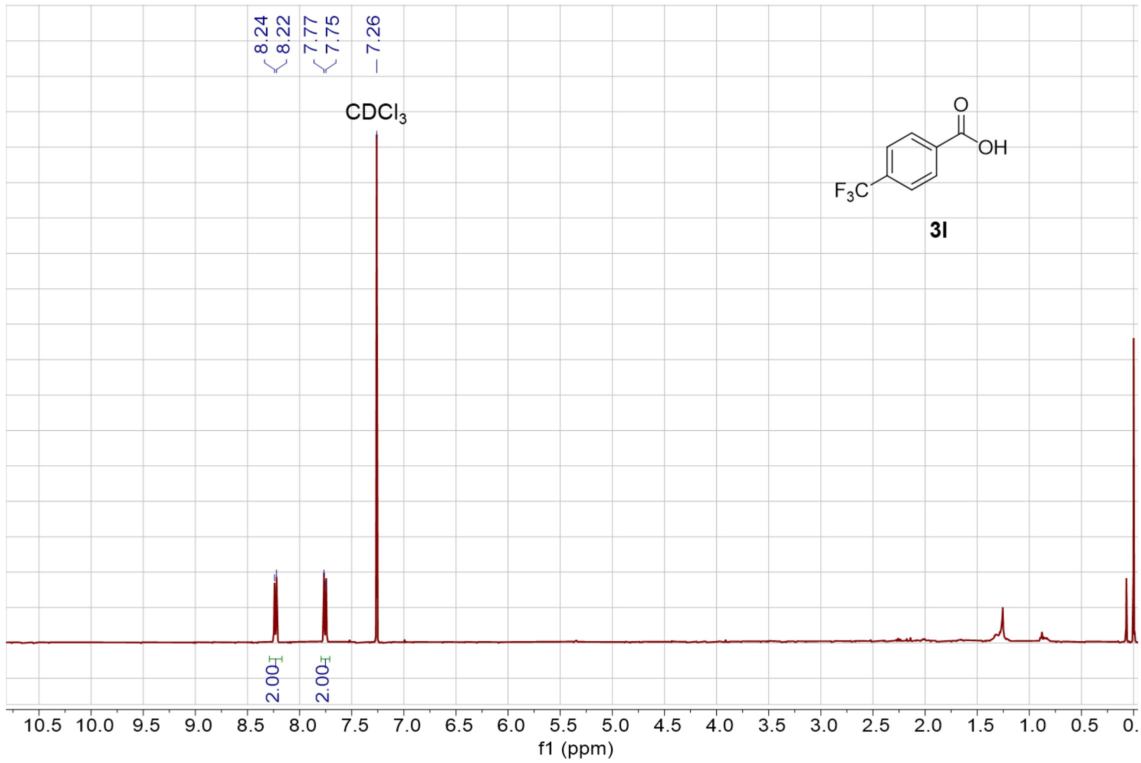

Figure S207: ^{1}H NMR spectrum of the isolated **3l** in $CDCl_3$ as the solvent.

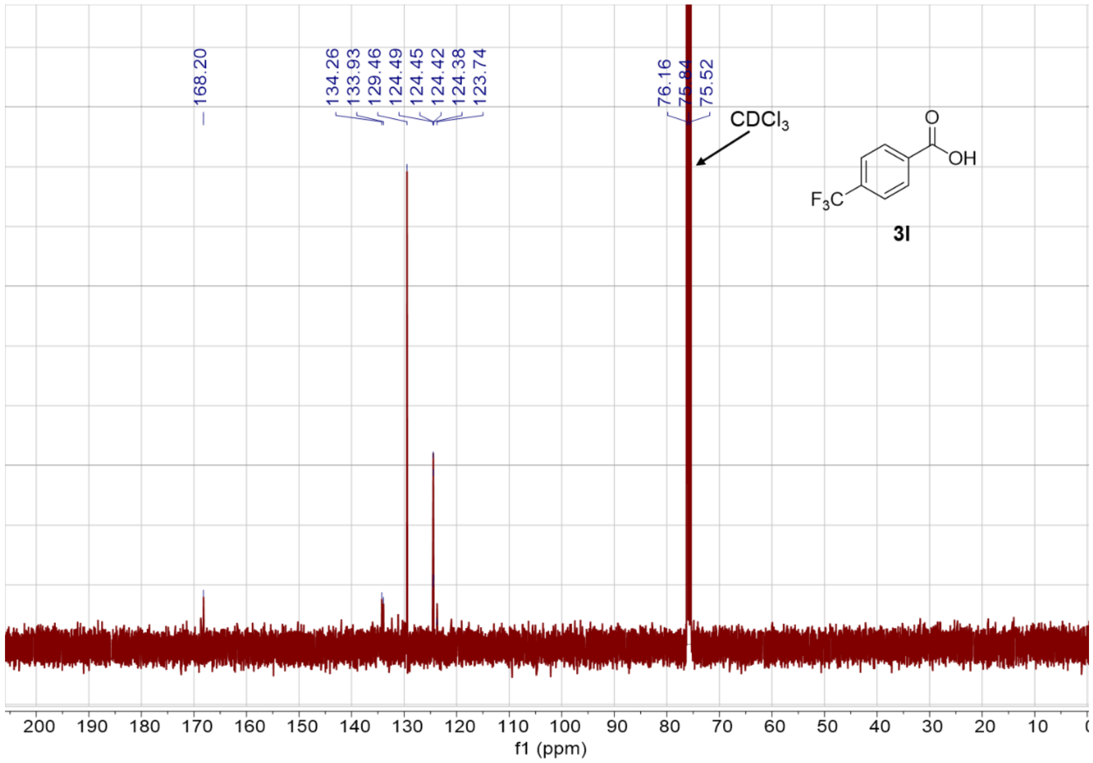

Figure S208: $^{13}C\{^{1}H\}$ NMR spectrum of the isolated **3l** in $CDCl_3$ as the solvent.

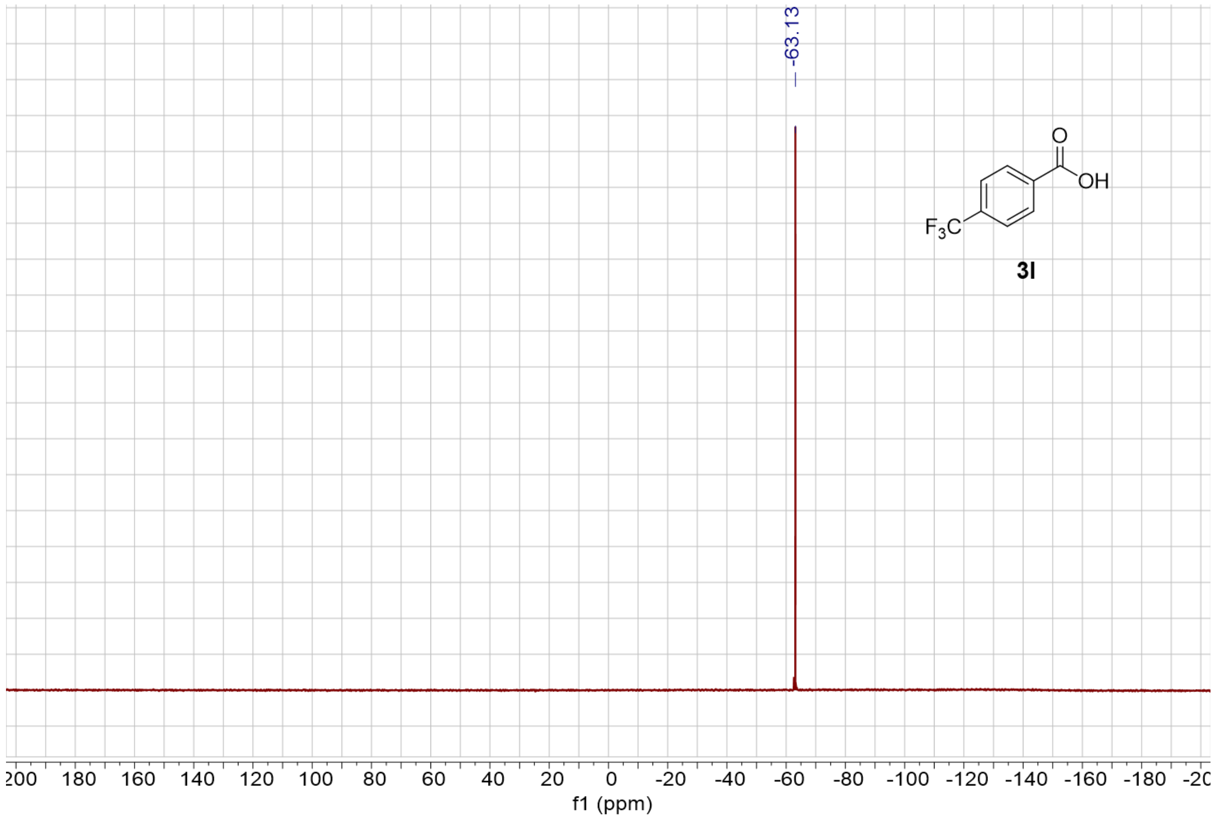

Figure S209: $^{19}F\{^{1}H\}$ NMR spectrum of the isolated **3l** in $CDCl_3$ as the solvent.

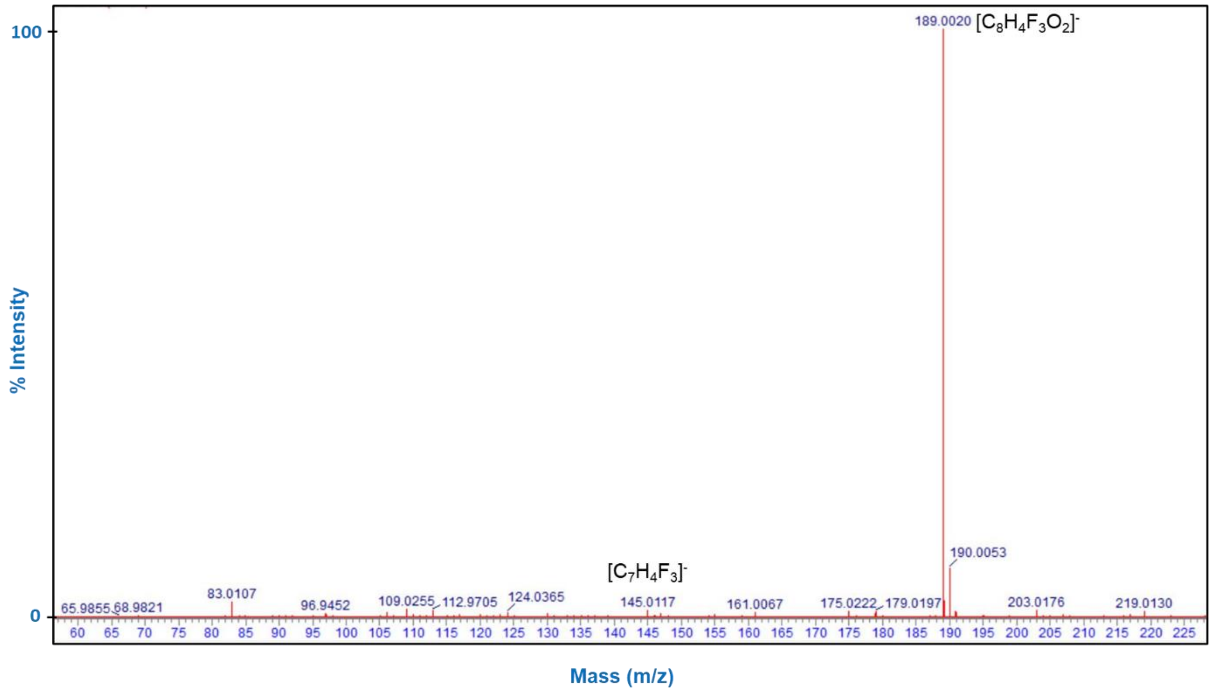

Figure S210: HRMS of the isolated **3l**.

4-cyanobenzoic acid (**3m**)

^{1}H NMR (400 MHz, CHLOROFORM-*D*) δ 7.79 (d, *J* = 8.0 Hz, 2 H), 8.20 (d, *J* = 8.1 Hz, 2 H).

^{13}C{^{1}H} NMR (100 MHz, CHLOROFORM-*D*) δ 116.0, 116.5, 129.4, 131.1, 131.6, 167.9.

HRMS (ESI-QTOF, m/z) calculated for $[C_8H_4NO_2]^-$ *m/z* = 146.0089, found 146.0095.

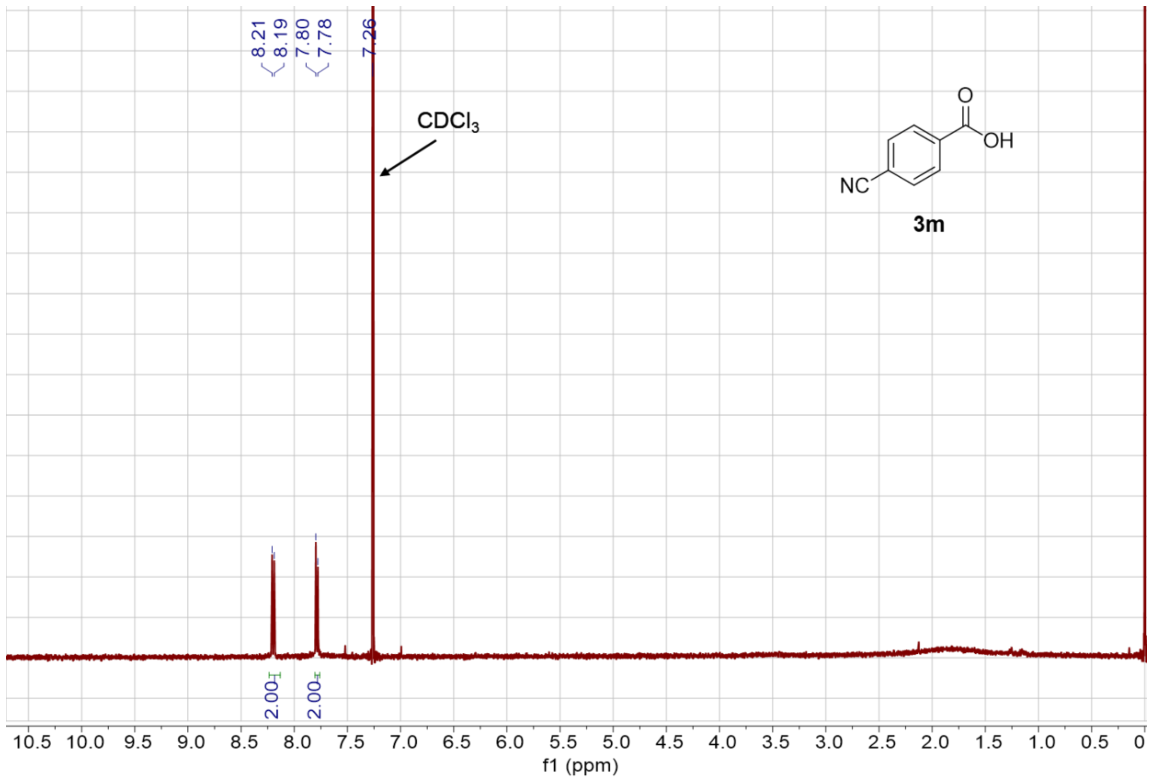

Figure S211: ^{1}H NMR spectrum of the isolated **3m** in $CDCl_3$ as the solvent.

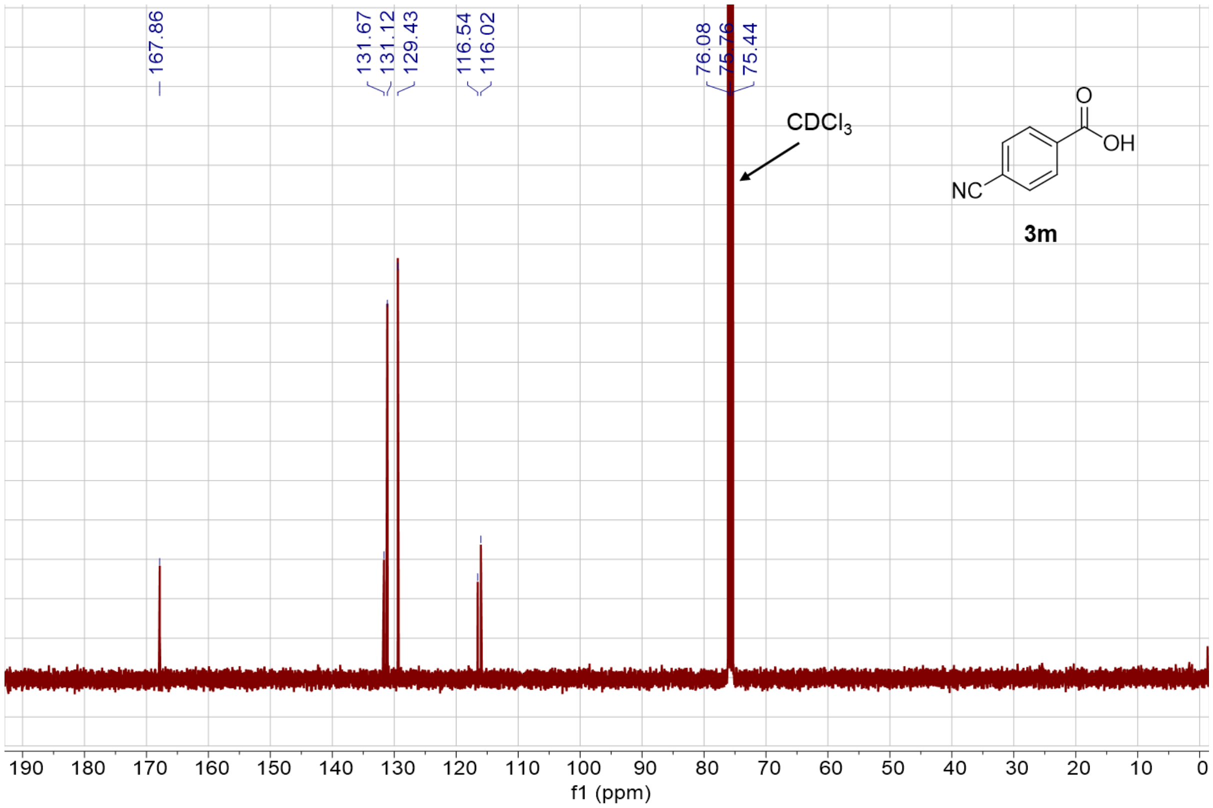

Figure S212: ^{13}C{^{1}H} NMR spectrum of the isolated **3m** in $CDCl_3$ as the solvent.

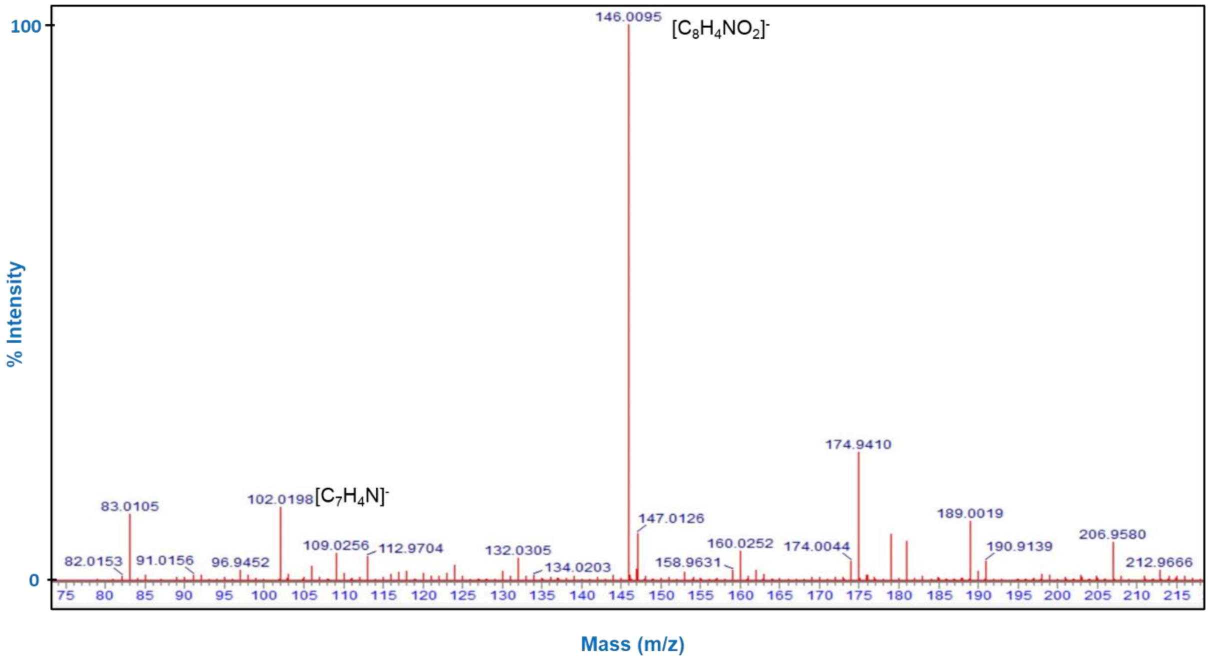

Figure S213: HRMS of the isolated **3m.**

3-nitrobenzoic acid (**3n**)
^{1}H NMR (400 MHz, CHLOROFORM-*D*) δ 7.72 (t, *J* = 8.0 Hz, 1 H), 8.45 (dt, *J* = 7.9, 1.3 Hz, 1 H), 8.46 – 8.53 (m, 1 H), 8.96 (s, 1 H).
^{13}C{^{1}H} NMR (100 MHz, CHLOROFORM-*D*) δ 124.1, 127.1, 128.7, 129.7, 134.6, 147.2, 168.5.
HRMS (ESI-QTOF, m/z) calculated for $[C_7H_4NO_4]^-$ *m/z* = 166.0140, found 166.0012.

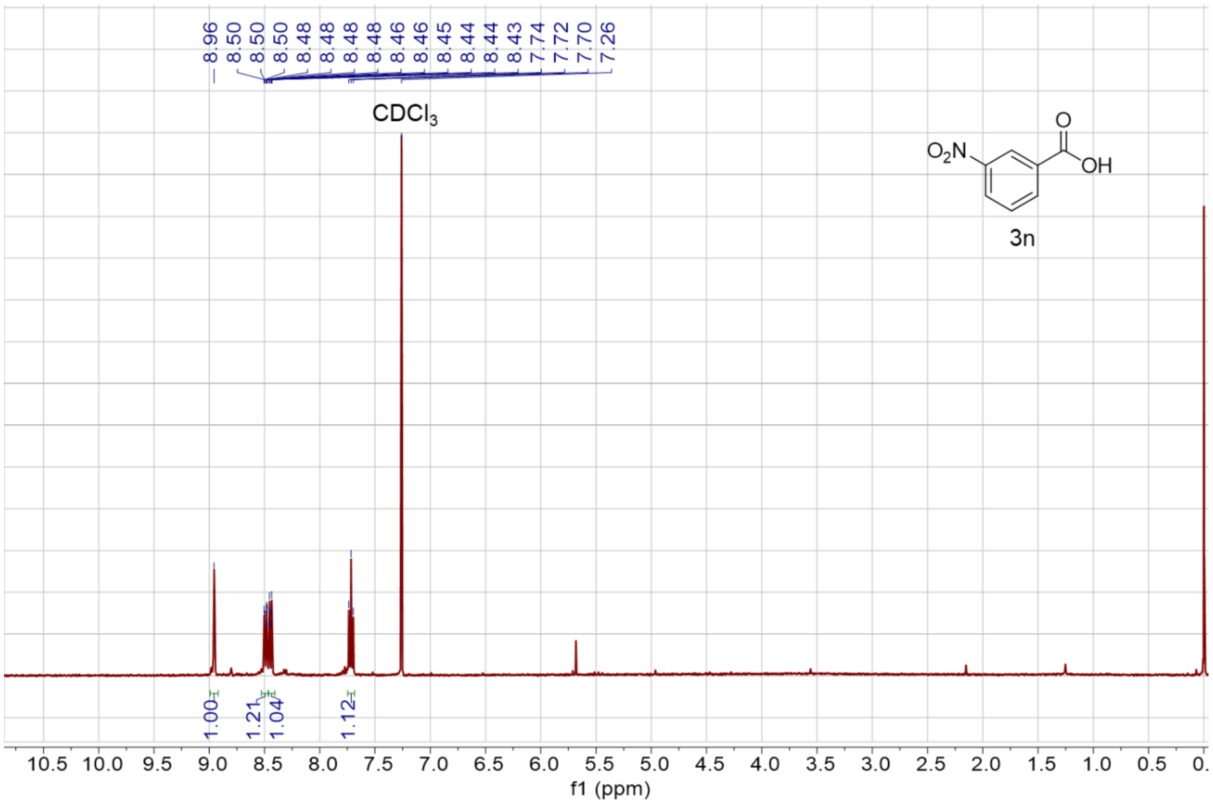

Figure S214: ^{1}H NMR spectrum of the isolated **3n** in $CDCl_3$ as the solvent.

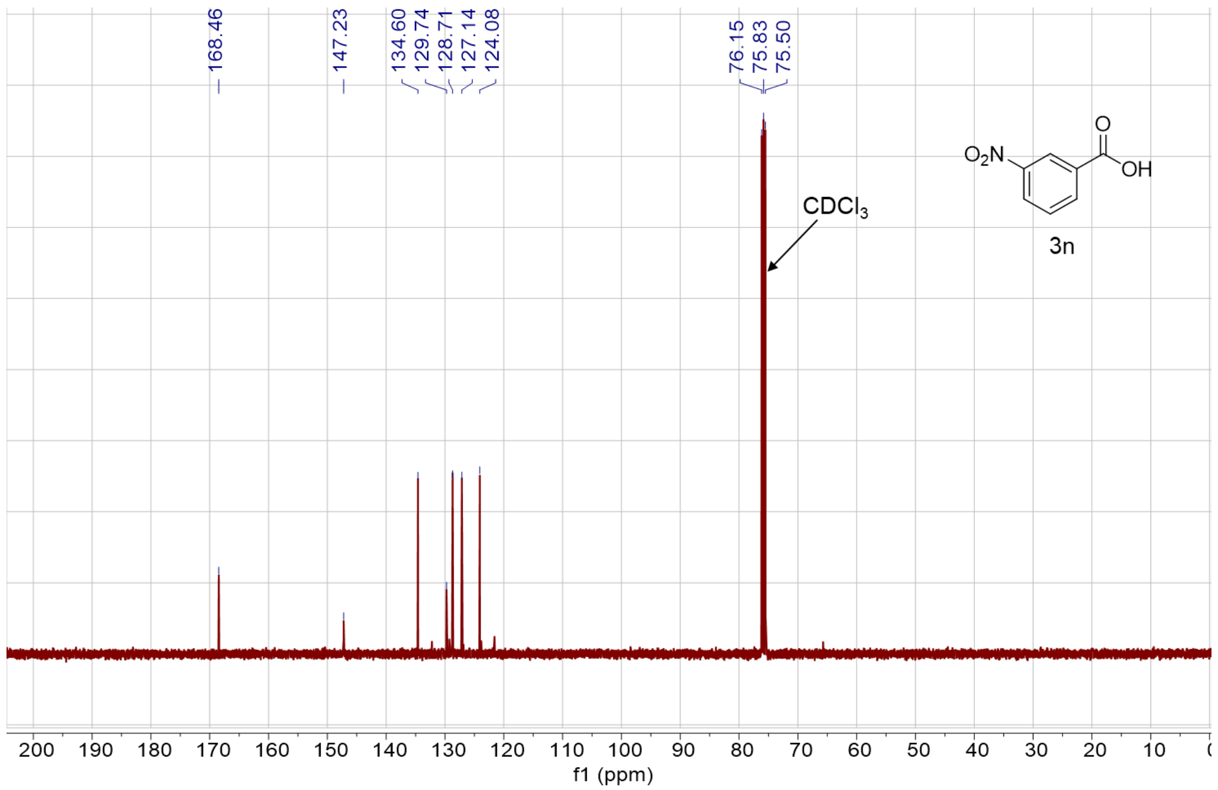

Figure S215: $^{13}C\{^1H\}$ NMR spectrum of the isolated **3n** in $CDCl_3$ as the solvent.

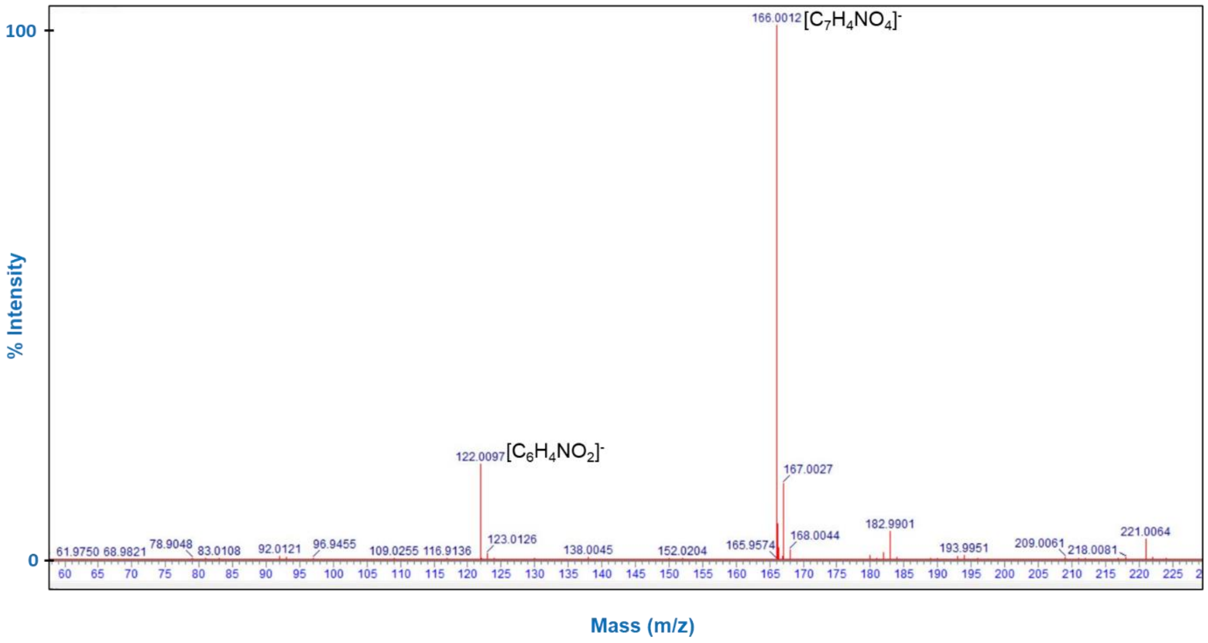

Figure S216: HRMS of the isolated **3n**.

Acetophenone (**2o**)

^{1}H NMR (400 MHz, CHLOROFORM-*D*) δ 2.51 (s, 3 H), 7.37 (t, *J* = 7.9 Hz, 2 H), 7.44 – 7.52 (m, 1 H), 7.88 (d, *J* = 5.6 Hz, 2 H).

^{13}C{^{1}H} NMR (100 MHz, CHLOROFORM-*D*) δ 25.3, 127.1, 127.3, 131.9, 135.9, 196.8.

HRMS (ESI-QTOF, m/z) calculated for $[C_8H_9O]^+$ *m/z* = 121.0501, found 121.0502.

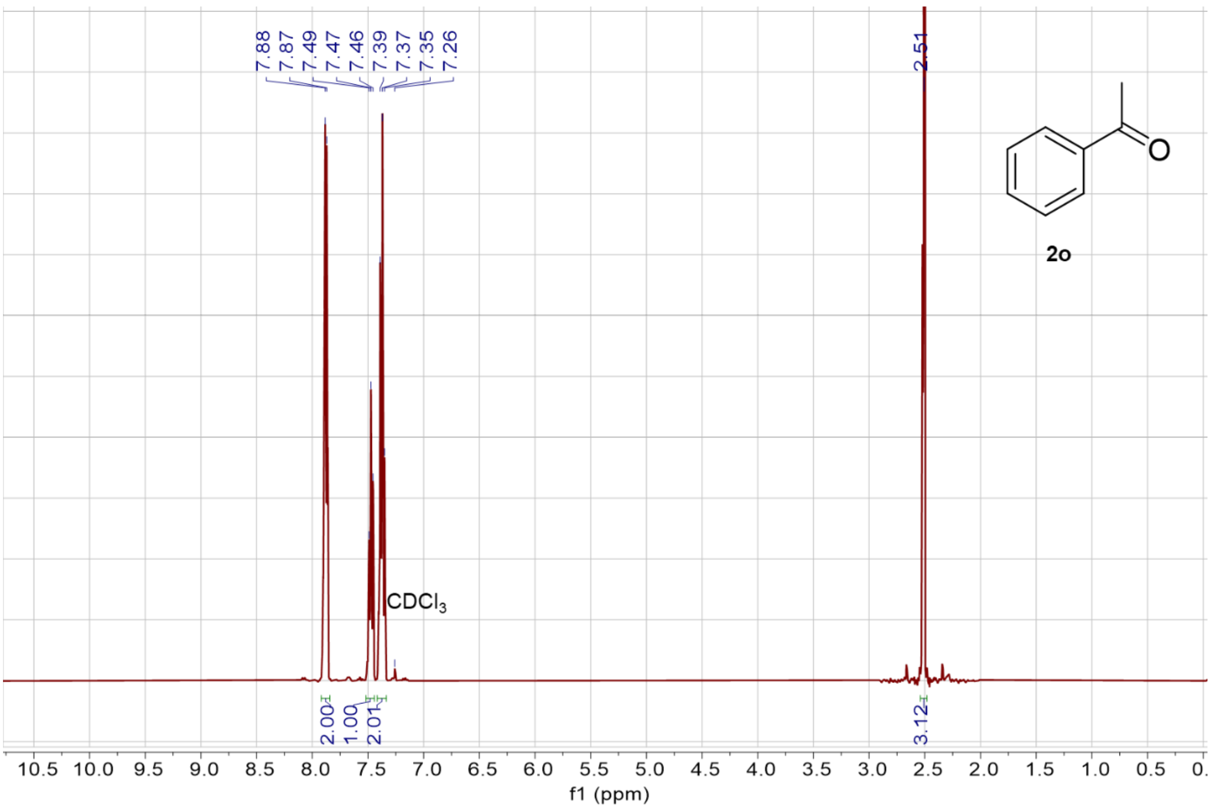

Figure S217: ^{1}H NMR spectrum of the isolated **2o** in $CDCl_3$ as the solvent.

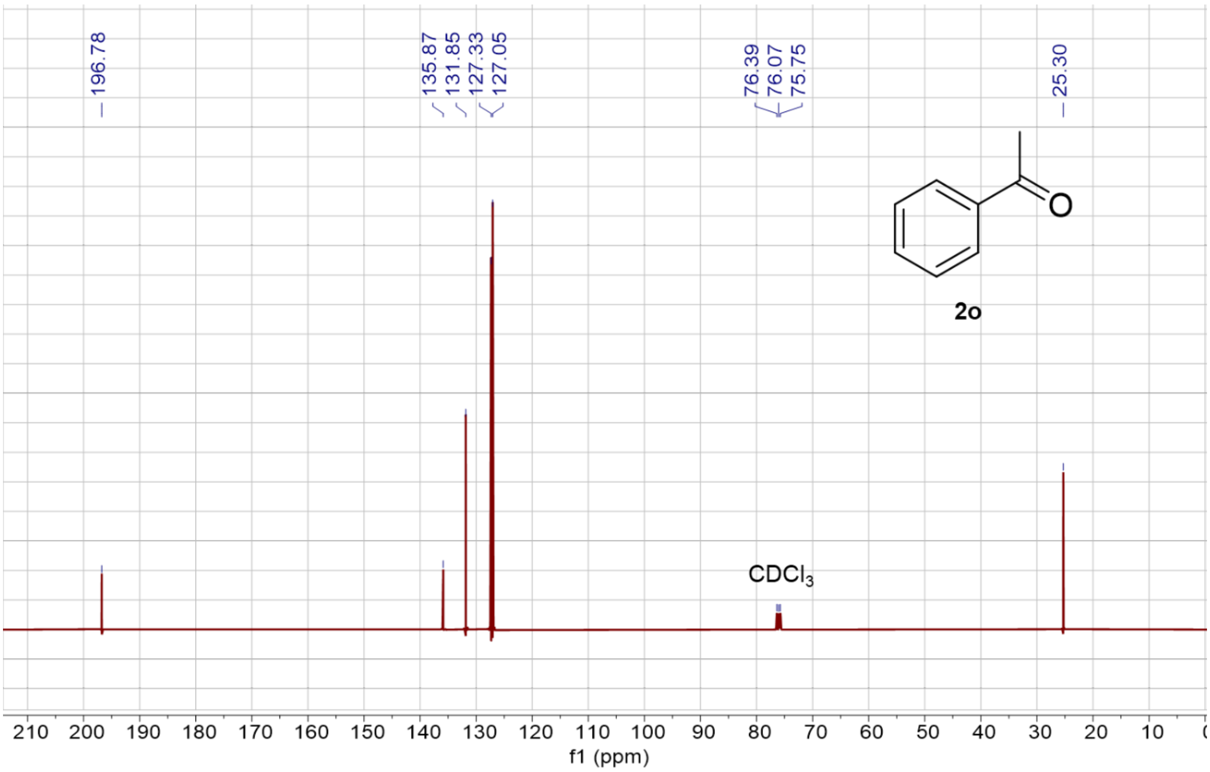

Figure S218: ^{13}C{^{1}H} NMR spectrum of the isolated **2o** in $CDCl_3$ as the solvent.

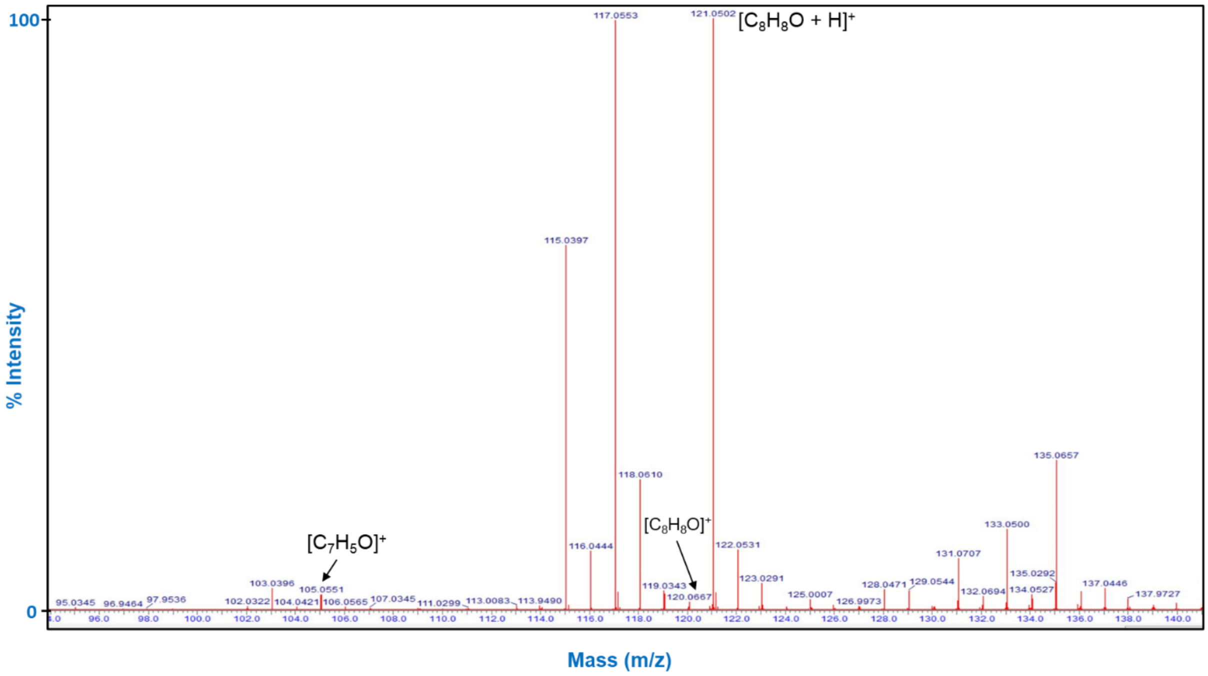

Figure S219: HRMS of the isolated **2o**.

2-naphthoic acid (**3v**)

^{1}H NMR (400 MHz, CHLOROFORM-*D*) δ 7.53– 7.66 (m, 2 H), 7.93 (dd, *J* = 8.5, 3.4 Hz, 2 H), 8.00 (d, *J* = 8.1 Hz, 1 H), 8.07– 8.18 (m, 1 H), 8.73 (s, 1 H).

^{13}C{^{1}H} NMR (100 MHz, CHLOROFORM-*D*) δ 124.2, 125.6, 125.3, 126.7, 127.2, 127.5, 128.4, 131.0, 131.3, 134.8, 170.3.

HRMS (ESI-QTOF, m/z) calculated for $[C_{11}H_7O_2]^-$ *m/z* = 171.0293, found 171.0300.

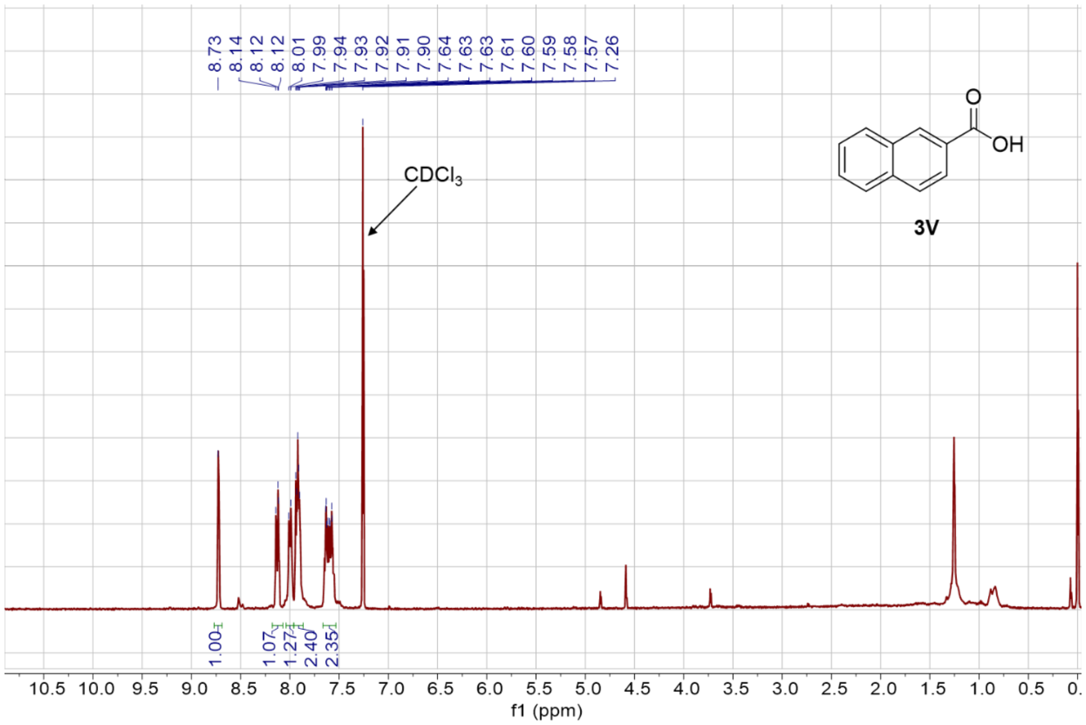

Figure S220: ^{1}H NMR spectrum of the isolated **3v** in $CDCl_3$ as the solvent.

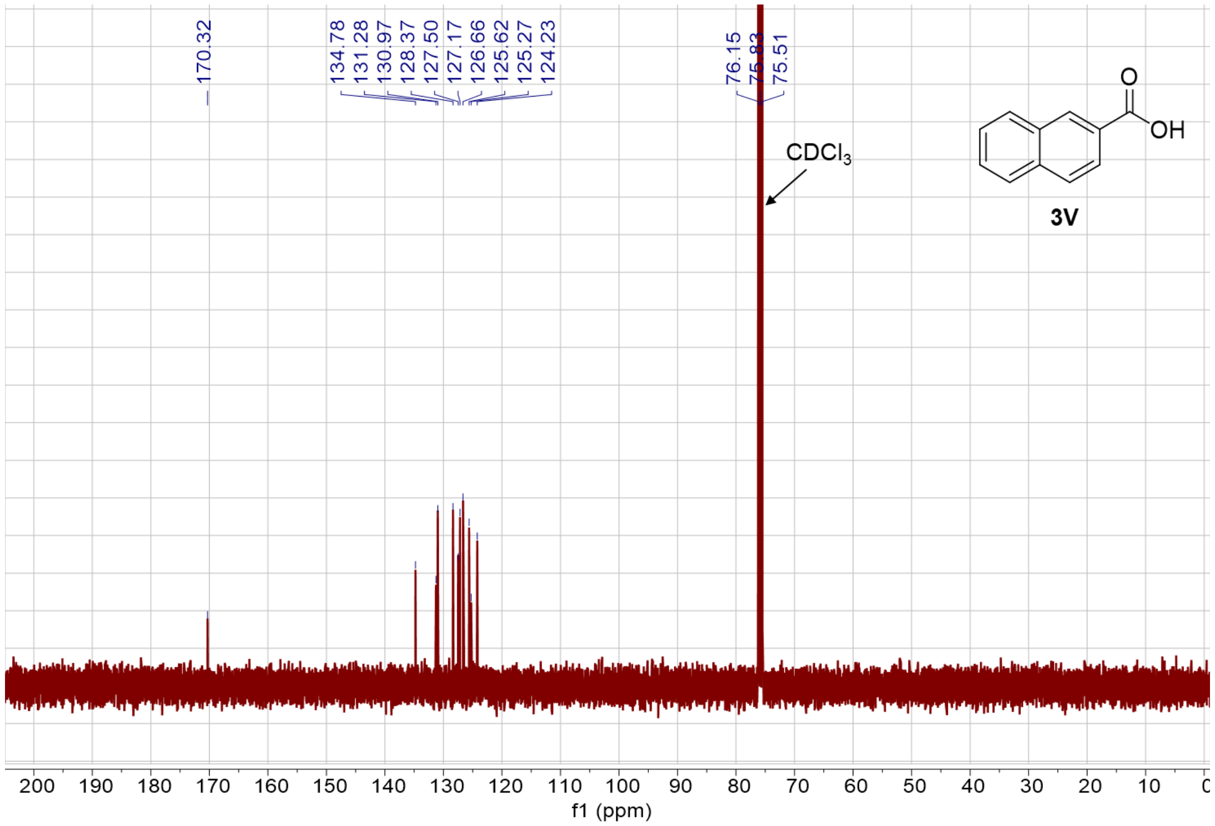

Figure S221: $^{13}C\{^{1}H\}$ NMR spectrum of the isolated **3v** in $CDCl_3$ as the solvent.

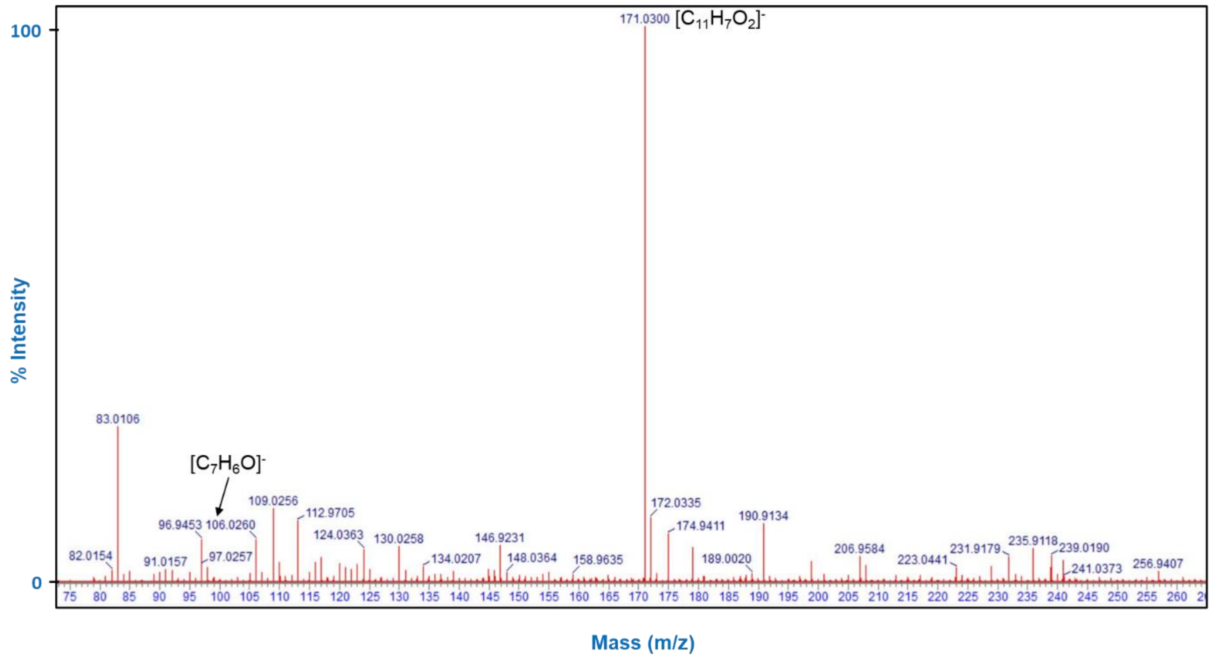

Figure S222: HRMS of the isolated **3v**.

Isonicotinic acid (**3w**)

^{1}H NMR (400 MHz, ACETONE-*D*$_6$) δ 7.87 (d, *J* = 6.0 Hz, 2 H), 8.78 (d, *J* = 6.0 Hz, 2 H).

$^{13}C\{^{1}H\}$ NMR (100 MHz, CHLOROFORM-*D*) δ 121.0, 123.1, 147.6, 170.8.

HRMS (ESI-QTOF, m/z) calculated for $[C_6H_4NO_2]^-$ *m/z* = 122.0089, found 122.0097.

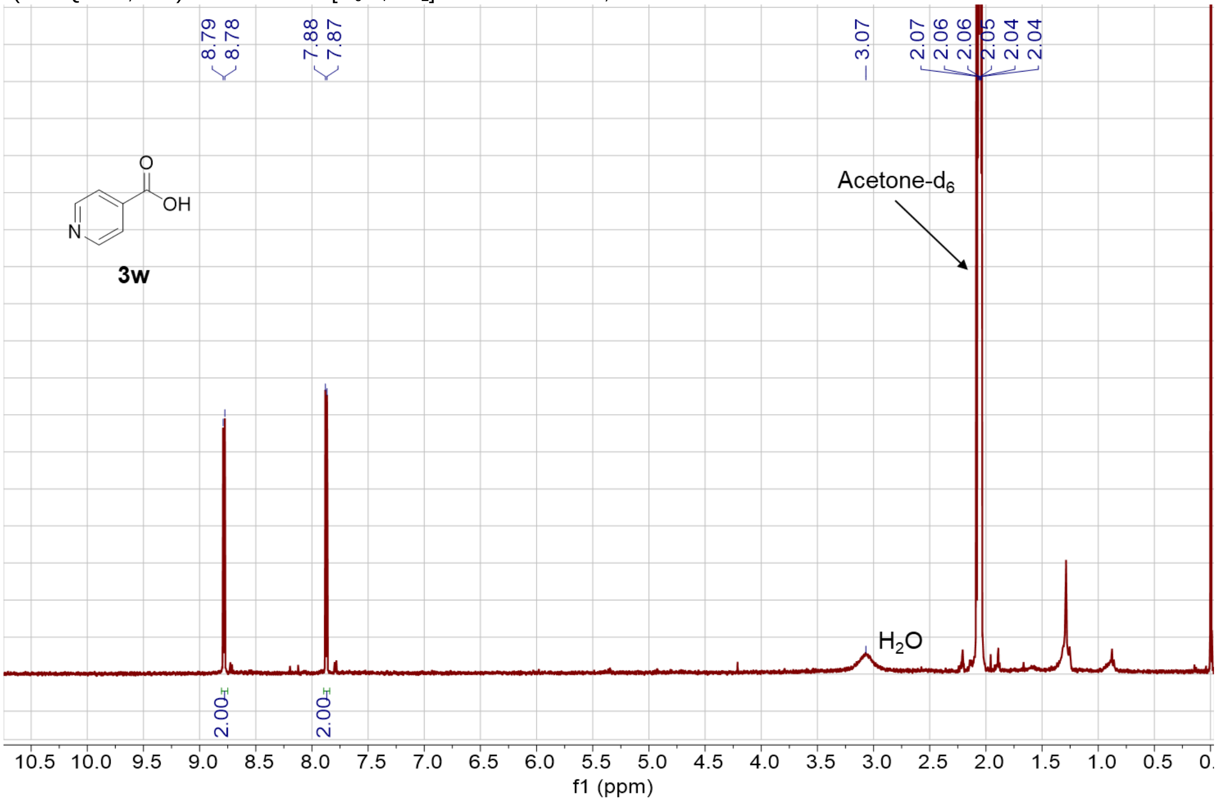

Figure S223: ^{1}H NMR spectrum of the isolated **3w** in acetone-d$_6$ as the solvent.

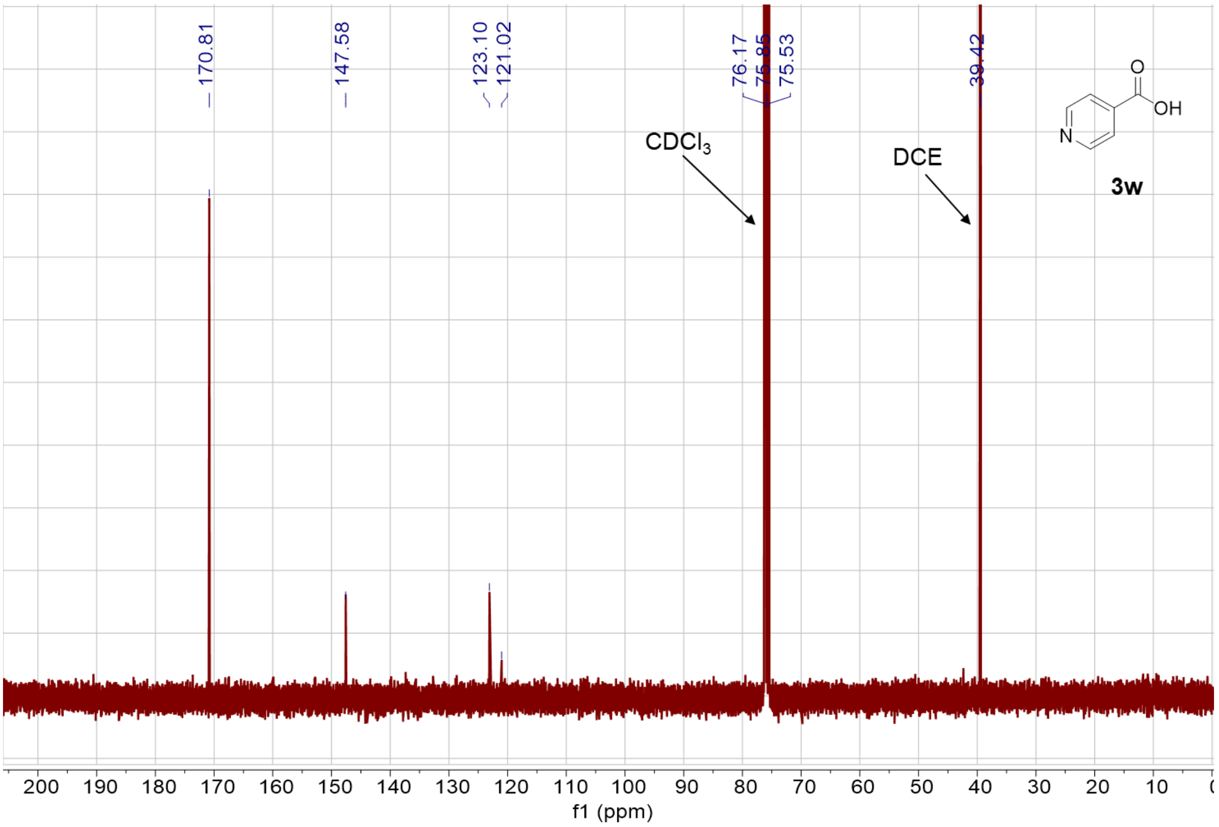

Figure S224: $^{13}C\{1H\}$ NMR spectrum of the isolated **3w** in $CDCl_3$ as the solvent.

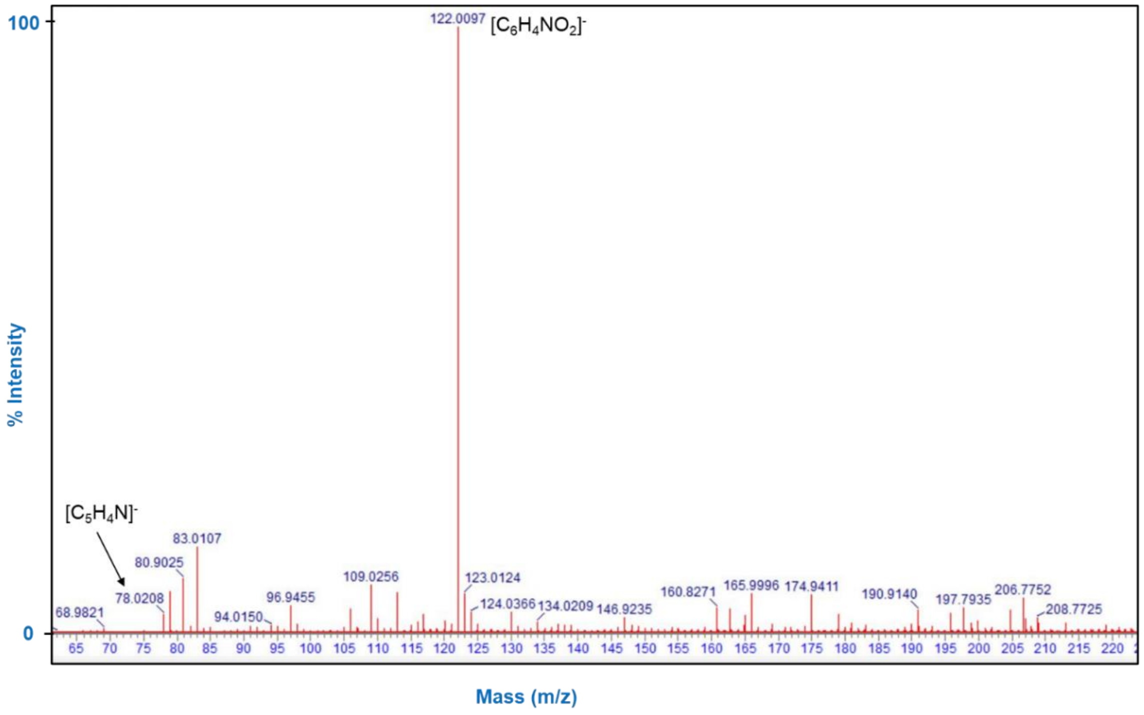

Figure S225: HRMS of the isolated **3w**.

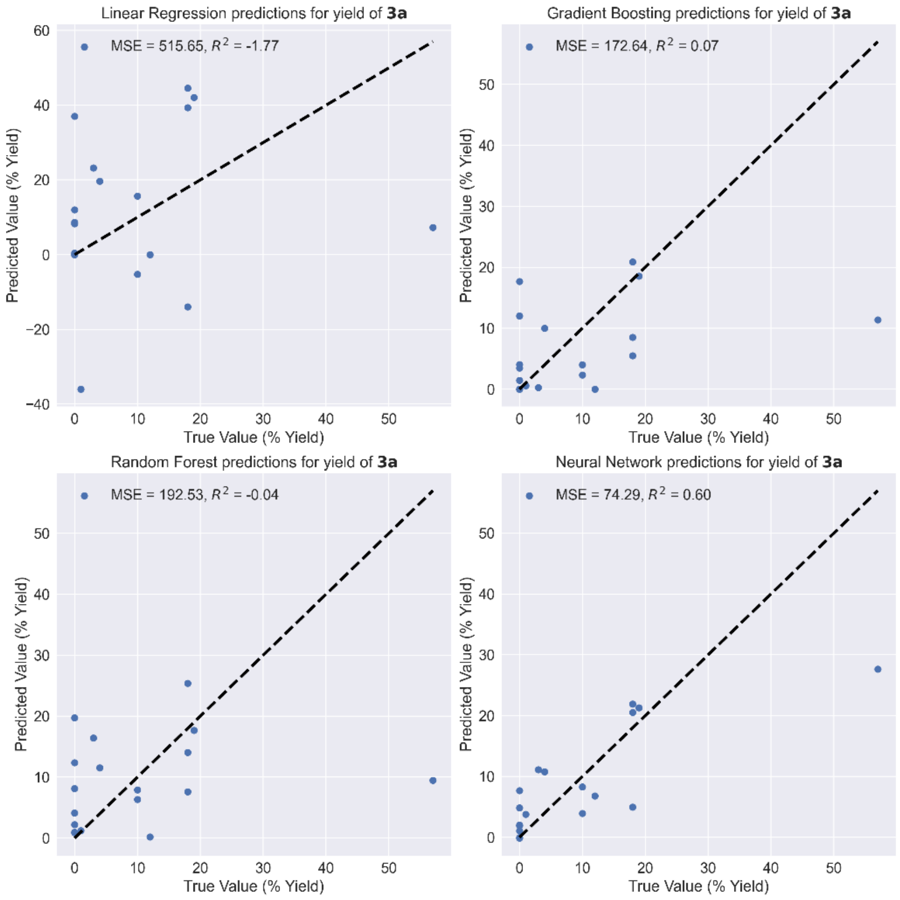

Figure S226: The performance comparison for the ML strategy based on the first dataset between linear regression, gradient boosting, random forest, and neural networks.

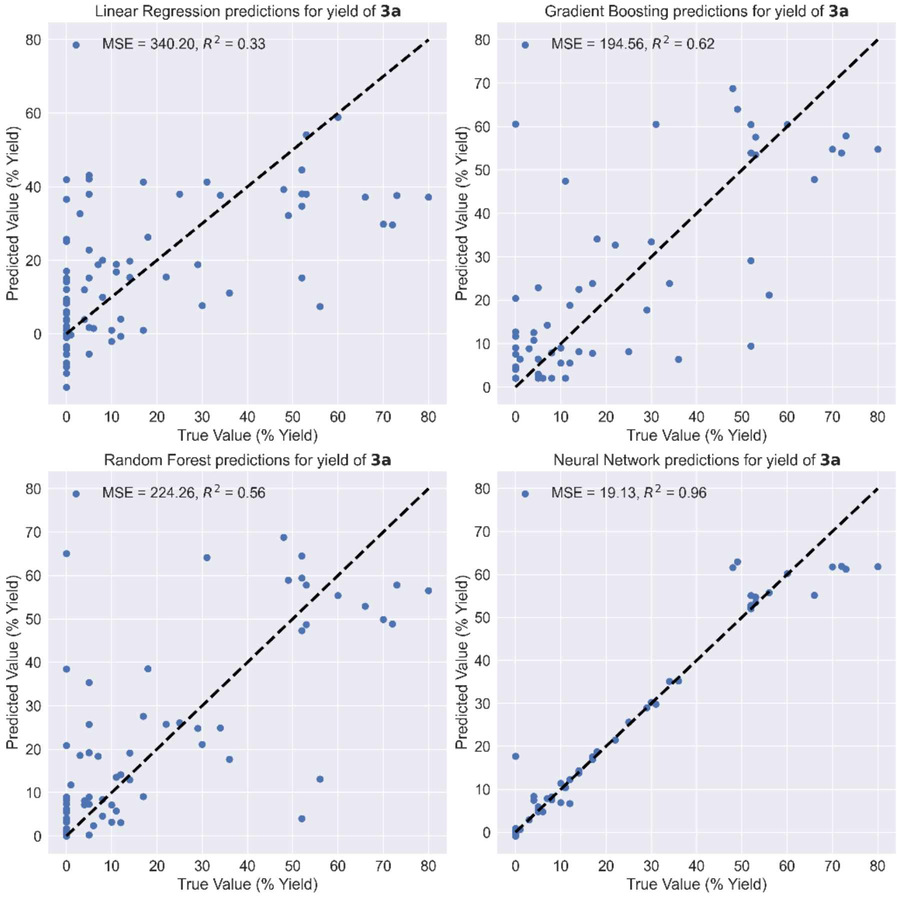

Figure S227: The performance comparison for the ML strategy based on the final dataset between linear regression, gradient boosting, random forest, and neural networks.

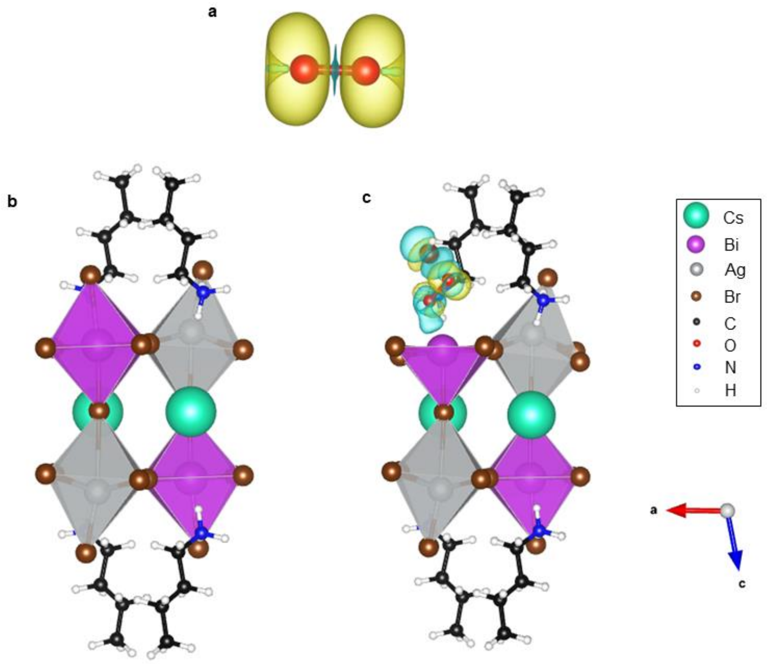

Figure S228: (a) Optimized geometry of gas phase O_2 superimposed with the spin-polarization map demonstrating the triplet spin. (b) Optimized geometry of $BA_2CsAgBiBr_7$ with the (002) facet exposed. (c) Optimized geometry of $BA_2CsAgBiBr_7$ with the (002) facet exposed and the adsorption of O_2 superimposed with the spin-polarization map demonstrating the quenched spin upon adsorption.

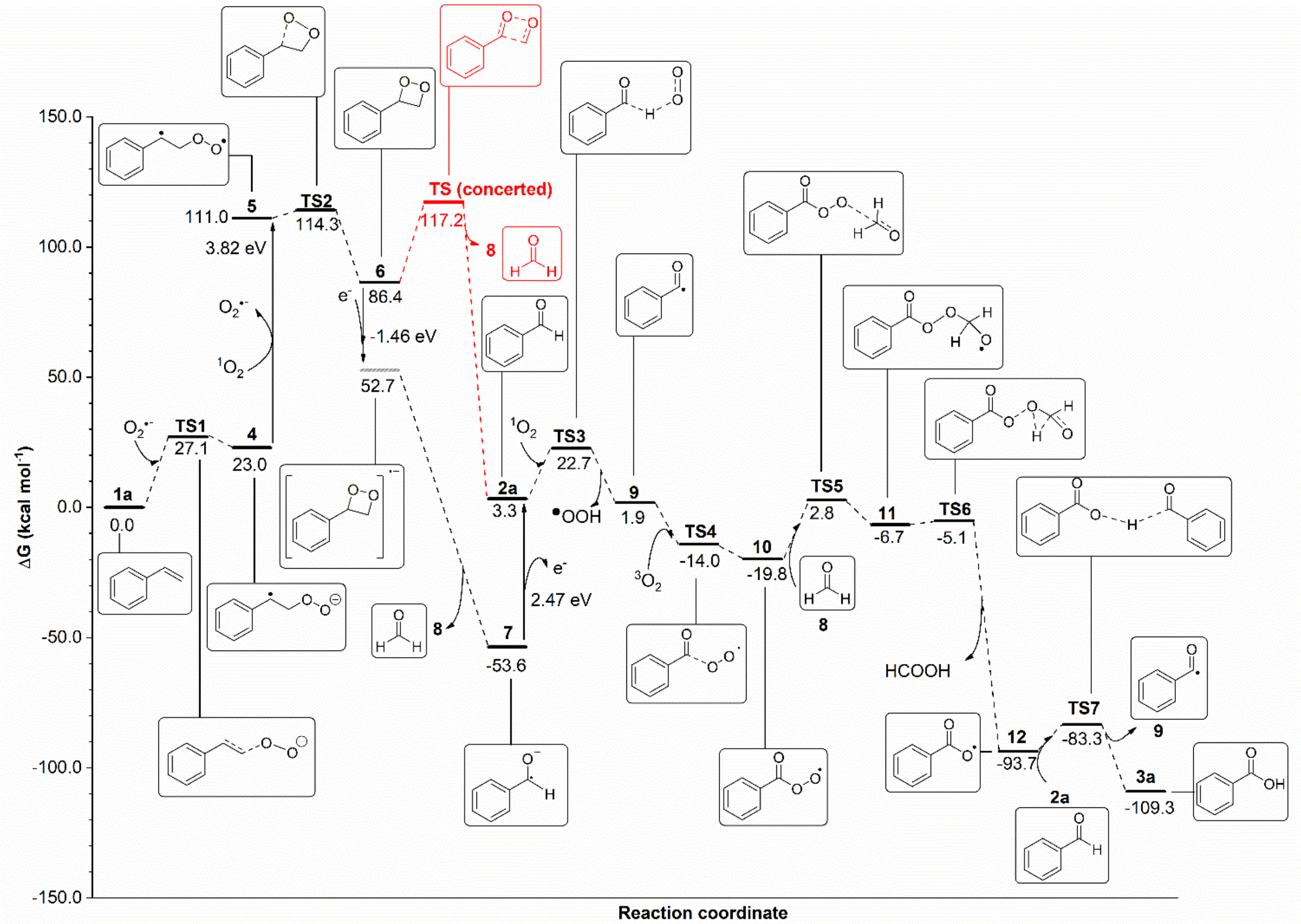

Figure S229: Energy profile (kcal mol-1) determined at the B3LYP/6-311G++(d,p) level for the oxidation of **1a** to **3a**, according to the proposed mechanism outlined in **Scheme 1** of the main text. The grey level represents the vertical electron affinity of **6** calculated with a single point energy calculation at the B3LYP/6-311G++(d,p) level using the optimized geometry for the neutral form of **6**. The pathway in red represents the kinetically unfavorable concerted retro-cycloaddition ring-opening of **6** to form **2a** and **8**.

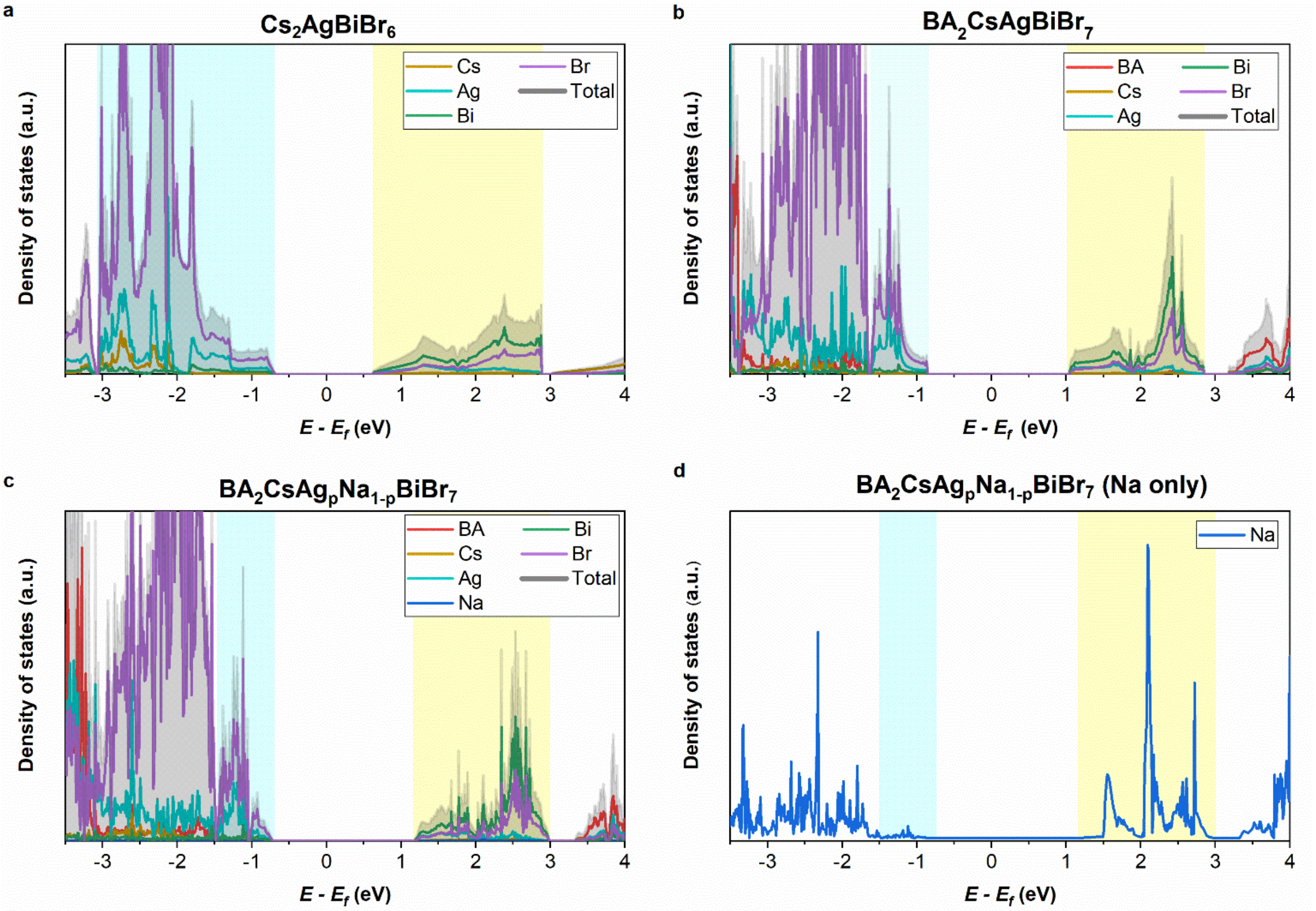

Figure S230: Densities of state of (a) $Cs_2AgBiBr_6$, (b) $BA_2CsAgBiBr_7$, (c) $BA_2CsAg_pNa_{1-p}Br_7$, and (d) only the Na contributions in $BA_2CsAg_pNa_{1-p}Br_7$, where p = 0.875. The valence and conduction bands are highlighted in blue and yellow respectively.

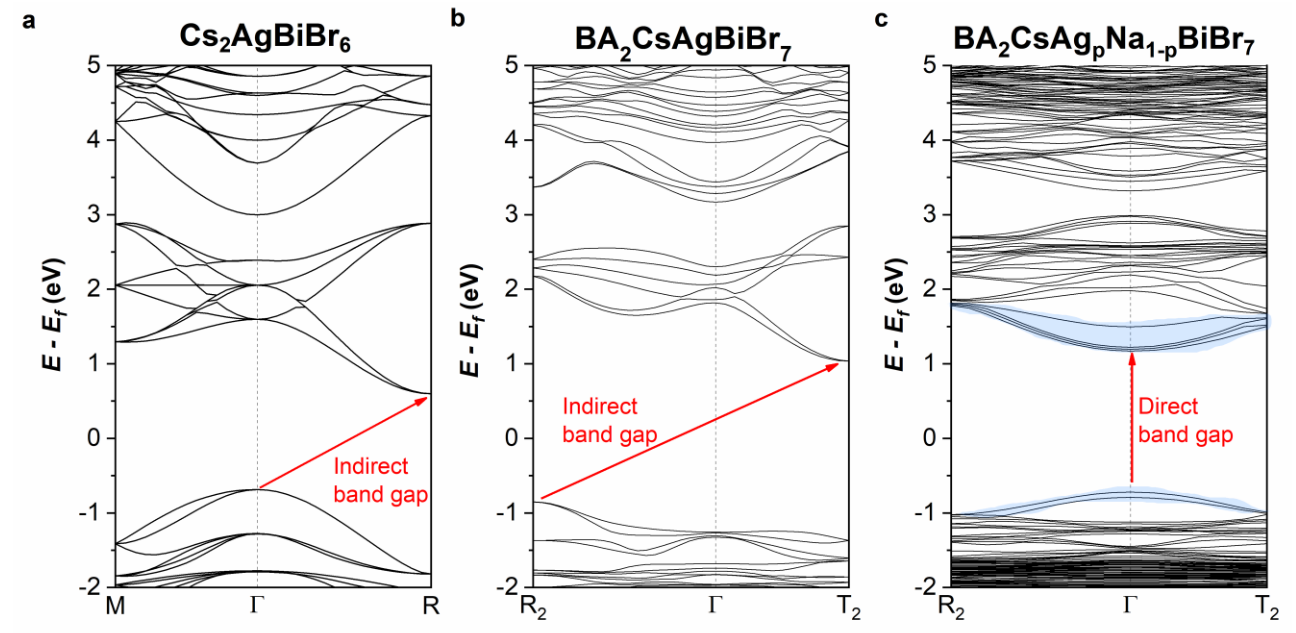

Figure S231: Band structure diagrams of (a) $Cs_2AgBiBr_6$, (b) $BA_2CsAgBiBr_7$, (c) $BA_2CsAg_pNa_{1-p}Br_7$, where p = 0.875. The additional electronic states at the valence and conduction band edges owing to the introduction of Na are highlighted in pale blue.

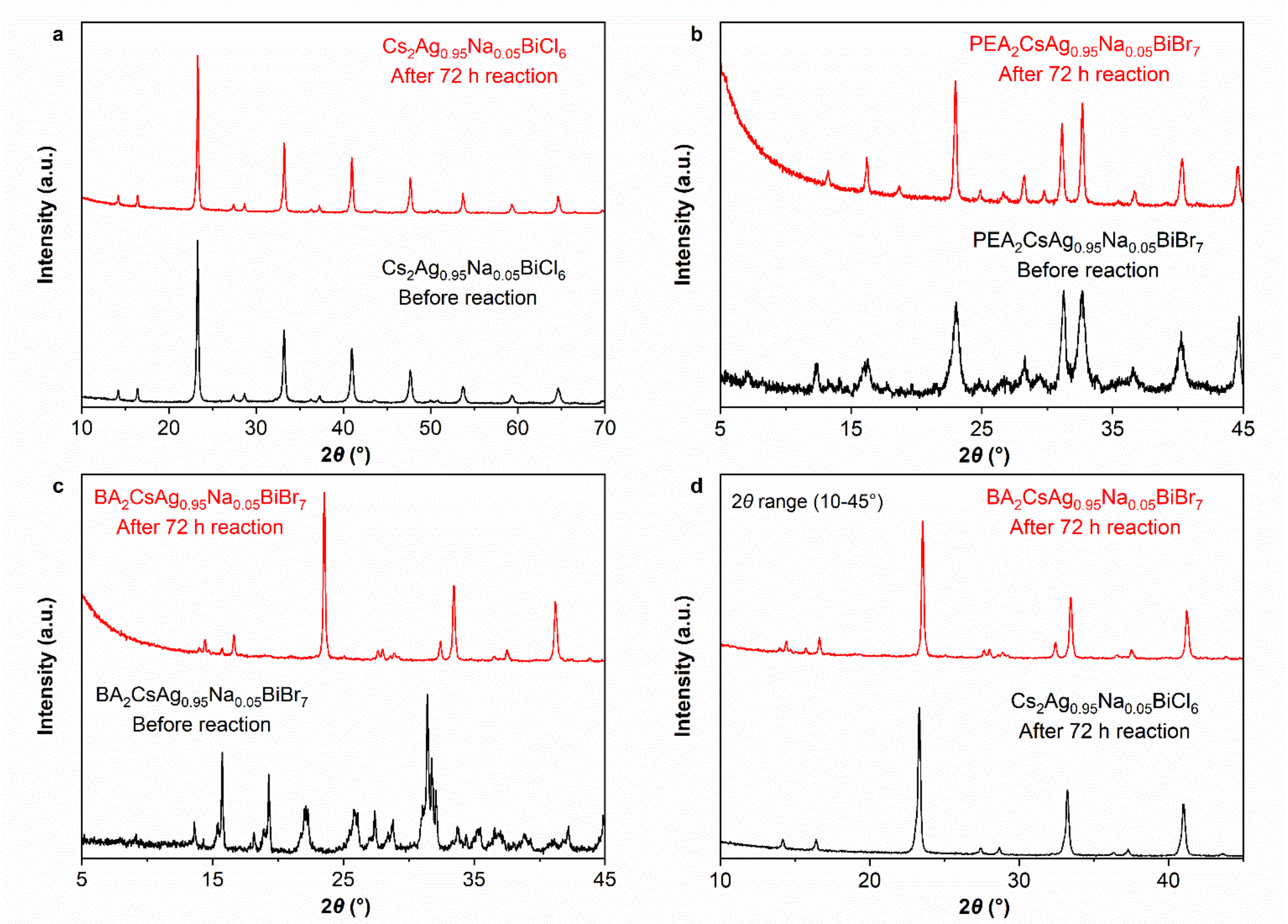

Figure S232: Comparisons of the XRD patterns of (a) $Cs_2Ag_{0.95}Na_{0.05}BiBr_6$, (b) $PEA_2CsAg_{0.95}Na_{0.05}BiBr_7$, and (c) $BA_2CsAg_{0.95}Na_{0.05}BiBr_7$ before and after 72 h of being used as the photocatalyst for the styrene oxidation reaction under the optimized conditions. (d) A comparison showing the similarity of the $BA_2CsAg_{0.95}Na_{0.05}BiBr_7$ that had been recovered from the 72 h photocatalytic oxidation of styrene with a 3D double perovskite phase.

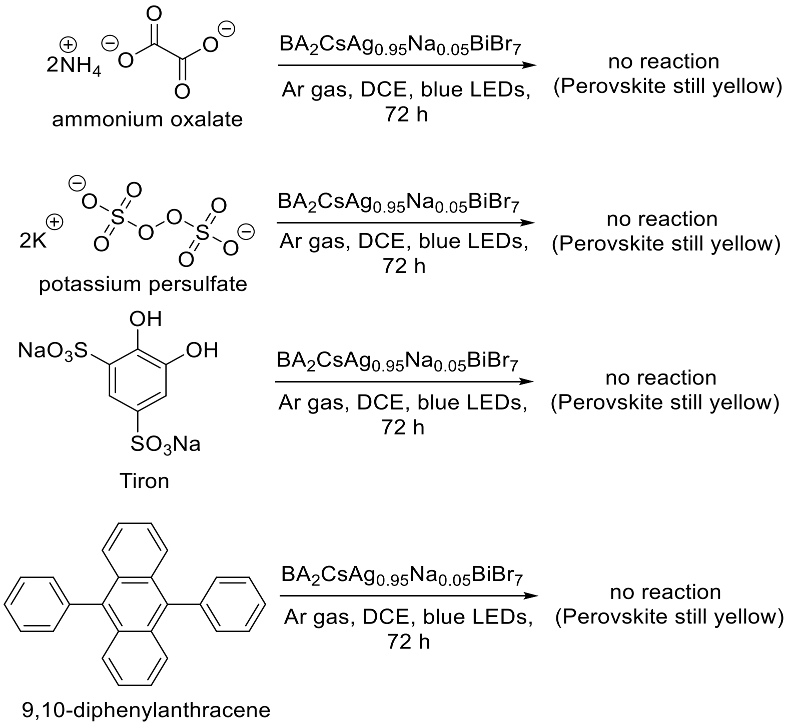

Scheme S1. Exploration of the stability of $BA_2CsAg_{0.95}Na_{0.05}BiBr_7$ after exposure to electron scavengers. All the radical scavenging experiments were performed with $BA_2CsAg_{0.95}Na_{0.05}BiBr_7$ in DCE under blue LEDs and Ar gas for 72 h.

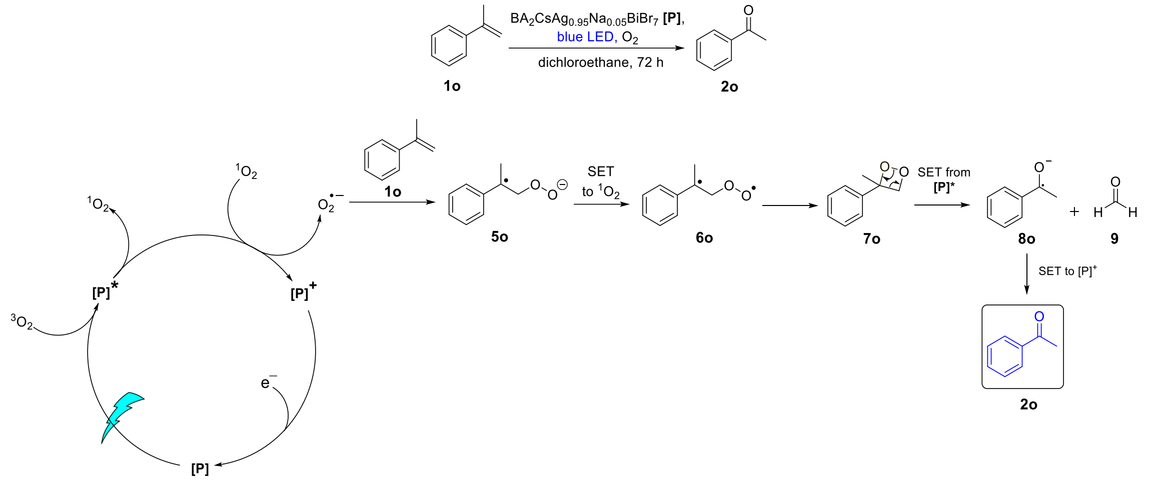

Scheme S2. Proposed mechanism for the $BA_2CsAg_{0.95}Na_{0.05}BiBr_7$ photocatalyzed oxidative cleavage of **1o** to generate the ketone **2o**.

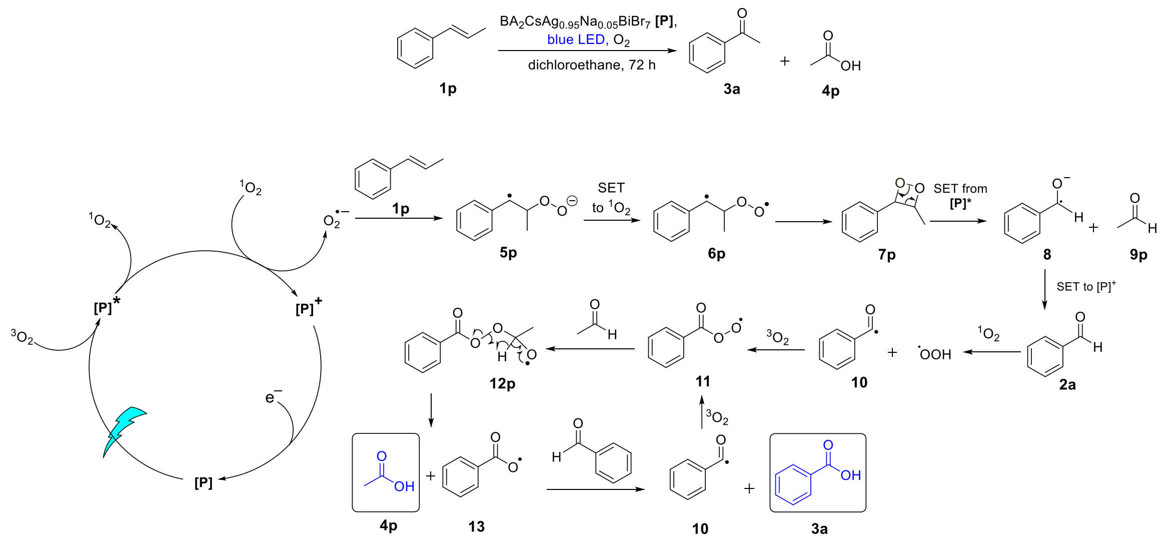

Scheme S3. Proposed mechanism for the $BA_2CsAg_{0.95}Na_{0.05}BiBr_7$ photocatalyzed oxidative cleavage of **1p** and **1q** to produce **4p** as a by-product.

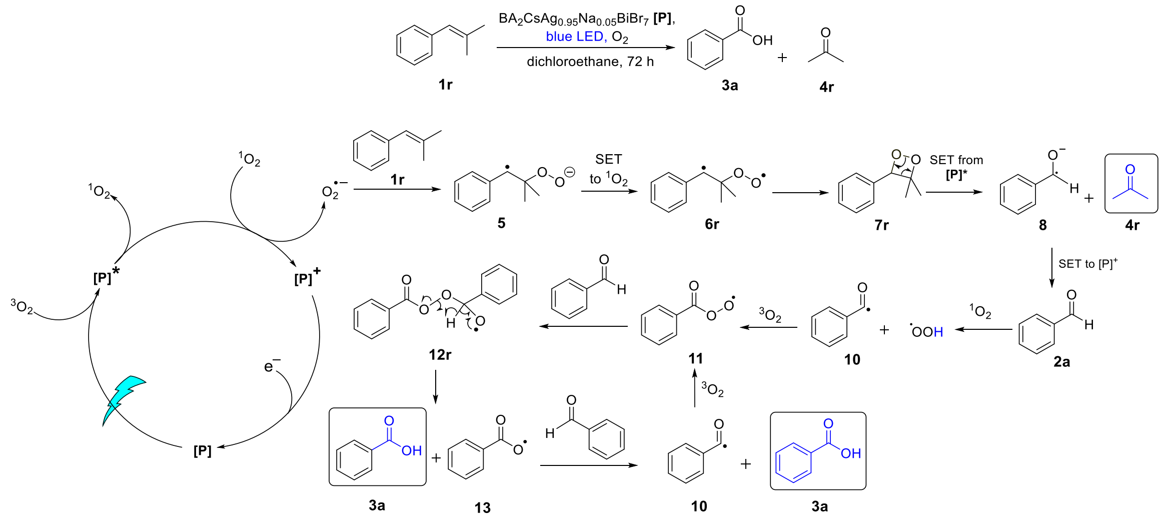

Scheme S4. Proposed mechanism for the $BA_2CsAg_{0.95}Na_{0.05}BiBr_7$ photocatalyzed oxidative cleavage of **1r** to produce **4r** as a by-product.

SUPPORTING INFORMATION

Supporting Tables

Table S1. Original candidates proposed.

No.	Possible candidates	TF	μ	XRD results
1.	$Cs_2AgBiCl_6$	0.90	0.57	$Cs_2AgBiCl_6$
2.	$Cs_2AgBiBr_6$	0.89	0.53	$Cs_2AgBiBr_6$
3.	Cs_2AgBiI_6	0.88	0.47	$Cs_3Bi_2I_9$
4.	$Cs_2CuBiCl_6$	0.96	0.57	$Cs_3Bi_2Cl_9$
5.	$Cs_2CuBiBr_6$	0.95	0.53	$Cs_3Bi_2Br_9$
6.	Cs_2CuBiI_6	0.93	0.47	$Cs_3Bi_2I_9$
7.	Cs_2KBiCl_6	0.87	0.57	$Cs_3Bi_2Cl_9$
8.	Cs_2KBiBr_6	0.86	0.53	$Cs_3Bi_2Br_9$
9.	Cs_2KBiI_6	0.85	0.47	$Cs_3Bi_2I_9$
10.	$Cs_2NaBiCl_6$	0.92	0.57	$Cs_2NaBiCl_6$
11.	$Cs_2NaBiBr_6$	0.91	0.53	$Cs_3Bi_2Br_9$
12.	Cs_2NaBiI_6	0.89	0.47	$Cs_3Bi_2I_9$
13.	$K_2AgBiCl_6$	0.84	0.57	KCl + AgCl + $BiCl_3$
14.	$K_2AgBiBr_6$	0.83	0.53	KBr + AgBr + $BiBr_3$
15.	K_2AgBiI_6	0.83	0.47	KI + AgI + BiI_3
16.	$K_2CuBiCl_6$	0.90	0.57	KCl + CuCl + $BiCl_3$
17.	$K_2CuBiBr_6$	0.89	0.53	KBr + CuBr + $BiBr_3$
18.	K_2CuBiI_6	0.88	0.47	KI + CuI + BiI_3
19.	$Rb_2AgBiCl_6$	0.86	0.57	$Rb_2AgBiCl_6$
20.	$Rb_2AgBiBr_6$	0.85	0.53	$Rb_3Bi_2Br_9$ + Rb_3BiBr_6 + AgBr
21.	Rb_2AgBiI_6	0.84	0.47	Rb_2AgI_3 + $Rb_3Bi_2I_9$ + AgI
22.	$Rb_2CuBiCl_6$	0.92	0.57	$Rb_7Bi_3Cl_{16}$ + $RbCuCl_3$
23.	$Rb_2CuBiBr_6$	0.91	0.53	$RbCu_2Br_3$ + $Rb_3Bi_2Br_9$
24.	Rb_2CuBiI_6	0.89	0.47	$Rb_3Bi_2I_9$ + CuI + RbI
25.	Rb_2KBiCl_6	0.83	0.57	RbCl + KCl + $BiCl_3$
26.	Rb_2KBiBr_6	0.82	0.53	RbBr + KBr + $BiBr_3$
27.	Rb_2KBiI_6	0.81	0.47	RbI + KI + BiI_3
28.	$Rb_2NaBiCl_6$	0.88	0.57	$Rb_7Bi_3Cl_{16}$
29.	$Rb_2NaBiBr_6$	0.87	0.53	Rb_3BiBr_6
30.	Rb_2NaBiI_6	0.86	0.47	$Rb_7Bi_3I_{16}$
31.	$Cs_2AgSbCl_6$	0.94	0.42	$Cs_2AgSbCl_6$
32.	$Cs_2AgSbBr_6$	0.93	0.39	$Cs_3Sb_2Br_9$
33.	Cs_2AgSbI_6	0.91	0.35	$Cs_3Sb_2I_9$
34.	$Cs_2CuSbCl_6$	1.01	0.42	$Cs_3Sb_2Cl_9$
35.	$Cs_2CuSbBr_6$	1.00	0.39	$CsCu_2Br_3$ + $Cs_3Sb_2Br_9$
36.	Cs_2CuSbI_6	0.97	0.35	$Cs_3Sb_2I_9$
37.	Cs_2KSbCl_6	0.91	0.42	$Cs_3Sb_2Cl_9$
38.	Cs_2KSbBr_6	0.90	0.39	$Cs_3Sb_2Br_9$
39.	Cs_2KSbI_6	0.88	0.35	$Cs_3Sb_2I_9$
40.	$Cs_2NaSbCl_6$	0.97	0.42	$Cs_3Sb_2Cl_9$
41.	$Cs_2NaSbBr_6$	0.95	0.39	$Cs_3Sb_2Br_9$
42.	Cs_2NaSbI_6	0.93	0.35	$Cs_3Sb_2I_9$
43.	$K_2AgSbCl_6$	0.88	0.42	K_2SbCl_5 + AgCl
44.	$K_2AgSbBr_6$	0.87	0.39	AgBr + KBr + $SbBr_3$
45.	K_2AgSbI_6	0.86	0.35	AgI + KI + SbI_3

46.	$K_2CuSbCl_6$	0.95	0.42	$CuCl_2 + K_2SbCl_5 + Cu$
47.	$K_2CuSbBr_6$	0.93	0.39	$K_2CuBr_3 + SbBr_3$
48.	K_2CuSbI_6	0.92	0.35	$CuI + KI + SbI_3$
49.	$Rb_2AgSbCl_6$	0.90	0.42	Rb_2AgCl_3
50.	$Rb_2AgSbBr_6$	0.89	0.39	$Rb_3Sb_2Br_9$
51.	Rb_2AgSbI_6	0.88	0.35	$Rb_3Sb_2I_9$
52.	$Rb_2CuSbCl_6$	0.97	0.42	$Rb_3Sb_2Cl_9$
53.	$Rb_2CuSbBr_6$	0.95	0.39	$Rb_3Sb_2Br_9$
54.	Rb_2CuSbI_6	0.93	0.35	$Rb_3Sb_2I_9$
55.	Rb_2KSbCl_6	0.87	0.42	Rb_3SbCl_9
56.	Rb_2KSbBr_6	0.86	0.39	$Rb_3Sb_2Br_9$
57.	Rb_2KSbI_6	0.85	0.35	$Rb_3Sb_2I_9$
58.	$Rb_2NaSbCl_6$	0.92	0.42	Rb_3SbCl_6
59.	$Rb_2NaSbBr_6$	0.91	0.39	$Rb_3Sb_2Br_9$
60.	Rb_2NaSbI_6	0.90	0.35	$Rb_3Sb_2I_9$
61.	$Cs_2AgInCl_6$	0.94	0.44	$Cs_2AgInCl_6$
62.	$Cs_2AgInBr_6$	0.93	0.41	$Cs_2AgBr_3 + Cs_3In_2Br_9$
63.	Cs_2AgInI_6	0.91	0.36	$Cs_2AgI_3 + CsInI_4 + AgI$
64.	$Cs_2CuInCl_6$	1.01	0.44	$CsCu_2Cl_3 + Cs_3In_2Cl_9$
65.	$Cs_2CuInBr_6$	0.99	0.41	$Cs_3InBr_6 + Cs_3Cu_2Br_5 + InBr_3$
66.	Cs_2CuInI_6	0.97	0.36	$CsInI_4$
67.	Cs_2KInCl_6	0.90	0.44	Cs_2KInCl_6
68.	Cs_2KInBr_6	0.89	0.41	$Cs_3In_2Br_9$
69.	Cs_2KInI_6	0.88	0.36	$CsInI_4 + CsI + KI$
70.	$Cs_2NaInCl_6$	0.96	0.44	$Cs_2NaInCl_6$
71.	$Cs_2NaInBr_6$	0.95	0.41	$Cs_3In_2Br_9$
72.	Cs_2NaInI_6	0.93	0.36	$CsInI_4 + NaI + CsI$
73.	$K_2AgInCl_6$	0.88	0.44	$K_3InCl_6 + InCl_3 + AgCl$
74.	$K_2AgInBr_6$	0.87	0.41	$KInBr_4 + KBr + AgBr$
75.	K_2AgInI_6	0.86	0.36	$InI_3 + KI + AgI$
76.	$K_2CuInCl_6$	0.94	0.44	$K_3InCl_6 + CuCl_2 + InCl_3 + Cu$
77.	$K_2CuInBr_6$	0.93	0.41	$K_2CuBr_3 + KInBr_4 + CuBr$
78.	K_2CuInI_6	0.91	0.36	$KInI_4$
79.	$Rb_2AgInCl_6$	0.90	0.44	$RbCl + AgCl + InCl_3$
80.	$Rb_2AgInBr_6$	0.89	0.41	$InBr_3 + Rb_2AgBr_3$
81.	Rb_2AgInI_6	0.87	0.36	$Rb_2AgI_3 + RbInI_4 + AgI$
82.	$Rb_2CuInCl_6$	0.96	0.44	$Rb_4Cu_5Cl_9$
83.	$Rb_2CuInBr_6$	0.95	0.41	$RbCu_2Br_3 + InBr_3 + RbBr$
84.	Rb_2CuInI_6	0.93	0.36	$RbCu_2I_3$

Table S2. Optimization of the solvents and light sources for the $Cs_2AgBiBr_6$ photocatalyzed oxidation of 1a.

Entry.	Catalyst (5 mol %)	Solvent	Light Source	2a (%)	3a (%)
1.	$Cs_2AgBiBr_6$	DCE	White LED	4	20
2.	$Cs_2AgBiBr_6$	CH_2Cl_2	White LED	10	0
3.	$Cs_2AgBiBr_6$	CH_3CN	White LED	4	0
4.	$Cs_2AgBiBr_6$	Neat	White LED	14	18
5.	$Cs_2AgBiBr_6$	Neat	Blue LED	28	45
6.	$Cs_2AgBiBr_6$	Heptane	Blue LED	6	8
7.	$Cs_2AgBiBr_6$	EtOAc	Blue LED	12	0
8.	$Cs_2AgBiBr_6$	DCE	Blue LED	20	6
9.	$Cs_2AgBiBr_6$ (10 mol %)	DCE	Blue LED	14	2

Condition: **1a** (60 µl, 0.524 mmol), $Cs_2AgBiBr_6$ (5 mol%), DCE (3 mL), O_2 (1 atm), RT, visible light.

Table S3. Evaluation of the different perovskite photocatalyzed oxidation of 1a.[a]

No.	Catalyst (5 mol %)	2a (%)	3a (%)
1.	Cs_3BiCl_6	18	0
2.	$Cs_3Bi_2Cl_9$	14	0
3.	$Cs_3Bi_2Br_9$	10	0
4.	$Cs_3Bi_2I_9$	28	18
5.	$Rb_3Bi_2Br_9$	18	1
6.	$MA_3Bi_2Cl_9$	18	0
7.	$MA_3Bi_2I_9$	16	10
8.	$FA_3Bi_2Cl_9$	32	12
9.	$FA_3Bi_2Br_9$	20	3
10.	$FA_3Bi_2I_9$	4	4
11.	$Cs_3Sb_2Br_9$	24	18
12.	$Cs_3Sb_2I_9$	18	19
13.	$Rb_3Sb_2Br_9$	12	0
14.	$Rb_3Sb_2I_9$	14	18
15.	$MA_3Sb_2Br_9$	0	57
16.	$MASnCl_3$	4	0
17.	$FASnCl_3$	6	0
18.	$FASnBr_3$	24	0
19.	$FASnI_3$	8	10
20.	$K_2CsBiCl_6$	14	0
21.	$Rb_2AgBiCl_6$	32	0
22.	$Cs_2NaBiCl_6$	32	6
23.	$Cs_2Ag_{0.05}Na_{0.95}BiCl_6$	14	0
24.	$Cs_2Ag_{0.1}Na_{0.9}BiCl_6$	20	0
25.	$Cs_2Ag_{0.2}Na_{0.8}BiCl_6$	16	0
26.	$Cs_2Ag_{0.4}Na_{0.6}BiCl_6$	16	0
27.	$Cs_2Ag_{0.5}Na_{0.5}BiCl_6$	22	0
28.	$Cs_2Ag_{0.6}Na_{0.4}BiCl_6$	26	0
29.	$Cs_2Ag_{0.8}Na_{0.2}BiCl_6$	30	11
30.	$Cs_2Ag_{0.9}Na_{0.1}BiCl_6$	26	11

31.	$Cs_2Ag_{0.95}Na_{0.05}BiCl_6$	0	45
32.	$Cs_2AgBiCl_6$	18	20
33.	$Cs_2AgBiBrCl_5$[b]	0	35
34.	$Cs_2AgBiBr_2Cl_4$	2	27
35.	$Cs_2AgBiBr_3Cl_3$	20	3
36.	$Cs_2AgBiBr_4Cl_2$	22	4
37.	$Cs_2AgBiBr_5Cl$	10	3
38.	$Cs_2AgBiBr_6$	20	6
39.	$Cs_2NaInCl_6$	10	2
40.	$Cs_2Ag_{0.2}Na_{0.8}InCl_6$	10	0
41.	$Cs_2Ag_{0.4}Na_{0.6}InCl_6$	06	0
42.	$Cs_2Ag_{0.6}Na_{0.4}InCl_6$	10	0
43.	$Cs_2Ag_{0.8}Na_{0.2}InCl_6$	10	0
44.	$Cs_2AgInCl_6$	18	0
45.	Cs_2KInCl_6	16	0
46.	$Cs_2AgSbCl_6$	0	0
47.	$PA_4AgBiBr_8$	08	0
48.	$BA_4AgInCl_8$	22	0
49.	$BA_4AgBiBr_8$	12	0
50.	$EA_2CsAgBiBr_7$	0	49
51.	$EA_2CsAg_{0.95}Na_{0.05}BiBr_7$	0	73
52.	$EA_2CsAg_{0.9}Na_{0.1}BiBr_7$	0	55
53.	$PA_2CsAgBiBr_7$	0	74
54.	$PA_2CsAg_{0.95}Na_{0.05}BiBr_7$	6	0
55.	$PA_2CsAg_{0.9}Na_{0.1}BiBr_7$	0	55
56.	$BA_2CsAgBiBr_7$	0	71
57.	$BA_2CsAg_{0.95}Na_{0.05}BiBr_7$	0	80
58.	$BA_2CsAg_{0.9}Na_{0.1}BiBr_7$	0	50
59.	$BA_2CsAg_{0.75}Na_{0.25}BiBr_7$	0	52
60.	$BA_2CsAg_{0.25}Na_{0.75}BiBr_7$	16	5
61.	$BA_2CsAgBiI_7$	10	12
62.	$BA_2CsAgBiBrI_6$	22	34
63.	$BA_2CsAgBiBr_2I_5$	14	25
64.	$BA_2CsAgBiBr_3I_4$	22	15
65.	$BA_2CsAgBiBr_4I_3$	20	14
66.	$BA_2CsAgBiBr_5I_2$	8	7
67.	$BA_2CsAgBiBr_6I$	22	14
68.	$BA_2CsAg_{0.95}Na_{0.05}BiBr_6I$	22	18
69.	$BA_2CsAg_{0.95}Na_{0.05}BiCl_6Br$	0	29
70.	$BA_2CsAg_{0.95}K_{0.05}BiBr_7$	0	31
71.	$BA_2CsAg_{0.95}Cu(I)_{0.05}BiBr_7$	6	0
72.	$BA_2CsAg_{0.95}Li_{0.05}BiBr_7$	12	0
73.	$BA_{1.5}Cs_{1.5}Ag_{0.95}Na_{0.05}BiBr_7$	0	52
74.	$PEA_2CsAgBiBr_7$	2	53
75.	$PEA_2CsAg_{0.95}Na_{0.05}BiBr_7$	0	60
76.	$BA_2CsAgSbBr_7$	4	0
77.	$BA_2CsAg_{0.95}Na_{0.05}SbBr_7$	10	0
78.	$BA_2CsAg_{0.9}Na_{0.1}SbBr_7$	12	0
79.	$Cs_4MnSb_2Cl_{12}$	2	0

80.	$Cs_2Cu_{0.2}Mn_{0.8}Sb_2Cl_{12}$	0	0
81.	$Cs_2Cu_{0.4}Mn_{0.6}Sb_2Cl_{12}$	4	0
82.	$Cs_2Cu_{0.5}Mn_{0.5}Sb_2Cl_{12}$	10	0
83.	$Cs_2Cu_{0.6}Mn_{0.4}Sb_2Cl_{12}$	0	0
84.	$Cs_2Cu_{0.8}Mn_{0.2}Sb_2Cl_{12}$	0	0
85.	$Cs_4CuSb_2Cl_{12}$	0	0

Condition: [a] **1a** (60 µl, 0.524 mmol), catalyst (5 mol%), DCE (3 mL), O_2 (1 atm), RT, blue light. [b] Reaction was conducted for 96 h.

Table S4. Examples of the electronegativity parameter definitions for the ML studies.

No.	Perovskites	A_1	A_2	A_{10}	B_1	B_2	B_{10}	B_3	B_4	X_1	X_2	X_{10}
1.	$FASnCl_3$	0.9	0.9	0	1.96	1.96	0	1.96	1.96	3.16	3.16	0
2.	$Cs_3Bi_2Cl_9$	0.79	0.79	0	2.02	2.02	0	2.02	2.02	3.16	3.16	0
3.	$Cs2AgBiCl_6$	0.79	0.79	0	1.93	1.93	0	2.02	2.02	3.16	3.16	0
4.	$Cs_2Ag_{0.05}Na_{0.95}BiCl_6$	0.79	0.79	0	0.93	1.93	0.05	2.02	2.02	3.16	3.16	0
5.	$PA_4AgBiBr_8$	0.9	0.9	0	1.93	1.93	0	2.02	2.02	2.96	2.96	0
6.	$BA_2CsAgBiBr7$	0.9	0.79	0.33	1.93	1.93	0	2.02	2.02	2.96	2.96	0
7.	$BA_2CsAg_{0.95}Na_{0.05}BiBr_7$	0.9	0.79	0.33	1.93	0.93	0.05	2.02	2.02	2.96	2.96	0
8.	$Cs_4MnSb_2Cl_{12}$	0.79	0.79	0	1.55	1.55	0	2.05	2.05	3.16	3.16	0
9.	$Cs_4Cu_{0.2}Mn_{0.8}Sb_2Cl_{12}$	0.79	0.79	0	1.55	1.9	0.2	2.05	2.05	3.16	3.16	0

Table S5. Analysis of the conduction bands (CBs) of $Cs_2AgBiBr_6$, $BA_2CsAgBiBr_7$, and $BA_2CsAg_pNa_{1-p}BiBr_7$, where p = 0.875.

	Number of $[B^IBr_6]^{5-}$ (B^I = Ag or Na) and $[BiBr_6]^{3-}$ octahedra per unit	Energy range of CB (eV)	Number of states in CB (states)	Normalized number of states in CB (states)[1]
$Cs_2AgBiBr_6$	8	0.61 to 2.88	23.4	5.8
$BA_2CsAgBiBr_7$	4	1.04 to 2.84	11.6	5.8
$BA_2CsAg_pNa_{1-p}BiBr_7$	16	1.18 to 2.98	48.0	6.0

[1]For quantitative comparison despite the unequal number of atoms in a unit cell used for calculations for the three perovskites, the number of states in the CB for each perovskite was normalized to represent that of a perovskite unit consisting of one $[B^IBr_6]^{5-}$ and one $[BiBr_6]^{5-}$ octahedron because these are the atoms that constitute majority of the CB.

References

[1] P. Vishnoi, R. Seshadri, A. K. Cheetham, *J. Phys. Chem. C* **2021**, *125*, 11756-11764.
[2] a) P. Giannozzi, S. Baroni, N. Bonini, M. Calandra, R. Car, C. Cavazzoni, D. Ceresoli, G. L. Chiarotti, M. Cococcioni, I. Dabo, *J. Phys.: Condens. Matter* **2009**, *21*, 395502; bJ. P. Perdew, K. Burke, M. Ernzerhof, *Phys. Rev. Lett.* **1996**, *77*, 3865.
[3] K. F. Garrity, J. W. Bennett, K. M. Rabe, D. Vanderbilt, *Comput. Mater. Sci.* **2014**, *81*, 446-452.
[4] S. Grimme, J. Antony, S. Ehrlich, H. Krieg, *J. Chem. Phys.* **2010**, *132*.
[5] a) A. Togo, I. Tanaka, *arXiv preprint arXiv:1808.01590* **2018**; b) Y. Hinuma, G. Pizzi, Y. Kumagai, F. Oba, I. Tanaka, *Comput. Mater. Sci.* **2017**, *128*, 140-184.
[6] K. Momma, F. Izumi, *J. Appl. Crystallogr.* **2011**, *44*, 1272-1276.
[7] M. e. Frisch, G. Trucks, H. Schlegel, G. Scuseria, M. Robb, J. Cheeseman, G. Scalmani, V. Barone, G. Petersson, H. Nakatsuji, Gaussian, Inc., Wallingford CT, **2016**.
[8] a) C. Lee, W. Yang, R. G. Parr, *Phys. Rev. B.* **1988**, *37*, 785; b) A. D. Becke, *Phys. Rev. A* **1988**, *38*, 3098.

Author Contributions

Y.F.L, F.G., and H.S.S. conceived and designed the project. H.S.S. supervised the project. Y.X. and X.H. performed the perovskite mechanosynthesis, structural characterizations, and data analysis. K.C., A.T., and M.S. performed the photocatalytic reactions and data analyses. V.C. and Y.F.L. obtained the hyperspectral imaging and PL results. Y.X., Y.F.L., and H.S.S. discussed and analyzed the parameters of the ML's model. Y.F.L. conducted the ML calculations. K.C., W.X.C., Y.H.T., R.G.L, S.N.B.S.M., K.J.Y.L., and J.Z.T. assisted in the mechanosyntheses of the perovskites. W.X.C. and T.S.C. conducted the DFT calculations. A.T. performed the mechanistic studies. M.S. completed the substrate scope. Y.X., K.C., W.X.C., A.T., M.S., and H.S.S. discussed the results and prepared the manuscript. Y.X., K.C., W.X.C., A.T., and M.S. were involved in revising the figures. All the authors reviewed and contributed to this paper.
